# Continuous Enantioselective α-Alkylation of Ketones via Direct Photoexcitation

**DOI:** 10.1021/acs.joc.4c00759

**Published:** 2024-06-10

**Authors:** Michael Weiser, Ádám Márk Pálvölgyi, Matthias Weil, Katharina Bica-Schröder

**Affiliations:** † Institute of Applied Synthetic Chemistry, TU Wien, 1060 Vienna, Austria; ‡ Institute of Chemical Technologies and Analytics, TU Wien, 1060 Vienna, Austria

## Abstract

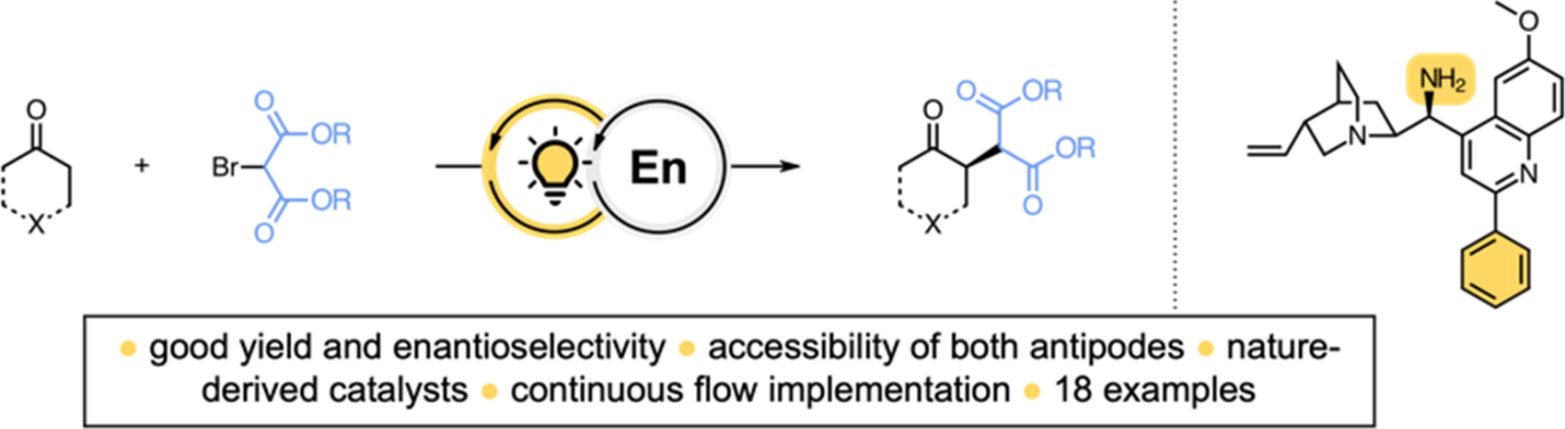

Motivated by the scarcity of enantioselective direct intermolecular α-alkylation reactions of ketones with simple alkyl halides, we report a photo-organocatalytic process to access diethyl 2-(2-oxocyclohexyl)malonate and derivatives in good yield and enantioselectivity. The reaction design is based on highly abundant and nature-derived 9-amino-9-deoxy-*epi*-cinchona alkaloids to activate ketones as transient secondary enamines, which exist unfavorably in equilibrium with imines. These condensed species can serve as powerful photoinitiators via direct photoexcitation. This concept provides access to both enantiomeric antipodes. In addition to introducing an uncomplicated batch-optimized procedure, we investigated the feasibility and limitations of implementing the reaction in continuous flow, thus enabling to obtain diethyl 2-(2-oxocyclohexyl)malonate with a productivity of 47 μmol/h and 84% enantioselectivity.

## Introduction

The α-alkylation of aldehydes and ketones is among the most crucial carbon–carbon bond-forming reactions in organic chemistry and indispensable for synthesizing numerous biologically active compounds and natural products.^1−4^ In many cases, stereocontrol is needed to force the formation of one defined product. Significant efforts have been made to develop enantioselective methods, primarily relying on chiral auxiliaries.^5^ Aiming to solve the main limitations associated with the use of chiral auxiliaries such as stoichiometric reagents and multiple-step procedures, catalytic methods to target the enantioselective α-alkylation of unfunctionalized aldehydes and ketones aroused particular interest. With a view to requirements in modern drug synthesis, organocatalysis offers a potent toolbox of highly enantioselective activation modes, using metal-free, nontoxic, and benign catalysts under mild conditions.^6^ Enamine catalysis enabled versatile advances in constructing α-stereogenic centers via nucleophilic addition to unsaturated electrophiles (e.g., aldol, Michael, and Mannich reaction).^7^ Although highly desirable, using simple alkyl halides for the direct enantioselective α-alkylation of aldehydes and ketones via an intermolecular S_N_2 mechanism has been particularly limited to phase transfer catalysis.^8−10^

The facile accessibility of reactive open-shelled species under mild conditions enabled by photochemistry guided new methodologies. Combining enamine catalysis with photochemically generated open-shelled species led to the development of versatile methods to target otherwise impossible transformations.^11^ While aldehydes have been frequently used as pronucleophiles for photo- and enamine catalytic enantioselective α-alkylation reactions with simple alkyl halides, there are far fewer examples for the significantly less reactive and more challenging ketones.^12^ Pioneering studies of Melchiorre et al. showed the ability of enamines to undergo direct excitation upon irradiation, respectively, to form excitable electron donor acceptor (EDA) complexes. These photochemically active intermediates were used to construct α-stereogenic centers of aldehydes with dialkyl 2-bromomalonates and (phenylsulfonyl)alkyl iodides, respectively, with benzyl and phenacyl bromides.^13−15^ The same group extended the application of EDA complexes to cyclic ketones, albeit limited to highly activated benzyl and phenacyl bromides.^16^ Moreover, Melchiorre et al. developed an enantioselective α-alkylation protocol for cyclic ketones, employing ground-state secondary enamines to trap alkyl radicals generated through dithiocarbamate catalyst-activated alkyl electrophiles upon irradiation.^17^

Herein, we present a photo-organocatalytic method for the direct enantioselective α-alkylation of secondary enamine-activated cyclic ketones with dialkyl 2-bromomalonates in batch and continuous flow. Cinchona alkaloid-based primary amino catalysts were used to generate light-absorbing species upon condensation with cyclic ketones, which were explored as potent photoinitiators via direct photoexcitation.

## Results and Discussion

The outstanding success of enantioselective enamine catalysis of unfunctionalized aldehydes is owed to chiral secondary amino catalysts, primarily based on amino acid moieties, e.g., l-proline. However, it has been shown that the enamine formation of sterically demanding aldehydes and, especially, ketones with secondary amines tend to be hindered.^18^ This limitation was overcome by using primary amino catalysts instead—most prominently, 9-amino-9-deoxy-*epi*-cinchona alkaloids. The flexibility of primary amines facilitates the formation of an imine via an acid-catalyzed condensation, which exists in equilibrium with an enamine. Although less favored in the equilibrium, the primary amine-based enamine can interact with numerous electrophiles and effectively activate even ketones in the α-position.^19^ Concerning photochemical reactions, Melchiorre et al. explored two distinct mechanisms by which enamines actively generate light-induced radicals, either via EDA complex formation or direct photoexcitation. In the latter case, the enamine must fulfill sufficient reductive properties to induce an efficient single electron transfer (SET) between its excited state and a quenching electrophile.^20^ Since dialkyl 2-bromomalonates have proven as easily reducible electrophiles for secondary amine-based enamines of aldehydes,^13^ we considered them as suitable alkylating agents for primary amine-based enamines of ketones to participate in the same reaction. Furthermore, we restricted the substrate scope to symmetric ketones and dialkyl 2-bromomalonates to avoid regiochemical issues and the generation of diastereomers and chose cyclohexanone and diethyl 2-bromomalonate as model substrates.

**Figure 1:**
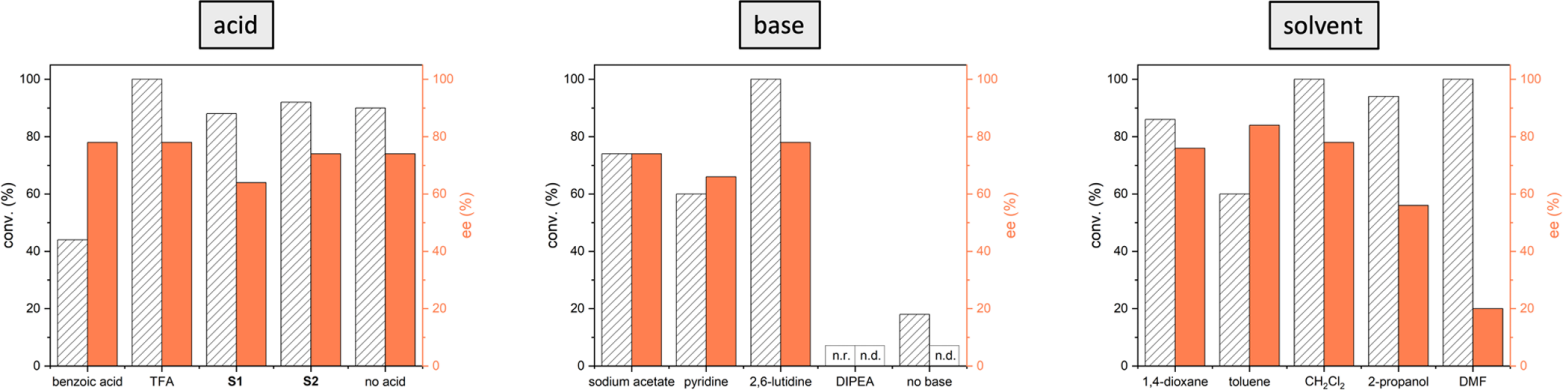
Optimization of the reaction conditions. All experiments were performed with **Qn** (20 mol %), cyclohexanone (2.0 equiv), diethyl 2-bromomalonate (1.0 equiv), TFA (40 mol %), and 2,6-lutidine (2.0 equiv) in CH_2_Cl_2_ (0.2 M, 1.0 mL), 36 W LED (365 nm), 25 °C, 18 h unless otherwise indicated.

We could show the feasibility of the concept by investigating initial reaction conditions based on 9-amino-9-deoxy-*epi*-cinchona alkaloids as catalysts. Several parameters (*catalyst*, *acid*, *base*, and *solvent*) were varied to find reaction conditions that maximize the conversion and enantioselectivity of diethyl 2-(2-oxocyclohexyl)malonate (**P1**) as shown in Table 1 and Figure 1. Based on our initial consideration, we showed that secondary amino catalysts (Table 1, entries 1 and 2) could not condense with cyclohexanone and could not promote the transformation. Also, the dual activation mode via enamine and *H*-bond catalysis (Table 1, entry 3) failed. Pure primary amines (Table 1, entry 4) and mainly primary amines with an adjacent tertiary amine functionality (Table 1, entry 5) showed promising reactivity. However, among all screened catalysts, 9-amino-9-deoxy-*epi*-cinchona alkaloids (Table 1, entries 6–9) performed the best, with **Qn** showing the most promising result. Notably, the nature-derived primary amines **Qn** and **Qd** and their related **Cd** and **Cn** are pseudo-enantiomers and provide access to both enantiomers of **P1**.

**Table 1:**
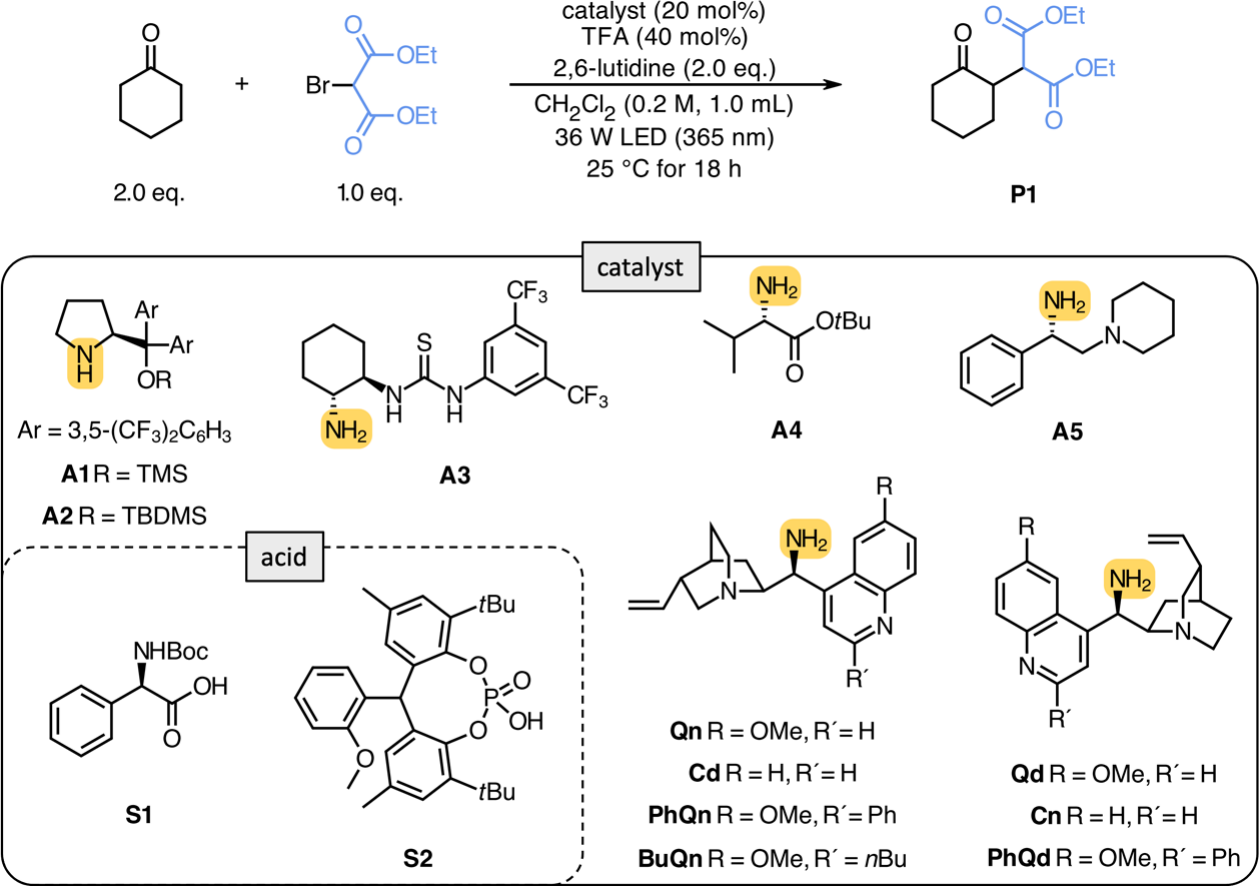
Catalyst Screening^a^

entry	catalyst	conv. (%)^b^	ee (%)^c^
1	**A1**	<5	n.d.
2	**A2**	<5	n.d.
3	**A3**	<5	n.d.
4	**A4**	60	14
5	**A5**	62	64
6	**Qn**	>95	78
7	**Cd**	>95	76
8	**Qd**	72	72
9	**Cn**	70	74

a All reactions were performed with catalyst (20 mol %), cyclohexanone (2.0 equiv), diethyl 2-bromomalonate (1.0 equiv), TFA (40 mol %), and 2,6-lutidine (2.0 equiv) in CH_2_Cl_2_ (0.2 M, 1.0 mL), 36 W LED (365 nm), 25 °C, 18 h.

b Determined by GCMS analysis.

c Determined for the crude products by chiral HPLC analysis on an IA-3 column.

It has been shown that an acidic cocatalyst is, on the one hand, needed to promote the condensation reaction between the primary amino catalyst and cyclohexanone. Still, on the other hand, the nature of its counterion also affects the spatial arrangement of the condensed species, as it surrounds the protonated quinuclidine moiety.^18^ Considering these aspects, different acids were screened (Figure 1, left). However, the weaker benzoic acid, the chiral *N*-protected d-phenylglycine (**S1**), and the sterically demanding and flexible phosphoric acid (**S2**) led to inferior results than trifluoroacetic acid (TFA). Interestingly, and in contrast to previous findings,^16^ there was only a minor deterioration if no acid was used. Still, the reaction required the presence of a base to scavenge the *in situ* generated hydrobromic acid, whereas different bases were screened (Figure 1, middle). The decreased steric demand of pyridine or sodium acetate led to minor results compared to 2,6-lutidine. Notably, the aliphatic amine *N*,*N*-diisopropylethylamine (DIPEA) prohibited the reaction, which can be rationalized by generating *N*-centered radical cations and subsequent side reactions.^21^ Since the dielectric constant of the solvent can significantly affect the ability of charged species to induce chirality,^22^ we screened different solvents (Figure 1, right). While 2-propanol and dimethylformamide (DMF), solvents with a higher dielectric constant compared to CH_2_Cl_2_, quantitatively promote the transformation, they lack enantiocontrol. On the other hand, more apolar solvents like 1,4-dioxane and toluene caused a decrease in conversion but not inevitably increased stereoselectivity. However, we observed that the rather apolar toluene led to a significant increase in enantioselectivity.

To increase the conversion in the case of toluene as solvent, we aimed to change the activity of **Qn** by modifying it either with a *n*Bu (**BuQn**) or a Ph (**PhQn**) group in the 2′-position. Interestingly, **BuQn** and **PhQn** (Table 2, entries 2 and 3) showed no significant improvement concerning the conversion and enantioselectivity compared to **Qn** (Table 2, entry 1) if CH_2_Cl_2_ was used as solvent. However, if the reaction was performed in toluene as solvent, **BuQn** and, significantly, **PhQn** (Table 2, entries 5 and 6) outperformed **Qn** (Table 2, entry 4) since a quantitative conversion and improved stereoselectivity were found. The optimized conditions stated in entry 6 of Table 2 allowed for the isolation of **P1** in good yield and enantioselectivity (78% isolated yield, 86% ee). The other enantiomeric antipode could be obtained using **PhQd** instead (34% isolated yield, 79% ee). Notably, CH_2_Cl_2_ provided homogeneous conditions throughout the reaction time, whereas the *in situ* formed bromide salt precipitated if toluene was used as solvent.

**Table 2:** Screening of 2′ Derivatized Cinchona Alkaloids^a^

entry	catalyst	solvent	conv. (%)^b^	ee (%)^c^
1	**Qn**	CH_2_Cl_2_	>95	78
2	**BuQn**		>95	78
3	**PhQn**		>95	78
4	**Qn**	toluene	60	84
5	**BuQn**		>95	84
6	**PhQn**		>95	86

a All reactions were performed with catalyst (20 mol %), cyclohexanone (2.0 equiv), diethyl 2-bromomalonate (1.0 equiv), TFA (40 mol %), and 2,6-lutidine (2.0 equiv) in solvent (0.2 M, 1.0 mL), 36 W LED (365 nm), 25 °C, 18 h.

b Determined by GCMS analysis.

c Determined for the crude products by chiral HPLC analysis on an IA-3 column.

**Scheme 1:**
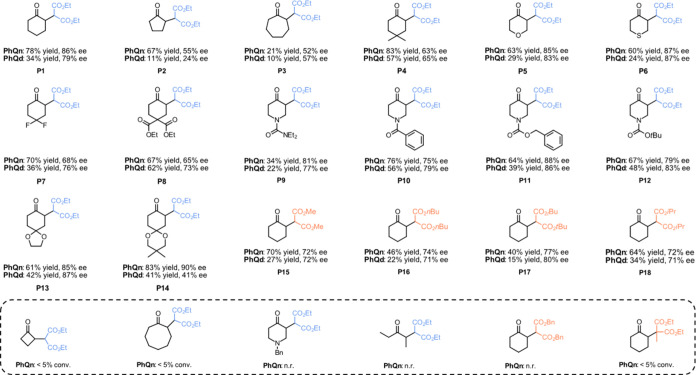
Substrate Scope All reactions were performed with **PhQn** or **PhQd** (20 mol %), ketone (2.0 equiv), dialkyl 2-bromomalonate (1.0 equiv), TFA (40 mol %), and 2,6-lutidine (2.0 equiv) in toluene (0.2 M, 1.0 mL), 36 W LED (365 nm), 25 °C, 18 h. Yield refers to isolated products, and enantiomeric excess was determined for the crude products by chiral HPLC on an IA-3 column.

With these optimized reaction conditions in hand, we demonstrated the synthetic potential of the developed photo-organocatalytic method for the enantioselective α-alkylation of unfunctionalized ketones with dialkyl 2-bromomalonates (Scheme 1). To evaluate the scope, we probed differently substituted cyclic and symmetric ketones **P2–14** and symmetric dialkyl 2-bromomalonates **P15–18**. Furthermore, all experiments were carried out for both pseudo-enantiomeric catalysts **PhQn** and **PhQd**, providing easy access to both enantiomeric antipodes in good yields and enantioselectivities. We observed an increased activity for **PhQn** compared to **PhQd** since all compounds were isolated with higher yields. However, no preferred catalyst could be observed in terms of enantioselectivity. When comparing different ring sizes of cycloalkanones, **P1** provided better results than **P2** and **P3**, probably attributed to a more efficient enamine orbital overlap.^23^ This also accounts for the inferior conversion of four- and eight-membered cycloalkanones. The steric and electronic properties accompanied by the geminal methyl groups of **P4** at the 4-position positively influenced the activity, but a lower enantioselectivity was observed compared to **P1**. Gratifyingly, *O*- and *S*-heterocycles (**P5** and **P6**) were well tolerated. However, *N*-heterocycles with a nonconjugated lone pair at the *N*-atom showed no reactivity, probably due to the same considerations as for the aliphatic base DIPEA.^21^ This theory is supported by the finding of good yields and enantioselectivities when using nonbasic *N*-functionalized substrates, including urea, carbamate, and amide moieties (**P9**-**P12**). Regarding functional group tolerance, electron-withdrawing substituents (**P7** and **P8**) and electron-donating ketals (**P13** and **P14**) at the 4-position did not reduce the efficiency of the transformation. Despite showing great tolerance for various 6-membered cyclic ketones, the catalyst system—in accordance with literature examples for enamine photocatalysis^16^—failed to activate linear ketones. Moreover, we observed that methyl (**P15**), as well as larger substituents (**P16**-**P18**) at the 2-bromomalonate, led to worse activity and stereoselectivity compared to ethyl substituents (**P1**). Inferior conversion was observed for the sterically more hindered diethyl 2-bromo-2-methylmalonate, and no reactivity was observed for dibenzyl 2-bromomalonate.

Furthermore, we observed that all α-alkylated products underwent mild racemization due to the acidic environment in the silica-stationary phase during the isolation process via flash chromatography. Different strategies to circumvent the acidic conditions (e.g., Celite, neutral aluminum oxide, or triethylamine-neutralized silica) led to no improvement. Thus, a minor loss of enantioselectivity had to be accepted for all α-alkylated products except for **P7** and **P9**-**P12**, which could only be isolated as racemates.

**Scheme 2:**
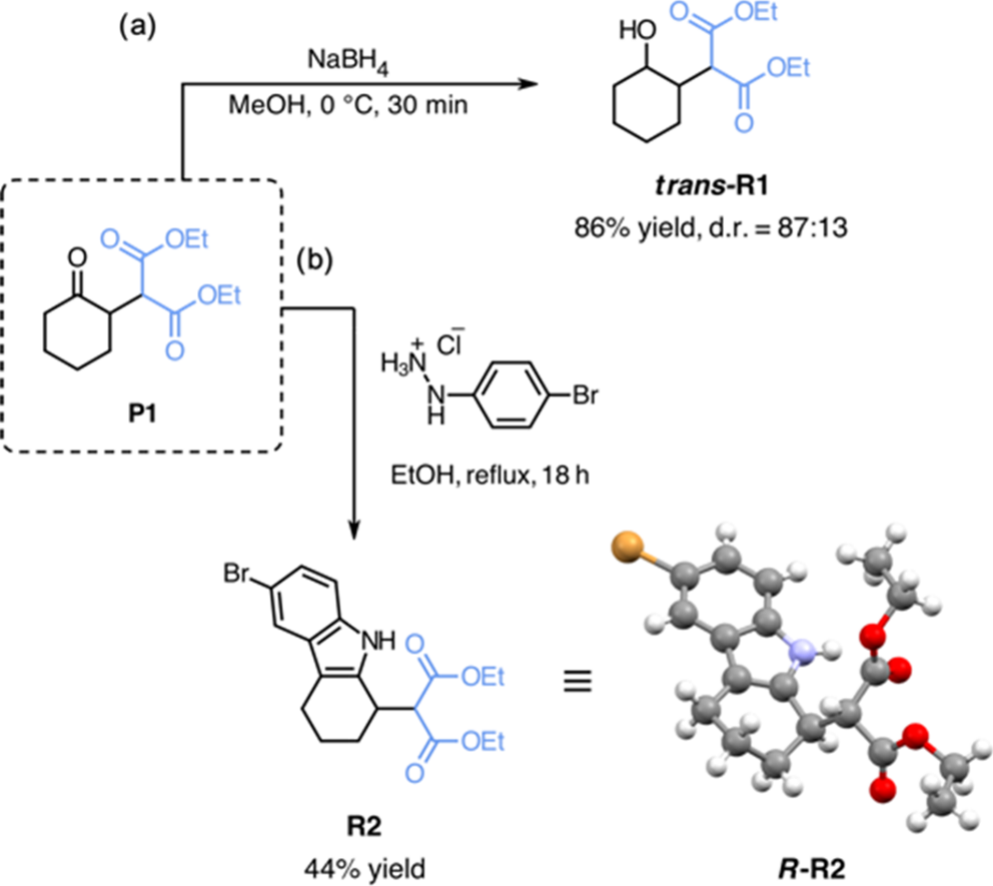
Subsequent Reactions of P1 The reactions were performed (a) using **P1** (1.0 equiv) and NaBH_4_ (1.5 equiv) in methanol (MeOH) (0.25 M, 2.0 mL), 0 °C, 30 min; (b) using **P1** (1.0 equiv) and 4-bromophenylhydrazine hydrochloride (1.1 equiv) in ethanol (EtOH) (0.25 M, 4.0 mL), reflux, 18 h. The deposition number of the XRD of ***R*****-R2** is CCDC 2313387.^26^

To demonstrate the synthetic utility of the obtained α-alkylated products, we examined their feasibility in subsequent transformations (Scheme 2). Among the screened reaction types, ketone reduction to afford ***trans*****-R1** and Fischer indole synthesis to afford **R2** worked the best, providing first access to possibly valuable intermediates.^24,25^

Aiming for further mechanistic insights, we performed control experiments as presented in Table 3. The addition of 2,2,6,6-tetramethylpiperidine-1-oxyl (TEMPO) (Table 3, entry 1) to the reaction mixture prohibited any formation of **P1**; thus, a radical mechanism was assumed.^27^ Furthermore, a significant decrease in productive reactivity was observed in the presence of molecular oxygen (Table 3, entry 2), further underlining the presence of a radical mechanism.^28^ An excess of water (Table 3, entry 3) disturbed the product formation but did not lead to complete inhibition. The exclusion of any irradiation (Table 3, entry 4) prohibited any product formation. To determine the influence of thermal activation, the reaction mixture was refluxed in CH_2_Cl_2_ (Table 3, entry 5) without any irradiation. Also, to consider a potentially higher activation barrier, the reaction mixture was refluxed in the higher-boiling solvent 1,4-dioxane (Table 3, entry 6). However, in both cases, no formation of **P1** was observed, thus verifying a purely photochemical transformation.

**Table 3:** Control Experiments^a^

entry	additives and conditions	conv. (%)^b^	ee (%)^c^
1	TEMPO (2.0 equiv)	n.r.	n.d.
2	air atmosphere	16	n.d.
3	water (20.0 equiv)	34	68
4	dark	n.r.	n.d.
5^d^	CH_2_Cl_2_ (reflux)	n.r.	n.d.
6^d^	1,4-dioxane (reflux)	n.r.	n.d.

a All reactions were performed with **Qn** (20 mol %), cyclohexanone (2.0 equiv), diethyl 2-bromomalonate (1.0 equiv), TFA (40 mol %), and 2,6-lutidine (2.0 equiv) in CH_2_Cl_2_ (0.2 M, 1.0 mL), 36 W LED (365 nm), 25 °C, 18 h plus the respective additive and/or condition deviation.

b Determined by GCMS analysis.

c Determined for the crude products by chiral HPLC analysis on an IA-3 column.

d Thermal reactions were performed in the dark and by refluxing the reaction mixture in the respective solvent.

**Figure 2:**
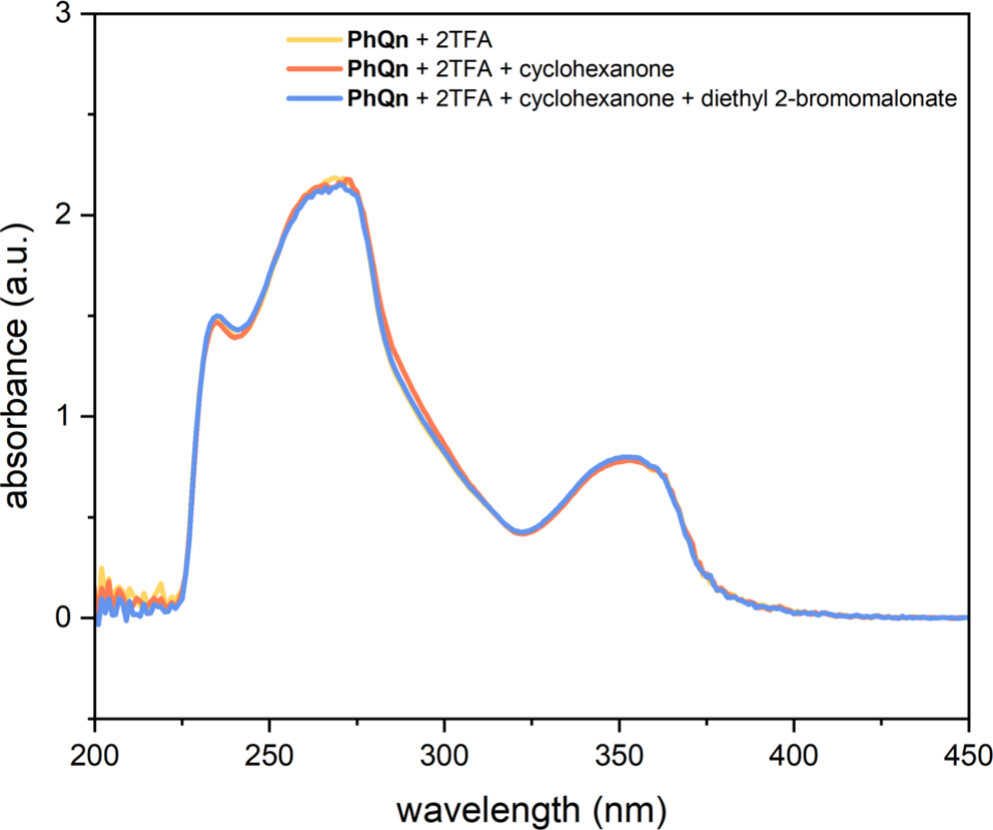
UV/vis absorption spectra of different mixtures. The spectra were recorded at a concentration of 0.1 mM for each component, respectively, 0.2 mM for TFA in CH_2_Cl_2_. Prior to measurement, 2.0 mL of the mixtures was dried over 50 mg of anhydrous MgSO_4_ at 25 °C for 10 min and filtrated.

Since the formation of an EDA complex can be easily verified by the appearance of a charge-transfer band, which cannot be observed for the individual components,^29^ UV/vis spectra of different component mixtures were measured (Figure 2). For a solution of **PhQn** · 2TFA, a broad absorption maximum at about 350 nm was measured, revealing the need for a small irradiation wavelength. An equimolar mixture of **PhQn** · 2TFA and cyclohexanone shows an identical absorbance to the pure catalyst, indicating a domination of the free catalyst over any condensed species. This behavior was confirmed by *in situ* NMR studies in CD_2_Cl_2_ (for details, see the SI). Nevertheless, *in situ* NMR studies in DMSO-*d*_6_ confirmed the marginal formation of an imine upon combining **PhQn** and cyclohexanone, which is known to exist in an unfavorable equilibrium with a secondary enamine.^18,30^ The potential for forming condensed species implies that they are also generated to a lesser extent in CH_2_Cl_2_. This finding, combined with the mere presence of enantioenriched **P1**, emphasizes the indispensability of transient enamine formation with the chiral primary amino catalyst for activating the α-position of cyclohexanone and facilitating the subsequent enantioselective reaction. However, this unfavored yet mandatory enamine formation renders its isolation and any further experiments (e.g., cyclic voltammetry and Stern–Volmer quenching) difficult if not impossible. The absorbance of a **PhQn** · 2TFA, cyclohexanone, and diethyl 2-bromomalonate mixture causes no deviation and is dominated by the free catalyst. Due to its marginal detectability, we cannot entirely dismiss the possibility of an EDA complex formation between the transient enamine and the alkyl halide. However, in accordance with the literature, as related structures of secondary amine-based enamines of aldehydes do not form an EDA complex with diethyl 2-bromomalonate,^13^ we propose that direct photoexcitation serves as the decisive mechanism. Notably, as we are unable to differentiate between the photochemical properties of the imine and the enamine, we cannot state with certainty which of the two species is responsible for initiating the reaction.

With these assumptions of a radical mechanism caused via direct photoexcitation, we further investigated the possibility of a self-propagating radical chain mechanism or radical coupling taking place. The light on/off experiment (for details, see the SI) demonstrated that **P1** was only formed during the on-periods. However, this behavior does not exclude the potential for a self-propagating radical chain mechanism, as propagating radicals can be promptly terminated upon cessation of irradiation.^31^ The most precise method to differentiate between the potential mechanisms is to determine the quantum yield. The ferrioxalate actinometer was selected for the determination of the photon flux of the photoreactor, and a quantum yield of 0.63 was measured for the transformation (for details, see the SI). Despite the quantum yield being lower than 1, this does not exclude a self-propagating radical chain mechanism because of nonproductive processes taking place, e.g., an inefficient initiation step.^31^

**Scheme 3:**
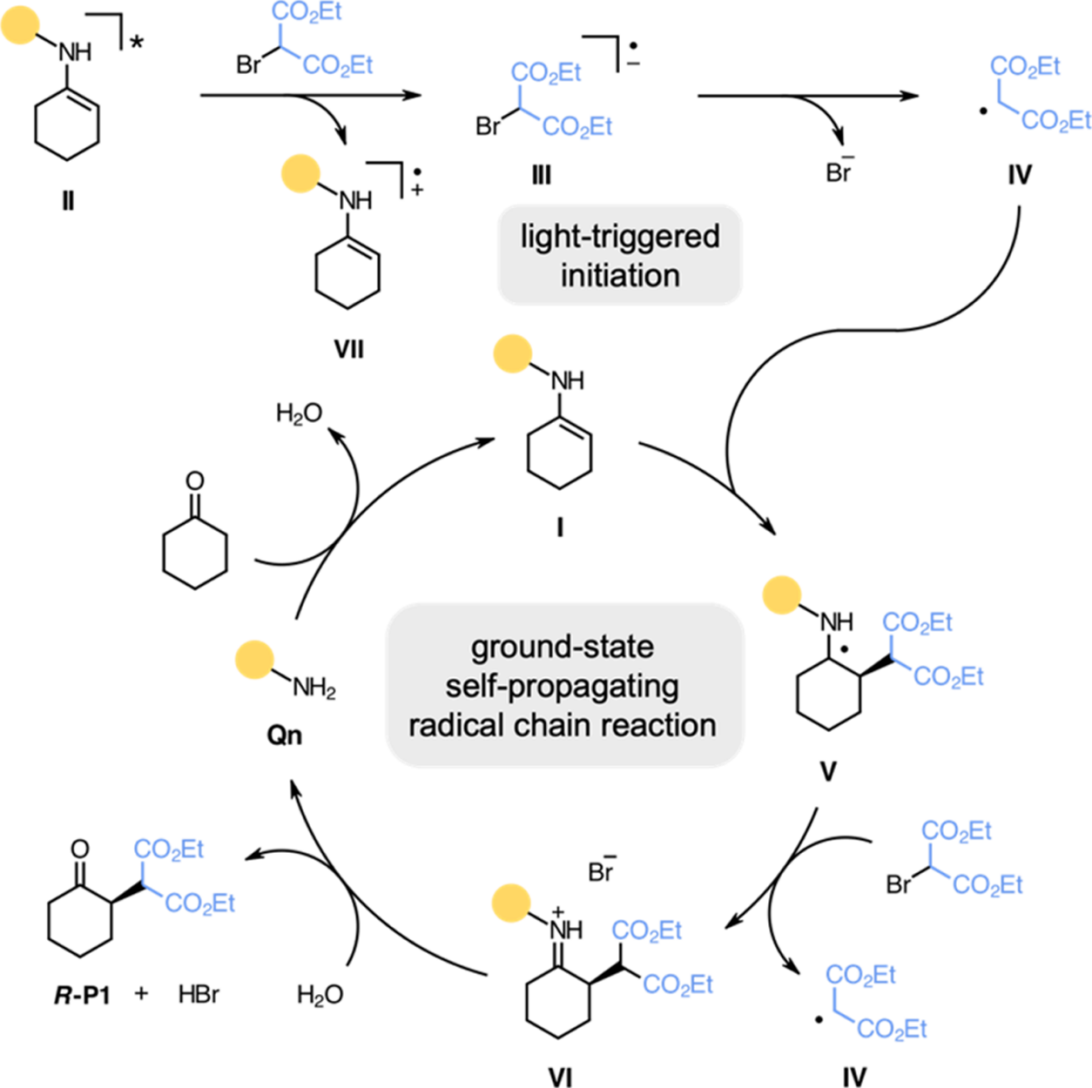
Proposed Mechanism

Based on these findings, we assume that the unfavored condensation renders the initiation step inefficient and propose that a self-propagating radical chain mechanism is initiated via direct photoexcitation (Scheme 3). A part of the transient enamine (**I**) or imine (not shown) formed by condensation of cyclohexanone with **Qn** reaches its excited state (**II**) upon irradiation and is readily quenched by diethyl 2-bromomalonate. The thereby formed alkyl bromide radical anion (**III**) cleaves off the leaving group, and the alkyl radical (**IV**) is enantioselectivity intercepted by the ground-state enamine (**I**). The α-amino radical (**V)** triggers the reductive cleavage of diethyl 2-bromomalonate, regenerating the propagating radical by forming an iminium ion (**VI**). Upon hydrolysis, **Qn** is regenerated, and ***R*****-P1** is released. Notably, the initiation step triggered by the excited enamine (**II**) or imine (not shown) results in the formation of either an enamine (**VII**) or imine radical cation (not shown) via SET, whose destiny is not connected to any product-forming processes; hence, the excited condensed species serve as sacrificial initiators.

**Figure 3:**
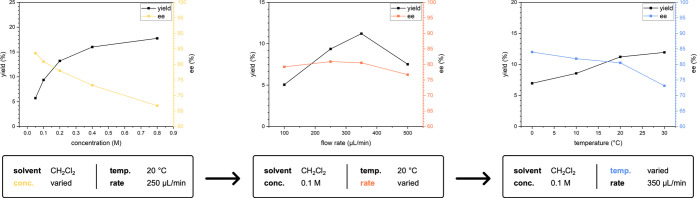
Parameter optimization in a 10 mL photoreactor. All experiments were performed with **PhQn** (20 mol %), cyclohexanone (2.0 equiv), diethyl 2-bromomalonate (1.0 equiv), TFA (40 mol %), and 2,6-lutidine (2.0 equiv) in CH_2_Cl_2_ (conc. M, 2.0 mL), 62 W LED (365 nm), rate μL/min, temp. °C. The yield was determined by GC analysis using *n*-dodecane as internal standard, and the ee was determined for the crude product by chiral HPLC analysis on an IA-3 column.

Given that photochemical batch processes cannot be efficiently scaled up by simply enlarging the volume, and due to beneficial effects with regard to process control,^32^ we aimed for the implementation of the developed photo-organocatalytic reaction into continuous flow. The optimized batch reaction conditions that used toluene as solvent could not be transferred to continuous flow due to the precipitation of the *in situ* formed 2,6-lutidinium hydrobromide, which caused blockage of the tubing. Thus, we first screened different solvents that provided homogeneous conditions throughout the reaction and observed that CH_2_Cl_2_ offered the best trade-off between yield and enantiocontrol. To iteratively optimize the conditions, we varied three process parameters: (i) concentration, (ii) flow rate, and (iii) temperature (Figure 3). As expected, increasing the concentration of the reaction mixture is known to increase the reaction rate and, thus, the yield. However, this increase in yield comes at the cost of enantioselectivity. That is why, we considered 0.1 M the best compromise for further optimization. Interestingly, when the flow rate is varied, the yield and enantioselectivity reach a maximum of approximately 350 μL/min. On the one hand, low yields were observed due to the limited mixing that accompanies laminar flow regimes at low flow rates.^33^ On the other hand, high flow rates caused the reagents to be washed out of the photoreactor before they had a chance to react. A medium flow rate of 350 μL/min appears to provide both a sufficiently long residence time and suitable circulation, which are inevitable for the reaction. When the temperature was varied, we observed an inverse relation between the yield and enantioselectivity. Since we prioritized the enantioselectivity over the yield, a temperature of 10 °C was chosen as the ideal reaction temperature. We achieved a good yield and productivity under optimized conditions for a reactor size of 10 mL while maintaining the enantioselectivity at levels comparable to those obtained under batch conditions (Table 4, entry 1).

**Table 4:**
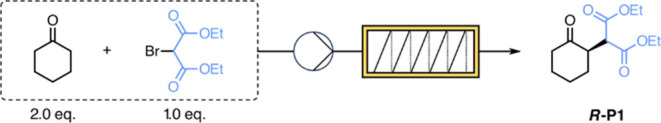
Comparison of Reactor Volumes^a^

entry	*V*_R_ (mL)	yield (%)^d^	prod. (μmol/h)	ee (%)^e^
1^b^	10	9	36	82
2^c^	25	28	47	84

a All experiments were performed with **PhQn** (20 mol %), cyclohexanone (2.0 equiv), diethyl 2-bromomalonate (1.0 equiv), TFA (40 mol %), and 2,6-lutidine (2.0 equiv) in CH_2_Cl_2_ (0.1 M, 2.0 mL), 350 μL/min, 10 °C.

b 62 or

c 73.8 W LED (365 nm).

d Determined by GC analysis using *n*-dodecane as internal standard.

e Determined for the crude product by chiral HPLC analysis on an IA-3 column.

Since the insufficient mixing at low flow rates did not allow for running the reaction at an increased residence time using the given reactor size of 10 mL, we decided to investigate the impact on the residence time by enlarging the reactor volume (*V*_R_). Therefore, we switched to a photoreactor with an increased volume of 25 mL and iteratively optimized the process parameters (for details, see the SI). Although the used photoreactors differed in construction properties, we observed a comparable trend in behavior when the temperature, flow rate, and concentration were varied. Under the same optimized conditions as for the smaller *V*_R_, the results of the larger *V*_R_ are presented in Table 4, entry 2. Comparing the results obtained for the two photoreactors, the longer residence time positively impacted the yield, and maintaining the same flow rate while increasing *V*_R_ still resulted in increased productivity.

## Conclusions

In summary, we developed a photo-organocatalytic method for the direct enantioselective α-alkylation of cyclic ketones with dialkyl 2-bromomalonates. 9-Amino-9-deoxy-*epi*-cinchona alkaloids were used to activate ketones as transient secondary enamines, which exist unfavorably in equilibrium with imines. These condensed species were explored as potential powerful photoinitiators *via* direct photoexcitation, facilitating a ground-state self-propagating radical chain mechanism upon 365 nm irradiation. With optimized conditions in hand, we showed the versatility of the developed catalytic system by reacting a range of dialkyl 2-bromomalonates with diverse substituted cyclic ketones in batch, throughout with good yields and enantioselectivities. To meet the requirements of modern synthesis,^34−36^ we furthermore investigated the performance of the developed method for the enantioselective photo-organocatalytic α-alkylation of cyclohexanone with diethyl 2-bromomalonate in continuous flow. We observed a concentration- and temperature-dependent trade-off between yield and enantioselectivity. Furthermore, we identified the insufficient mixing at low flow rates, respectively, at high residence times, as the main limitation for the efficient production of **P1** to combine a high yield, productivity, and enantioselectivity.

## Experimental Section

### General Procedure for the Batch Synthesis of α-Alkylated Ketones

All batch photoreactions were conducted in a custom-made 36 W (365 nm) photoreactor that was cooled by a beside-positioned fan to ensure ambient temperature. Into an 8 mL Schlenk tube, a mixture of **PhQn** or **PhQd** (0.04 mmol, 0.2 equiv), TFA (0.08 mmol, 0.4 equiv), ketone (0.40 mmol, 2.0 equiv), dialkyl 2-bromomalonate (0.20 mmol, 1.0 equiv), and 2,6-lutidine (0.40 mmol, 2.0 equiv), dissolved in anhydrous toluene (0.2 M considering the limiting component, 1.0 mL) was added under Ar counterflow using standard Schlenk technique. The sealed Schlenk tube was positioned into the photoreactor, and the reaction mixture was irradiated at 25 °C for 18 h while stirring. After completion, two identical parallel runs were merged, and an aliquot was taken for chiral HPLC measurement. After the evaporation of the reaction mixture, the crude product was isolated *via* flash chromatography.

### General Procedure for the Flow Synthesis of **P1**

The flow photoreactions were conducted using a Vaportec E-Series flow machine equipped with a Vaportec UV-150 coiled photochemical reactor module (10 mL, 62 W, 365 nm) or a custom-made coiled photochemical reactor module (25 mL, 73.8 W, 365 nm). For example, for a concentration of 0.1 M, a mixture of **PhQn** or **PhQd** (0.04 mmol, 0.2 equiv), TFA (0.08 mmol, 0.4 equiv), cyclohexanone (0.40 mmol, 2.0 equiv), diethyl 2-bromomalonate (0.20 mmol, 1.0 equiv), and 2,6-lutidine (0.40 mmol, 2.0 equiv), dissolved in anhydrous CH_2_Cl_2_ (0.1 M considering the limiting component, 2.0 mL) was added into a septum-closed vial under Ar counterflow using standard Schlenk technique. One end of tubing was introduced through the septum into the reaction mixture, and the other end was connected to the flow machine. After conditioning with CH_2_Cl_2_, setting the temperature, and switching on the irradiation, the reaction mixture was pumped at a certain flow rate through the coiled photochemical reactor. An internal standard was added to the collected reaction mixture, and an aliquot was taken for GC and chiral HPLC measurement.

## Data Availability

The data underlying this study are available in the published article and its Supporting Information.

## Supporting Information Available

Detailed experimental, analytical, and crystal data and copies of spectra and chromatograms (PDF)
