# Supplementary material for: Continuous Enantioselective α-Alkylation of Ketones via Direct Photoexcitation
Source: J Org Chem. 2024 Jun 10;89(12):8906–14. doi: 10.1021/acs.joc.4c00759 (PMC11197082; doi:10.1021/acs.joc.4c00759)
Supplement: Supplementary file 1 — jo4c00759_si_001.pdf [file jo4c00759_si_001.pdf]

Supporting Information

# Continuous Enantioselective $\alpha$-Alkylation of Ketones *via* Direct Photoexcitation

Michael Weiser,[a] Ádám Márk Pálvölgyi,[a] Matthias Weil,[b] and Katharina Bica-Schröder[a,*]

[a]Institute of Applied Synthetic Chemistry, TU Wien, 1060 Vienna, Austria

[b]Institute of Chemical Technologies and Analytics, TU Wien, 1060 Vienna, Austria

*Corresponding author: Katharina Bica-Schröder. E-mail: katharina.schroeder@tuwien.ac.at,

Tel.: +43 1 58801 163601

# Table of Contents


1. General remarks ..... S3

2. Catalyst synthesis ..... S5

2.1. Synthesis of *L*-proline derived secondary amino catalysts ..... S5

2.2. Synthesis of dual enamine and *H*-bond catalyst ..... S8

2.3. Synthesis of 9-amino-9-deoxy-*epi*-cinchona alkaloids ..... S9

2.4. Synthesis of 2´-derived 9-amino-9-deoxy-*epi*-cinchona alkaloids ..... S11

3. Substrate Synthesis ..... S15

3.1. Synthesis of 4-piperidone-derived ketones ..... S15

3.2. Synthesis of dialkyl 2-bromomalonates ..... S17

4. Enantioselective $\alpha$-alkylation of unfunctionalized ketones *via* direct photoexcitation ..... S19

4.1. Batch reactor set-up and general considerations ..... S19

4.2. Synthesis and analytical data of $\alpha$-alkylated ketones ..... S20

4.3. Large-scale experiment for the synthesis of P1 ..... S30

5. Control experiment with TEMPO ..... S31

6. Subsequent reactions from P1 ..... S32

6.1. Ketone reduction ..... S32

6.2. Fischer indole synthesis ..... S33

6.3. *In-situ* derivatization ..... S34

7. Continuous flow implementation ..... S35

7.1. Flow reactor set-up and general considerations ..... S35

7.2. Optimization for the 25 mL photoreactor ..... S37

8. Light on/off experiment ..... S38

9. NMR Experiments to prove PhQn condensation ..... S39

10. Quantum yield determination ..... S41

10.1. Synthesis of potassium ferrioxalate trihydrate ..... S41

10.2. Determination of the photon flux ..... S41

10.3. Determination of the quantum yield ..... S43

11. Structure determination of *R*-R2 by single-crystal X-ray diffraction ..... S45

11.1. Experimental ..... S45

12. Abbreviations ..... S47

13. NMR Spectra ..... S48

14. Chiral HPLC Chromatograms ..... S93

15. References ..... S103

# 1. General remarks

All chemicals used for synthesis were purchased from commercial suppliers with a purity greater than 95% or were available in the laboratory and were used without further purification. Special thanks to Fabian Scharinger for providing substances **A4**, **A5**, **S1** and **S2**.[1, 2] Solvents were used as follows:

- Anhydrous solvents: Dichloromethane (DCM), toluene, tetrahydrofuran (THF), 1,4-dioxane, and methanol (MeOH) were pre-distilled and desiccated on aluminum oxide using a PureSolv Innovative-Technology® purification system.
- Other solvents were technical grade and purchased from commercial suppliers.

The reaction heating (metal block), stirring and temperature control were conducted using VELP Scientifica® Arex-6 digital PRO hot plate stirrers.
Manual glass columns and Merck® silica (40 to 60 µm) were used for preparative flash chromatography, and the column size and the eluent were adapted to the respective separation problem. Solvent compositions for elution or reaction purposes are stated as volume ratios. TLC analysis for reaction and elution monitoring was performed using precoated aluminum-backed silica 60 F254 plates purchased from Merck®. The spot visualization was achieved using UV light (254 nm) for UV-active compounds and staining solutions for non-UV active compounds. Staining agents were used as follows:

- Anisaldehyde: Ethanol (135 mL) plus concentrated sulfuric acid (5 mL) plus glacial acetic acid (1.5 mL) plus *p*-anisaldehyde (3.7 mL).
- Ninhydrin: *n*-Butanol (100 mL) plus ninhydrin (1.5 g) plus concentrated acetic acid (3 mL).
- Vanillin: Ethanol (250 mL) plus vanillin (15 g) plus concentrated sulfuric acid (2.5 mL).

NMR spectra were recorded at 297 K on a Bruker® Advance UltraShield ($^{1}$H: 400 MHz, $^{13}$C{$^{1}$H}: 101 MHz,$^{19}$F{$^{1}$H}: 376 MHz) spectrometer. Chemical shifts (δ) were reported in ppm with tetramethylsilane as an internal standard by calibration to the respective solvent signal and coupling constants (*J*) were reported in Hz. Deuterated solvents were purchased from commercial suppliers and stored over molecular sieve. The following abbreviations are used in the NMR codes to explain multiplicities: s (singlet), d (doublet), t (triplet), q (quartet), combinations thereof, and m (multiplet). Structural assignments were made with additional information from COSY, HSQC, and HMBC experiments.

GC measurements were performed on a Thermo Scientific® Focus equipped with a flame ionization detector on a BGB5 column.

GCMS measurements were performed on a Thermo Scientific® DSQ II equipped with a quadrupole MS detector on a BGB5 column.

Chiral HPLC measurements were performed on a Thermo Scientific® DIONEX UPLC equipped with a photodiode array detector (190 to 360 nm) on columns from Chiralcel, Daicel® AS-H, and IA-3 (all 250 × 4.60 mm, 5 µm).

Infrared spectra were recorded at 297 K on a Perkin Elmer® Spectrum 65 FT-IR spectrometer equipped with a Specac® MK II Golden Gate Single Reflection ATR unit.

UV/Vis spectra were recorded at 297 K on a Shimadzu® UV/Vis 1800 spectrometer using 1 cm path-length quartz cuvettes.

High-resolution MS measurements were performed using a CTC® HTCPAL system autosampler, an Agilent® 1100/1200 high-performance liquid chromatograph, and an Agilent® 6230 AJS ESI-time-of-flight mass spectrometer.

Specific optical rotations were recorded on an Anton Paar® MCP500 polarimeter at 297 K with the concentration stated for the specific entries.

# 2. Catalyst synthesis

## 2.1. Synthesis of *L*-proline derived secondary amino catalysts

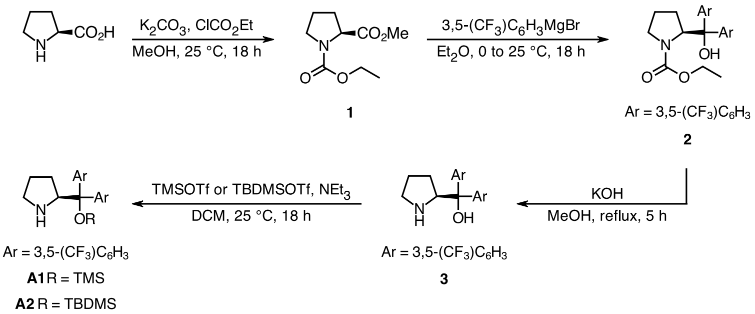


<u>(*S*)-Proline-*N*-ethyl carbamate methyl ester (**1**)</u>

*L*-Proline (3.0 g, 26.1 mmol, 1.0 eq.) and $K_2CO_3$ (3.61 g, 26.1 mmol, 1.0 eq.) were suspended in MeOH (40 mL). Ethyl chloroformate (6.23 g, 57.4 mmol, 2.2 eq.) was added, and the suspension was stirred at 25 °C for 18 h. After evaporating the reaction mixture, the residue was dissolved in distilled $H_2O$ and DCM. After the phase separation, the aqueous phase was extracted three times with DCM. The combined organic phases were washed with brine, dried over anhydrous $MgSO_4$, filtrated, and evaporated. **1** was obtained as a colorless liquid (5.21 g, 99% yield).

**$^1$H NMR** (400 MHz, $CDCl_3$) δ 4.40 – 4.26 (m, 1H), 4.20 – 4.01 (m, 2H), 3.75 – 3.69 (m, 3H), 3.64 – 3.40 (m, 2H), 2.30 – 2.12 (m, 1H), 2.05 – 1.82 (m, 3H), 1.31 – 1.15 (m, 3H).

Analytical data was in accordance with literature.[3]

<u>(*S*)-2-[Bis(3,5-bis(trifluoromethyl)phenyl)hydroxymethyl]pyrrolidine-1-carboxylic acid ethyl ester (**2**)</u>

Mg (2.84 g, 117 mmol, 3.5 eq.) was suspended in anhydrous diethyl ether (110 mL). 1-Bromo-3,5-bis(trifluoromethyl)benzene (25.4 g, 86.8 mmol, 2.6 eq.) was dissolved in anhydrous diethyl ether (30 mL) and dropwise added while stirring so that the reaction mixture was kept at reflux. The reaction mixture was refluxed for 2 h, cooled to 0 °C, and a solution of **1** (6.72 g, 33.4 mmol, 1.0 eq.) in anhydrous diethyl ether (30 mL) was added dropwise while stirring. The reaction mixture was stirred at 25 °C for 18 h and then quenched with saturated aq. $NH_4Cl$. After the phase separation, the organic phase was washed with brine, and the combined aqueous phases were extracted three times with chloroform. The combined organic phases were dried over anhydrous $MgSO_4$, filtrated, and evaporated. The crude product was crystallized from methylcyclohexane. **2** was obtained as a red solid (16.2 g, 81% yield).

**$^1$H NMR** (400 MHz, $CDCl_3$) δ 7.89 (s, 1H), 7.86 (s, 3H), 7.82 (s, 2H), 7.00 (s, 1H), 4.90 – 4.81 (m, 1H), 4.25 – 4.04 (m, 2H), 3.59 – 3.49 (m, 1H), 2.99 – 2.88 (m, 1H), 2.17 – 2.03 (m, 1H), 1.85 – 1.74 (m, 1H), 1.71 – 1.58 (m, 1H), 1.22 (t, *J* = 7.1 Hz, 3H), 1.06 – 0.97 (m, 1H).

Analytical data was in accordance with literature.[4]

<u>(*S*)-Bis(3,5-bis(trifluoromethyl)phenyl)(pyrrolidin-2-yl)methanol (**3**)</u>

**2** (16.1 g, 27.0 mmol, 1.0 eq.) was dissolved in KOH in MeOH (2 M, 450 mL) and refluxed for 5 h. The reaction mixture was evaporated, and the residue was dissolved in distilled $H_2O$ and DCM. After the phase separation, the aqueous phase was extracted three times with DCM, and the combined organic phases were washed with brine, dried over anhydrous $MgSO_4$, filtrated, and evaporated. **3** was obtained as a brown solid (13.2 g, 93% yield).

**$^1$H NMR** (400 MHz, $CDCl_3$) δ 8.04 (s, 2H), 7.96 (s, 2H), 7.77 (s, 1H), 7.76 (s, 1H), 5.06 (s, 1H), 4.35 (t, *J* = 7.7 Hz, 1H), 3.13 – 2.99 (m, 2H), 1.82 – 1.50 (m, 5H).

Analytical data was in accordance with literature.[4]

<u>(*S*)-2-{Bis[3,5-bis(trifluoromethyl)phenyl][(trimethylsilanyl)oxy]methyl}pyrrolidine (**A1**)</u>

**3** (4.00 g, 7.61 mmol, 1.0 eq.) was dissolved in anhydrous DCM (50 mL), followed by the addition of $NEt_3$ (1.00 g, 9.89 mmol, 1.3 eq.). After cooling the solution to 0 °C, TMSOTf (2.20 g, 9.89 mmol, 1.3 eq.) was dropwise added during vigorous stirring. The reaction mixture was stirred at 25 °C for 18 h and then quenched with distilled $H_2O$. After the phase separation, the aqueous phase was extracted three times with DCM. The combined organic phases were dried over anhydrous $MgSO_4$, filtrated, and evaporated. The crude product was purified *via* flash chromatography (silica, $Et_2O$:PE = 1:10, Ninhydrin staining agent). **A1** was obtained as a yellow liquid (1.97 g, 43% yield).

**$^1$H NMR** (400 MHz, $CDCl_3$) δ 8.00 (s, 2H), 7.84 (s, 1H), 7.83 (s, 1H), 7.76 (s, 2H), 4.22 (t, *J* = 7.4 Hz, 1H), 2.92 (dt, *J* = 10.3, 6.3 Hz, 1H), 2.56 (dt, *J* = 10.3, 6.7 Hz, 1H), 1.74 – 1.65 (m, 1H), 1.59 – 1.49 (m, 2H), 1.48 – 1.40 (m, 1H), 1.15 – 1.07 (m, 1H), -0.09 (s, 9H).

**$^{13}$C{$^1$H} NMR** (101 MHz, $CDCl_3$) δ 148.3 (s), 146.5 (s), 131.7 (q, *J* = 33.3 Hz), 131.0 (q, *J* = 33.2 Hz), 128.7 (d, *J* = 4.0 Hz), 128.2 (d, *J* = 3.8 Hz), 123.5 (q, *J* = 272.8 Hz), 123.3 (q, *J* = 272.8 Hz), 121.9 – 121.8 (m), 121.8 – 121.6 (m) , 82.4 (s), 64.4 (s), 47.4 (s), 27.7 (s), 25.4 (s), 2.0 (s).

**$^{19}$F{$^1$H} NMR** (376 MHz, $CDCl_3$) δ -62.8 (s), -62.9 (s).

The analytical data was in accordance with literature.[5]

<u>(*S*)-2-{Bis[3,5-bis(trifluoromethyl)phenyl][(*tert*-butyldimethylsilyl)oxy]methyl}pyrrolidine (**A2**)</u>

**3** (4.00 g, 7.61 mmol, 1.0 eq.) was dissolved in anhydrous DCM (50 mL), followed by the addition of $NEt_3$ (1.00 g, 9.89 mmol, 1.3 eq.). After cooling the solution to 0 °C, TBDMSOTf (6.03 g, 22.8 mmol, 3.0 eq.) was dropwise added during vigorous stirring. The reaction mixture was stirred at 25 °C for 18 h and then quenched with distilled $H_2O$. After the phase separation, the aqueous phase was extracted three times with DCM. The combined organic phases were dried over anhydrous $MgSO_4$, filtrated, and evaporated. The crude product was purified *via* flash chromatography (silica, $Et_2O$:PE = 1:20, Ninhydrin staining agent). **A2** was obtained as a yellow liquid (1.50 g, 31% yield).

**$^1$H NMR** (400 MHz, $CDCl_3$) δ 8.09 (s, 2H), 7.85 (s, 2H), 7.74 (s, 2H), 4.30 – 4.18 (m, 1H), 2.97 – 2.84 (m, 1H), 2.53 (ddd, *J* = 10.1, 6.9, 5.3 Hz, 1H), 1.84 – 1.73 (m, 2H), 1.60 – 1.43 (m, 3H), 0.94 (s, 9H), -0.21 (s, 3H), -0.47 (s, 3H).

**$^{13}$C{$^1$H} NMR** (101 MHz, $CDCl_3$) δ 147.9 (s), 146.2 (s), 131.7 (q, *J* = 33.3 Hz), 130.7 (q, *J* = 33.2 Hz), 129.2 (d, *J* = 3.8 Hz), 128.9 (d, *J* = 3.9 Hz), 123.5 (q, *J* = 272.7 Hz), 123,3 (q, *J* = 272.8 Hz), 122.1 – 121.8 (m), 121.8 – 121.5 (m), 82.4 (s), 64.1 (s), 47.4 (s), 28.0 (s), 26.0 (s), 25.4 (s), 19.0 (s), -2.6 (s), -3.3 (s).

**$^{19}$F{$^1$H} NMR** (376 MHz, $CDCl_3$) δ -62.8 (s), -62.9 (s).

Analytical data was in accordance with literature.[6]

## 2.2. Synthesis of dual enamine and *H*-bond catalyst

1-((1*R*,2*R*)-2-Aminocyclohexyl)-3-(3,5-bis(trifluoromethyl)phenyl)thiourea (**A3**)

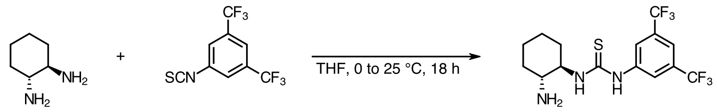

**A3**

(1*R*,2*R*)-1,2-Diaminocyclohexane (0.57 g, 5.00 mmol, 1.0 eq.) was dissolved in anhydrous THF (25 mL). At 0 °C, 3,5-ditrifluoromethylisocyanate (1.28 g, 5.00 mmol, 1.0 eq.) was slowly added during vigorous stirring. The reaction mixture was stirred at 25 °C for 18 h and evaporated. The crude product was purified *via* flash chromatography (silica, MeOH:DCM = 1:10, Vanillin staining agent). **A3** was obtained as a white solid (1.73 g, 90% yield).

**$^1$H NMR** (400 MHz, DMSO-d$_6$) δ 8.24 (s, 2H), 7.66 (s, 1H), 3.85 (s, 1H), 2.61 – 2.52 (m, 1H), 2.15 – 1.95 (m, 1H), 1.91 – 1.80 (m, 1H), 1.72 – 1.54 (m, 2H), 1.33 – 1.00 (m, 4H).

**$^{19}$F{$^1$H} NMR** (376 MHz, DMSO-d$_6$) δ -61.7 (s).

Analytical data was in accordance with literature.[7]

## 2.3. Synthesis of 9-amino-9-deoxy-*epi*-cinchona alkaloids

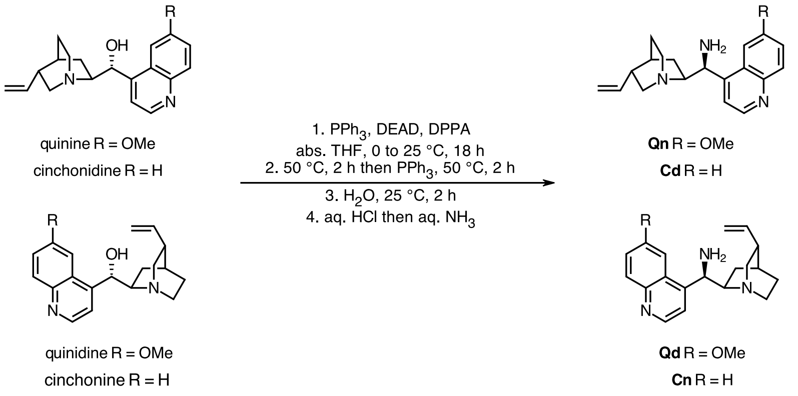


General Procedure for Mitsunobu-Staudinger reaction of cinchona alkaloids (GP1)

According to a modified literature procedure,[8] the corresponding 9-amino-9-deoxy-*epi*-cinchona alkaloids (**Qn**, **Cd**, **Qd** and **Cn**) were synthesized on a 4.0 to 10.0 mmol scale. The cinchona alkaloid (1.0 eq.) and $PPh_3$ (1.2 eq.) were dissolved in anhydrous THF (0.2 M). The solution was cooled to 0 °C, and DEAD (1.2 eq.) was added while stirring, followed by the dropwise addition of DPPA (1.2 eq.). The yellow solution was stirred at 25 °C for 18 h and then at 50 °C for 2 h. $PPh_3$ (2.0 eq.) was added in portions at 50 °C under the generation of $N_2$. The yellow solution was stirred at 50 °C for 2 h, distilled $H_2O$ (10 mL) was added at 25 °C, and the reaction mixture was stirred at 25 °C for 2 h. The reaction mixture was evaporated, and the residue was dissolved in aq. HCl (10%, 50 mL) and DCM (50 mL). After phase separation, the aqueous phase was extracted three times with DCM. To the aqueous phase, DCM and aq. $NH_3$ (5 M) was added until a pH of 9 was reached. After the phase separation, the aqueous phase was extracted three times with DCM. The combined organic phases were washed with brine, dried over anhydrous $MgSO_4$, filtrated, and evaporated. The brown crude product was purified *via* flash chromatography (silica, EtOAc:MeOH:$NEt_3$ = 50:50:1, Ninhydrin staining agent).

9-amino-9-deoxy-*epi*-quinine (**Qn**)

Prepared according to the general procedure (GP1) from quinine (3.24 g, 10.0 mmol), affording **Qn** as a yellow liquid (2.59 g, 80% yield).

**$^1$H NMR** (400 MHz, $CDCl_3$) δ 8.75 (d, *J* = 4.5 Hz, 1H), 8.03 (d, *J* = 9.2 Hz, 1H), 7.66 (s, 1H), 7.46 (d, *J* = 4.6 Hz, 1H), 7.39 (dd, *J* = 9.2, 2.7 Hz, 1H), 5.80 (ddd, *J* = 17.5, 10.3, 7.5 Hz, 1H), 5.06 – 4.92 (m, 2H), 4.60 (d, *J* = 10.2 Hz, 1H), 3.97 (s, 3H), 3.28 (dd, *J* = 13.8, 10.0 Hz, 1H), 3.25 – 3.17 (m, 1H), 3.13 – 3.03 (m, 1H), 2.86 – 2.75 (m, 2H), 2.34 – 2.24 (m, 1H), 1.95 (s, 2H), 1.66 – 1.60 (m, 1H), 1.60 – 1.51 (m, 2H), 1.49 – 1.37 (m, 1H), 0.81 – 0.73 (m, 1H).

Analytical data was in accordance with literature.[9]

9-amino-9-deoxy-*epi*-cinchonidine (**Cd**)

Prepared according to the general procedure (GP1) from cinchonidine (1.18 g, 4.00 mmol), affording **Cd** as a yellow liquid (0.71 g, 61% yield).

**$^1$H NMR** (400 MHz, $CDCl_3$) δ 8.91 (d, *J* = 4.6 Hz, 1H), 8.36 (s, 1H), 8.14 (dd, *J* = 8.5, 1.3 Hz, 1H), 7.72 (ddd, *J* = 8.4, 6.8, 1.4 Hz, 1H), 7.60 (ddd, *J* = 8.3, 6.8, 1.4 Hz, 1H), 7.53 (d, *J* = 4.6 Hz, 1H), 5.81 (ddd, *J* = 17.5, 10.3, 7.5 Hz, 1H), 5.04 – 4.94 (m, 2H), 4.77 – 4.64 (m, 1H), 3.28 (dd, *J* = 13.8, 10.0 Hz, 1H), 3.24 – 3.16 (m, 1H), 3.13 – 3.01 (m, 1H), 2.86 – 2.76 (m, 2H), 2.34 – 2.23 (m, 1H), 2.06 (s, 2H), 1.65 – 1.50 (m, 3H), 1.47 – 1.37 (m, 1H), 0.80 – 0.70 (m, 1H).

Analytical data was in accordance with literature.[9]

9-amino-9-deoxy-*epi*-quinidine (**Qd**)

Prepared according to the general procedure (GP1) from quinidine (3.24 g, 10.0 mmol), affording **Qd** as a yellow liquid (1.52 g, 47% yield).

**$^1$H NMR** (400 MHz, $CDCl_3$) δ 8.75 (d, *J* = 4.6 Hz, 1H), 8.02 (d, *J* = 9.2 Hz, 1H), 7.61 (s, 1H), 7.53 (d, *J* = 4.6 Hz, 1H), 7.38 (dd, *J* = 9.2, 2.7 Hz, 1H), 5.89 (ddd, *J* = 17.1, 10.6, 6.5 Hz, 1H), 5.13 – 5.01 (m, 2H), 4.67 (d, *J* = 10.0 Hz, 1H), 3.97 (s, 3H), 3.11 – 2.88 (m, 5H), 2.35 – 2.23 (m, 1H), 1.94 (s, 2H), 1.66 – 1.49 (m, 3H), 1.20 – 1.10 (m, 1H), 1.01 – 0.90 (m, 1H).

Analytical data was in accordance with literature.[9]

9-amino-9-deoxy-*epi*-cinchonine (**Cn**)

Prepared according to the general procedure (GP1) from cinchonine (2.94 g, 10.0 mmol), affording **Cn** as a yellow liquid (1.68 g, 57% yield).

**$^1$H NMR** (400 MHz, $CDCl_3$) δ 8.90 (d, *J* = 4.5 Hz, 1H), 8.35 (s, 1H), 8.14 (dd, *J* = 8.3, 1.3 Hz, 1H), 7.72 (ddd, *J* = 8.4, 6.8, 1.4 Hz, 1H), 7.62 – 7.53 (m, 2H), 5.86 (ddd, *J* = 16.9, 10.8, 6.8 Hz, 1H), 5.12 – 5.02 (m, 2H), 4.83 – 4.69 (m, 1H), 3.11 – 2.89 (m, 5H), 2.33 – 2.21 (m, 1H), 1.91 (s, 2H), 1.63 – 1.50 (m, 3H), 1.17 – 1.06 (m, 1H), 1.00 – 0.89 (m, 1H).

Analytical data was in accordance with literature.[10]

## 2.4. Synthesis of 2´-derived 9-amino-9-deoxy-*epi*-cinchona alkaloids

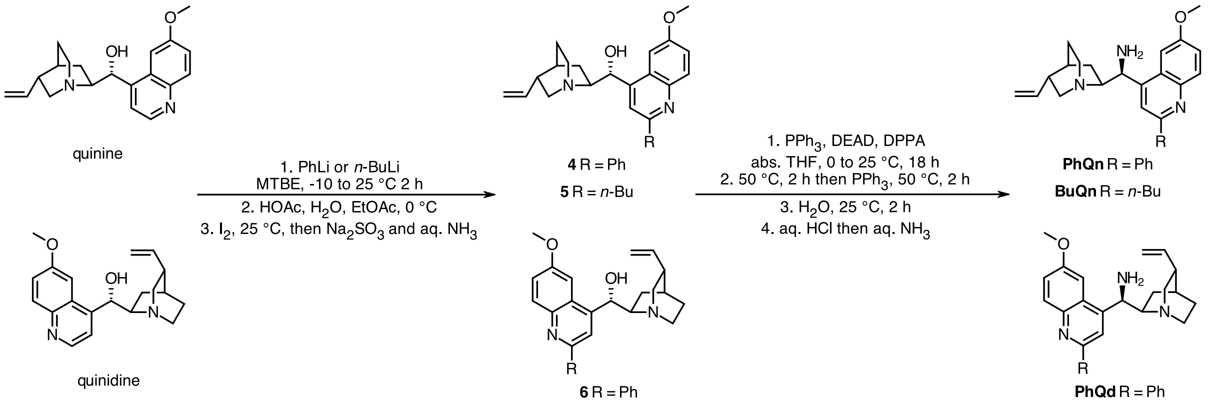


General Procedure for the alkylation of cinchona alkaloids with RLi reagents (GP2)

According to a modified literature procedure,[11] the corresponding 2´-derived cinchona alkaloids (**4**, **5** and **6**) were synthesized on a 10.0 to 15.0 mmol scale. The cinchona alkaloid (1.0 eq.) was suspended in anhydrous MTBE (0.2 M) under an Ar atmosphere. The suspension was cooled to -10 °C, and PhLi or *n*-BuLi (3.0 eq.) was added during vigorous stirring. The reaction mixture was stirred at -10 °C for 30 min and at 25 °C for 2 h. At 0 °C, concentrated acetic acid (5 mL) was slowly added during vigorous stirring, followed by the addition of distilled $H_2O$ (50 mL) and EtOAc (50 mL). At 25 °C, iodine was added until a dark color remained. To quench excess iodine, $Na_2SO_3$ was added until a yellow color remained. During vigorous stirring, aq. $NH_3$ (25%, 20 mL) was added, and the biphasic system was stirred at 25 °C for 10 min. After the phase separation, the organic phase was washed with brine, and the combined aqueous phases were extracted three times with DCM. The combined organic phases were dried over anhydrous $MgSO_4$, filtrated, and evacuated. The crude product was purified *via* flash chromatography (silica, PE:EtOAc:MeOH:$NEt_3$ = 8:1:0.5:0.5, Vanillin staining agent).

<u>2'-phenyl-quinine (**4**)</u>

Prepared according to the general procedure (GP2) from quinine (4.87 g, 15.0 mmol) and PhLi (23.7 mL, 1.9 M in dibutyl ether), affording **4** as a yellow solid (4.58 g, 76% yield).

**$^1$H NMR** (400 MHz, $CDCl_3$) δ 8.06 – 7.99 (m, 3H), 7.87 (s, 1H), 7.46 – 7.38 (m, 3H), 7.30 (dd, *J* = 9.2, 2.7 Hz, 1H), 7.13 (d, *J* = 2.7 Hz, 1H), 5.70 (ddd, *J* = 17.1, 10.3, 7.6 Hz, 1H), 5.51 (d, *J* = 3.7 Hz, 1H), 4.98 – 4.86 (m, 2H), 3.87 (s, 3H), 3.55 – 3.44 (m, 1H), 3.14 – 3.03 (m, 2H), 2.71 – 2.60 (m, 2H), 2.31 – 2.22 (m, 1H), 1.84 – 1.68 (m, 3H), 1.55 – 1.43 (m, 2H).

**$^{13}C\{^1H\}$ NMR** (101 MHz, $CDCl_3$) δ 157.8 (s), 154.6 (s), 148.2 (s), 144.4 (s), 141.9 (s), 139.7 (s), 131.9 (s), 129.1 (s), 128.8 (s), 127.4 (s), 125.6 (s), 121.8 (s), 116.3 (s), 114.6 (s), 101.3 (s), 72.2 (s), 60.1 (s), 57.2 (s), 55.9 (s), 43.4 (s), 40.0 (s), 28.0 (s), 27.7 (s), 21.6 (s).

Analytical data was in accordance with literature.[12]

2′-*n*-butyl-quinine (**5**)

Prepared according to the general procedure (GP2) from quinine (1.62 g, 5.00 mmol) and *n*-BuLi (9.4 mL, 1.6 M in hexane), affording **5** as a yellow liquid (0.67 g, 35% yield).

**$^1$H NMR** (400 MHz, $CDCl_3$) δ 7.92 (d, *J* = 9.2 Hz, 1H), 7.39 (s, 1H), 7.28 (dd, *J* = 9.2, 2.7 Hz, 1H), 7.16 (d, *J* = 2.8 Hz, 1H), 5.73 (ddd, *J* = 17.1, 10.3, 7.7 Hz, 1H), 5.47 (d, *J* = 4.0 Hz, 1H), 4.99 – 4.87 (m, 2H), 3.87 (s, 3H), 3.65 (s, 1H), 3.50 – 3.39 (m, 1H), 3.14 – 3.01 (m, 2H), 2.87 – 2.78 (m, 2H), 2.69 – 2.59 (m, 2H), 2.29 – 2.20 (m, 1H), 1.82 – 1.77 (m, 1H), 1.77 – 1.64 (m, 4H), 1.54 – 1.42 (m, 2H), 1.35 (sext, *J* = 7.4 Hz, 2H), 0.90 (t, *J* = 7.3 Hz, 3H).

**$^{13}$C{$^1$H} NMR** (101 MHz, $CDCl_3$) δ 160.3 (s), 157.2 (s), 147.9 (s), 144.1 (s), 142.1 (s), 131.0 (s), 125.0 (s), 121.2 (s), 118.6 (s), 114.4 (s), 101.5 (s), 72.4 (s), 60.1 (s), 57.3 (s), 55.8 (s), 43.4 (s), 40.2 (s), 39.0 (s), 32.4 (s), 28.1 (s), 27.9 (s), 22.8 (s), 21.8 (s), 14.1 (s).

Analytical data was in accordance with literature.[12]

2′-phenyl-quinidine (**6**)

Prepared according to the general procedure (GP2) from quinidine (3.24 g, 10.0 mmol) and PhLi (15.8 mL, 1.9 M in dibutyl ether), affording **6** as a yellow solid (2.86 g, 71% yield).

**$^1$H NMR** (400 MHz, $CDCl_3$) δ 8.10 – 8.03 (m, 3H), 7.93 (s, 1H), 7.51 – 7.38 (m, 3H), 7.32 (dd, *J* = 9.2, 2.7 Hz, 1H), 7.13 (d, *J* = 2.7 Hz, 1H), 6.10 – 5.96 (m, 1H), 5.61 (d, *J* = 4.1 Hz, 1H), 5.09 – 4.98 (m, 2H), 3.87 (s, 3H), 3.40 – 3.30 (m, 1H), 3.13 – 3.02 (m, 1H), 2.99 – 2.85 (m, 2H), 2.85 – 2.71 (m, 1H), 2.30 – 2.17 (m, 1H), 2.11 – 2.01 (m, 1H), 1.80 – 1.70 (m, 1H), 1.60 – 1.40 (m, 2H), 1.23 – 1.14 (m, 1H).

**$^{13}$C{$^1$H} NMR** (101 MHz, $CDCl_3$) δ 157.8 (s), 154.8 (s), 148.1 (s), 144.5 (s), 140.7 (s), 139.9 (s), 132.1 (s), 129.1 (s), 128.9 (s), 127.5 (s), 125.6 (s), 121.9 (s), 116.3 (s), 114.7 (s), 101.2 (s), 72.4 (s), 59.9 (s), 55.9 (s), 50.3 (s), 49.8 (s), 40.2 (s), 28.4 (s), 26.5 (s), 21.1 (s).

**HRMS** (ESI-TOF) m/z: $[M+H]^+$ calcd for C26H29N2O2 401.2223; found 401.2248.

**$[\alpha]_D^{25}$** +101.465 (c = 0.157 g/100mL, $CHCl_3$)

**IR** 2967, 2934, 2872, 1691, 1620, 1501, 1450, 1351, 1229, 1030 $cm^{-1}$

General Procedure for Mitsunobu-Staudinger reaction of 2´-derived cinchona alkaloids (GP3)

According to a modified literature procedure,[11] the corresponding 2´-derived 9-amino-9-deoxy-*epi*-cinchona alkaloids (**PhQn**, **BuQn** and **PhQd**) were prepared on a 1.0 to 10.0 mmol scale. The 2´-derived cinchona alkaloid (1.0 eq.) and $PPh_3$ (1.2 eq.) were dissolved in anhydrous THF (0.2 M). The solution was cooled to 0 °C, and DEAD (1.2 eq.) was added while stirring, followed by the dropwise addition of DPPA (1.2 eq.). The yellow solution was stirred at 25 °C for 18 h and then at 50 °C for 2 h. $PPh_3$ (2.0 eq.) was added in portions at 50 °C under the generation of $N_2$. The yellow solution was stirred at 50 °C for 2 h, distilled $H_2O$ (10 mL) was added at 25 °C, and the reaction mixture was stirred at 25 °C for 2 h. The reaction mixture was evaporated, and the residue was dissolved in HCl (10%, 50 mL) and DCM (50 mL). After phase separation, the aqueous phase was extracted three times with DCM. To the aqueous phase, DCM and aq. $NH_3$ (5 M) was added until a pH of 9 was reached. After the phase separation, the aqueous phase was extracted three times with DCM. The combined organic phases were washed with brine, dried over anhydrous $MgSO_4$, filtrated, and evaporated. The brown crude product was purified *via* flash chromatography (silica, EtOAc:MeOH:$NEt_3$ = 50:50:1, Ninhydrin staining agent).

(1*S*)-(6-methoxy-2-phenylquinolin-4-yl)((2*S*,4*S*,5*R*)-5-vinylquinuclidin-2-yl)methanamine (**PhQn**)

Prepared according to the general procedure (GP3) from **4** (4.01 g, 10.00 mmol), affording **PhQn** as a yellow solid (2.98 g, 75% yield).

| | |
|---|---|
| **$^1$H NMR** | (400 MHz, $CDCl_3$) δ 8.18 – 8.09 (m, 3H), 7.99 (s, 1H), 7.66 (s, 1H), 7.55 – 7.49 (m, 2H), 7.47 – 7.38 (m, 2H), 5.79 (ddd, *J* = 17.5, 10.3, 7.5 Hz, 1H), 5.05 – 4.92 (m, 2H), 4.75 – 4.60 (m, 1H), 3.98 (s, 3H), 3.31 (dd, *J* = 13.8, 10.0 Hz, 1H), 3.27 – 3.10 (m, 2H), 2.89 – 2.78 (m, 2H), 2.34 – 2.25 (m, 1H), 2.11 (s, 2H), 1.68 – 1.54 (m, 3H), 1.50 – 1.39 (m, 1H), 0.91 – 0.79 (m, 1H). |
| **$^{13}$C{$^1$H} NMR** | (101 MHz, $CDCl_3$) δ 157.7 (s), 155.0 (s), 147.8 (s), 145.0 (s), 141.9 (s), 134.0 (s), 132.3 (s), 129.1 (s), 128.9 (s), 127.8 (s), 127.5 (s), 121.4 (s), 118.0 (s), 114.5 (s), 102.2 (s), 62.6 (s), 56.5 (s), 55.7 (s), 41.2 (s), 40.0 (s), 28.4 (s), 27.7 (s), 26.1 (s). |
| **HRMS** | (ESI-TOF) m/z: $[M+H]^+$ calcd for C26H30N3O 400.2384; found 400.2416 m/z. |
| **$[\alpha]_D^{25}$** | +29.235 (c = 0.183 g/100mL, $CHCl_3$) |
| **IR** | 3382, 3293, 3071, 2939, 2861, 2099, 1620, 1479, 1353, 1224 $cm^{-1}$ |

Analytical data was in accordance with literature.[9]

(1*S*)-(2-butyl-6-methoxyquinolin-4-yl)((2*S*,4*S*,5*R*)-5-vinylquinuclidin-2-yl)methanamine (**BuQn**)

Prepared according to the general procedure (GP3) from **5** (0.38 g, 1.00 mmol), affording **BuQn** as a yellow solid (0.22 g, 58% yield).

| | |
|---|---|
| **$^1$H NMR** | (400 MHz, $CDCl_3$) δ 7.97 (d, *J* = 9.2 Hz, 1H), 7.59 (s, 1H), 7.40 – 7.30 (m, 2H), 5.80 (ddd, *J* = 17.6, 10.4, 7.6 Hz, 1H), 5.04 – 4.91 (m, 2H), 4.64 – 4.50 (m, 1H), 3.94 (s, 3H), 3.27 (dd, *J* = 13.8, 10.0 Hz, 1H), 3.23 – 3.15 (m, 1H), 3.12 – 3.00 (m, 1H), 2.97 – 2.88 (m, 2H), 2.86 – 2.75 (m, 2H), 2.34 – 2.22 (m, 1H), 2.07 (s, 2H), 1.86 – 1.71 (m, 2H), 1.66 – 1.50 (m, 3H), 1.48 – 1.35 (m, 3H), 0.95 (t, *J* = 7.4 Hz, 3H), 0.83 – 0.72 (m, 1H). |
| **$^{13}C\{^1H\}$ NMR** | (101 MHz, $CDCl_3$) δ 160.5 (s), 157.2 (s), 147.2 (s), 144.6 (s), 142.0 (s), 131.3 (s), 127.2 (s), 124.9 (s), 120.8 (s), 120.4 (s), 114.4 (s), 102.4 (s), 62.0 (s), 56.5 (s), 55.7 (s), 41.1 (s), 40.1 (s), 39.0 (s), 32.4 (s), 28.4 (s), 27.7 (s), 26.1 (s), 22.8 (s), 14.2 (s). |
| **HRMS** | (ESI-TOF) m/z: $[M+H]^+$ calcd for C24H34N3O 380.2697; found 380.2717. |
| **$[\alpha]_D^{25}$** | +69.998 (c = 0.202 g/100mL, $CHCl_3$) |
| **IR** | 3377, 3297,3074,2930,2862, 1620, 1599, 1503, 1478, 1228 $cm^{-1}$ |

(1*R*)-(6-methoxy-2-phenylquinolin-4-yl)((2*R*,4*S*,5*R*)-5-vinylquinuclidin-2-yl)methanamine (**PhQd**)

Prepared according to the general procedure (GP3) from **6** (2.00 g, 5.00 mmol), affording **PhQd** as a yellow solid (1.51 g, 76% yield).

| | |
|---|---|
| **$^1$H NMR** | (400 MHz, $CDCl_3$) δ 8.19 – 8.06 (m, 4H), 7.56 – 7.47 (m, 3H), 7.47 – 7.36 (m, 2H), 5.91 (ddd, *J* = 17.1, 10.6, 6.5 Hz, 1H), 5.13 – 5.04 (m, 2H), 4.82 – 4.70 (m, 1H), 3.98 (s, 3H), 3.15 – 2.91 (m, 5H), 2.35 – 2.25 (m, 1H), 2.25 – 2.06 (m, 2H), 1.75 – 1.50 (m, 4H), 1.05 – 0.94 (m, 1H). |
| **$^{13}C\{^1H\}$ NMR** | (101 MHz, $CDCl_3$) δ 157.7 (s), 155.0 (s), 148.3 (s), 145.0 (s), 140.8 (s), 134.0 (s), 132.3 (s), 129.1 (s), 128.9 (s), 127.8 (s), 127.4 (s), 121.9 (s), 117.9 (s), 114.7 (s), 102.1 (s), 62.8 (s), 55.6 (s), 49.7 (s), 47.6 (s), 39.6 (s), 27.7 (s), 26.8 (s), 25.1 (s). |
| **HRMS** | (ESI-TOF) m/z: $[M+H]^+$ calcd for C26H30N3O 400.2384; found 400.2406. |
| **$[\alpha]_D^{25}$** | +78.708 (c = 0.178 g/100mL, $CHCl_3$) |
| **IR** | 3377, 3293, 3068, 2939, 2871, 2099, 1621, 1497, 1353, 1223 $cm^{-1}$ |

Analytical data was in accordance with literature.[13]

# 3. Substrate Synthesis

## 3.1. Synthesis of 4-piperidone-derived ketones

*N,N*-diethyl-4-oxopiperidine-1-carboxamide (**7**)

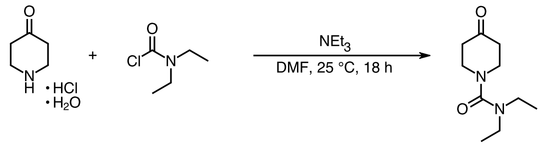

7

4-Piperidone monohydrate hydrochloride (3.07 g, 20.0 mmol, 1.0 eq.) was suspended in anhydrous DMF (40 mL). After the addition of $NEt_3$ (6.07 g, 60.0 mmol, 3.0 eq.), dimethylcarbamoyl chloride (4.07 g, 30.0 mmol, 1.5 eq.) was slowly added during vigorous stirring. The reaction mixture was stirred at 25 °C for 18 h. The reaction mixture was evaporated, and the residue was dissolved in EtOAc and distilled $H_2O$. After the phase separation, the aqueous phase was extracted with EtOAc three times, and the combined organic phases were washed with brine, dried over $MgSO_4$, filtrated, and evaporated. The crude product was purified *via* flash chromatography (silica, MeOH:DCM = 1:100, Anisaldehyde staining agent). **7** was obtained as a yellow liquid (2.45 g, 62% yield).

**$^1$H NMR** (400 MHz, $CDCl_3$) δ 3.49 (t, *J* = 6.1 Hz, 4H), 3.26 (q, *J* = 7.1 Hz, 4H), 2.48 (t, *J* = 6.2 Hz, 4H), 1.15 (t, *J* = 7.1 Hz, 6H).

**$^{13}$C{$^1$H} NMR** (101 MHz, $CDCl_3$) δ 208.3 (s), 164.1 (s), 46.8 (s), 42.1 (s), 41.5 (s), 13.4 (s).

Analytical data was in accordance with literature.[14]

Benzyl 4-oxopiperidine-1-carboxylate (**8**)

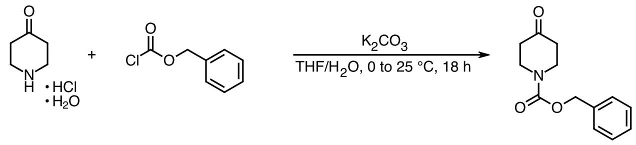

8

4-Piperidone monohydrate hydrochloride (3.14 g, 20.0 mmol, 1.0 eq.) was dissolved in distilled $H_2O$ (25 mL) and THF (25 mL). After adding $K_2CO_3$ (5.53 g, 40.0 mmol, 2.0 eq.), the biphasic system was cooled to 0 °C. Benzyl chloroformate (3.75 g, 22.0 mmol, 1.1 eq.) was added during vigorous stirring. The reaction mixture was stirred at 25 °C for 18 h. After the phase separation, the aqueous phase was extracted with EtOAc three times, and the combined organic phases were dried over anhydrous $MgSO_4$, filtrated, and evaporated. **8** was obtained as a yellow liquid and used without further purification (4.60 g, 99% yield).

**$^1$H NMR** (400 MHz, $CDCl_3$) δ 7.40 – 7.33 (m, 5H), 5.18 (s, 2H), 3.80 (t, $J$ = 6.3 Hz, 4H), 2.53 – 2.36 (m, 4H).

**$^{13}$C{$^1$H} NMR** (101 MHz, $CDCl_3$) δ 207.3 (s), 155.3 (s), 136.5 (s), 128.7 (s), 128.4 (s), 128.2 (s), 67.8 (s), 43.3 (s), 41.2 (s).

Analytical data was in accordance with literature.[15]

*N-tert*-butyloxycarbonylpiperidin-4-one (**9**)

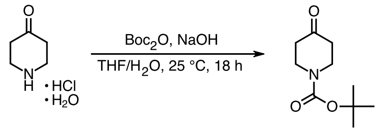


**9**

4-Piperidone monohydrate hydrochloride (3.07 g, 20.0 mmol, 1.0 eq.) was dissolved in distilled $H_2O$ (25 mL) and THF (25 mL). NaOH (0.80 g, 20.0 mmol, 1.0 eq.) was added to the biphasic system, followed by a solution of $Boc_2O$ (5.24 g, 24.0 mmol, 1.2 eq.) in THF (20 mL). The reaction mixture was stirred at 25 °C for 18 h. After the phase separation, the aqueous phase was extracted with EtOAc three times, and the combined organic phases were dried over anhydrous $MgSO_4$, filtrated, and evaporated. The solid crude product was recrystallized from *n*-heptane (20 mL). **9** was a white solid (1.86 g, 47% yield).

**$^1$H NMR** (400 MHz, $CDCl_3$) δ 3.71 (t, $J$ = 6.2 Hz, 4H), 2.43 (t, $J$ = 6.2 Hz, 4H), 1.49 (s, 9H).

**$^{13}$C{$^1$H} NMR** (101 MHz, $CDCl_3$) δ 208.0 (s), 154.6 (s), 80.6 (s), 43.2 (s), 41.3 (s), 28.5 (s).

Analytical data was in accordance with literature.[16]

## 3.2. Synthesis of dialkyl 2-bromomalonates

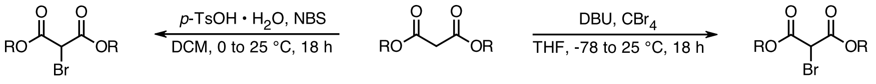


**10** R = Me
**11** R = *n*-Bu

**12** R = *t*-Bu
**13** R = *i*-Pr
**14** R = Bn

General Procedure for the bromination of malonates with NBS (GP4)

According to a modified literature procedure,[17] dialkyl 2-bromomalonates (**10** and **11**) were prepared on a 5.0 to 20.0 mmol scale. Malonic acid dialkyl ester (1.0 eq.) was dissolved in DCM (0.5 M), followed by the addition of *p*-TsOH · $H_2O$ (0.2 eq.). At 0 °C, NBS (1.0 eq.) was added, and the reaction mixture was stirred at 25 °C for 18 h. After adding distilled $H_2O$, the phases were separated, and the aqueous phase was extracted three times with DCM. The combined organic phases were dried over $MgSO_4$, filtrated, and evaporated, affording the crude product of the corresponding reaction.

<u>Dimethyl 2-bromomalonate (**10**)</u>

Prepared according to the general procedure (GP4) from malonic acid dimethyl ester (2.64 g, 20.0 mmol), affording **10** as a colorless liquid (2.46 g, 58% yield) after purification *via* distillation (115 °C at 25 mbar).

**$^1$H NMR** (400 MHz, $CDCl_3$) δ 4.86 (s, 1H), 3.84 (s, 6H).

**$^{13}$C{$^1$H} NMR** (101 MHz, $CDCl_3$) δ 165.2 (s), 54.1 (s), 41.7 (s).

Analytical data was in accordance with literature.[18]

<u>Dibutyl 2-bromomalonate (**11**)</u>

Prepared according to the general procedure (GP4) from malonic acid dibutyl ester (1.08 g, 5.00 mmol), affording **11** as a colorless liquid (0.69 g, 47% yield) after purification *via* flash chromatography (silica, EE:PE = 1:100, Anisaldehyde staining agent).

**$^1$H NMR** (400 MHz, $CDCl_3$) δ 4.83 (s, 1H), 4.23 (t, *J* = 6.6 Hz, 4H), 1.71 – 1.60 (m, 4H), 1.45 – 1.33 (m, 4H), 0.94 (t, *J* = 7.4 Hz, 6H).

**$^{13}$C{$^1$H} NMR** (101 MHz, $CDCl_3$) δ 164.8 (s), 67.2 (s), 42.6 (s), 30.5 (s), 19.1 (s), 13.7 (s).

Analytical data was in accordance with literature.[17]

General Procedure for the bromination of malonates with carbon tetrabromide (GP5)

According to a modified literature procedure,[19] dialkyl 2-bromomalonates (**12**, **13**, and **14**) were prepared on a 5.0 to 10.0 mmol scale. Malonic acid dialkyl ester (1.0 eq.) was dissolved in anhydrous THF (0.2 M) and DBU (1.0 eq.) was added at 0 °C. The solution was cooled to -78 °C, and $CBr_4$ (1.0 eq.) was added. The reaction mixture was stirred at 25 °C for 18 h. Saturated aq. $NH_4Cl$ was added, and after the phase separation, the aqueous phase was extracted three times with DCM. The combined organic phases were dried over anhydrous $MgSO_4$, filtrated, and evaporated. The crude product was purified *via* flash chromatography (silica, EtOAc:PE = 1:50, Vanillin staining agent).

Di-*tert*-butyl 2-bromomalonate (**12**)

Prepared according to the general procedure (GP5) from malonic acid di-*tert*-butyl ester (1.08 g, 5.00 mmol), affording **12** as a colorless liquid (0.62 g, 42% yield).

**$^1$H NMR** (400 MHz, $CDCl_3$) δ 4.65 (s, 1H), 1.49 (s, 18H).

**$^{13}$C{$^1$H} NMR** (101 MHz, $CDCl_3$) δ 163.8 (s), 84.0 (s), 45.8 (s), 27.8 (s).

Analytical data was in accordance with literature.[20]

Di-*iso*-propyl 2-bromomalonate (**13**)

Prepared according to the general procedure (GP5) from malonic acid di-*iso*-propyl ester (0.94 g, 5.00 mmol), affording **13** as a colorless liquid (0.40 g, 30% yield).

**$^1$H NMR** (400 MHz, $CDCl_3$) δ 5.10 (hept, *J* = 6.3 Hz, 2H), 4.77 (s, 1H), 1.29 (d, *J* = 6.3 Hz, 12H).

**$^{13}$C{$^1$H} NMR** (101 MHz, $CDCl_3$) δ 164.3 (s), 71.3 (s), 43.5 (s), 21.6 (s), 21.5 (s).

**HRMS** (ESI-TOF) m/z: $[M+H]^+$ calcd for C9H16BrO4 267.0227; found 267.0231.

**IR** 2984, 2941, 1736, 1467, 1376, 1288, 1248, 1147, 1097, 968 $cm^{-1}$

Dibenzyl 2-bromomalonate (**14**)

Prepared according to the general procedure (GP5) from malonic acid dibenzyl ester (2.84 g, 10.0 mmol), affording **14** as a colorless liquid (0.98 g, 27% yield).

**$^1$H NMR** (400 MHz, $CDCl_3$) δ 7.37 – 7.29 (m, 10H), 5.22 (s, 4H), 4.92 (s, 1H).

**$^{13}$C{$^1$H} NMR** (101 MHz, $CDCl_3$) δ 164.4 (s), 134.6 (s), 128.8 (s), 128.8 (s), 128.5 (s), 68.88 (s), 42.4(s).

Analytical data was in accordance with literature.[21]

# 4. Enantioselective α-alkylation of unfunctionalized ketones *via* direct photoexcitation

## 4.1. Batch reactor set-up and general considerations

All batch photoreactions were performed in a custom-made photoreactor composed of a cylindric case lined with LED strips (36 W, 365 nm) (Figure S1). An optical power of 3.0 ± 0.2 mW was recorded using a Thorlabs® PM100D device. The photoreactor was placed on a magnetic stirrer to enable stirring during the reaction. A beside-positioned fan ensured temperature control to maintain 25 °C during the reaction. A matching perforated rack was placed on top of the photoreactor to ensure uniform irradiation of the Schlenk tubes (four simultaneously) at a distance of 1 cm from the light source.

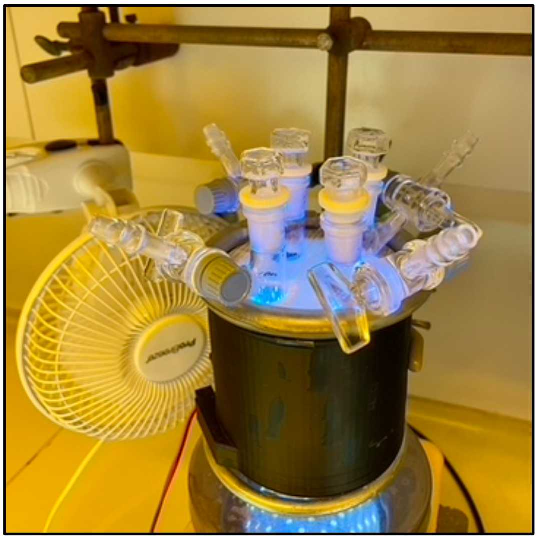

**Figure S1:** Batch Photoreactor. The picture was captured from behind the fume hood window, which was covered with an orange UV-protective foil (cut-off at 525 nm).

All batch photoreactions were performed in 8 mL Schlenk tubes and were prepared using standard Schlenk technique. The catalyst (0.04 mmol, 0.2 eq.) was dissolved in anhydrous solvent (0.2 M regarding the limiting component, 1.0 mL). To this solution, the corresponding acid (0.08 mmol, 0.4 eq.), ketone (0.40 mmol, 2.0 eq.), and dialkyl 2-bromomalonate (0.20 mmol, 1.0 eq.) were added. If the base (0.40 mmol, 2.0 eq.) was soluble, it was added to the reaction mixture. Otherwise, it was placed into the Schlenk tube. After evacuating and refilling the Schlenk tube with Ar three times, the reaction mixture was added under Ar counterflow. The Schlenk tube was sealed and placed into the custom-made photoreactor, and the reaction mixture was stirred and irradiated at 25 °C for 18 h. After completion, an aliquot was taken for GCMS and chiral HPLC measurement.

## 4.2. Synthesis and analytical data of α-alkylated ketones

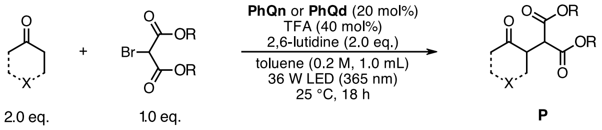

General Procedure for enantioselective α-alkylation of unfunctionalized ketones (GP6)

**PhQn** or **PhQd** (16.0 mg, 0.04 mmol, 0.2 eq.) was dissolved in anhydrous toluene (0.2 M considering the limiting component, 1.0 mL). To this solution, TFA (9.2 mg, 0.08 mmol, 0.4 eq.), ketone (0.40 mmol, 2.0 eq.), dialkyl 2-bromomalonate (0.20 mmol, 1.0 eq.), and 2,6-lutidine (42.9 mg, 0.40 mmol, 2.0 eq.) were added. After evacuating and refilling the Schlenk tube with Ar three times, the reaction mixture was added under Ar counterflow. The reaction mixture was stirred under Ar at 25 °C for 18 h during irradiation in the custom-made photoreactor. After completion, two identical parallel runs were merged. An aliquot was taken for chiral HPLC measurement. To afford isolated **P**, the remaining reaction mixture was evaporated, and the crude product was purified *via* flash chromatography (silica, EtOAc:PE = 1:5-50, Anisaldehyde staining agent).

The absolute configurations of **P2**-**P18** were assigned analogously to **P1** and based on the comparison of the optical rotation with literature.[22]

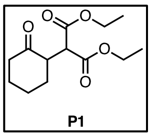

P1

Diethyl 2-(2-oxocyclohexyl)malonate (**P1**)

Prepared according to the general procedure (GP6) from cyclohexanone (39.3 mg, 0.40 mmol) and diethyl 2-bromomalonate (47.8 mg, 0.20 mmol), affording **P1** as a yellow liquid.

**Yield** 40.1 mg, (78%), using **PhQn**

17.5 mg (34%), using **PhQd**

**ee** 86% (*R*), using **PhQn**

79% (*S*), using **PhQd**

**$^1$H NMR** (400 MHz, $CDCl_3$) δ 4.24 – 4.14 (m, 4H), 3.65 (d, *J* = 9.5 Hz, 1H), 3.24 – 3.13 (m, 1H), 2.47 – 2.38 (m, 2H), 2.16 – 2.01 (m, 2H), 1.95 – 1.87 (m, 1H), 1.72 – 1.52 (m, 3H), 1.27 (t, *J* = 7.1 Hz, 3H), 1.26 (t, *J* = 7.1 Hz, 3H).

**$^{13}$C{$^1$H} NMR** (101 MHz, $CDCl_3$) δ 209.9 (s), 168.7 (s), 168.5 (s), 61.6 (s), 52.4 (s), 50.5 (s), 42.1 (s), 31.3 (s), 27.9 (s), 25.2 (s), 14.2 (s), 14.2 (s).

**$[\alpha]_D^{25}$** +53.973 (c = 0.219 g/100mL, $CHCl_3$), using **PhQn**

–32.565 (c = 0.269 g/100mL, $CHCl_3$), using **PhQd**

**HRMS** (ESI-TOF) m/z: $[M+H]^+$ calcd for C13H21O5 257.1384; found 257.1405.

**IR** 2982, 2940, 2865, 1730, 1712, 1448, 1368, 1274, 1177, 1026 $cm^{-1}$

Analytical data was in accordance with literature. [22]

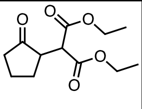

P2

Diethyl 2-(2-oxocyclopentyl)malonate (**P2**)

Prepared according to the general procedure (GP6) from cyclopentanone (33.6 mg, 0.40 mmol) and diethyl 2-bromomalonate (47.8 mg, 0.20 mmol), affording **P2** as a yellow liquid.

**Yield** 32.5 mg (67%), using **PhQn**

5.5 mg (11%), using **PhQd**

**ee** 55% (*R*), using **PhQn**

24% (*S*), using **PhQd**

**$^1$H NMR** (400 MHz, $CDCl_3$) δ 4.26 – 4.14 (m, 4H), 3.80 (d, *J* = 5.6 Hz, 1H), 2.75 – 2.64 (m, 1H), 2.35 – 2.16 (m, 3H), 2.14 – 2.04 (m, 1H), 1.98 – 1.84 (m, 1H), 1.84 – 1.74 (m, 1H), 1.28 (t, *J* = 7.1 Hz, 3H), 1.25 (t, *J* = 7.1 Hz, 3H).

**$^{13}$C{$^1$H} NMR** (101 MHz, $CDCl_3$) δ 217.3 (s), 168.7 (s), 168.1 (s), 61.7 (s), 51.4 (s), 48.6 (s), 37.5 (s), 26.6 (s), 20.7 (s), 14.2 (s), 14.1 (s).

**$[\alpha]_D^{25}$** –30.684 (c = 0.468 g/100mL, $CHCl_3$), using **PhQn**

+19.610 (c = 0.231 g/100mL, $CHCl_3$), using **PhQd**

**HRMS** (ESI-TOF) m/z: $[M+H]^+$ calcd for C12H19O5 243.1227; found 243.1230.

**IR** 2962, 1740, 1370, 1258, 1084, 1012, 864, 792, 662, 631 $cm^{-1}$

Analytical data was in accordance with literature.[23]

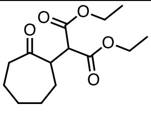

P3

Diethyl 2-(2-oxocycloheptyl)malonate (**P3**)

Prepared according to the general procedure (GP6) from cycloheptanone (44.9 mg, 0.40 mmol) and diethyl 2-bromomalonate (47.8 mg, 0.20 mmol), affording **P3** as a yellow liquid.

**Yield** 11.6 mg (21%), using **PhQn**

5.6 mg (10%), using **PhQd**

**ee** 52% (*R*), using **PhQn**

57% (*S*), using **PhQd**

**$^1$H NMR** (400 MHz, $CDCl_3$) δ 4.25 – 4.10 (m, 4H), 3.79 (d, *J* = 10.1 Hz, 1H), 3.46 – 3.36 (m, 1H), 2.74 – 2.64 (m, 1H), 2.51 – 2.38 (m, 1H), 1.99 – 1.65 (m, 6H), 1.58 – 1.48 (m, 1H), 1.30 – 1.21 (m, 7H).

**$^{13}C\{^1H\}$ NMR** (101 MHz, $CDCl_3$) δ 213.1 (s), 168.9 (s), 168.7 (s), 61.7 (s), 61.7 (s), 54.3 (s), 50.5 (s), 43.6 (s), 29.6 (s), 29.0 (s), 28.5 (s), 23.1 (s), 14.3 (s), 14.1 (s).

**$[\alpha]_D^{25}$** +30.052 (c = 0.767 g/100mL, $CHCl_3$), using **PhQn**

–20.890 (c = 0.292 g/100mL, $CHCl_3$), using **PhQd**

**HRMS** (ESI-TOF) m/z: $[M+H]^+$ calcd for C14H23O5 271.1540; found 271.1545.

**IR** 2933, 2859, 1729, 1705, 1447, 1368, 1229, 1137, 1029, 933 $cm^{-1}$

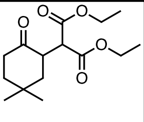

P4

Diethyl 2-(5,5-dimethyl-2-oxocyclohexyl)malonate (**P4**)

Prepared according to the general procedure (GP6) from 4,4-dimethylcyclohexanone (50.5 mg, 0.40 mmol) and diethyl 2-bromomalonate (47.8 mg, 0.20 mmol), affording **P4** as a yellow liquid.

**Yield** 47.3 g (83%), using **PhQn**

32.5 g (57%), using **PhQd**

**ee** 63% (*R*), using **PhQn**

65% (*S*), using **PhQd**

**$^1H$ NMR** (400 MHz, $CDCl_3$) δ 4.27 – 4.12 (m, 4H), 3.60 (d, *J* = 9.3 Hz, 1H), 3.37 – 3.27 (m, 1H), 2.62 – 2.50 (m, 1H), 2.34 – 2.24 (m, 1H), 1.79 – 1.70 (m, 1H), 1.70 – 1.61 (m, 2H), 1.61 – 1.51 (m, 1H), 1.27 (t, *J* = 7.1 Hz, 3H), 1.26 (t, *J* =7.1 Hz, 3H), 1.24 (s, 3H), 1.01 (s, 3H).

**$^{13}C\{^1H\}$ NMR** (101 MHz, $CDCl_3$) δ 210.5 (s), 168.5 (s), 61.7 (s), 61.6 (s), 52.4 (s), 46.4 (s), 43.6 (s), 40.0 (s), 38.1 (s), 31.5 (s), 31.0 (s), 24.4 (s), 14.3 (s), 14.2 (s).

**$[\alpha]_D^{25}$** +47.254 (c = 0.386 g/100mL, $CHCl_3$), using **PhQn**

–34.583 (c = 0.336 g/100mL, $CHCl_3$), using **PhQd**

**HRMS** (ESI-TOF) m/z: $[M+H]^+$ calcd for C15H25O 285.1697; found 285.1701.

**IR** 2956, 2870, 1751, 1732, 1715, 1466, 1369, 1252, 1179, 1030 $cm^{-1}$

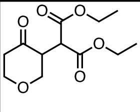

P5

Diethyl 2-(4-oxotetrahydro-2*H*-pyran-3-yl)malonate (**P5**)

Prepared according to the general procedure (GP6) from tetrahydro-4*H*-pyran-4-one (40.0 mg, 0.40 mmol) and diethyl 2-bromomalonate (47.8 mg, 0.20 mmol), affording **P5** as a yellow liquid.

**Yield** 32.8 mg (63%), using **PhQn**

15.2 mg (29%), using **PhQd**

**ee** 85% (*R*), using **PhQn**

83% (*S*), using **PhQd**

| | |
|---|---|
| **$^{1}$H NMR** | (400 MHz, $CDCl_3$) δ 4.30 – 4.14 (m, 6H), 3.76 – 3.63 (m, 3H), 3.40 – 3.29 (m, 1H), 2.78 – 2.65 (m, 1H), 2.45 – 2.38 (m, 1H), 1.26 (t, *J* = 7.1 Hz, 6H). |
| **$^{13}$C{$^{1}$H} NMR** | (101 MHz, $CDCl_3$) δ 205.1 (s), 167.9 (s), 167.8 (s), 70.2 (s), 68.5 (s), 61.9 (s), 61.9 (s), 50.5 (s), 49.1 (s), 42.6 (s), 14.1 (s), 14.1 (s). |
| **$[\alpha]_D^{25}$** | +3.773 (c = 0.501 g/100mL, $CHCl_3$), using **PhQn**<br>–1.787 (c = 0.235 g/100mL, $CHCl_3$), using **PhQd** |
| **HRMS** | (ESI-TOF) m/z: $[M+H]^+$ calcd for C12H19O6 259.1176; found 259.1181. |
| **IR** | 2982, 1718, 1466, 1369, 1255, 1218, 1177, 1150, 1096, 1025 $cm^{-1}$ |

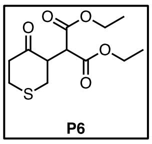
P6

Diethyl 2-(4-oxotetrahydro-2*H*-thiopyran-3-yl)malonate (**P6**)

Prepared according to the general procedure (GP6) from tetrahydro-4*H*-thiopyran-4-one (46.5 mg, 0.40 mmol) and diethyl 2-bromomalonate (47.8 mg, 0.20 mmol), affording **P6** as a yellow liquid.

| | |
|---|---|
| **Yield** | 33.1 mg (60%), using **PhQn**<br>13.4 mg (24%), using **PhQd** |
| **ee** | 87% (*R*), using **PhQn**<br>87% (*S*), using **PhQd** |
| **$^{1}$H NMR** | (400 MHz, $CDCl_3$) δ 4.23 – 4.16 (m, 4H), 3.79 (d, *J* = 8.9 Hz, 1H), 3.53 – 3.47 (m, 1H), 3.05 – 2.85 (m, 4H), 2.85 – 2.74 (m, 2H), 1.29 – 1.24 (m, 6H). |
| **$^{13}$C{$^{1}$H} NMR** | (101 MHz, $CDCl_3$) δ 207.3 (s), 168.1 (s), 168.0 (s), 61.9 (s), 52.6 (s), 52.1 (s), 44.6 (s), 33.3 (s), 30.9 (s), 14.2 (s), 14.1 (s). |
| **$[\alpha]_D^{25}$** | +18.949 (c = 0.276 g/100mL, $CHCl_3$), using **PhQn**<br>–8.350 (c = 0.206 g/100mL, $CHCl_3$), using **PhQd** |
| **HRMS** | (ESI-TOF) m/z: $[M+H]^+$ calcd for C12H19O5S 275.0948; found 275.0948. |
| **IR** | 2970, 1728, 1712, 1425, 1369, 1211, 1151, 1092, 1027, 830 $cm^{-1}$ |

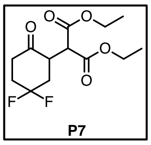
P7

Diethyl 2-(5,5-difluoro-2-oxocyclohexyl)malonate (**P7**)

Prepared according to the general procedure (GP6) from 4,4-difluorocyclohexanone (53.6 mg, 0.40 mmol) and diethyl 2-bromomalonate (47.8 mg, 0.20 mmol), affording **P7** as a yellow liquid.

| | |
|---|---|
| **Yield** | 41.1 mg (70%), using **PhQn**<br>21.2 mg (36%), using **PhQd** |

| | |
|---|---|
| **ee** | 68% (*R*), using **PhQn** |
| | 76% (*S*), using **PhQd** |
| **$^1$H NMR** | (400 MHz, $CDCl_3$) δ 4.28 – 4.15 (m, 4H), 3.78 (d, *J* = 7.5 Hz, 1H), 3.44 – 3.32 (m, 1H), 2.78 – 2.65 (m, 1H), 2.55 – 2.17 (m, 5H), 1.28 (t, *J* = 7.1 Hz, 3H), 1.28 (t, *J* = 7.1 Hz, 3H). |
| **$^{13}$C{$^1$H} NMR** | (101 MHz, $CDCl_3$) δ 205.5 (s), 168.0 (s), 167.9 (s), 121.4 (dd, *J* = 244.2, 239.7 Hz), 62.0 (s), 62.0 (s), 51.0 (s), 45.1 (d, *J* = 10.6 Hz), 36.3 (d, *J* = 9.8 Hz), 36.0 (t, *J* = 26.4 Hz), 33.0 (t, *J* = 26.0 Hz), 14.2 (s), 14.2 (s). |
| **$^{19}$F{$^1$H} NMR** | NMR (376 MHz, $CDCl_3$) δ -95.0 (d, *J* = 244.4 Hz), -102.0 (d, *J* = 243.8 Hz). |
| **HRMS** | (ESI-TOF) m/z: $[M+H]^+$ calcd for C13H19F2O5 293.1195; found 293.1198. |
| **IR** | 2984, 1722, 1441, 1370, 1281, 1258, 1227, 1136, 1028, 963 $cm^{-1}$ |

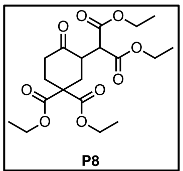

P8

Diethyl 2-(5,5-bis-ethoxycarbonyl-2-oxocyclohexyl)malonate (**P8**)

Prepared according to the general procedure (GP6) from diethyl 4-oxocyclohexane-1,1-dicarboxylate (96.9 mg, 0.40 mmol) and diethyl 2-bromomalonate (47.8 mg, 0.20 mmol), affording **P8** as a yellow liquid.

| | |
|---|---|
| **Yield** | 53.9 mg (67%), using **PhQn** |
| | 50.0 mg (62%), using **PhQd** |
| **ee** | 65% (*R*), using **PhQn** |
| | 73% (*S*), using **PhQd** |
| **$^1$H NMR** | (400 MHz, $CDCl_3$) δ 4.37 – 4.27 (m, 2H), 4.25 – 4.14 (m, 6H), 3.69 (d, *J* = 8.3 Hz, 1H), 3.34 – 3.24 (m, 1H), 2.72 – 2.57 (m, 3H), 2.47 – 2.39 (m, 1H), 2.21 – 2.08 (m, 2H), 1.33 – 1.22 (m, 12H). |
| **$^{13}$C{$^1$H} NMR** | (101 MHz, $CDCl_3$) δ 207.7 (s), 170.6 (s), 170.1 (s), 168.2 (s), 168.0 (s), 62.1 (s), 61.8 (s), 61.8 (s), 54.3 (s), 51.9 (s), 46.2 (s), 37.9 (s), 34.5 (s), 31.5 (s), 14.2 (s), 14.2 (s), 14.1 (s). |
| **HRMS** | (ESI-TOF) m/z: $[M+H]^+$ calcd for C19H29O9 401.1806; found 401.1821. |
| **IR** | 2982, 1727, 1446, 1368, 1235, 1178, 1094, 1024, 862, 631 $cm^{-1}$ |

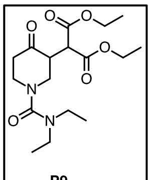

P9

Diethyl 2-[1-(diethylcarbamoyl)-4-oxopiperidin-3-yl]malonate (**P9**)

Prepared according to the general procedure (GP6) from **7** (79.3 mg, 0.40 mmol) and diethyl 2-bromomalonate (47.8 mg, 0.20 mmol), affording **P9** as a yellow liquid.

| | |
|---|---|
| **Yield** | 24.5 mg (34%), using **PhQn** |
| | 16.0 mg (22%), using **PhQd** |

| | |
|---|---|
| **ee** | 81% (*R*), using **PhQn** |
| | 77% (*S*), using **PhQd** |
| **$^1$H NMR** | (400 MHz, $CDCl_3$) δ 4.26 – 4.15 (m, 4H), 3.96 – 3.82 (m, 2H), 3.74 (d, *J* = 7.9 Hz, 1H), 3.37 – 3.20 (m, 5H), 3.20 – 3.04 (m, 2H), 2.77 – 2.64 (m, 1H), 2.47 – 2.39 (m, 1H), 1.27 (t, *J* = 7.1 Hz, 6H), 1.14 (t, *J* = 7.1 Hz, 6H). |
| **$^{13}$C{$^1$H} NMR** | (101 MHz, $CDCl_3$) δ 206.6 (s), 168.1 (s), 168.0 (s), 163.8 (s), 61.9 (s), 61.9 (s), 50.0 (s), 49.9 (s), 49.4 (s), 47.1 (s), 42.1 (s), 41.2 (s), 14.2 (s), 14.1 (s), 13.3 (s). |
| **HRMS** | (ESI-TOF) m/z: $[M+H]^+$ calcd for C17H29N2O6 357.2020; found 357.2027. |
| **IR** | 3458, 2971, 2935, 1717, 1639, 1420, 1369, 1262, 1169, 1097 $cm^{-1}$ |

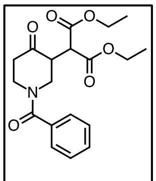

P10

Diethyl 2-(1-benzoyl-4-oxopiperidin-3-yl)malonate (**P10**)

Prepared according to the general procedure (GP6) from 1-benzoyl-4-piperidone (81.3 mg, 0.40 mmol) and diethyl 2-bromomalonate (47.8 mg, 0.20 mmol), affording **P10** as a yellow liquid.

| | |
|---|---|
| **Yield** | 55.1 mg (76%), using **PhQn** |
| | 40.4 mg (56%), using **PhQd** |
| **ee** | 75% (*R*), using **PhQn** |
| | 79% (*S*), using **PhQd** |
| **$^1$H NMR** | (400 MHz, $CDCl_3$) δ 7.51 – 7.39 (m, 5H), 5.11 – 4.61 (m, 1H), 4.39 – 3.89 (m, 5H), 3.76 (s, 1H), 3.64 – 3.02 (m, 3H), 2.84 – 2.33 (m, 2H), 1.29 – 1.10 (m, 6H). |
| **$^{13}$C{$^1$H} NMR** | (101 MHz, $CDCl_3$) δ 205.3 (s), 171.1 (s), 167.8 (s), 167.7 (s), 135.0 (s), 130.5 (s), 128.8 (s), 127.2 (s), 62.1 (s), 62.0 (s), 49.9 (s), 41.0 (s), 14.1 (s). |
| **HRMS** | (ESI-TOF) m/z: $[M+H]^+$ calcd for C19H24NO6 362.1598; found 362.1601. |
| **IR** | 2981, 1720, 1638, 1430, 1370, 1276, 1243, 1178, 1132, 1024 $cm^{-1}$ |

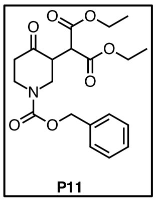

P11

Diethyl 2-(1-phenoxycarbonyl-4-oxopiperidin-3-yl)malonate (**P11**)

Prepared according to the general procedure (GP6) from **8** (93.3 mg, 0.40 mmol) and diethyl 2-bromomalonate (47.8 mg, 0.20 mmol), affording **P11** as a yellow liquid.

| | |
|---|---|
| **Yield** | 50.2 mg (64%), using **PhQn** |
| | 30.4 mg (39%), using **PhQd** |
| **ee** | 88% (*R*), using **PhQn** |
| | 86% (*S*), using **PhQd** |

| | |
|---|---|
| **$^{1}$H NMR** | (400 MHz, $CDCl_3$) δ 7.41 – 7.29 (m, 5H), 5.17 (s, 2H), 4.54 – 4.30 (m, 2H), 4.29 – 4.08 (m, 4H), 3.76 (d, *J* = 7.1 Hz, 1H), 3.35 – 3.11 (m, 3H), 2.66 – 2.41 (m, 2H), 1.31 – 1.17 (m, 6H). |
| **$^{13}$C{$^{1}$H} NMR** | (101 MHz, $CDCl_3$) δ 205.7 (s), 167.9 (s), 167.8 (s), 155.1 (s), 136.3 (s), 128.7 (s), 128.4 (s), 128.2 (s), 67.9 (s), 62.0 (s), 62.0 (s), 50.0 (s), 49.5 (s), 46.1 (s), 43.8 (s), 41.0 (s), 14.1 (s). |
| **HRMS** | (ESI-TOF) m/z: $[M+H]^+$ calcd for C20H26NO7 392.1704; found 392.1714. |
| **IR** | 2982, 1728, 1689, 1431, 1369, 1274, 1219, 1178, 1142, 1026 $cm^{-1}$ |

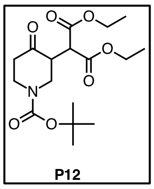
P12

Diethyl 2-(1-(*tert*-butoxycarbonyl)-4-oxopiperidin-3-yl)malonate (**P12**)

Prepared according to the general procedure (GP6) from **9** (79.7 mg, 0.40 mmol) and diethyl 2-bromomalonate (47.8 mg, 0.20 mmol), affording **P12** as a yellow liquid.

| | |
|---|---|
| **Yield** | 48.0 mg (67%), using **PhQn** |
| | 34.3 mg (48%), using **PhQd** |
| **ee** | 79% (*R*), using **PhQn** |
| | 83% (*S*), using **PhQd** |
| **$^{1}$H NMR** | (400 MHz, $CDCl_3$) δ 4.45 – 4.15 (m, 6H), 3.74 (d, *J* = 7.7 Hz, 1H), 3.24 – 3.08 (m, 3H), 2.62 – 2.51 (m, 1H), 2.51 – 2.41 (m, 1H), 1.49 (s, 9H), 1.29 (t, *J* = 7.1 Hz, 3H), 1.28 (t, *J* = 7.1 Hz, 3H). |
| **$^{13}$C{$^{1}$H} NMR** | (151 MHz, $CDCl_3$) δ 206.3 (s), 168.0 (s), 167.9 (s), 154.4 (s), 80.9 (s), 62.0 (s), 62.0 (s), 50.0 (s), 49.6 (s), 41.1 (s), 28.5 (s), 14.2 (s), 14.2 (s). |
| **HRMS** | (ESI-TOF) m/z: $[M+H]^+$ calcd for C17H28NO7 358.1860; found 358.1858. |
| **IR** | 2979, 1729, 1697, 1601, 1421, 1367, 1237, 1157, 1024, 863 $cm^{-1}$ |

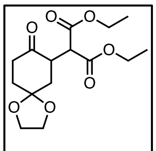
P13

Diethyl 2-(8-oxo-1,4-dioxaspiro[4.5]dec-7-yl)malonate (**P13**)

Prepared according to the general procedure (GP6), from cyclohexanedione monoethylene ketal (62.5 mg, 0.40 mmol) and diethyl 2-bromomalonate (47.8 mg, 0.20 mmol), affording **P13** as a yellow liquid.

| | |
|---|---|
| **Yield** | 38.6 mg (61%), using **PhQn** |
| | 26.4 mg (42%), using **PhQd** |
| **ee** | 85% (*R*), using **PhQn** |
| | 87% (*S*), using **PhQd** |

**$^{1}$H NMR** (400 MHz, $CDCl_3$) δ 4.23 – 4.13 (m, 4H), 4.06 – 3.95 (m, 4H), 3.68 (d, *J* = 8.5 Hz, 1H), 3.53 – 3.42 (m, 1H), 2.79 – 2.65 (m, 1H), 2.44 – 2.34 (m, 1H), 2.05 – 1.94 (m, 4H), 1.25 (t, *J* = 7.1 Hz, 3H), 1.24 (t, *J* = 7.1 Hz, 3H).

**$^{13}C\{^{1}H\}$ NMR** (101 MHz, $CDCl_3$) δ 208.3 (s), 168.2 (s), 107.1 (s), 64.9 (s), 64.7 (s), 61.7 (s), 61.6 (s), 51.6 (s), 46.4 (s), 37.9 (s), 37.5 (s), 34.5 (s), 14.2 (s), 14.1 (s).

**$[\alpha]_D^{25}$** +53.973 (c = 0.219 g/100mL, $CHCl_3$), using **PhQn**

–32.565 (c = 0.269 g/100mL, $CHCl_3$), using **PhQd**

**HRMS** (ESI-TOF) m/z: $[M+H]^+$ calcd for C15H23O7 315.1438; found 315.1445.

**IR** 2971, 1717, 1440, 1368, 1275, 1230, 1141, 1052, 1026, 949 $cm^{-1}$

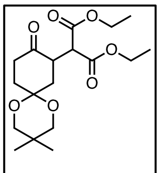

P14

Diethyl 2-(3,3-dimethyl-9-oxo-1,5-dioxaspiro[5.5]undec-8-yl)malonate (**P14**)

Prepared according to the general procedure (GP6) from 3,3-dimethyl-1,5-dioxaspiro[5.5]undecan-9-one (79.3 mg, 0.40 mmol) and diethyl 2-bromomalonate (47.8 mg, 0.20 mmol), affording **P14** as a yellow liquid.

**Yield** 58.9 mg (83%), using **PhQn**

29.2 mg (41%), using **PhQd**

**ee** 90% (*R*), using **PhQn**

41% (*S*), using **PhQd**

**$^{1}$H NMR** (400 MHz, $CDCl_3$) δ 4.26 – 4.15 (m, 4H), 3.70 (d, *J* = 8.2 Hz, 1H), 3.64 – 3.46 (m, 4H), 3.40 – 3.29 (m, 1H), 2.69 – 2.57 (m, 2H), 2.51 – 2.41 (m, 1H), 2.38 – 2.29 (m, 1H), 1.87 – 1.68 (m, 2H), 1.28 (t, *J* = 7.1 Hz, 3H), 1.27 (t, *J* = 7.1 Hz, 3H), 1.04 (s, 3H), 0.94 (s, 3H).

**$^{13}C\{^{1}H\}$ NMR** (101 MHz, $CDCl_3$) δ 208.7 (s), 168.4 (s), 168.4 (s), 96.3 (s), 71.0 (s), 70.5 (s), 61.7 (s), 61.7 (s), 51.5 (s), 45.3 (s), 36.8 (s), 33.7 (s), 33.0 (s), 30.3 (s), 22.9 (s), 22.7 (s), 14.3 (s), 14.2 (s).

**$[\alpha]_D^{25}$** +10.552 (c = 0.308 g/100mL, $CHCl_3$), using **PhQn**

–11.163 (c = 0.129 g/100mL, $CHCl_3$), using **PhQd**

**HRMS** (ESI-TOF) m/z: $[M+H]^+$ calcd for C18H29O7 357.1908; found 357.1915.

**IR** 2958, 2871, 1719, 1467, 1367, 1275, 1231, 1139, 1022, 919 $cm^{-1}$

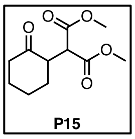
P15

Dimethyl 2-(2-oxocyclohexyl)malonate (**P15**)

Prepared according to the general procedure (GP6) from cyclohexanone (39.3 mg, 0.40 mmol) and **10** (42.2 mg, 0.20 mmol), affording **P15** as a yellow liquid.

**Yield** 32.1 mg (70%), using **PhQn**

12.3 mg (27%), using **PhQd**

**ee** 72% (*R*), using **PhQn**

72% (*S*), using **PhQd**

**$^1$H NMR** (400 MHz, $CDCl_3$) δ 3.73 (s, 3H), 3.73 (s, 3H), 3.68 (d, *J* = 9.5 Hz, 1H), 3.24 – 3.14 (m, 1H), 2.47 – 2.38 (m, 2H), 2.16 – 2.08 (m, 1H), 2.08 – 1.98 (m, 1H), 1.95 – 1.87 (m, 1H), 1.75 – 1.61 (m, 2H), 1.59 – 1.48 (m, 1H).

**$^{13}$C{$^1$H} NMR** (101 MHz, $CDCl_3$) δ 209.8 (s), 169.0 (s), 168.9 (s), 52.8 (s), 52.7 (s), 52.1 (s), 50.5 (s), 42.0 (s), 31.3 (s), 27.9 (s), 25.2 (s).

**$[\alpha]_D^{25}$** +33.753 (c = 0.445 g/100mL, $CHCl_3$), using **PhQn**

–31.398 (c = 0.465 g/100mL, $CHCl_3$), using **PhQd**

**HRMS** (ESI-TOF) m/z: $[M+H]^+$ calcd for C11H17O5 229.1071; found 229.1073.

**IR** 2956, 2864, 1736, 1711, 1435, 1346, 1258, 1231, 1013, 794 $cm^{-1}$

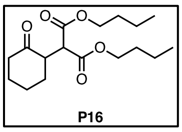
P16

Dibutyl 2-(2-oxocyclohexyl)malonate (**P16**)

Prepared according to the general procedure (GP6) from cyclohexanone (39.3 mg, 0.40 mmol) and **11** (59.0 mg, 0.20 mmol), affording **P16** as a yellow liquid.

**Yield** 28.7 mg (46%), using **PhQn**

13.9 mg (22%), using **PhQd**

**ee** 74% (*R*), using **PhQn**

71% (*S*), using **PhQd**

**$^1$H NMR** (400 MHz, $CDCl_3$) δ 4.19 – 4.07 (m, 4H), 3.66 (d, *J* = 9.5 Hz, 1H), 3.24 – 3.13 (m, 1H), 2.47 – 2.35 (m, 2H), 2.17 – 2.09 (m, 1H), 2.09 – 2.00 (m, 1H), 1.96 – 1.87 (m, 1H), 1.75 – 1.51 (m, 7H), 1.42 – 1.32 (m, 4H), 0.96 – 0.89 (m, 6H).

**$^{13}$C{$^1$H} NMR** (101 MHz, $CDCl_3$) δ 209.9 (s), 168.8 (s), 168.6 (s), 65.5 (s), 65.5 (s), 52.5 (s), 50.5 (s), 42.1 (s), 31.4 (s), 30.6 (s), 30.6 (s), 28.0 (s), 25.2 (s), 19.2 (s), 13.8 (s), 13.8 (s).

**$[\alpha]_D^{25}$** +40.706 (c = 0.439 g/100mL, $CHCl_3$), using **PhQn**

–33.195 (c = 0.482 g/100mL, $CHCl_3$), using **PhQd**

**HRMS** (ESI-TOF) m/z: $[M+H]^+$ calcd for C17H29O5 313.2010; found 313.2013.

**IR** 2959, 2936, 2873, 1751, 1731, 1713, 1450, 1276, 1218, 1134 $cm^{-1}$

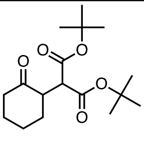

P17

Di-*tert*-butyl 2-(2-oxocyclohexyl)malonate (**P17**)

Prepared according to the general procedure (GP6) from cyclohexanone (39.3 mg, 0.40 mmol) and **12** (59.0 mg, 0.20 mg), affording **P17** as a yellow solid.

**Yield** 25.2 mg (40%), using **PhQn**

9.2 mg (15%), using **PhQd**

**ee** 77% (*R*), using **PhQn**

80% (*S*), using **PhQd**

**$^1$H NMR** (400 MHz, $CDCl_3$) δ 3.47 (d, *J* = 9.6 Hz, 1H), 3.14 – 3.01 (m, 1H), 2.46 – 2.37 (m, 2H), 2.16 – 2.02 (m, 2H), 1.94 – 1.86 (m, 1H), 1.78 – 1.60 (m, 2H), 1.55 – 1.49 (m, 1H), 1.46 (s, 9H), 1.45 (s, 9H).

**$^{13}$C{$^1$H} NMR** (101 MHz, $CDCl_3$) δ 210.1 (s), 168.1 (s), 167.8 (s), 81.8 (s), 81.5 (s), 54.3 (s), 50.3 (s), 42.3 (s), 31.3 (s), 28.1 (s), 28.1 (s), 28.0 (s), 25.3 (s).

**$[\alpha]_D^{25}$** +89.423 (c = 0.156 g/100mL, $CHCl_3$), using **PhQn**

–39.486 (c = 0.447 g/100mL, $CHCl_3$), using **PhQd**

**HRMS** (ESI-TOF) m/z: $[M+H]^+$ calcd for C17H29O5 313.2010; found 313.2012.

**IR** 2972, 2936, 2867, 1745, 1704, 1449, 1365, 1301, 1243, 1124 $cm^{-1}$

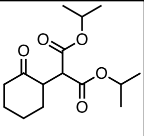

P18

Di-*iso*-propyl 2-(2-oxocyclohexyl)malonate (**P18**)

Prepared according to the general procedure (GP6) from cyclohexanone (39.3 mg, 0.40 mmol) and **13** (53.4 mg, 0.20 mmol), affording **P18** as a yellow liquid.

**Yield** 36.3 mg (64%), using **PhQn**

19.3 mg (34%), using **PhQd**

**Ee** 72% (*R*), using **PhQn**

71% (*S*), using **PhQd**

**$^1$H NMR** (400 MHz, $CDCl_3$) δ 5.10 – 4.97 (m, *J* = 6.2 Hz, 2H), 3.58 (d, *J* = 9.5 Hz, 1H), 3.21 – 3.11 (m, 1H), 2.46 – 2.35 (m, 2H), 2.16 – 2.01 (m, 2H), 1.95 – 1.85 (m, 1H), 1.78 – 1.59 (m, 2H), 1.57 – 1.49 (m, 1H), 1.28 – 1.20 (m, 12H).

**$^{13}$C{$^1$H} NMR** (101 MHz, $CDCl_3$) δ 209.9 (s), 168.2 (s), 168.1 (s), 69.1 (s), 69.0 (s), 52.8 (s), 50.3 (s), 42.1 (s), 31.3 (s), 28.0 (s), 25.3 (s), 21.8 (s), 21.8 (s), 21.7 (s).

**$[\alpha]_D^{25}$** +40.969 (c = 0.289 g/100mL, $CHCl_3$), using **PhQn**

–40.060 (c = 0.331 g/100mL, $CHCl_3$), using **PhQd**

**HRMS** (ESI-TOF) m/z: $[M+H]^+$ calcd for C15H25O5 285.1697; found 285.1700.

**IR** 2984, 2942, 2862, 1747, 1713, 1439, 1275, 1179, 1099, 1034 $cm^{-1}$

## 4.3. Large-scale experiment for the synthesis of **P1**

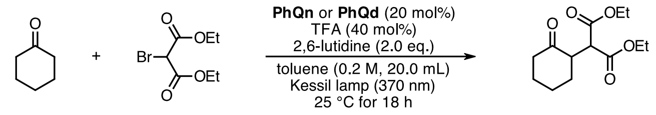


**PhQn** or **PhQd** (319.6 mg, 0.80 mmol, 0.2 eq.) was dissolved in anhydrous toluene (0.2 M considering the limiting component, 20.0 mL). To this solution, TFA (230.4 mg, 1.60 mmol, 0.4 eq.), cyclohexanone (785.2 mg, 8.00 mmol, 2.0 eq.), diethyl 2-bromomalonate (956.2 mg, 4.00 mmol, 1.0 eq.) and 2,6-lutidine (857.3 mg, 8.00 mmol, 2.0 eq.) were added. After evacuating and refilling a three-neck round bottom flask equipped with a stopcock with Ar three times, the reaction mixture was added under Ar counterflow. The reaction mixture was stirred under Ar at 25 °C (fan-cooling ensured temperature control) for 18 h during irradiation (two Kessil PR160L-370nm lamps, 10 cm irradiation distance, 100% output power). After completion, an aliquot was taken for chiral HPLC measurement. To afford isolated **P1**, the remaining reaction mixture was evaporated, and the crude product was purified *via* flash chromatography (silica, EtOAc:PE = 1:5, Anisaldehyde staining agent).

| | |
|---|---|
| **Yield** | 650.4 mg (63%), using **PhQn** |
| | 279.2 mg (27%), using **PhQd** |
| **ee** | 85% (*R*), using **PhQn** |
| | 78% (*S*), using **PhQd** |

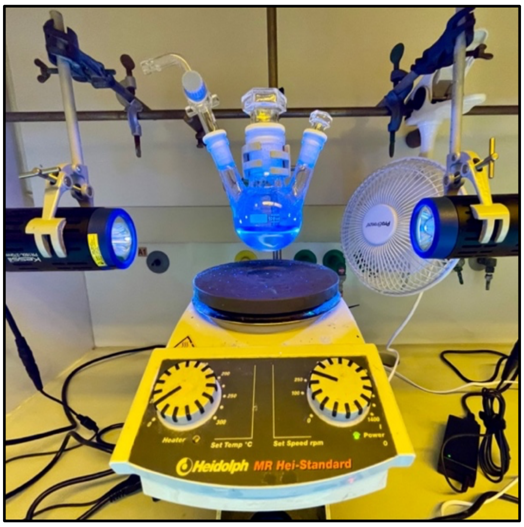


**Figure S2:** Large-scale experiment. The picture was captured from behind the fume hood window, which was covered with an orange UV-protective foil (cut-off at 525 nm).

# 5. Control experiment with TEMPO

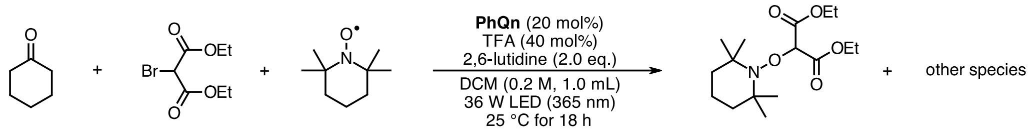


**PhQn** (16.0 mg, 0.04 mmol, 0.2 eq.) was dissolved in anhydrous DCM (0.2 M considering the limiting component, 1.0 mL). To this solution, TFA (9.2 mg, 0.08 mmol, 0.4 eq.), cyclohexanone (39.7 mg, 0.40 mmol, 2.0 eq.), diethyl 2-bromomalonate (48.8 mg, 0.20 mmol, 1.0 eq.) and 2,6-lutidine (42.9 mg, 0.40 mmol, 2.0 eq.) were added. After evacuating and refilling the Schlenk tube with Ar three times, TEMPO (63.8 mg, 0.40 mmol, 2.0 eq.) and the reaction mixture were added under Ar counterflow. The reaction mixture was stirred under Ar at 25 °C for 18 h during irradiation in the custom-made photoreactor. An aliquot was taken for GCMS measurement, confirming the absence of product formation. After evaporation of the reaction mixture an aliquot was taken for HRMS measurement to confirm the formation of the TEMPO adduct.

**HRMS** (ESI-TOF) m/z: $[M+H]^+$ calcd for C16H30NO5 316.2119; found 316.2124.

# 6. Subsequent reactions from **P1**

## 6.1. Ketone reduction

Diethyl 2-(*trans*-2-hydroxycyclohexyl)malonate (***trans*-R1**)

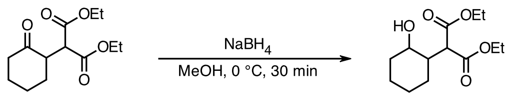


**P1** (128.1 mg, 0.50 mmol, 1.0 eq.) was dissolved in MeOH (2.0 mL) and cooled to 0 °C. $NaBH_4$ (28.4 mg, 0.75 mmol, 1.5 eq.) was added, and the reaction mixture was stirred at 0 °C for 30 min. Saturated aq. $NH_4Cl$ (5.0 mL) was added, the phases were separated, and the aqueous layer was extracted three times with EtOAc. The combined organic layers were dried over anhydrous $MgSO_4$, filtrated, and evaporated. The crude product was purified *via* flash chromatography (silica, EtOAc:PE = 1:5, Anisaldehyde staining agent). ***trans*-R1** was obtained as a yellow liquid (110.7 mg, 86% yield, d.r. = 87:13). Based on literature,[24] we assume the *trans*-product to be dominant. During the transformation, only a marginal racemization occurred, providing the product with an optical purity very close to that of **P1**.

| | |
|---|---|
| **ee** | 83% (*R*), starting from ***R*-P1** |
| | 76% (*S*), starting from ***S*-P1** |
| **$^1$H NMR** | (400 MHz, $CDCl_3$) δ 4.24 – 4.15 (m, 4H), 3.64 (d, *J* = 5.6 Hz, 1H), 3.51 – 3.40 (m, 1H), 2.12 – 1.97 (m, 2H), 1.81 – 1.66 (m, 4H), 1.30 – 1.24 (m, 9H). |
| **$^{13}$C{$^1$H} NMR** | (101 MHz, $CDCl_3$) δ 169.8 (s), 169.4 (s), 72.9 (s), 61.4 (s), 61.4 (s), 54.2 (s), 45.8 (s), 36.2 (s), 28.6 (s), 25.6 (s), 25.0 (s), 14.3 (s), 14.2 (s). |
| **$[\alpha]_D^{25}$** | +34.103 (c = 0.095 g/100mL, $CHCl_3$), starting from ***R*-P1** |
| | –22.727 (c = 0.231 g/100mL, $CHCl_3$), starting from ***S*-P1** |
| **HRMS** | (ESI-TOF) m/z: $[M+H]^+$ calcd for C13H23O5 259.1540; found 259.1542. |
| **IR** | 3445, 2930, 2857, 1726, 1489, 1369, 1240, 1175, 1030, 863 $cm^{-1}$ |

## 6.2. Fischer indole synthesis

Diethyl 2-(6-bromo-2,3,4,9-tetrahydro-1*H*-carbazol-1-yl)malonate (**R2**)

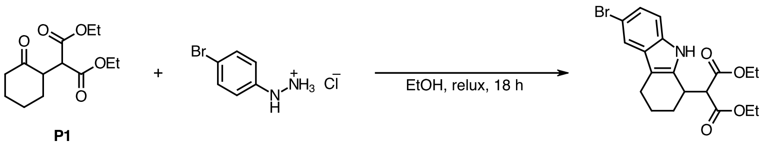


**P1** (256.3 mg, 1.00 mmol, 1.0 eq.) was dissolved in EtOH (4.0 mL), followed by the addition of 4-bromophenylhydrazine hydrochloride (245.9 mg, 1.10 mmol, 1.1 eq.). The reaction mixture was refluxed for 18 h and evaporated. The crude product was purified *via* flash chromatography (silica, EtOAc:PE = 1:20, Anisaldehyde staining agent). **R2** was obtained as a white solid (181.9 mg, 44% yield). For crystallization, **R2** was dissolved in MeOH and slowly evaporated.

**ee** 8% (*R*), starting from ***R*-P1**

7% (*S*), starting from ***S*-P1**

**$^1$H NMR** (400 MHz, $CDCl_3$) δ 8.74 (s, 1H), 7.61 – 7.55 (m, 1H), 7.22 – 7.12 (m, 2H), 4.34 – 4.24 (m, 2H), 4.22 – 4.03 (m, 2H), 3.71 – 3.61 (m, 2H), 2.71 – 2.59 (m, 2H), 2.01 – 1.79 (m, 4H), 1.32 (t, *J* = 7.1 Hz, 3H), 1.11 (t, *J* = 7.1 Hz, 3H).

**$^{13}$C{$^1$H} NMR** (101 MHz, $CDCl_3$) δ 169.8 (s), 169.2 (s), 135.0 (s), 134.3 (s), 129.0 (s), 124.4 (s), 120.9 (s), 112.3 (s), 112.3 (s), 111.4 (s), 62.1 (s), 62.0 (s), 57.2 (s), 33.2 (s), 28.0 (s), 21.0 (s), 20.8 (s), 14.3 (s), 14.0 (s).

**HRMS** (ESI-TOF) m/z: $[M+H]^+$ calcd for C19H23BrNO4 408.0805; found 408.0802.

**IR** 3380, 2969, 2929, 2844, 1744, 1704, 1465, 1441, 1318, 1195 $cm^{-1}$

## 6.3. *In-situ* derivatization

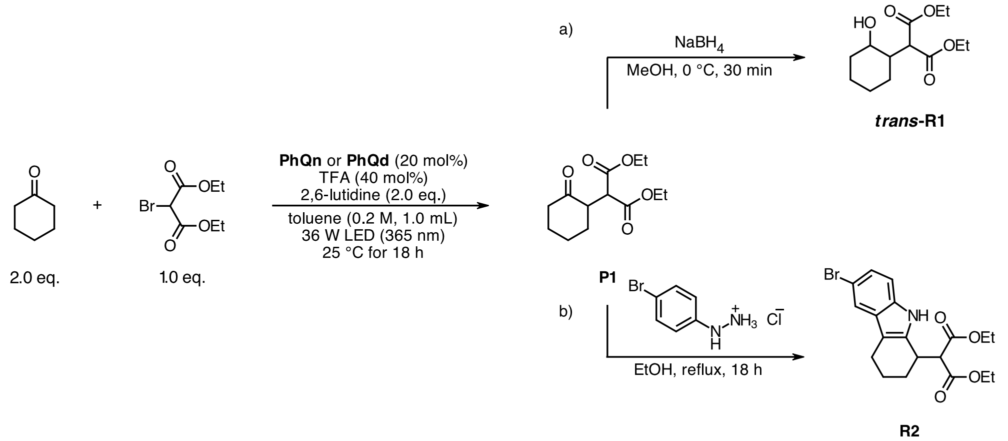


**P1** was prepared according to the general procedure (GP6) from cyclohexanone (39.3 mg, 0.40 mmol) and diethyl 2-bromomalonate (47.8 mg, 0.20 mmol). Two identical samples were merged and evaporated.

*Route a* (ketone reduction): MeOH (2.0 mL) was added, followed by the addition of $NaBH_4$ (90.8 mg, 2.40 mmol, 3.0 eq. referring to cyclohexanone) at 0 °C. After stirring the reaction mixture at 0 °C for 30 min, saturated aq. $NH_4Cl$ (5.0 mL) was added. After the phase separation, the aqueous layer was extracted three times with EtOAc. The combined organic layers were dried over anhydrous $MgSO_4$, filtrated, and evaporated. The crude product was purified *via* flash chromatography (silica, EtOAc:PE = 1:5, Anisaldehyde staining agent). ***trans*-R1** was obtained as a yellow liquid.

| | |
|---|---|
| **Yield** | 35.2 mg (34%), d.r. = 84:16, using **PhQn** |
| | 15.4 mg (15%), d.r. = 82:18, using **PhQd** |
| **ee** | 63% (*R*), using **PhQn** |
| | 57% (*S*), using **PhQd** |

*Route b* (Fischer indole synthesis): EtOH (2.0 mL) was added, followed by the addition of 4-bromophenylhydrazine hydrochloride (357.6 mg, 1.60 mmol, 2.0 eq. referring to cyclohexanone). The reaction mixture was refluxed for 18 h and evaporated. The crude product was purified *via* flash chromatography (silica, EtOAc:PE = 1:20, Anisaldehyde staining agent). **R2** was obtained as a white solid.

| | |
|---|---|
| **Yield** | 28.6 mg (18%), using **PhQn** |
| | 23.8 mg (15%), using **PhQd** |
| **ee** | racemic, using **PhQn** |
| | racemic, using **PhQd** |

# 7. Continuous flow implementation

## 7.1. Flow reactor set-up and general considerations

All flow photoreactions were performed using a Vapourtec® E-Series continuous flow machine. The pump system was either connected to a Vapourtec® UV-150 photochemical reactor module (Figure S3.a) or a custom-made photochemical reactor module (Figure S3.b). The former was constructed of UV transparent tubing (i.d. 1.0 mm, FEP, 10 mL) coiled around a cartridge. A Gen-2 LED assembly (62 W, 365 nm) was positioned within the cartridge. The temperature was controlled with a dry-ice-reliant integrated cooling system. The latter was constructed of UV transparent tubing (i.d. 0.8 mm, FEP, 25 mL) coiled around a condenser connected to a cryostat. This assembly was positioned within a cylindric case lined with Waveform Lighting® realUV LED strip lights (73.8 W, 365 nm). The flow reactor set-up is schematically illustrated in Figure S3.c, regardless of which of the two photochemical reactor modules was used. The reaction mixture was stored in a vial under an Ar atmosphere, and pure solvent was stored in a container. The split allowed for switching between the extraction of the reaction mixture and the pure solvent. The pump controlled the flow rate, and the back pressure regulator (BPR) regulated the pressure. After passing through the photochemical reactor, the reaction mixture was collected in a separate container.

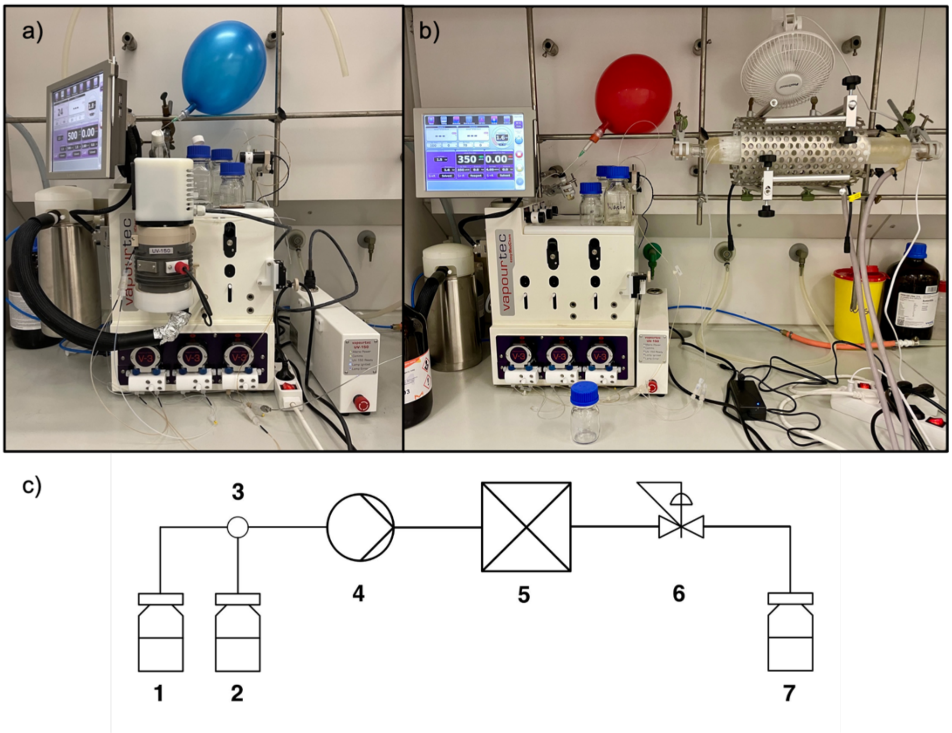


**Figure S3:** Vaportec® E-Series continuous flow machine, connected to a Vapourtec® UV-150 photochemical reactor (a); connected to a custom-made photochemical reactor (b). Flow chart of the flow reactor set-up (c): reaction mixture (1), solvent reservoir (2), split (3), pump (4), photoreactor (5), back pressure regulator (6), and collector (7).

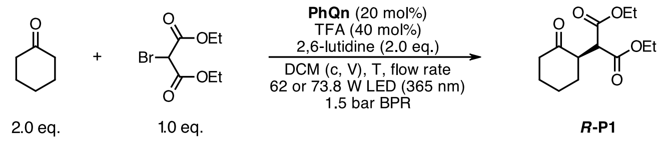

All flow photoreactions were carried out in septum-closed vials sealed with parafilm and were prepared using the standard Schlenk technique. The reaction mixture was prepared according to the general procedure for batch photoreactions. E.g. for a 0.1 M concentrated reaction mixture, **PhQn** (16.0 mg, 0.04 mmol, 0.2 eq.) was dissolved in anhydrous DCM (0.1 M regarding the limiting component, 2.0 mL). To this solution, TFA (9.1 mg, 0.08 mmol, 0.4 eq.), cyclohexanone (39.3 mg, 0.40 mmol, 2.0 eq.), diethyl 2-bromomalonate (47.2 mg, 0.20 mmol, 1.0 eq.) and 2,6-lutidine (42.9 mg, 0.40 mmol, 2.0 eq.) was added. The empty vial was evacuated and refilled with Ar three times *via* a syringe through the septum. The reaction mixture was added *via* syringe through the septum under Ar counterflow. The septum was pierced with a needle, and a tubing with the proper connection to the Vaportec® E-Series continuous flow machine was inserted above the solvent level. An Ar-filled balloon equipped with a needle was inserted through the septum above the solvent level to ensure a positive Ar pressure inside the vial when removing it from the Schlenk line. One end of the tubing was connected to the pump system, and the other was introduced into the reaction mixture. The tubing system was conditioned with the same solvent as used for the reaction mixture, the temperature was set, and the UV irradiation was switched on before the reaction mixture was pumped at a certain flow rate through the photochemical reactor. Once the reaction mixture was consumed, pure solvent was extracted. Mentionable, dilution of the reaction mixture was inevitable, but since all experiments were equally executed, its effect on the result´s comparability can be neglected. The collected reaction mixture was evaporated, and the residue was dissolved in DCM (5 mL). To this solution, an internal standard (*n*-dodecane, 50 µL) was added. An aliquot was taken for GC and chiral HPLC measurement.

## 7.2. Optimization for the 25 mL photoreactor

Following the general procedure for flow experiments, the parameters (i) concentration, (ii) flow rate, and (iii) temperature were optimized for the 25 mL photoreactor. The best result was obtained when the reaction was conducted at 0.1 M, 350 µL/min, and 10 °C, whereas the same considerations as for the 10 mL photoreactor are true. Under these optimized conditions, the desired product was obtained with 28% yield, 47 µmol/h productivity, and 84% ee.

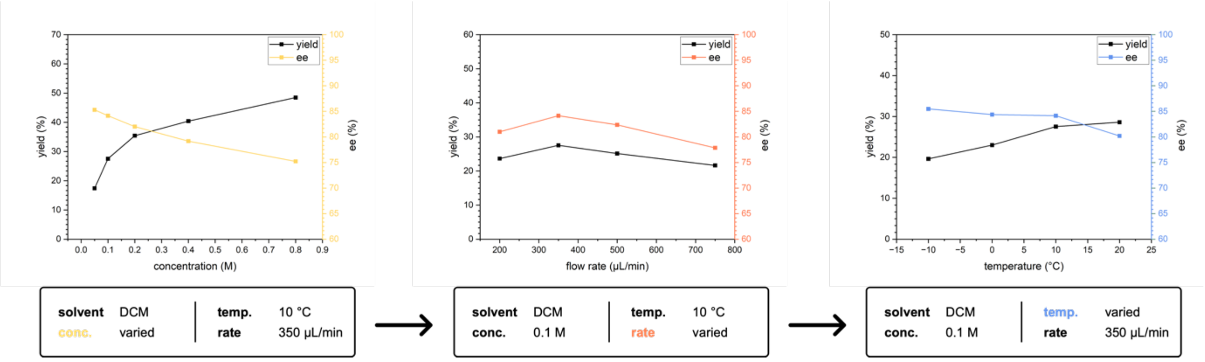


**Figure S4:** Parameter optimization for the 25 mL photoreactor.

# 8. Light on/off experiment

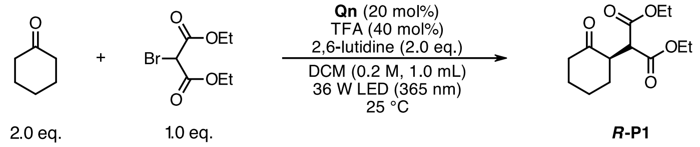


The light on/off experiment was performed in an 8 mL Schlenk tube using standard Schlenk technique. **Qn** (0.04 mmol, 0.2 eq.) was dissolved in anhydrous DCM (0.2 M regarding the limiting component, 1.0 mL). To this solution, TFA (0.08 mmol, 0.4 eq.), cyclohexanone (0.40 mmol, 2.0 eq.), diethyl 2-bromomalonate (0.20 mmol, 1.0 eq.), and 2,6 lutidine (0.40 mmol, 2.0 eq.) were added. After evacuating and refilling the Schlenk tube with Ar three times, the reaction mixture was added under Ar counterflow. The Schlenk tube was sealed and placed into the custom-made photoreactor, and the reaction mixture was stirred at 25 °C. The reaction mixture was irradiated for 30 min intervals (light on), followed by 30 min dark periods (light off) for a total of 480 min. Every 30 min, an aliquot was taken using standard Schlenk technique, and the conversion was determined by GC analysis. Then, the remaining reaction mixture was irradiated for additional 600 min (18 h total time), followed by the determination of the conversion by GC analysis.

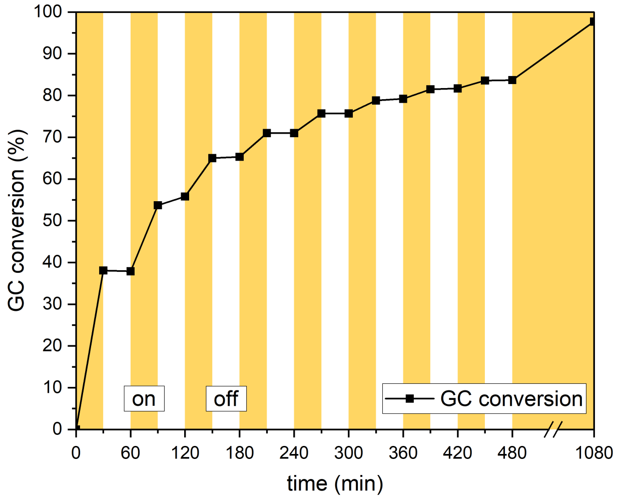


**Figure S5:** Light on/off experiment.

# 9. NMR Experiments to prove **PhQn** condensation

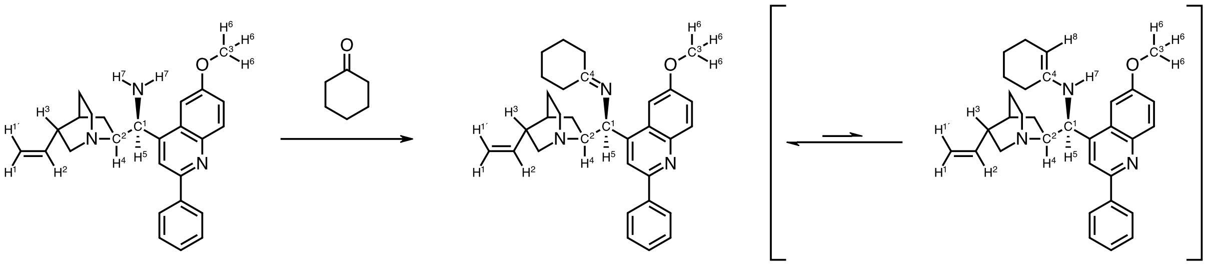


To prove the formation of condensed species upon mixing **PhQn** and cyclohexanone, we conducted *in-situ* NMR experiments. In an NMR tube, **PhQn** (19.98 mg, 50.0 µmol) and cyclohexanone (5.17 µL, 50.0 µmol) were dissolved in 1.0 mL DMSO-$d_6$ to obtain a 50 mM solution. We selected DMSO-$d_6$ as solvent as it demonstrated supportive properties for tertiary enamine formation, using *L*-proline as the secondary amine.[25] NMR spectra were measured at 297 K on a Bruker® Advance UltraShield ($^1$H: 600 MHz, $^{13}$C{$^1$H}: 151 MHz) spectrometer. Simulated NMR spectra were created using the integrated tool within the MestReNova® Software.

In the experimental $^1$H-NMR spectra of the mixture (Figure S6.a), additional signals to the components are present, indicating a reaction. E.g., an additional $H^2$-signal (ddd, *J* = 17.5, 10.3, 7.4 Hz) and an additional $H^6$-signal (s, at 3.92 ppm) (not shown in Figure S6) are prominently visible. When comparing Figure S6.a with the simulated $^1$H-NMR spectra of the imine (Figure S6.b) and of the enamine (Figure S6.c), the formation of an enamine was excluded, since no Enamine-$H^8$ nor an Enamine-$H^7$ signal (not shown in Figure S6) were visible. Since an additional $H^5$-signal (d, *J* = 9.3 Hz) shifted to higher ppm values and additional $H^1$ and $H^{1'}$-signals at 4.94 ppm (dt, *J* = 17.2, 1.6 Hz) and at 4.83 ppm (dt, *J* = 10.5, 1.4 Hz) were visible the formation of an imine was suggested. The connections of the spin systems were proven with a COSY measurement (Figure S6.d). The Imine-$H^2$ signal shows cross peaks to Imine-$H^3$ (hidden beneath a cyclohexanone signal) and to Imine-$H^1$ and $H^{1'}$, and the Imine-$H^5$ signal shows a cross peak to Imine-$H^4$ (q, J = 9.1 Hz).

An HMBC measurement (Figure S6.e) provided further insight. The Imine-$H^5$ signal (Imine-$C^1$ at 61.73 ppm) shows cross peaks to Imine-$C^2$ (s, at 59.99 ppm), to signals of the quinoline moiety, and to a signal at 171.67 ppm. The cross peak assignment between the H and C signals was accomplished with an HSQC measurement. Given that the signal at 171.67 ppm displayed cross peaks solely to the aliphatic region in addition to the mentioned cross peak to Imine-$H^5$ (not shown in Figure S6) in the HMBC spectra, along with its high chemical shift (compare Figure S6.f and g) and the absence of cross peaks in the HSQC spectra, it was concluded that this signal was assigned to Imine-$C^4$, providing evidence of imine formation. Notably, no condensation reaction was observed in the NMR spectra, using $CD_2Cl_2$ as solvent.

The formation of enantioenriched **P1** according to GP6, thereby involving the activation of the cyclohexanone α-position and relying solely on **PhQn** for stereochemical control, aligns with literature[26] evidence supporting the unfavored *in-situ* equilibrium of the imine with a secondary enamine.

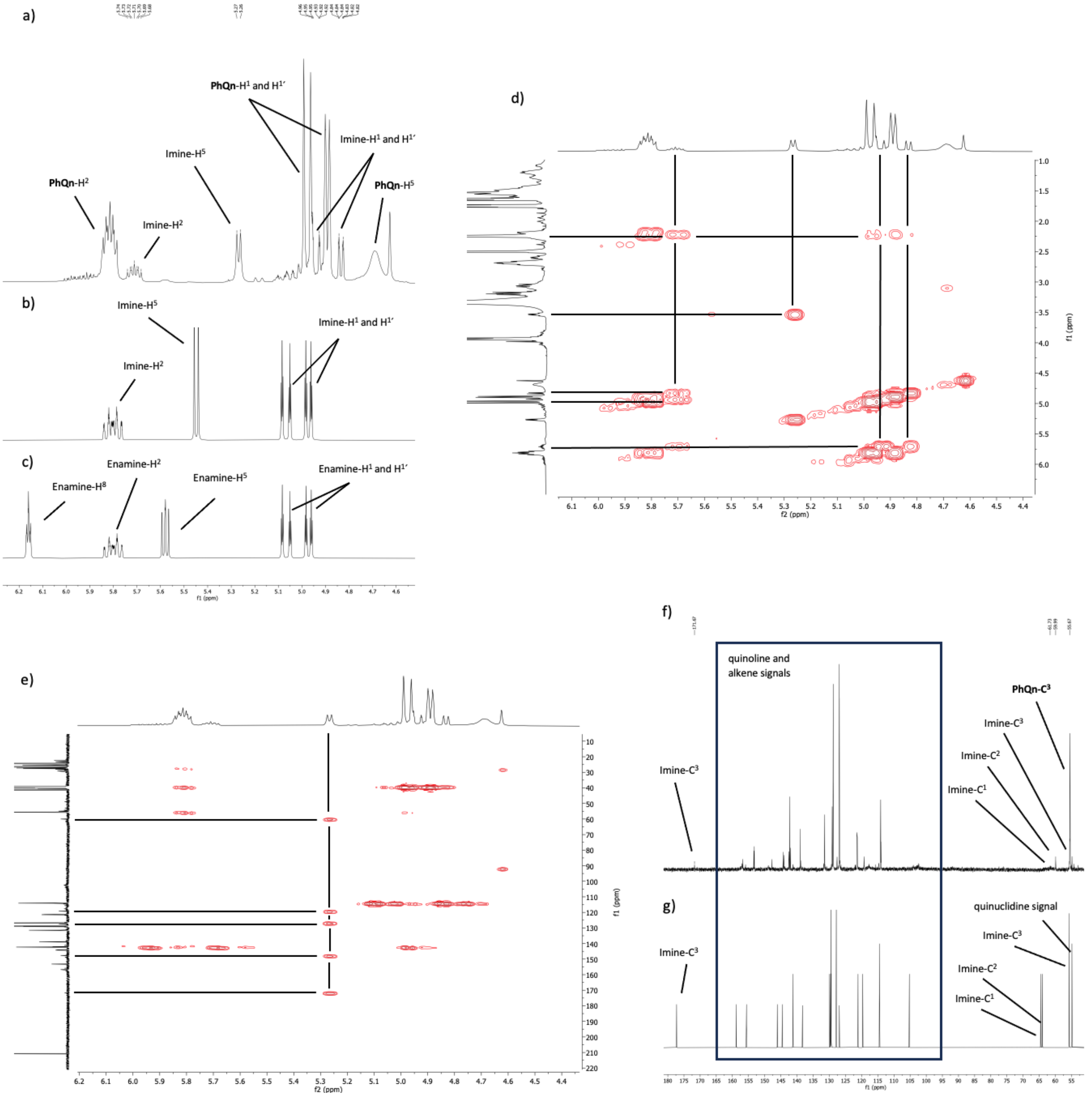


**Figure S6:** $^1$H NMR spectra of **PhQn** and cyclohexanone (1:1) in DMSO-d$_6$ (a). Simulated spectra of the imine (b). Simulated spectra of the enamine (c). COSY spectra of **PhQn** and cyclohexanone (1:1) in DMSO-d$_6$ (d). HMBC spectra of PhQn and cyclohexanone (1:1) in DMSO-d$_6$ (e). $^{13}$C{$^1$H} NMR spectra of **PhQn** and cyclohexanone (1:1) in DMSO-d$_6$ (f). Simulated spectra of the imine (g).

# 10. Quantum yield determination

The determination of the photon flux of the custom-made photoreactor and the calculation of the quantum yield were performed according to a modified literature procedure.[27]

## 10.1. Synthesis of potassium ferrioxalate trihydrate

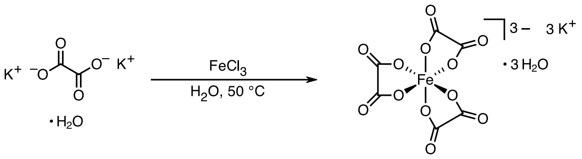


Potassium oxalate monohydrate (2.93 g, 15.9 mmol, 3.0 eq.) was dissolved in distilled $H_2O$ (10 mL), followed by the addition of $FeCl_3$ (0.86 g, 5.30 mmol, 1.0 eq.). The reaction mixture was stirred at 50 °C for 2 h and was then cooled to 0 °C for 18 h. The green crystals were filtrated, washed with distilled $H_2O$, dried, and stored in the dark. Potassium ferrioxalate trihydrate was obtained as a green crystalline solid (1.84 g, 70% yield).

**IR** 3434, 1711, 1663, 1646, 1379, 1269, 1255, 891, 787, 647 $cm^{-1}$

Analytical data was in accordance with literature.[28]

## 10.2. Determination of the photon flux

Ferrioxalate solution: Potassium ferrioxalate trihydrate (147.4 mg, 0.30 mmol) and sulfuric acid (96%, 69.5 µL) were placed in a 25 mL volumetric flask and filled up with distilled $H_2O$.

1,10-Phenanthroline solution: 1,10-Phenanthroline (100.0 mg, 0.55 mmol) was placed in a 50 mL volumetric flask and filled with distilled $H_2O$.

Buffer solution: NaOAc (4.94 g, 60.2 mmol) and sulfuric acid (96%, 1.0 mL) were placed in a 100 mL volumetric flask and filled up with distilled $H_2O$.

The photon flux of the custom-made photoreactor was determined using the ferrioxalate actinometer. Four 8 mL Schlenk tubes were charged with Ferrioxalate solution (1.0 mL) and irradiated in the custom-made photoreactor for 3, 5, 10, and 15 s. The irradiated samples and a non-irradiated sample (0 s) were transferred into a 10 mL volumetric flask, followed by the addition of 1,10-phenanthroline solution (0.5 mL), buffer solution (2.0 mL) and it was filled up with distilled $H_2O$. The samples were wrapped in aluminum foil to avoid light exposure. After a period of 1 h at 25 °C, the mixtures were analyzed by UV/Vis spectroscopy (Figure S7).

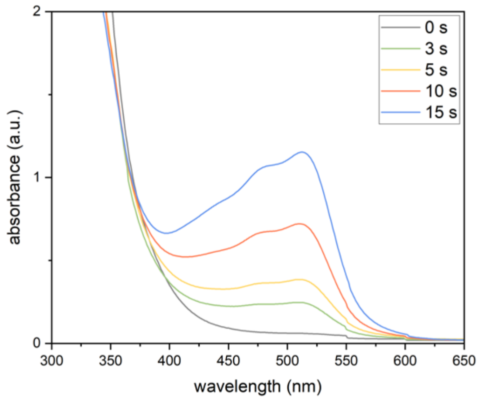


**Figure S7:** UV/Vis spectra of ferrioxalate actinometer mixtures after 0 to 15 s irradiation time.

The moles of formed ferrous iron ($Fe^{2+}$) in each sample were calculated according to the Lambert-Beer equation (Eq. 1).

$$n(Fe^{2+}) = \frac{V \cdot \Delta A(510\ nm)}{l \cdot \varepsilon(510\ nm)} \qquad \text{(Eq. 1)}$$

| | |
|---|---|
| $n(Fe^{2+})$ | … amount of substance of ferrous iron (mol) |
| $V$ | … final volume after complexation with phenanthroline (0.01 L) |
| $l$ | … optical path length (1 cm) |
| $\Delta A(510\ nm)$ | … absorption difference of the irradiated and the non-irradiated sample at 510 nm |
| $\varepsilon(510\ nm)$ | … extinction coefficient of ferroin at 510 nm (11100 L · $mol^{-1}$ · $cm^{-1}$) |

The amount of substance of ferrous iron ($Fe^{2+}$) is plotted as a function of time (Figure S8).

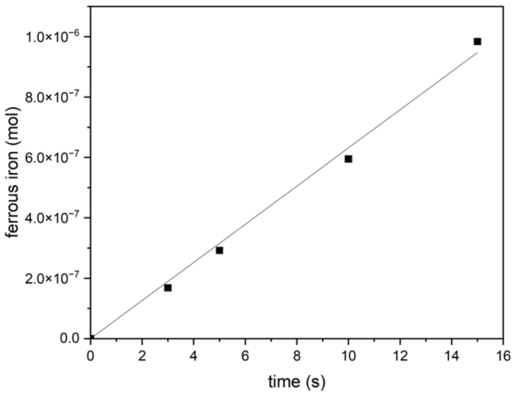


**Figure S8:** Moles of formed ferrous iron versus the irradiation time. y = 6.31E-08x, $R^2$ = 9.97E-01.

The slope of this straight line is used to determine the moles of incident photons by the unit of time by the custom-made photoreactor (Eq. 2).

$$\text{photon flux} = \frac{\text{slope}_{\text{actinometer}}}{\Phi_{\text{actinometer}}(\lambda)\cdot\left[1-10^{-A_{\text{ferrioxalate}}(\lambda)}\right]} \qquad \text{(Eq. 2)}$$

$\text{photon flux}$ … photon flux of the custom-made photoreactor (Einstein · $s^{-1}$)

$\text{slope}_{\text{actinometer}}$ … moles of formed ferrous ions by the unit of time (mol ·$s^{-1}$)

$\Phi_{\text{actinometer}}(\lambda)$ … quantum yield of potassium ferrioxalate at 365 nm (1.27)[29]

$A_{\text{ferrioxalate}}(\lambda)$ … absorption of the ferrioxalate solution at 365 nm (1.054)

The photon flux of the custom-made photoreactor was calculated as **5.45 × $10^{-8}$ Einstein $s^{-1}$**.

## 10.3. Determination of the quantum yield

Standard solution: Ethyl benzoate (330.0 mg, 2.20 mmol) was placed in a 5 mL volumetric flask and filled with DCM.

In four parallel experiments, **Qn** (12.9 mg, 0.04 mmol, 0.2 eq.) was dissolved in anhydrous DCM (1.0 mL), followed by the addition of TFA (9.1 mg, 0.08 mmol, 0.4 eq.), cyclohexanone (39.3 mg, 0.40 mmol, 2.0 eq.), diethyl 2-bromomalonate (47.8 mg, 0.20 mmol, 1.0 eq.) and 2,6-lutidine (42.9 mg, 0.40 mmol, 2.0 eq.). After evacuating and refilling with Ar for three times, each reaction mixture was transferred into an 8 mL Schlenk tube in Ar counterflow. The samples were irradiated in the custom-made photoreactor for 10, 20, 30, and 40 min. Then, a standard solution (150 µL) was added to each sample, and the yield was determined from an aliquot *via* GC analysis. The amount of substance of the product is plotted against the number of absorbed photons (Figure S9).

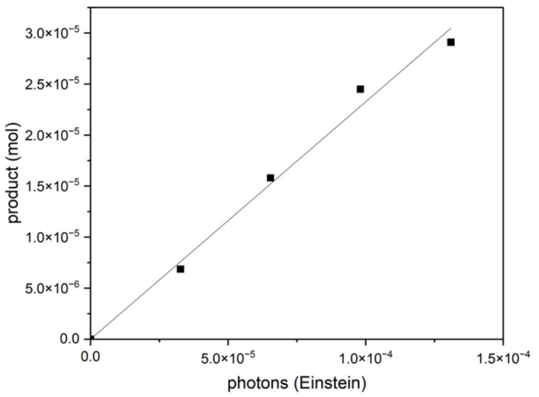


**Figure S9:** Moles of formed product versus the number of absorbed photons. y = 2.33E-01x, $R^2$ = 9.97E-01.

The slope of this straight line is used for the determination of the reaction´s quantum yield (Eq. 3).

$$\Phi_{\text{reaction}}(\lambda) = \frac{\text{slope}_{\text{reaction}}}{\text{photon flux} \cdot \left[1 - 10^{-A_{\text{reaction}}(\lambda)}\right]} \qquad \text{(Eq. 3)}$$

| | |
|---|---|
| $\Phi_{\text{reaction}}(\lambda)$ | … quantum yield of the reaction at 365 nm |
| photon flux | … photon flux of the custom-made photoreactor (Einstein · $s^{-1}$) |
| $\text{slope}_{\text{reaction}}$ | … moles of the formed product by the unit of absorbed photons |
| $A_{\text{reaction}}(\lambda)$ | … absorption of the catalyst-free reaction mixture at 365 nm (0.199) |

The quantum yield of the model reaction was calculated as **0.63**.

# 11. Structure determination of *R*-R2 by single-crystal X-ray diffraction

## 11.1. Experimental

Crystallization was achieved by slow evaporation of a methanolic solution. Suitable single crystals of ***R*-R2** were preselected under a polarizing microscope, embedded in perfluorinated polyether, and mounted on MiTeGen® loops. The single crystal X-ray diffraction measurement was performed on a Bruker® AXS APEXII four-circle diffractometer equipped with a CCD camera. Intensity data were collected at –173 °C using graphite monochromatized Mo *K*α radiation (λ = 0.71073 Å). Correction for absorption effects was carried out with the multi-scan approach of SADABS.[30] The crystal structure was solved by SHELXT and was refined by the full-matrix least-squares technique on $F^2$ with the SHELXL[31] program package.[32] H atoms were positioned geometrically (C–H = 0.95-1.00 Å) and were refined as riding with $U_{iso}$(H) = 1.2$U_{eq}$(C) for aromatic, methine and methylene H atoms and with $U_{iso}$(H) = 1.5$U_{eq}$(C) for methyl H atoms. The H atom bonded to the N atom was located from a difference-Fourier map. It was refined with a bond length of N–H = 0.88 Å and with $U_{iso}$(H) = 1.2$U_{eq}$(N).

Deposition number 2313387 contains the supplementary crystallographic data for this paper. These data are provided free of charge by the joint Cambridge Crystallographic Data Centre and Fachinformationszentrum Karlsruhe Access Structures service at https://www.ccdc.cam.ac.uk/structures.

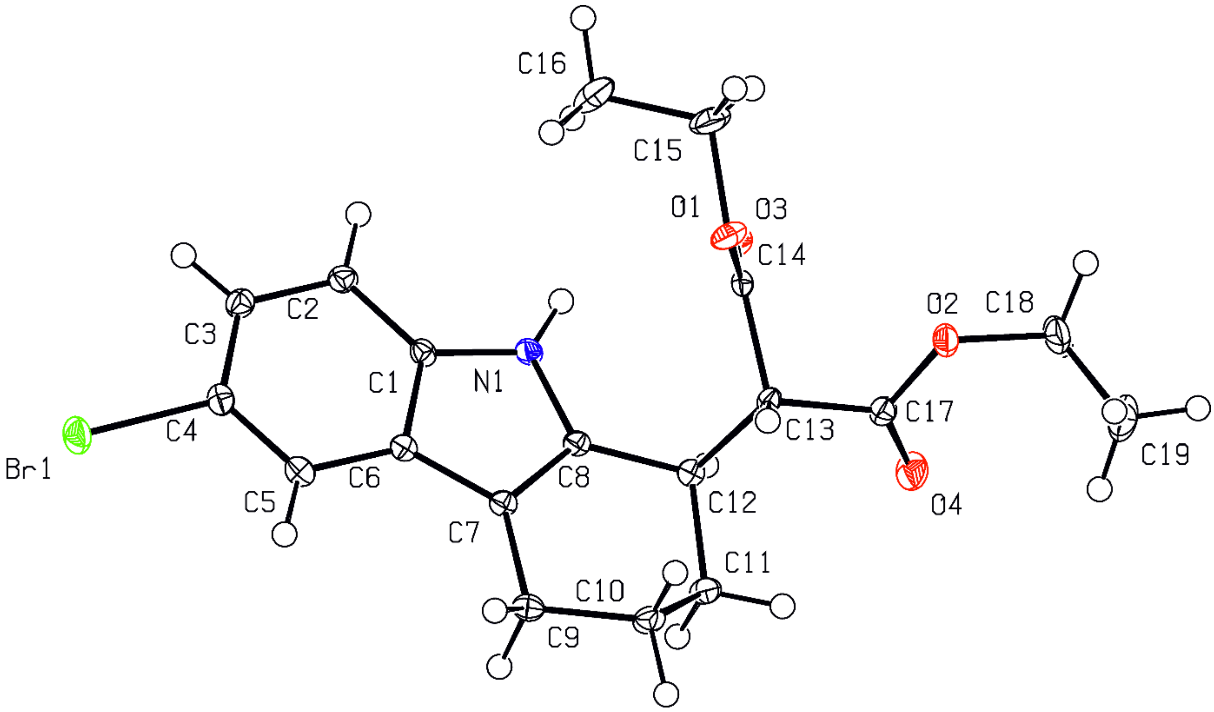


**Figure S10:** Molecular structure of ***R*-R2** with atom labeling scheme and displacement ellipsoids drawn at the 50% probability level.

**Table S1:** Crystal Data and Details of the Structure Determination for: ***R*-R2**

| | | |
|---|---|---|
| **Crystal Data** | Formula | $C_{19}H_{22}BrNO_4$ |
| | Formula Weight | 408.28 |
| | Crystal System | monoclinic |
| | Space group | $P2_1/c$ (No. 14) |
| | *a*, *b*, *c* /Å | 10.3479(6), 19.0329(10), 9.8952(5) |
| | $\alpha$, $\beta$, $\gamma$ /° | 90, 110.989(1), 90 |
| | *V* / $Å^3$ | 1819.56(17) |
| | *Z* | 4 |
| | D(calc) /g $cm^{-3}$ | 1.490 |
| | $\mu$(MoKa) /$mm^{-1}$ | 2.282 |
| | F(000) | 840 |
| | Crystal Size /$mm^3$ | 0.21 x 0.38 x 0.39 |
| **Data Collection** | Temperature /K | 100 |
| | Radiation /Å | MoKa /0.71073 |
| | Theta Min-Max [Deg] | 2.1, 34.6 |
| | Dataset (h;k;l) | -16:16; -30:30; -15:15 |
| | Tot., Uniq. Data, R(int) | 45410, 7723, 0.052 |
| | Observed Data [I > 2.0 sigma(I)] | 5958 |
| **Refinement** | Nref, Npar | 7723, 228 |
| | *R*, w*R*2, S with w = ^2^(FO^2^)+(0.0264P)^2^+1.2135P] WHERE P=(FO^2^+2FC^2^)/3' | 0.0355, 0.0746, 1.01 |
| | Max. and Av. Shift/Error | 0.00, 0.00 |
| | Min. and Max. Resd. Dens. /e $Å^{-3}$ | −0.44, 0.59 |

**Table S2:** Hydrogen Bonds / Å, ° for: ***R*-R2**

| ***D*–H···*A*** | ***D*–H** | **H···*A*** | ***D*···*A*** | ***D*–H···*A*** |
|---|---|---|---|---|
| N1–H1···O3 | 0.8800 | 2.3500 | 2.9678(16) | 128.00 |
| N1–H1···O3$^{i}$ | 0.8800 | 2.1100 | 2.8646(16) | 144.00 |

Symmetry code i) 1-x, 1-y, 2-z

# 12. Abbreviations

| | |
|---|---|
| $Boc_2O$ | Di-*tert*-butyl decarbonate |
| BPR | Back pressure regulator |
| Bn | Benzyl |
| Bu | Butyl |
| CCD | Charge-coupled device |
| DBU | 1,8-Diazabicyclo(5.4.0)undec-7-ene |
| DCM | Dichloromethane |
| DEAD | Diethyl azodicarboxylate |
| DIPEA | *N*,*N*-Diisopropylethylamine |
| DMF | Dimethyl formamide |
| DMSO | Dimethyl sulfoxide |
| DPPA | Diphenylphosphoryl azide |
| EDA | Electron donor acceptor |
| ee | Enantiomeric excess |
| Et | Ethyl |
| GC | Gas chromatography |
| GCMS | Gas chromatography mass spectrometry |
| HPLC | High-performance liquid chromatography |
| HRMS | High-resolution mass spectrometry |
| LED | Light emitting diode |
| Me | Methyl |
| MTBE | Methyl *tert*-butyl ether |
| NBS | *N*-Bromsuccinimide |
| NMR | Nuclear magnetic resonance |
| OAc | Acetate |
| OTf | Triflate |
| OTs | Tosylate |
| PE | Petrolether |
| Ph | Phenyl |
| Pr | Propyl |
| SET | Single electron transfer |
| TBDMS | *tert*-Butyl-trimethylsilyl |
| TEMPO | 2,2,6,6-tetramethylpiperidine-1-oxyl |
| TFA | Trifluoroacetic acid |
| THF | Tetrahydrofuran |
| TLC | Thin layer chromatography |
| TMS | Trimethylsilyl |
| UV/Vis | Ultraviolet/Visible light |
| XRD | X-ray diffraction |

# 13. NMR Spectra

(*S*)-Proline-*N*-ethyl carbamate methyl ester (**1**)

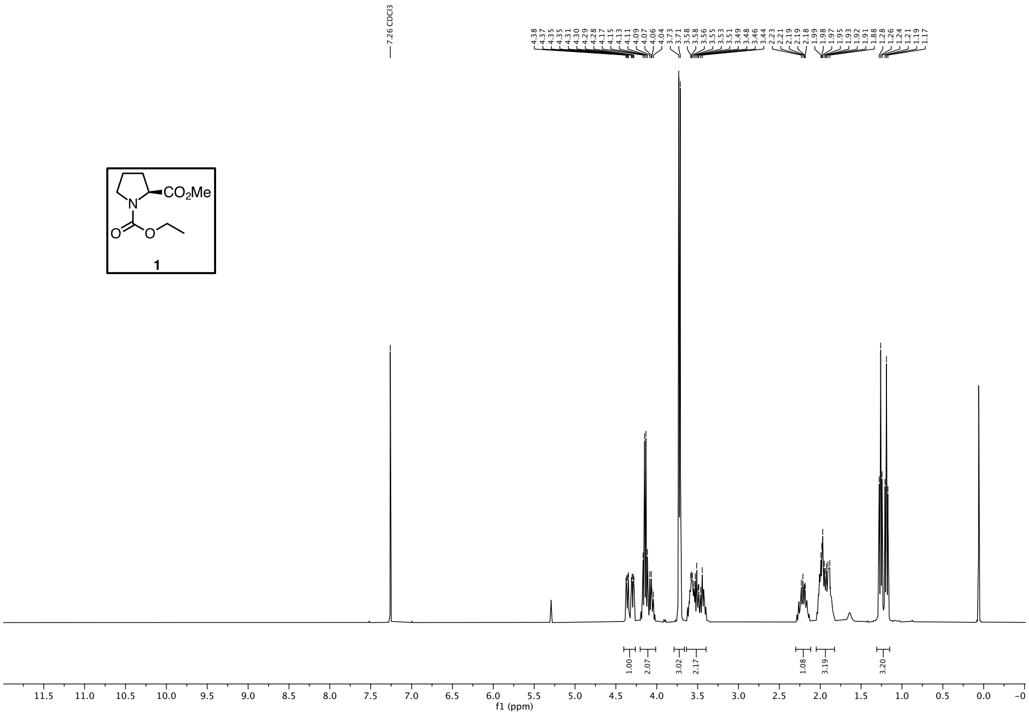

**Figure S11:** $^1H$ NMR Spectra of **1** (400 MHz, $CDCl_3$).

(*S*)-2-[Bis(3,5-bis(trifluoromethyl)phenyl)hydroxymethyl]pyrrolidine-1-carboxylic acid ethyl ester (**2**)

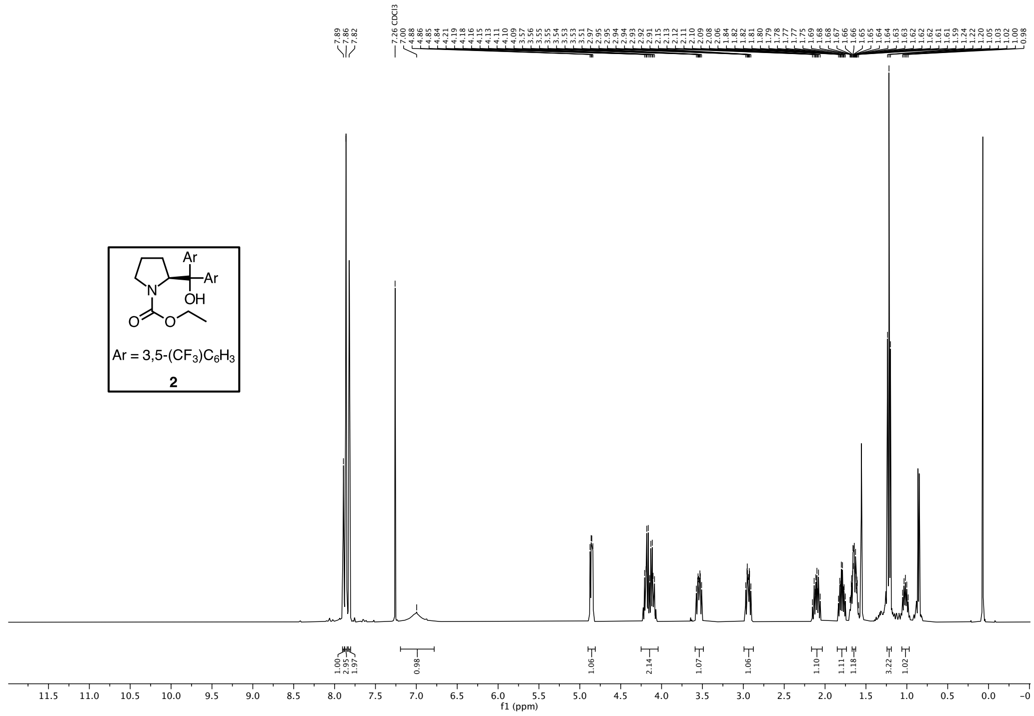

**Figure S12:** $^1H$ NMR Spectra of **2** (400 MHz, $CDCl_3$).

(*S*)-Bis(3,5-bis(trifluoromethyl)phenyl)(pyrrolidin-2-yl)methanol (**3**)

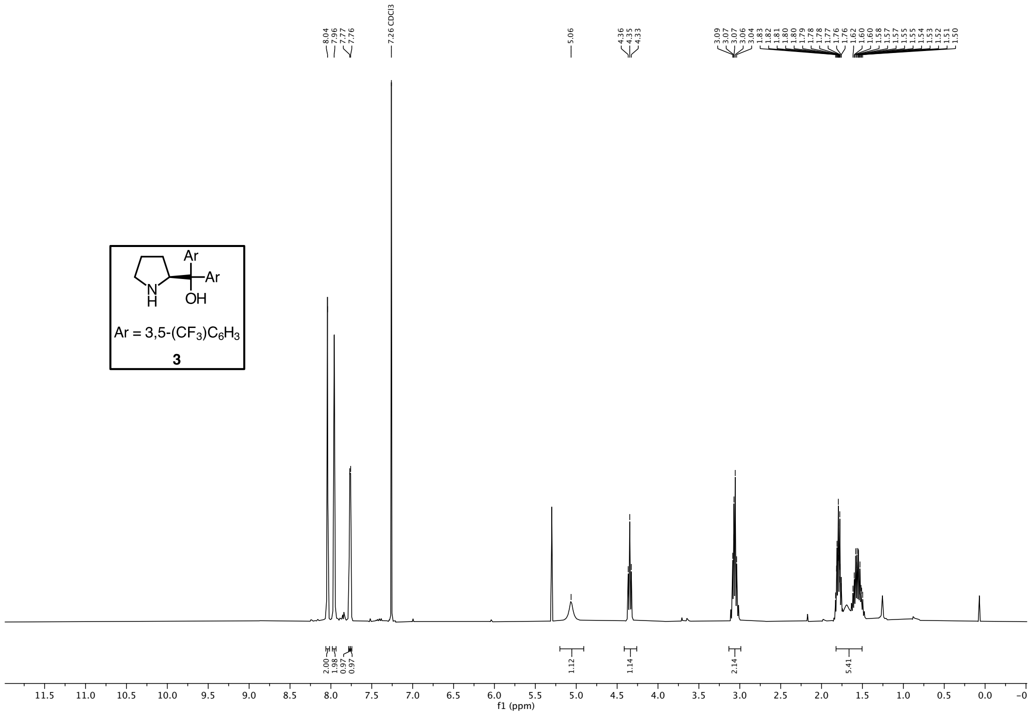

**Figure S13:** $^{1}$H NMR Spectra of **3** (400 MHz, $CDCl_3$).

(*S*)-2-{Bis[3,5-bis(trifluoromethyl)phenyl][(trimethylsilanyl)oxy]methyl}pyrrolidine (**A1**)

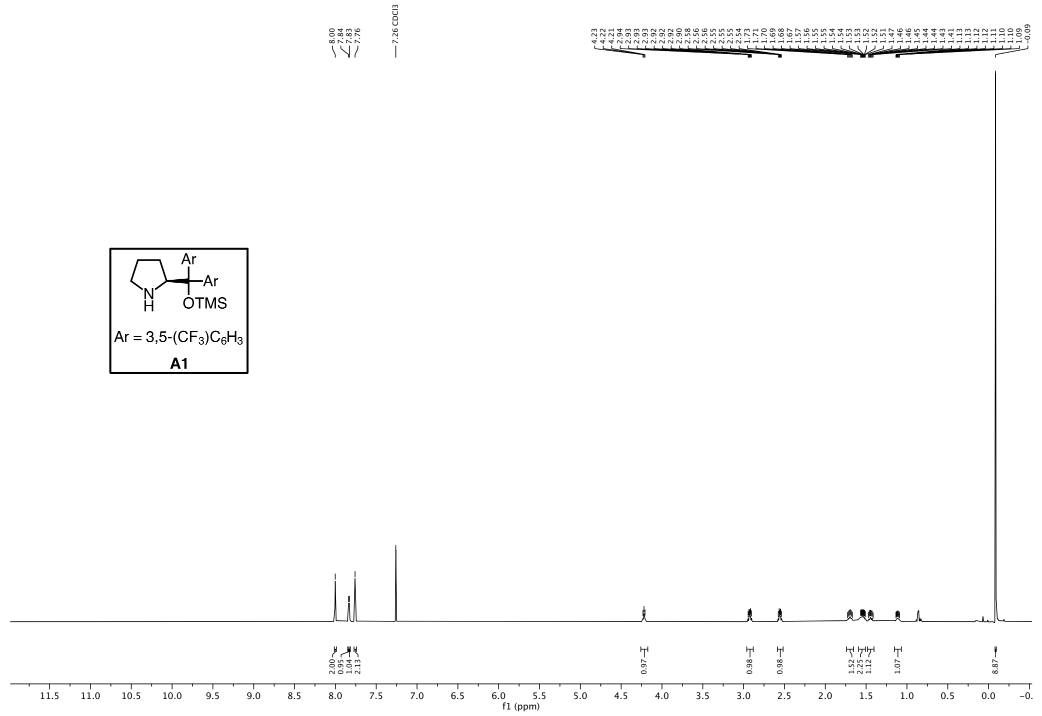

**Figure S14:** $^{1}$H NMR Spectra of **A1** (400 MHz, $CDCl_3$).

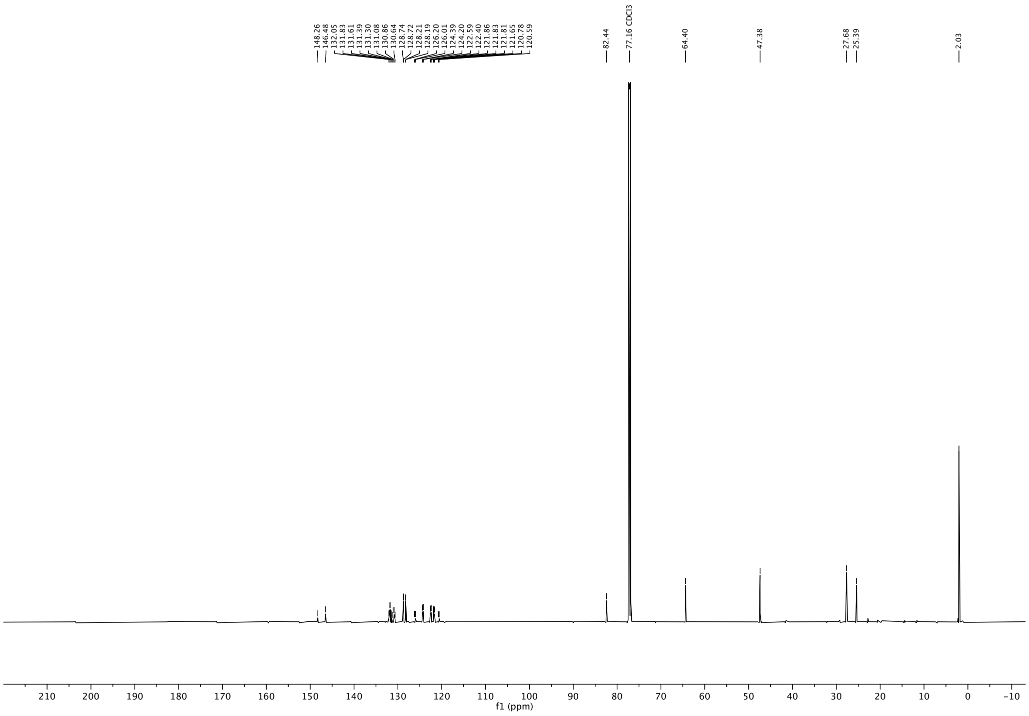

**Figure S15:** $^{13}C\{^1H\}$ NMR Spectra of **A1** (101 MHz, $CDCl_3$).

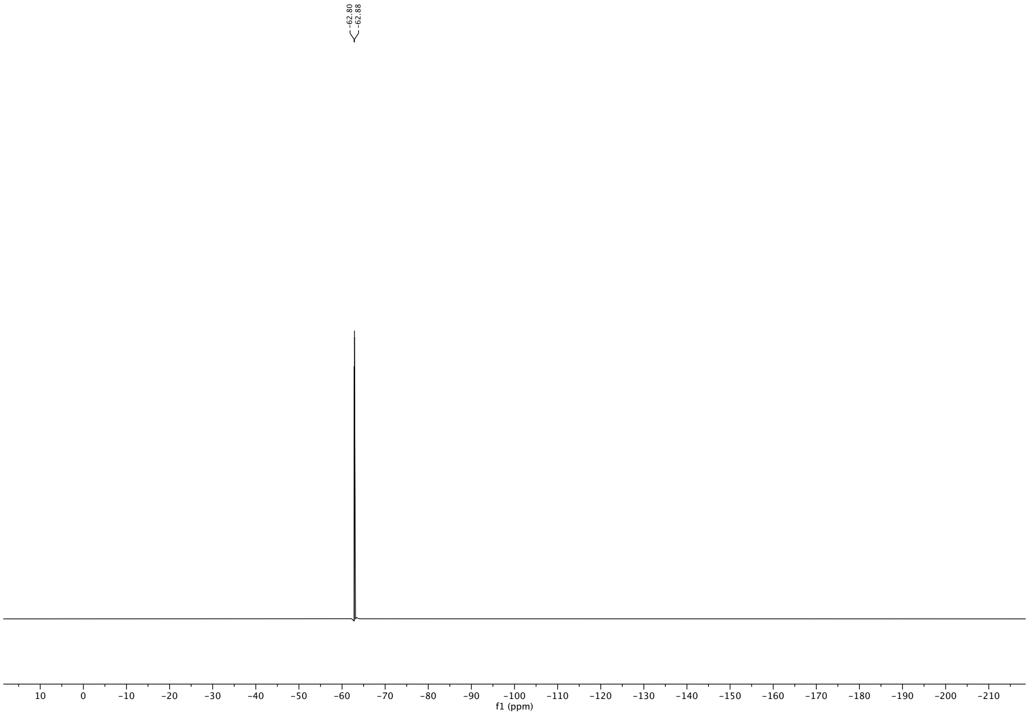

**Figure S16:** $^{19}F\{^1H\}$ NMR Spectra of **A1** (376 MHz, $CDCl_3$).

(*S*)-2-{Bis[3,5-bis(trifluoromethyl)phenyl][(*tert*-butyldimethylsilyl)oxy]methyl}pyrrolidine (**A2**)

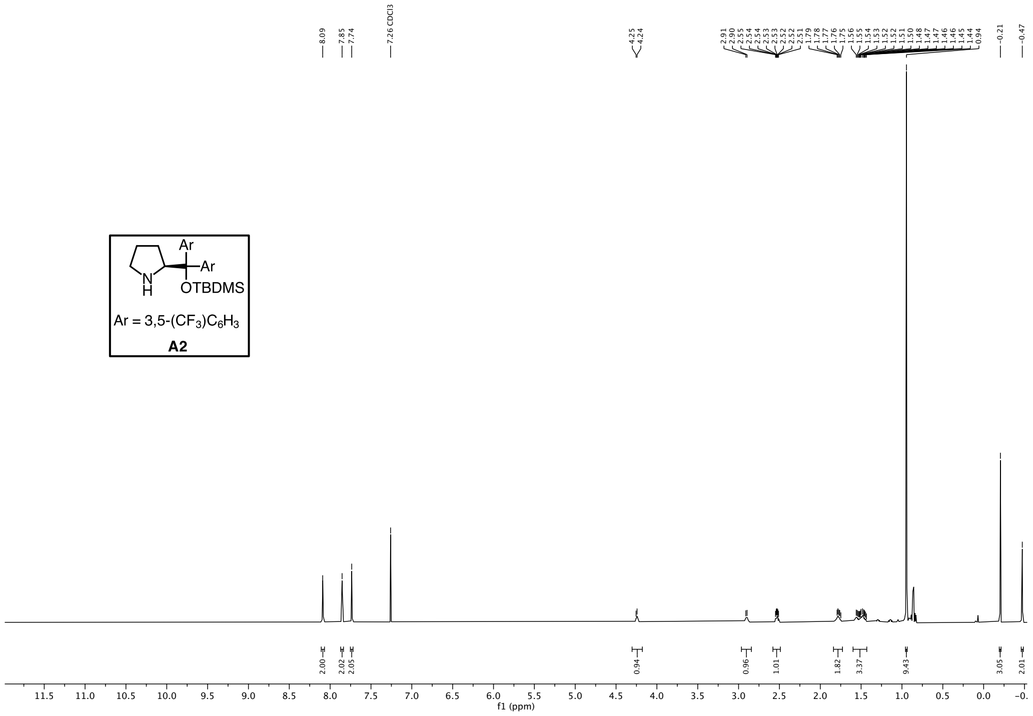

**Figure S17:** $^1$H NMR Spectra of **A2** (400 MHz, $CDCl_3$).

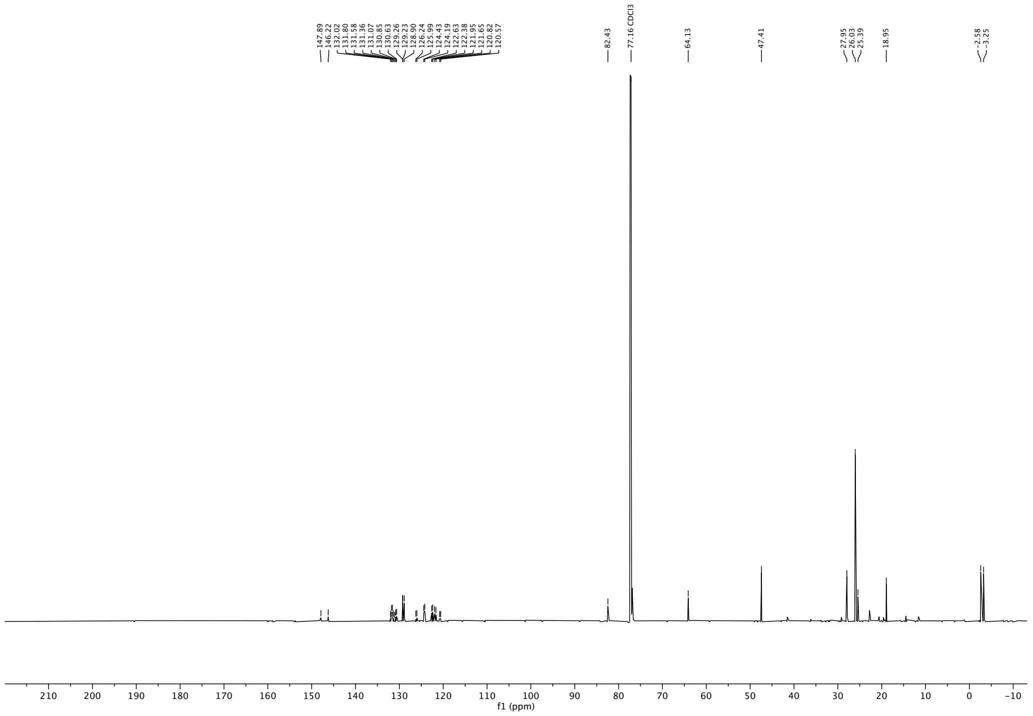

**Figure S18:** $^{13}C\{^1H\}$ NMR Spectra of **A2** (101 MHz, $CDCl_3$).

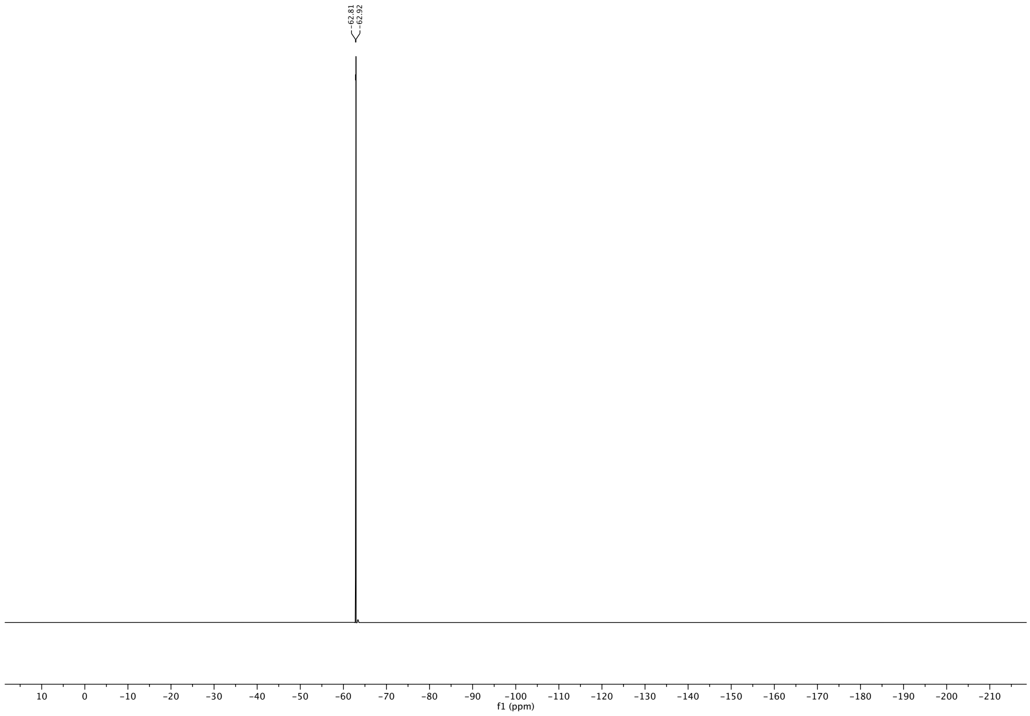

**Figure S19:** $^{19}F\{^{1}H\}$ NMR Spectra of **A2** (376 MHz, $CDCl_3$).

## 1-((1*R*,2*R*)-2-aminocyclohexyl)-3-(3,5-bis(trifluoromethyl)phenyl)thiourea (**A3**)

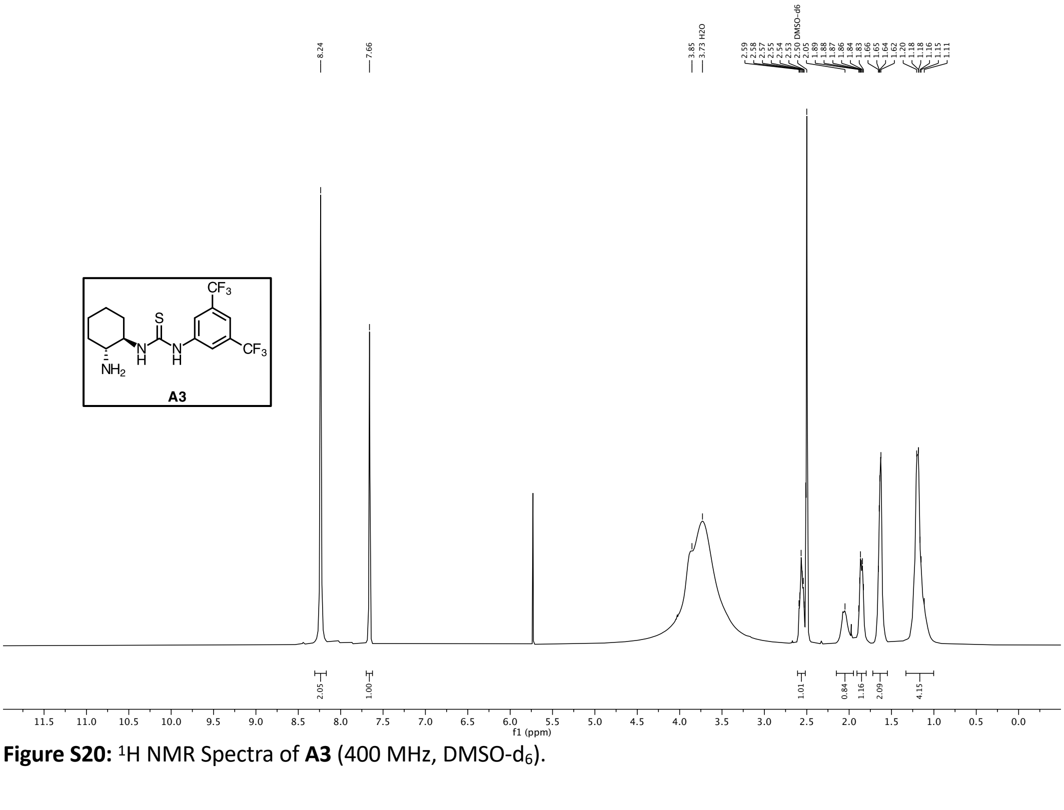


**Figure S20:** $^{1}H$ NMR Spectra of **A3** (400 MHz, DMSO-$d_6$).

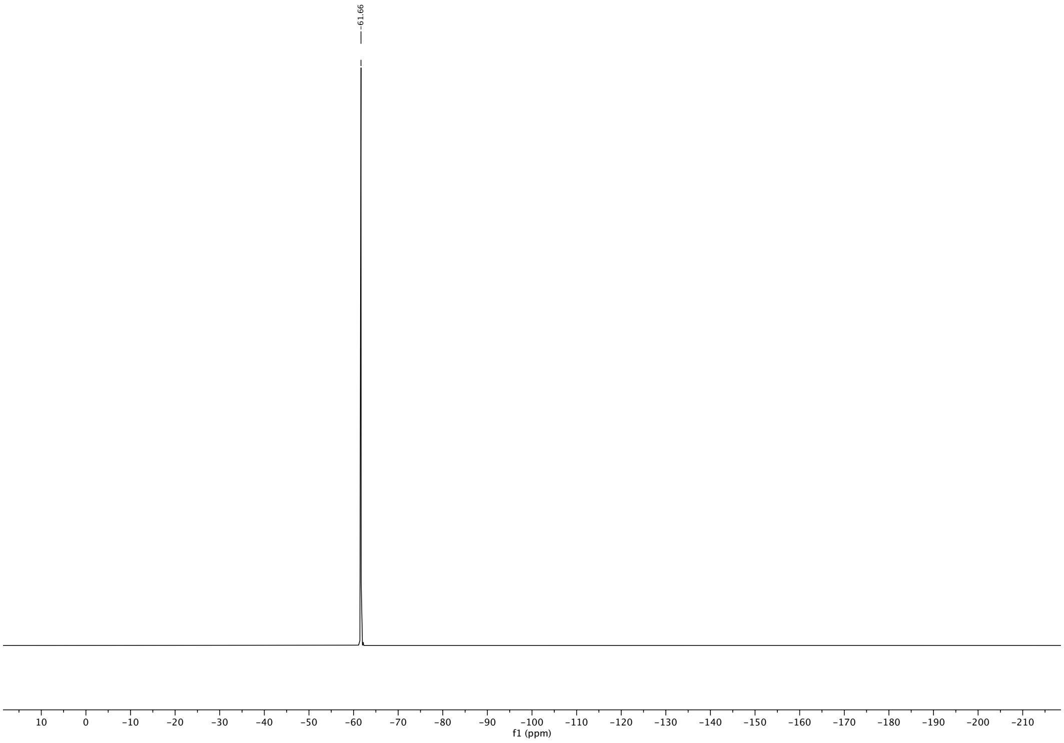


**Figure S21:** $^{19}F\{^1H\}$ NMR Spectra of **A3** (376 MHz, DMSO-d$_6$).

## 9-amino-9-deoxy-*epi*-quinine (**Qn**)

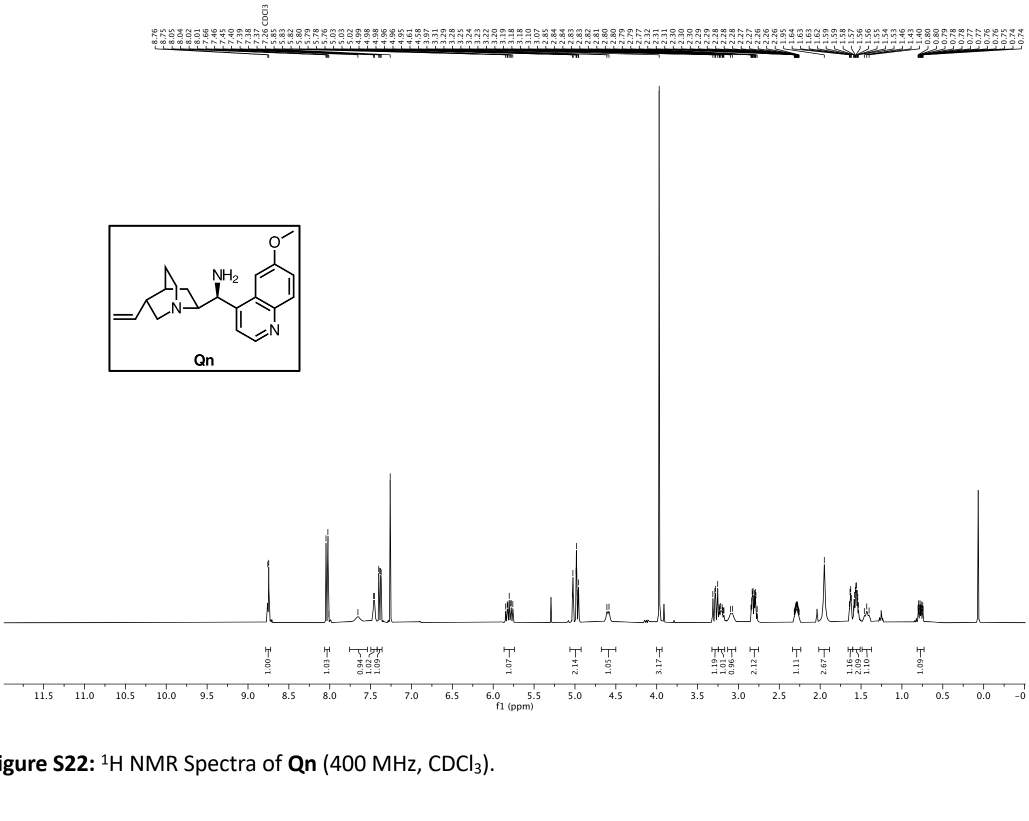


**Figure S22:** $^1H$ NMR Spectra of **Qn** (400 MHz, $CDCl_3$).

9-amino-9-deoxy-*epi*-cinchonidine (**Cd**)

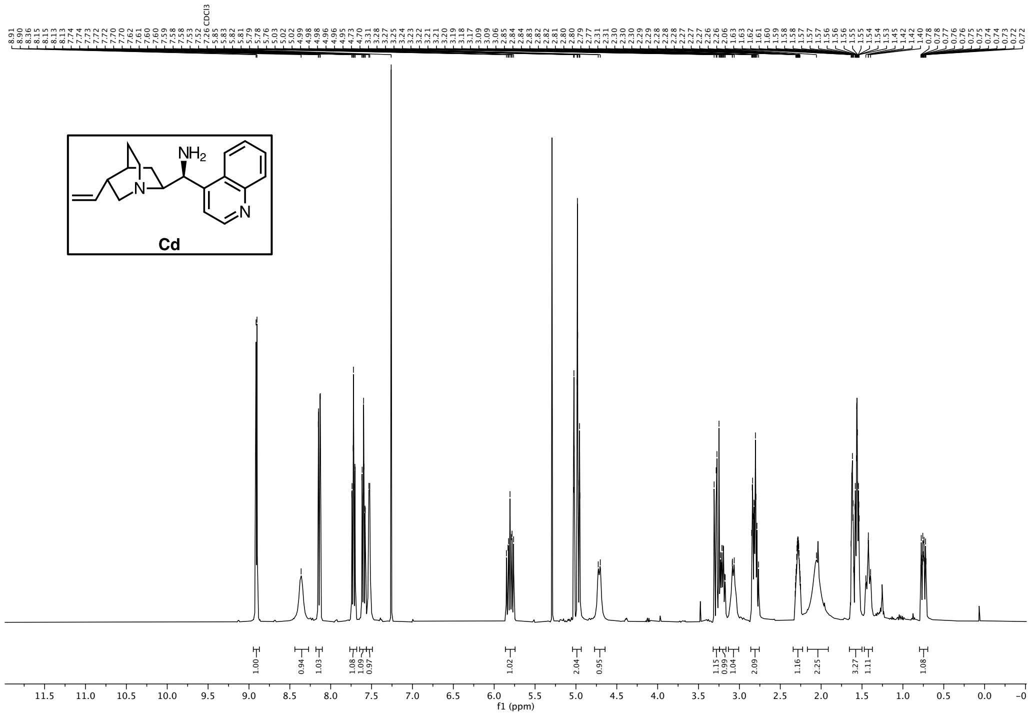


**Figure S23:** $^{1}$H NMR Spectra of **Cd** (400 MHz, $CDCl_3$).

9-amino-9-deoxy-*epi*-quinidine (**Qd**)

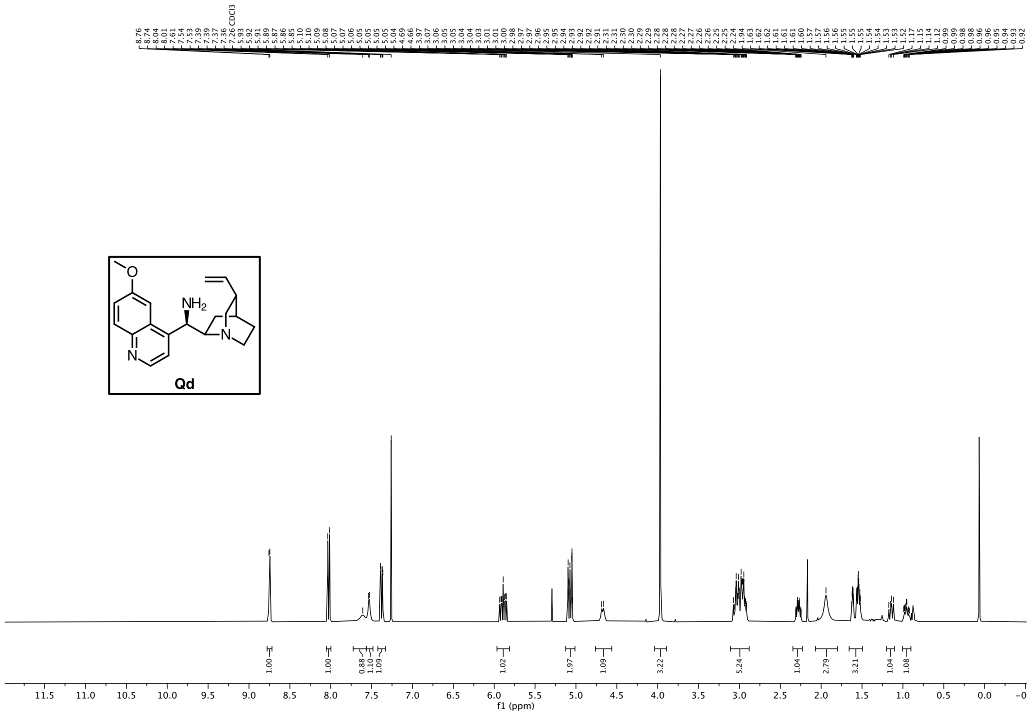


**Figure S24:** $^{1}$H NMR Spectra of **Qd** (400 MHz, $CDCl_3$).

9-amino-9-deoxy-*epi*-cinchonine (**Cn**)

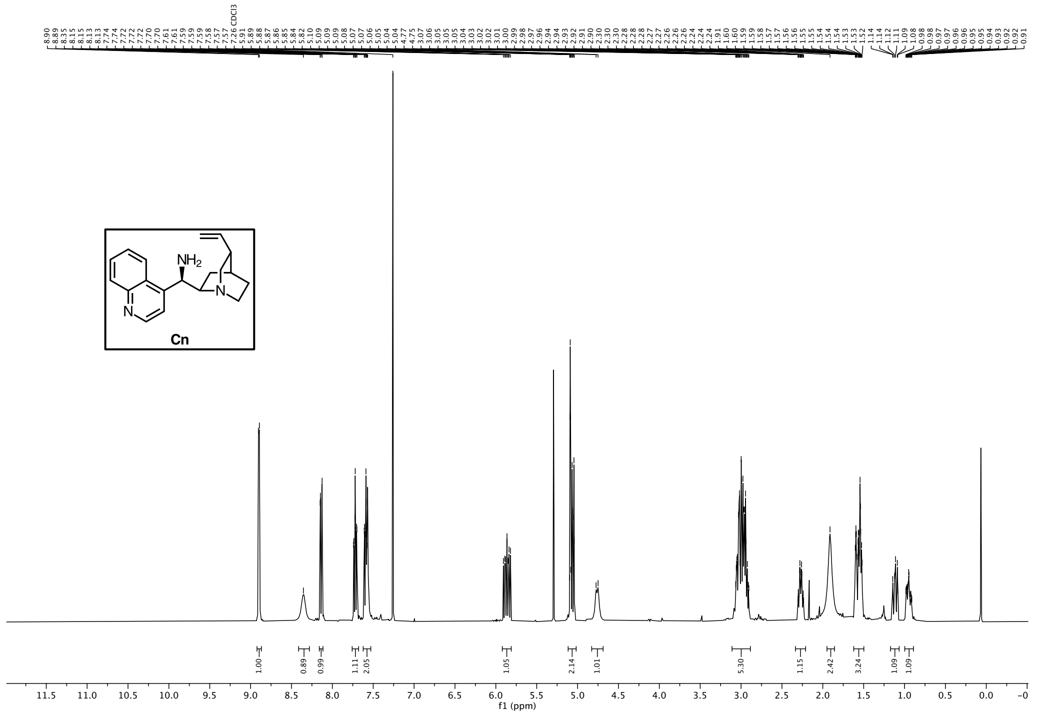

**Figure S25:** $^{1}$H NMR Spectra of **Cn** (400 MHz, $CDCl_3$).

2'-phenyl-quinine (**4**)

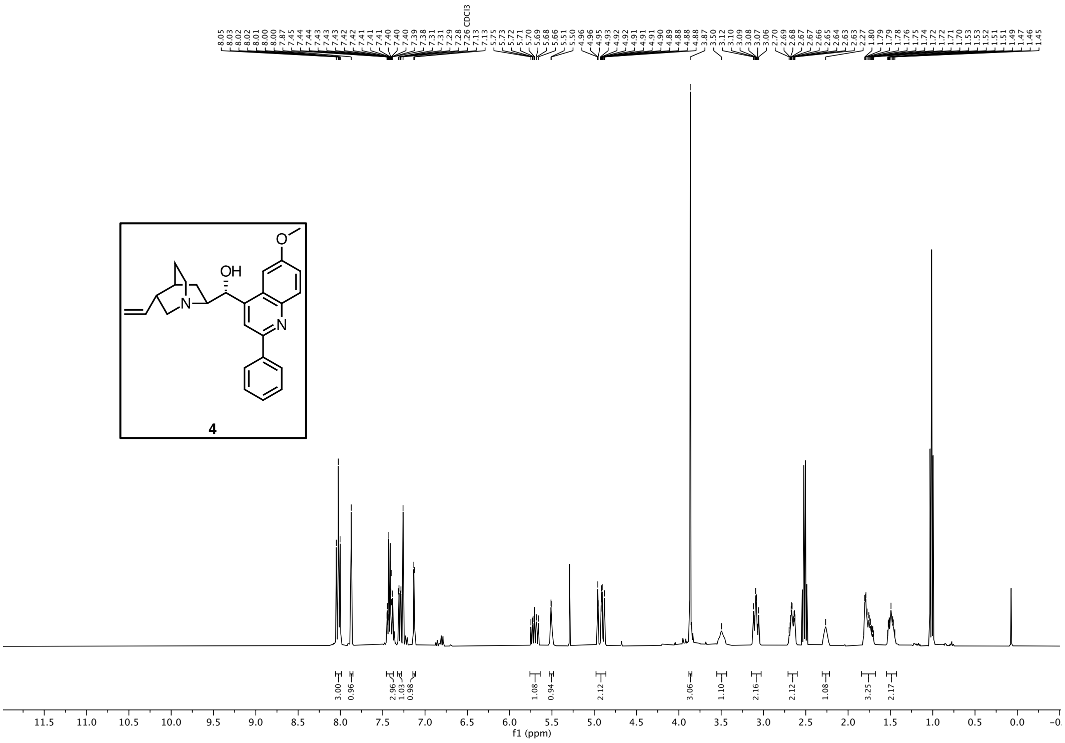

**Figure S26:**$^{1}$H NMR Spectra of **4** (400 MHz, $CDCl_3$).

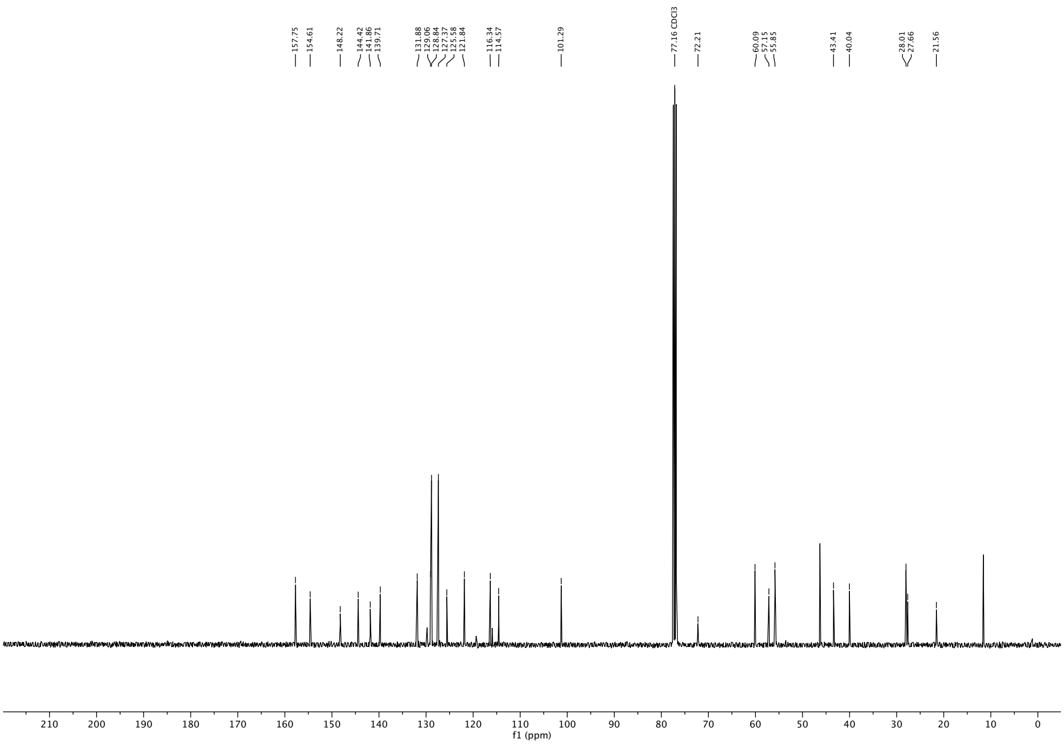


**Figure S27:** $^{13}C\{^{1}H\}$ NMR Spectra of **4** (101 MHz, $CDCl_3$).

## 2´-*n*-butyl-quinine (**5**)

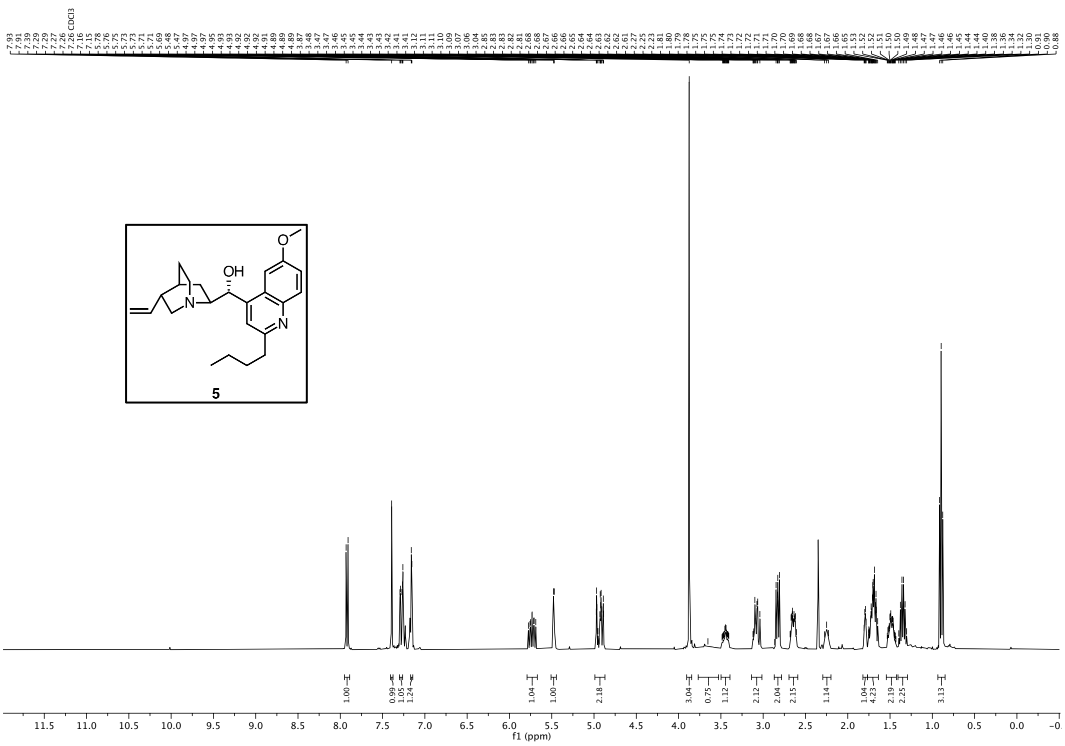


**Figure S28:** $^{1}H$ NMR Spectra of **5** (400 MHz, $CDCl_3$).

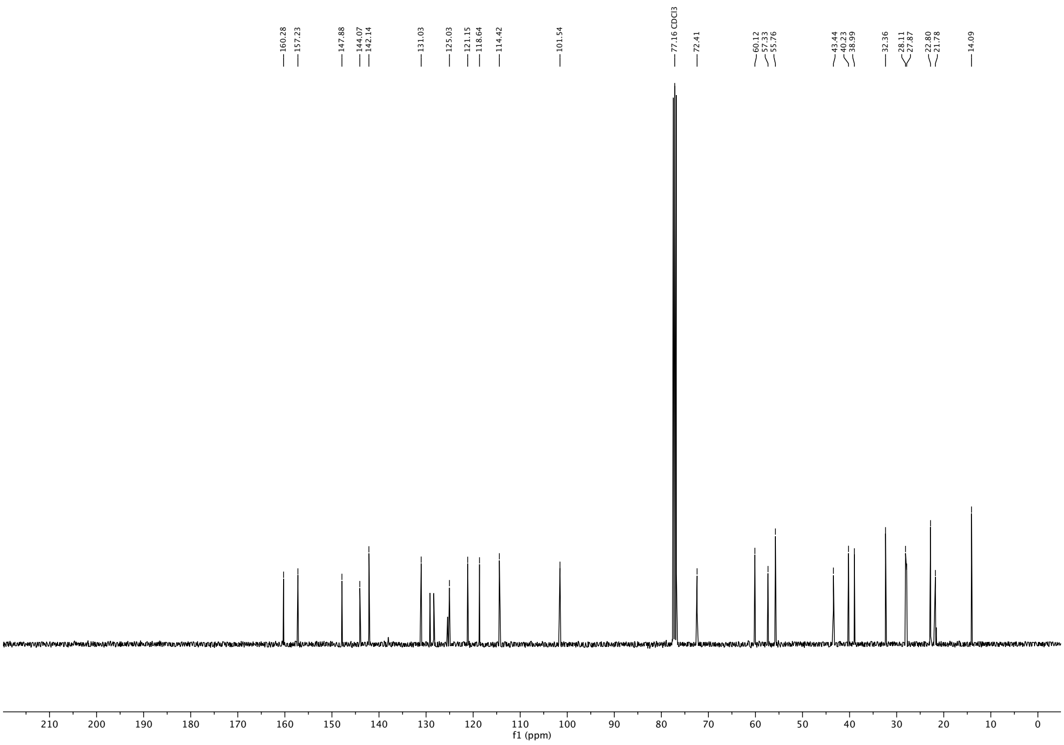


**Figure S29:** $^{13}C\{^{1}H\}$ NMR Spectra of **5** (101 MHz, $CDCl_3$).

# 2´-phenyl-quinidine (6)

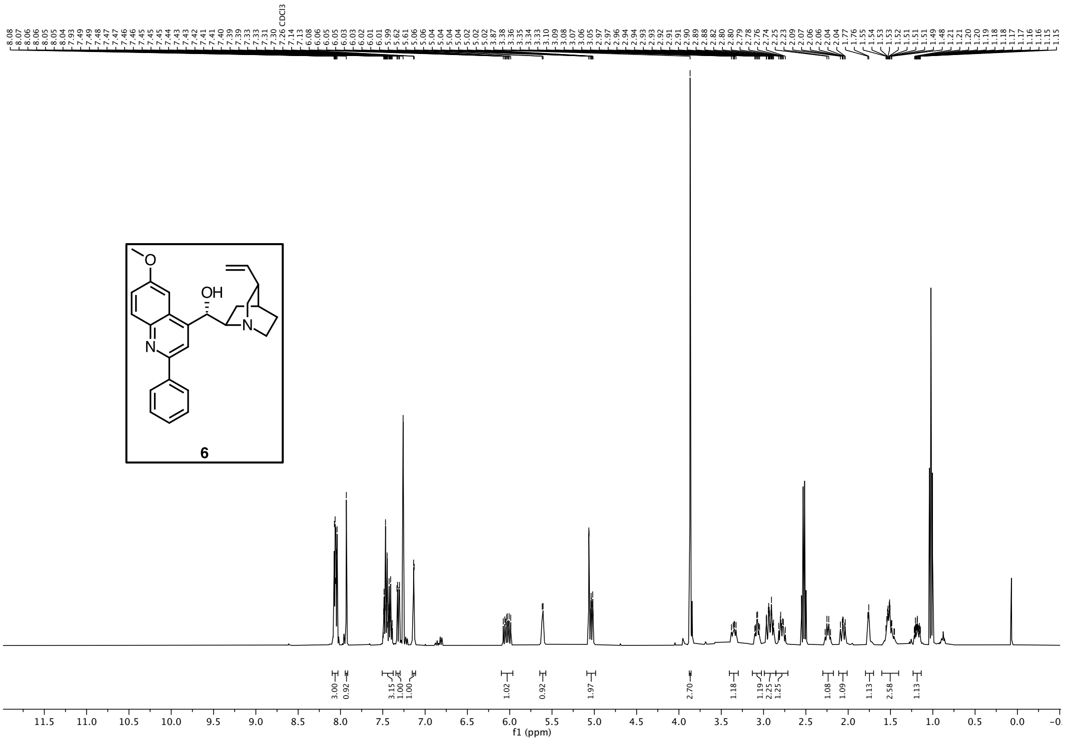


**Figure S30:** $^{1}H$ NMR Spectra of **6** (400 MHz, $CDCl_3$).

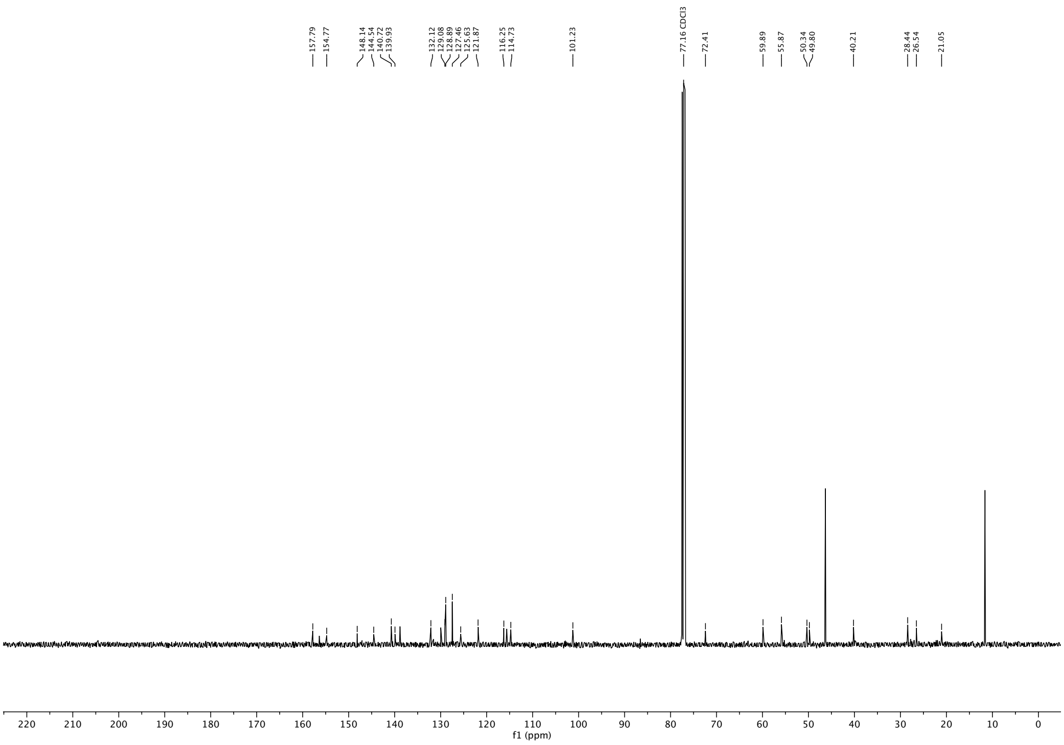

**Figure S31:** $^{13}C\{^1H\}$ NMR Spectra of **6** (101 MHz, $CDCl_3$).

(1*S*)-(6-methoxy-2-phenylquinolin-4-yl)((2*S*,4*S*,5*R*)-5-vinylquinuclidin-2-yl)methanamine (**PhQn**)

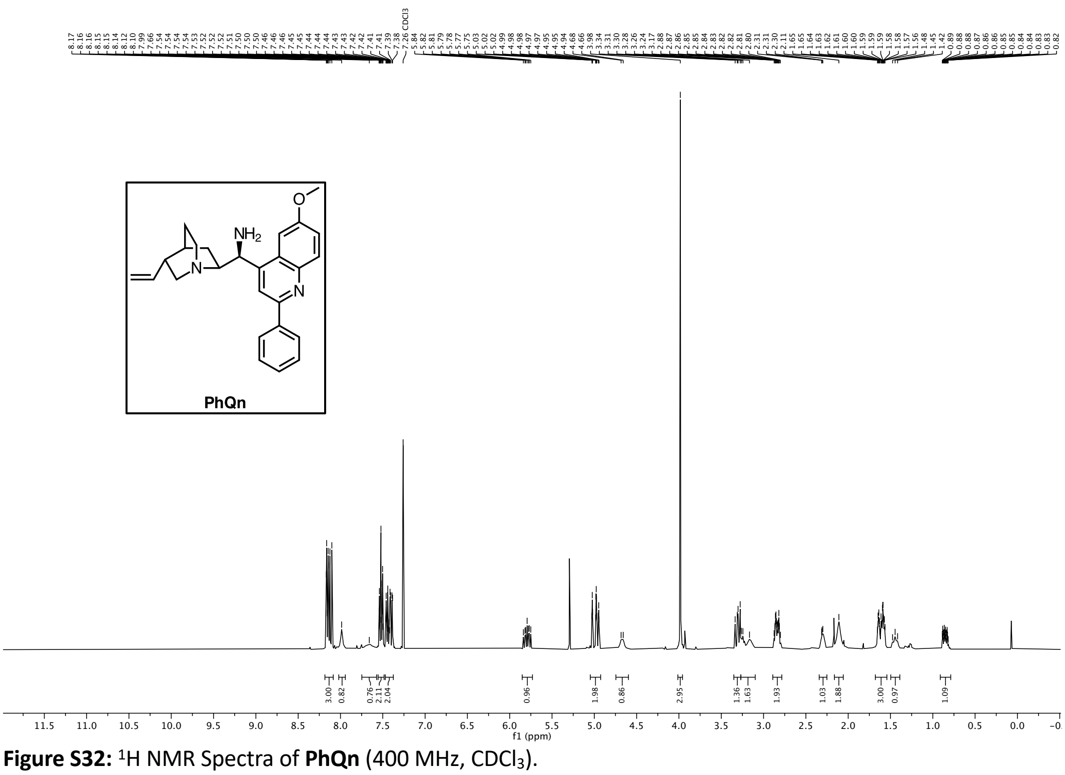

**Figure S32:** $^1H$ NMR Spectra of **PhQn** (400 MHz, $CDCl_3$).

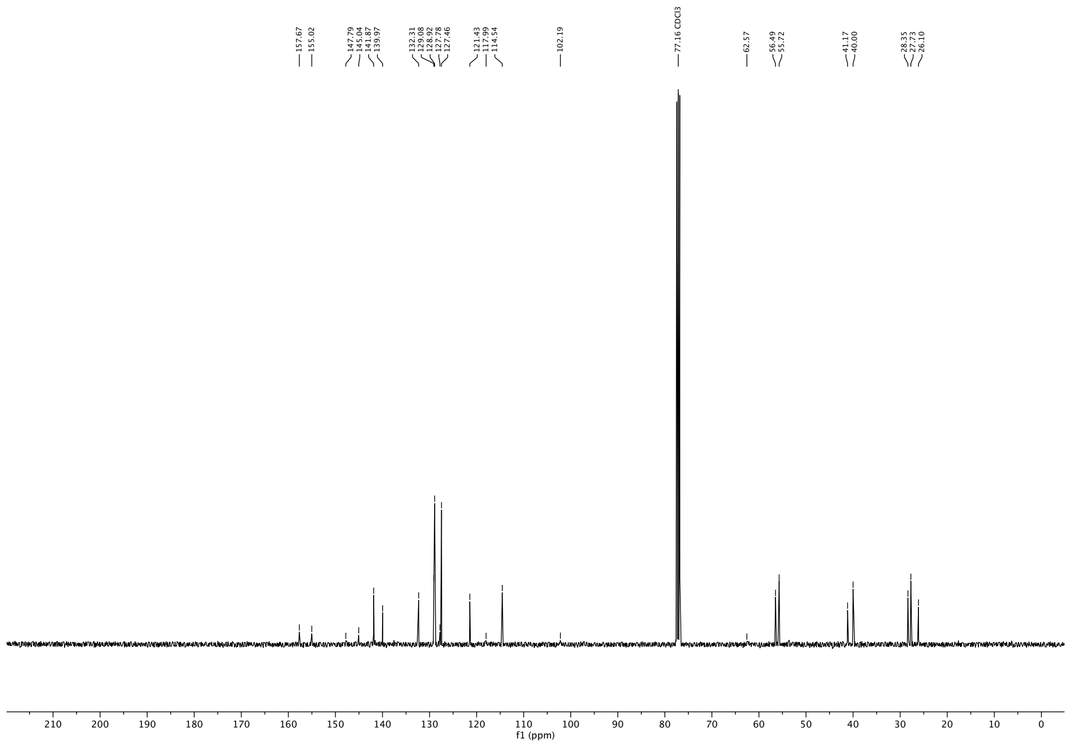


**Figure S33:** $^{13}C\{^{1}H\}$ NMR Spectra of **PhQn** (101 MHz, $CDCl_3$).

(1*S*)-(2-butyl-6-methoxyquinolin-4-yl)((2*S*,4*S*,5*R*)-5-vinylquinuclidin-2-yl)methanamine (**BuQn**)

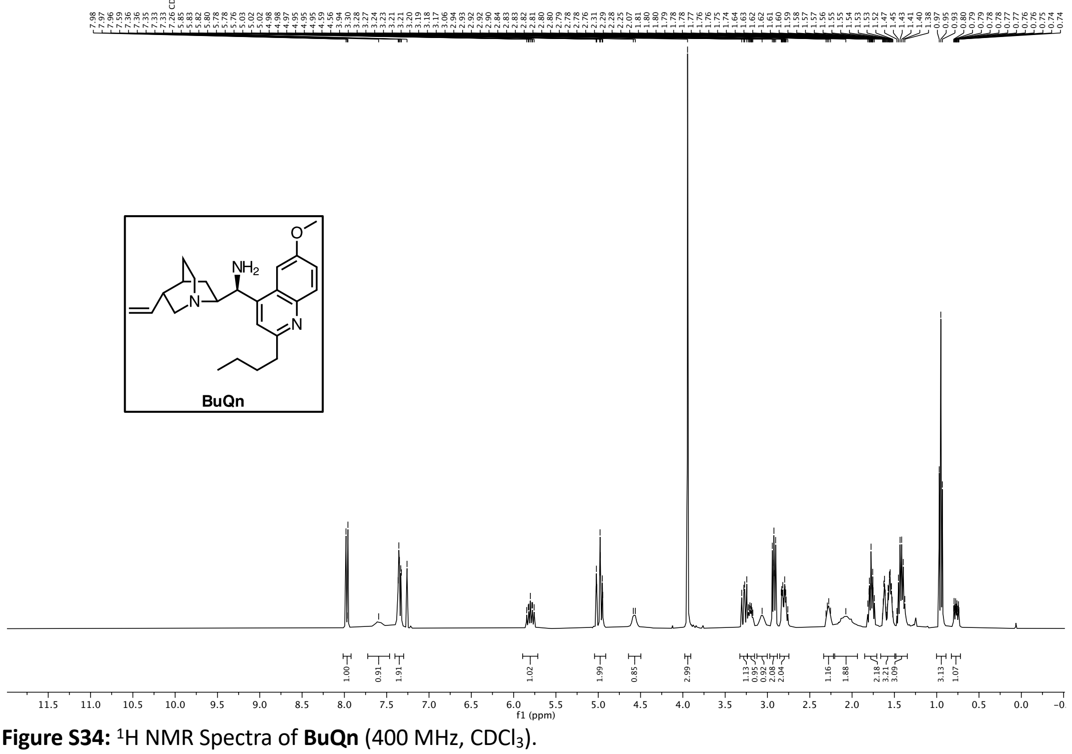


**Figure S34:** $^{1}H$ NMR Spectra of **BuQn** (400 MHz, $CDCl_3$).

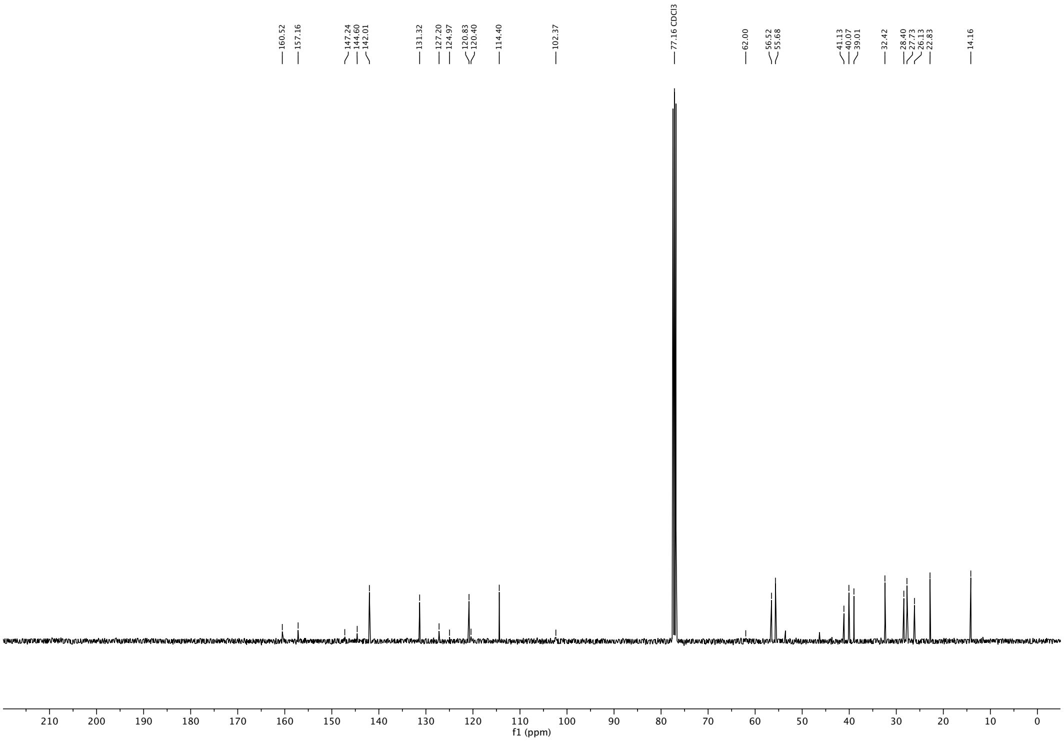


**Figure S35:** $^{13}C\{^1H\}$ NMR Spectra of **BuQn** (101 MHz, $CDCl_3$).

(1*R*)-(6-methoxy-2-phenylquinolin-4-yl)((2*R*,4*S*,5*R*)-5-vinylquinuclidin-2-yl)methanamine (**PhQd**)

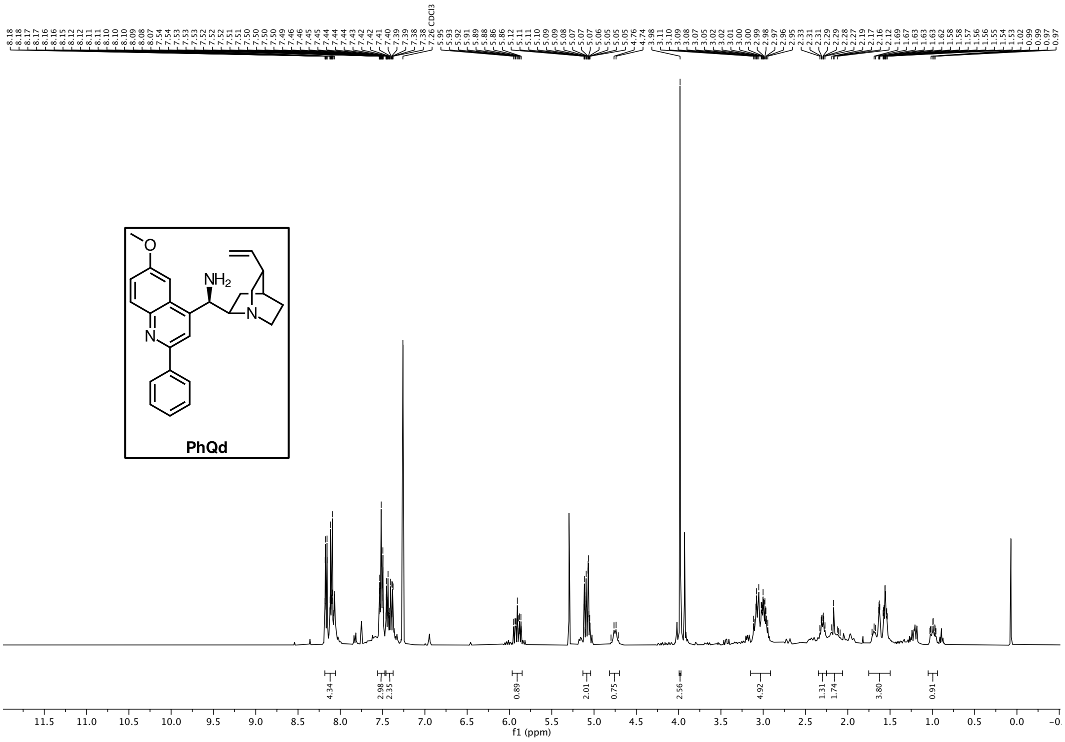


**Figure S36:** $^1H$ NMR Spectra of **PhQd** (400 MHz, $CDCl_3$).

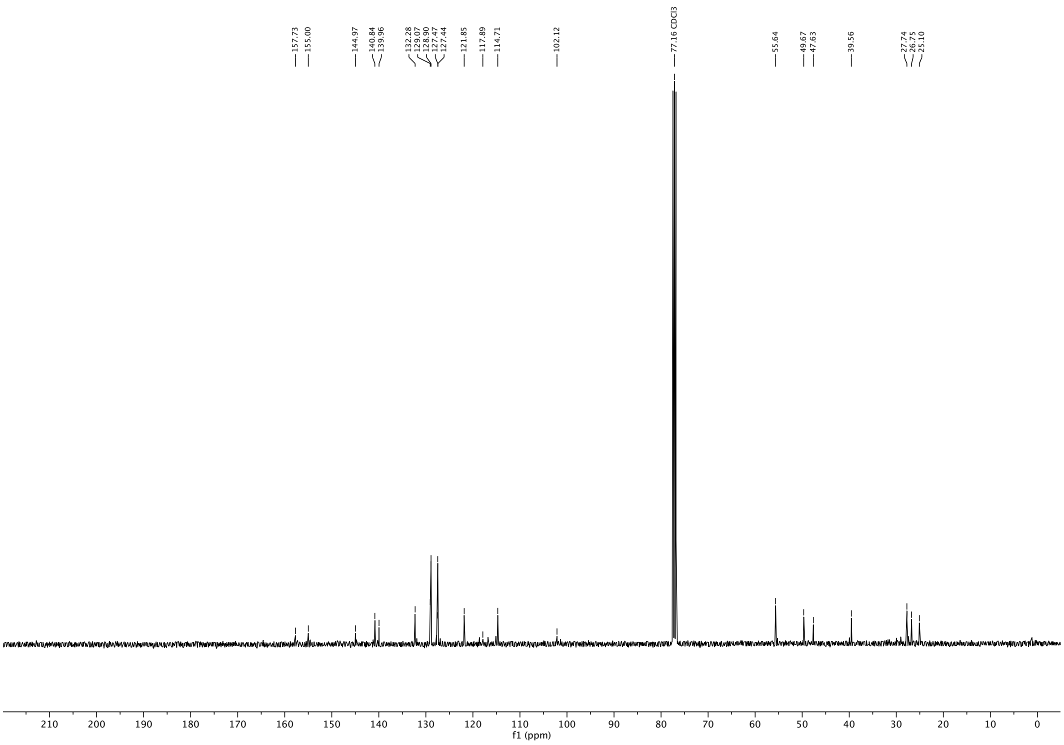

**Figure S37:** $^{13}C\{^1H\}$ NMR Spectra of **PhQd** (101 MHz, $CDCl_3$).

*N*,*N*-diethyl-4-oxopiperidine-1-carboxamide (**7**)

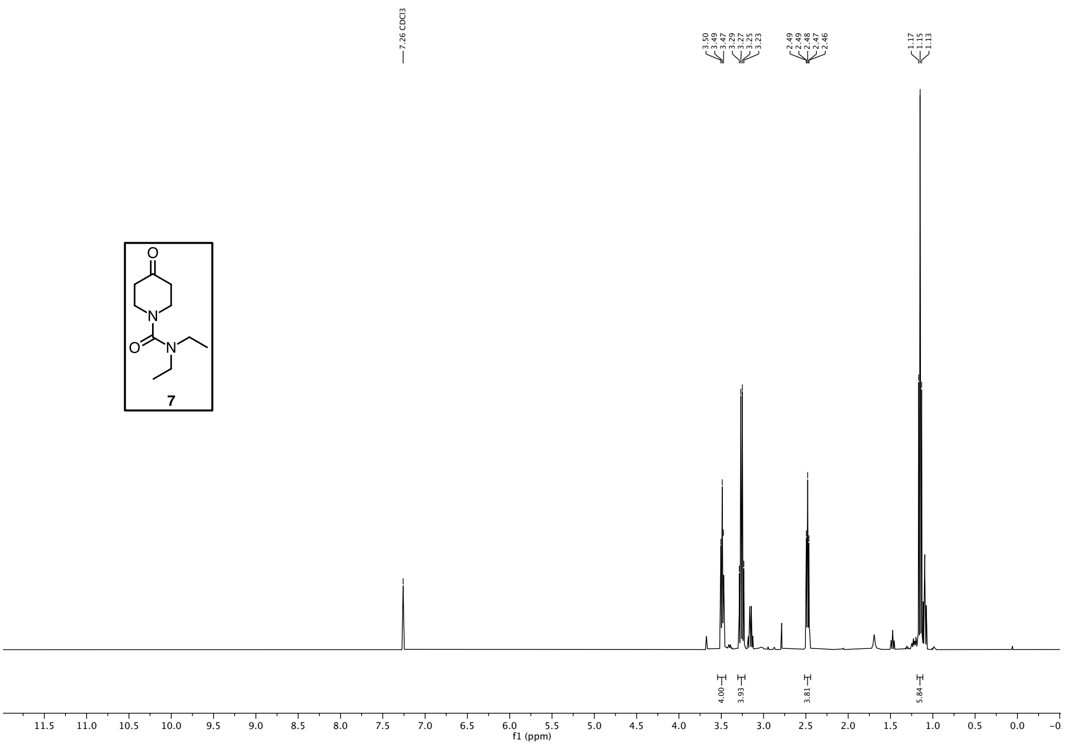

**Figure S38:** $^1H$ NMR Spectra of **7** (400 MHz, $CDCl_3$).

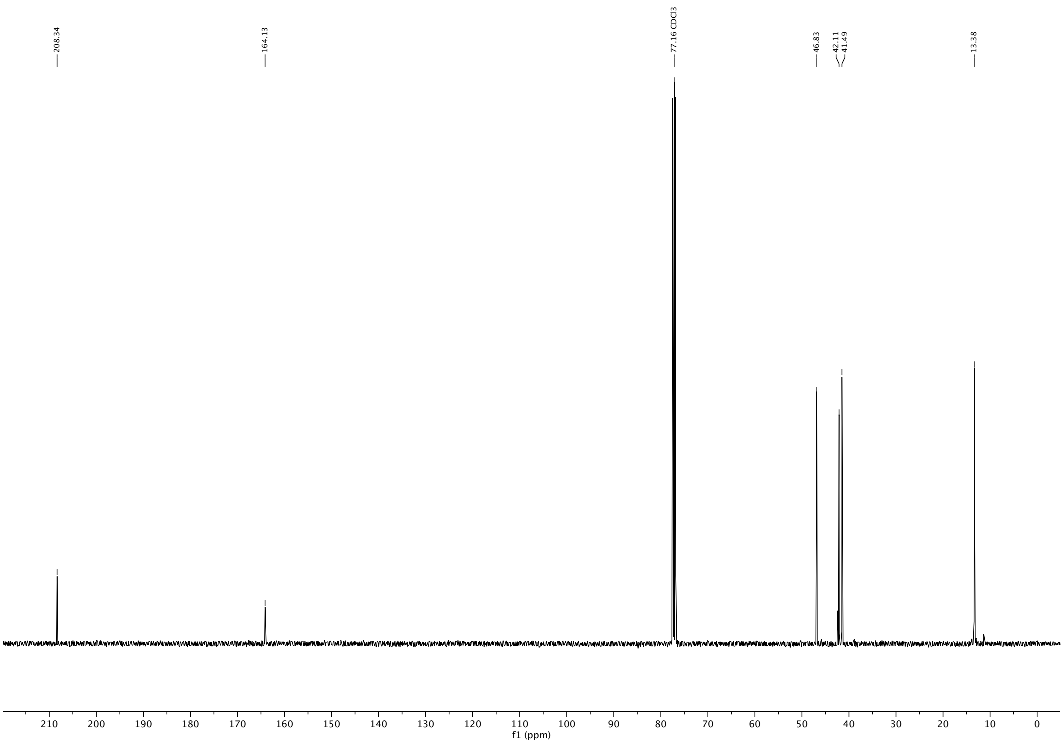

**Figure S39:** $^{13}C\{^1H\}$ NMR Spectra of **7** (101 MHz, $CDCl_3$).

Benzyl 4-oxopiperidine-1-carboxylate (**8**)

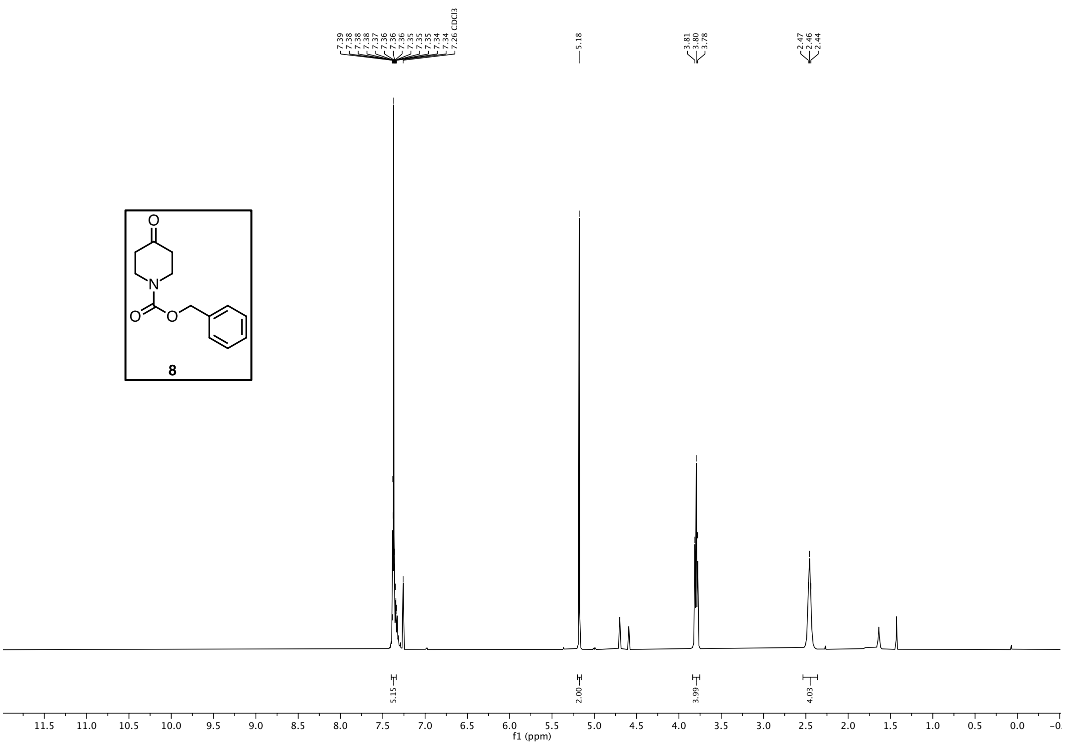

**Figure S40:** $^1H$ NMR Spectra of **8** (400 MHz, $CDCl_3$).

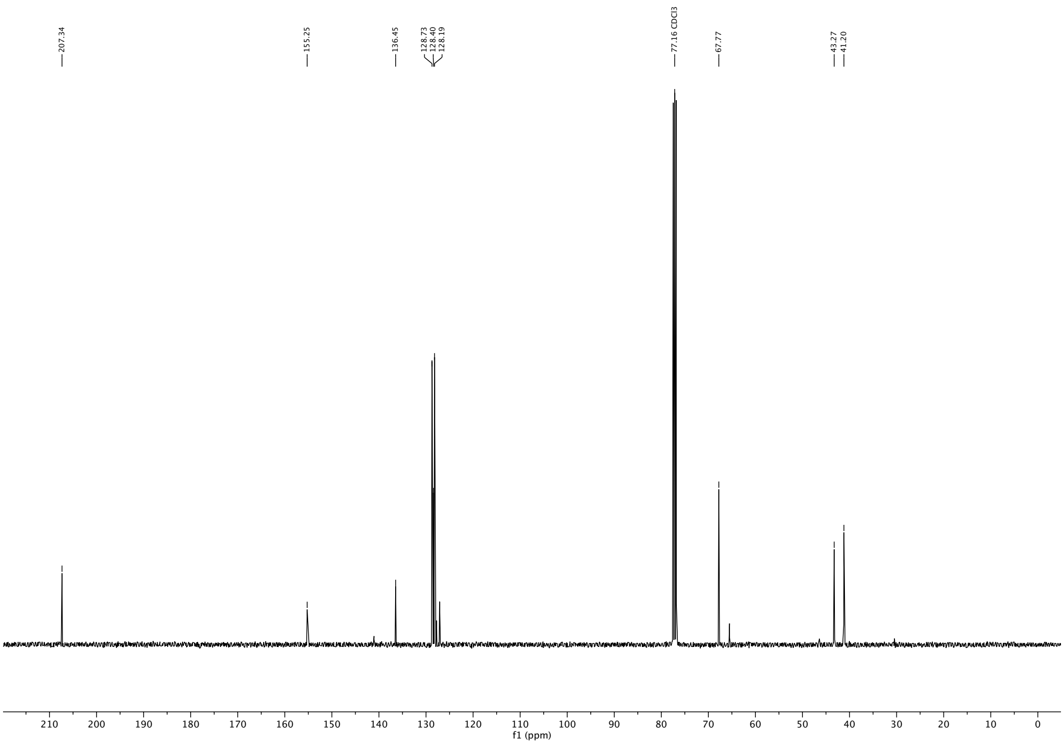


**Figure S41:** $^{13}C\{^{1}H\}$ NMR Spectra of **8** (101 MHz, $CDCl_3$).

## *N*-*tert*-butyloxycarbonylpiperidin-4-one (9)

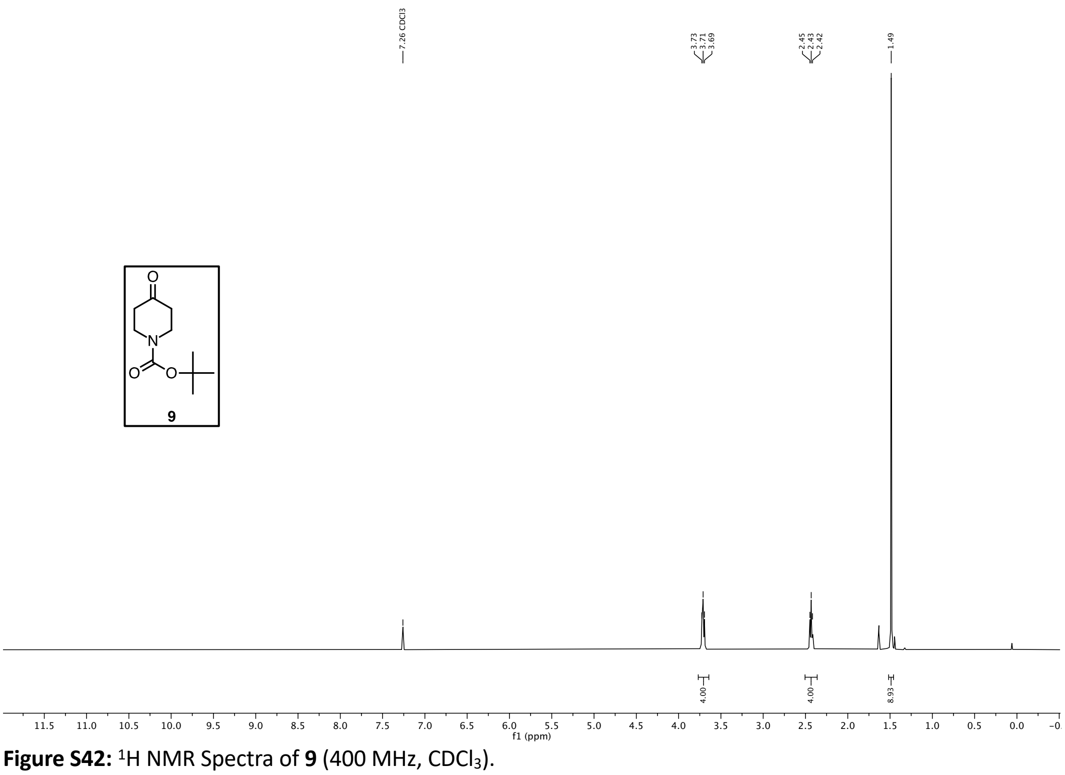


**Figure S42:** $^{1}H$ NMR Spectra of **9** (400 MHz, $CDCl_3$).

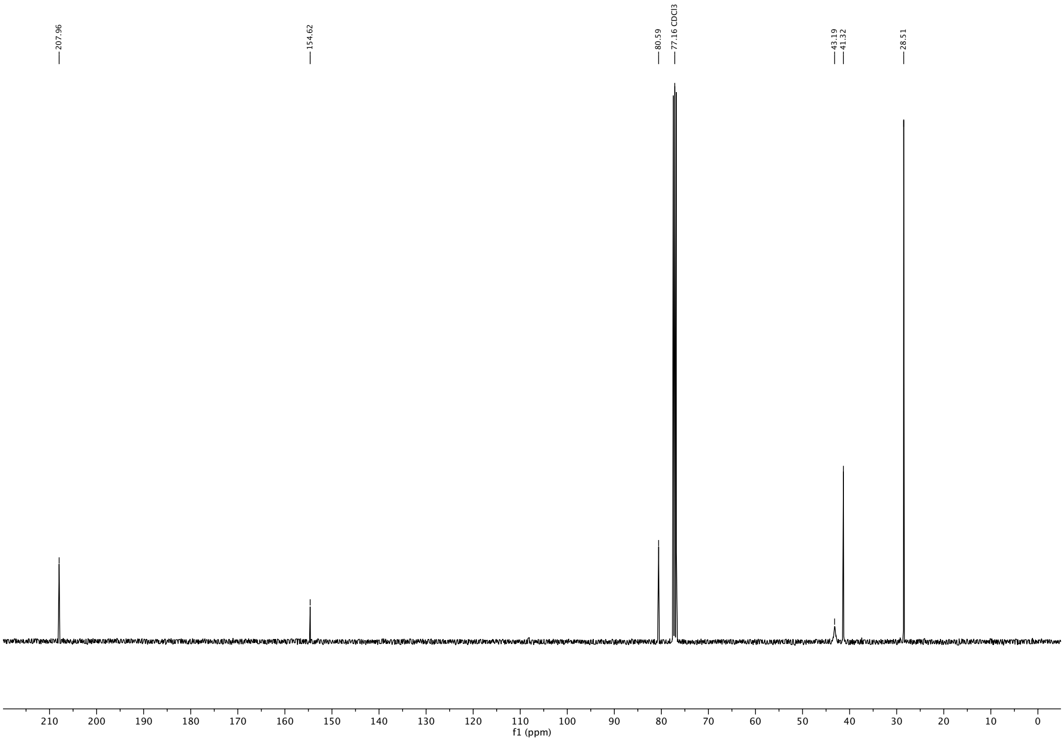


**Figure S43:** $^{13}C\{^1H\}$ NMR Spectra of **9** (101 MHz, $CDCl_3$).

## Dimethyl 2-bromomalonate (**10**)

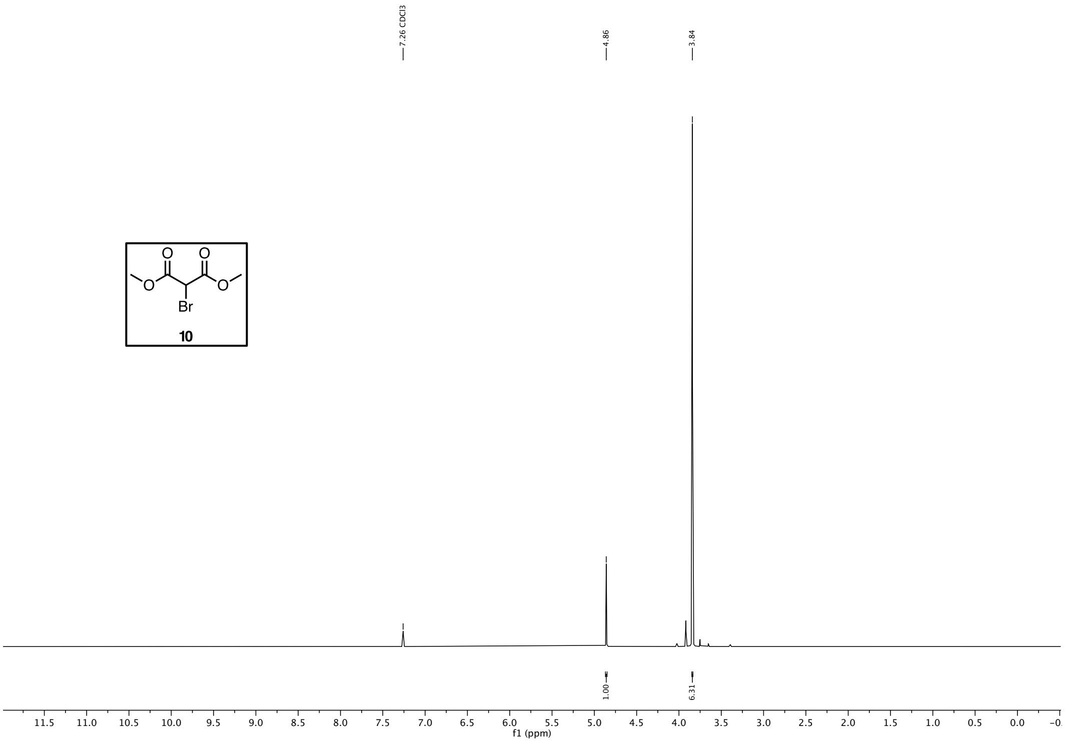


**Figure S44:** $^1H$ NMR Spectra of **10** (400 MHz, $CDCl_3$).

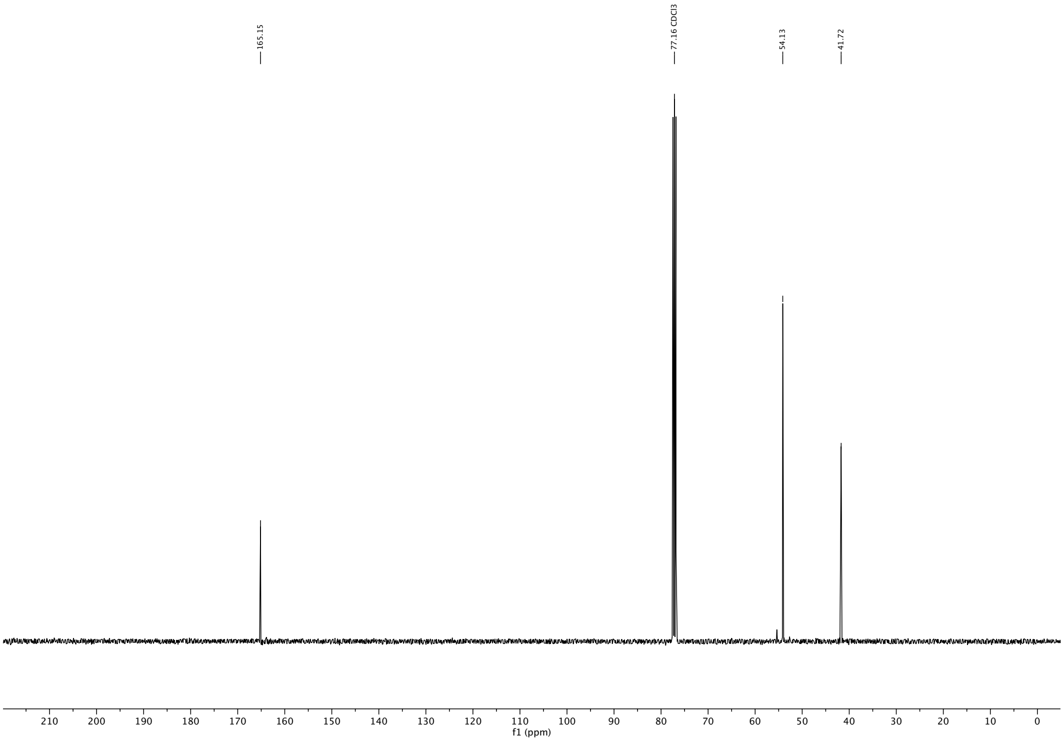


**Figure S45:** $^{13}C\{^{1}H\}$ NMR Spectra of **10** (101 MHz, $CDCl_3$).

## Dibutyl 2-bromomalonate (**11**)

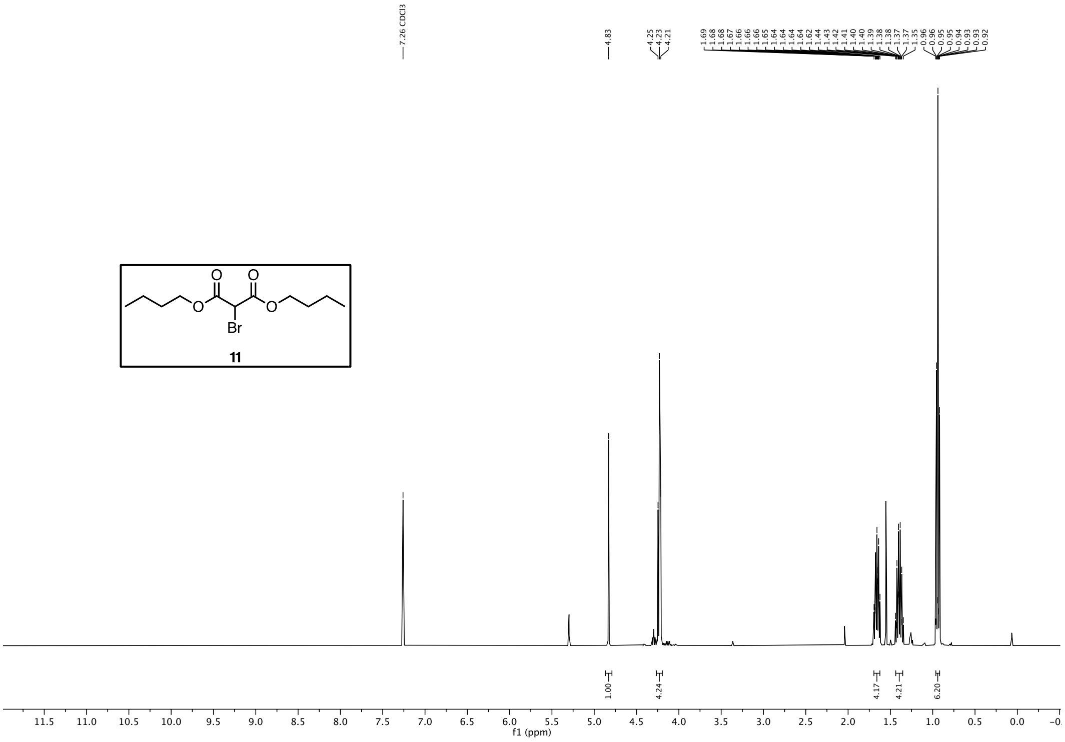


**Figure S46:** $^{1}H$ NMR Spectra of **11** (400 MHz, $CDCl_3$).

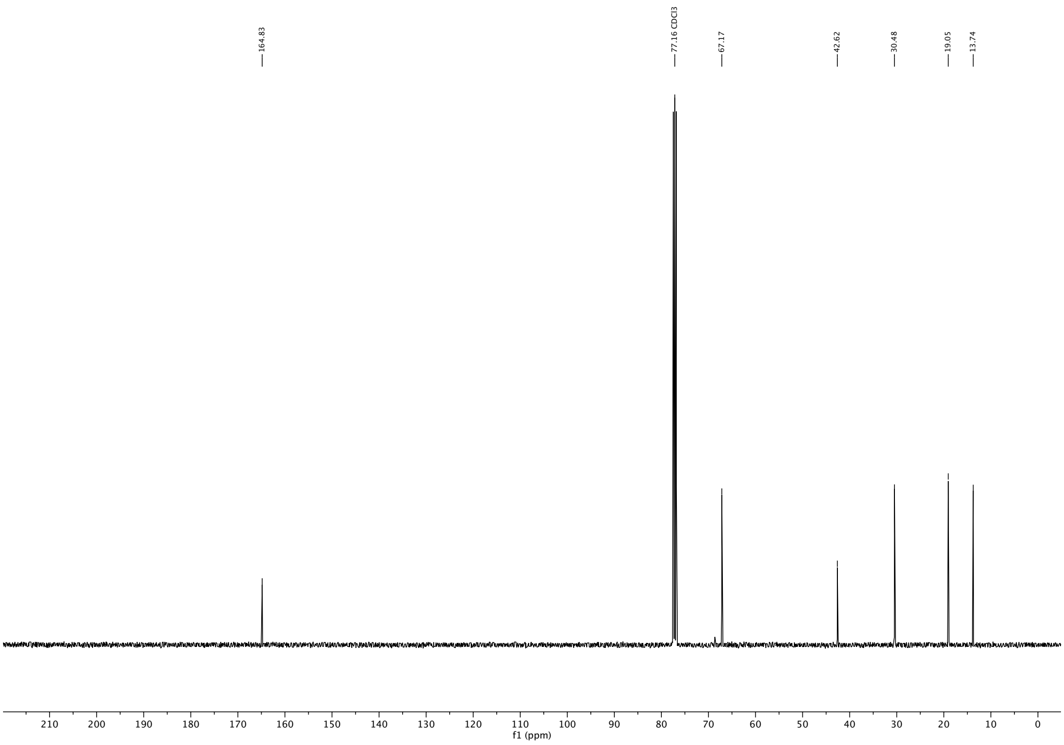


**Figure S47:** $^{13}C\{^{1}H\}$ NMR Spectra of **11** (101 MHz, $CDCl_3$).

## Di-*tert*-butyl 2-bromomalonate (**12**)

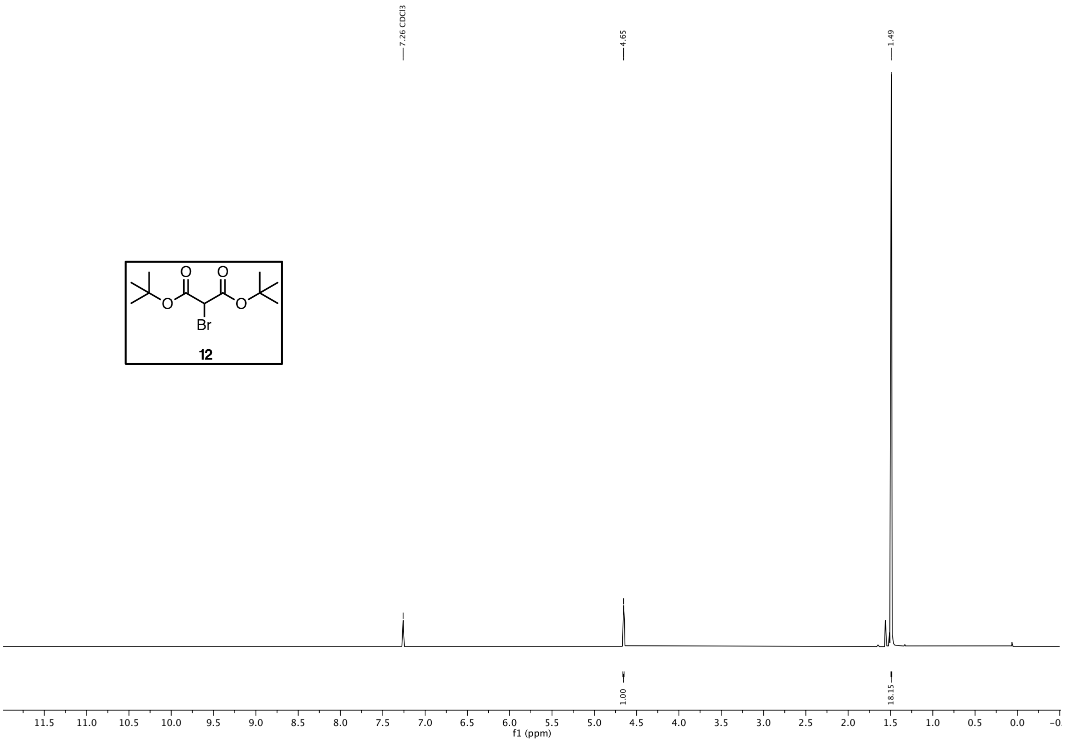


**Figure S48:** $^{1}H$ NMR Spectra of **12** (400 MHz, $CDCl_3$).

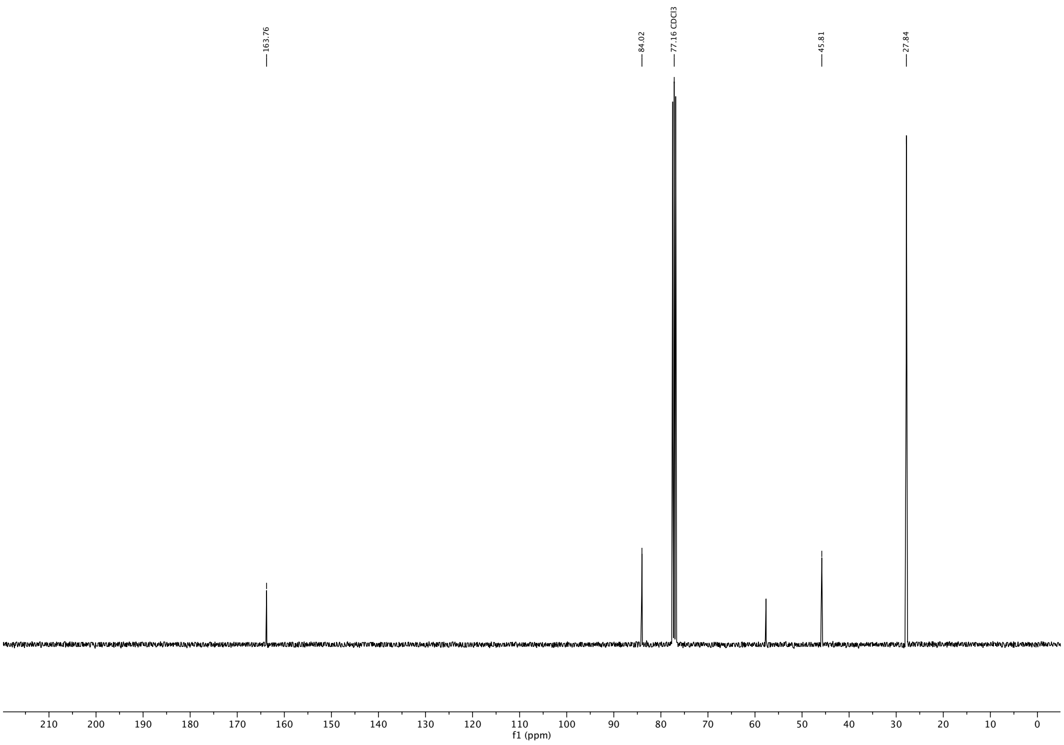


**Figure S49:** $^{13}C\{^{1}H\}$ NMR Spectra of **12** (101 MHz, $CDCl_3$).

## Di-*iso*-propyl 2-bromomalonate (**13**)

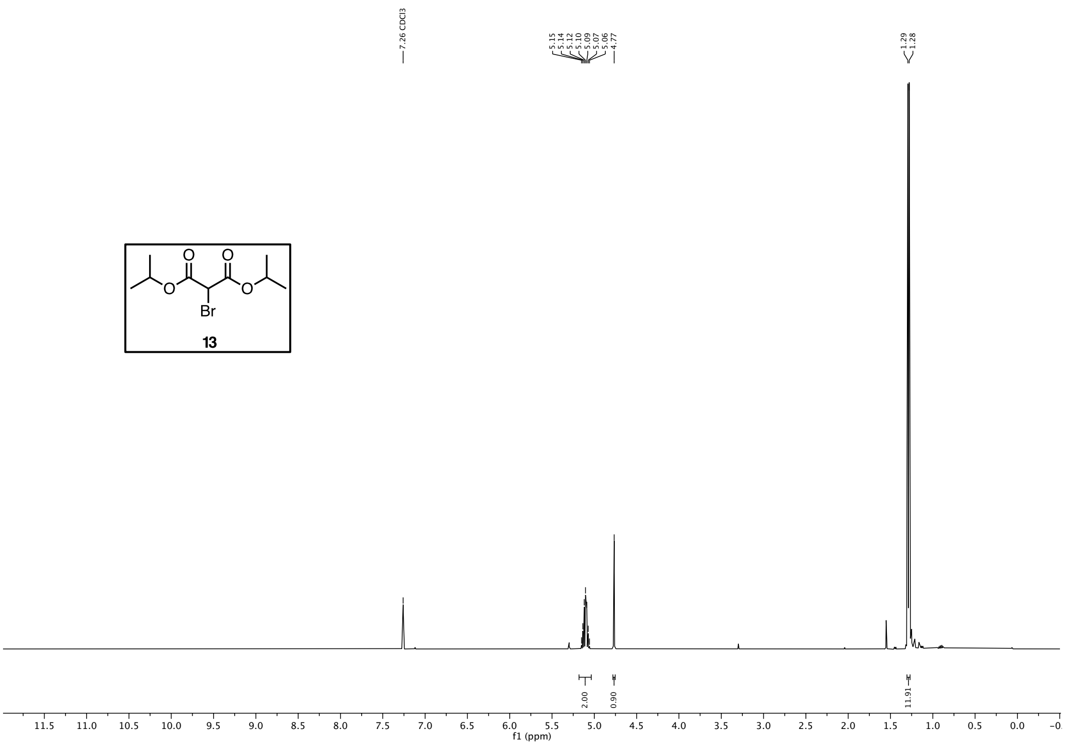


**Figure S50:** $^{1}H$ NMR Spectra of **13** (400 MHz, $CDCl_3$).

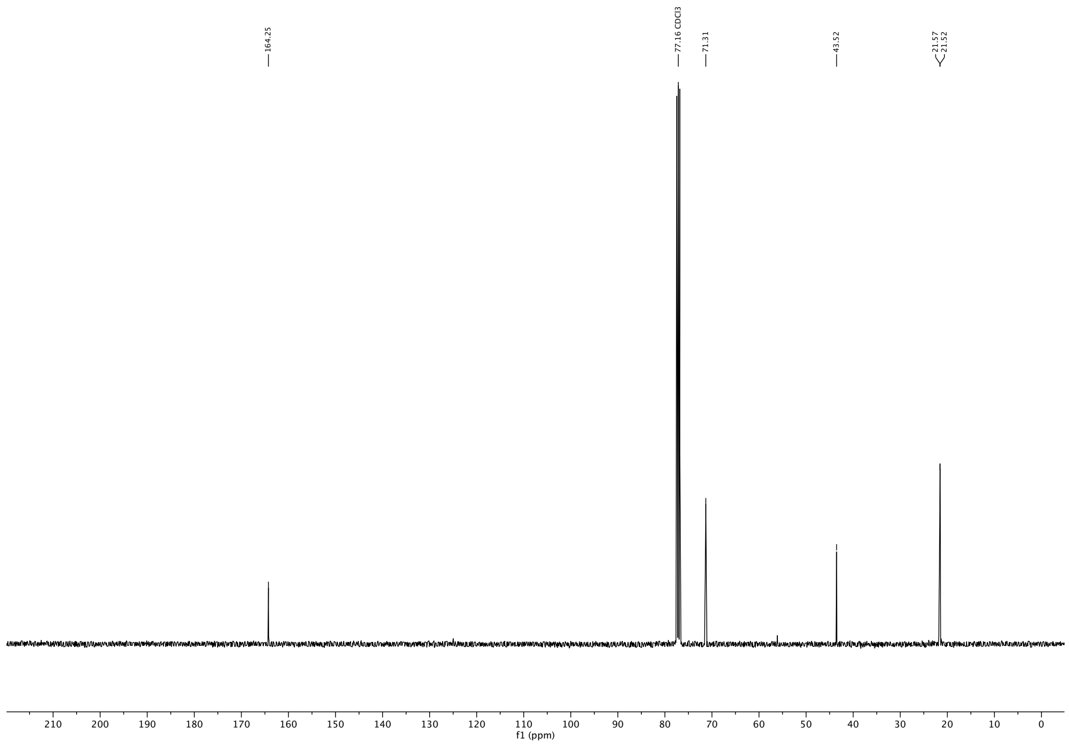

**Figure S51:** $^{13}C\{^{1}H\}$ NMR Spectra of **13** (101 MHz, $CDCl_3$).

Dibenzyl 2-bromomalonate (**14**)

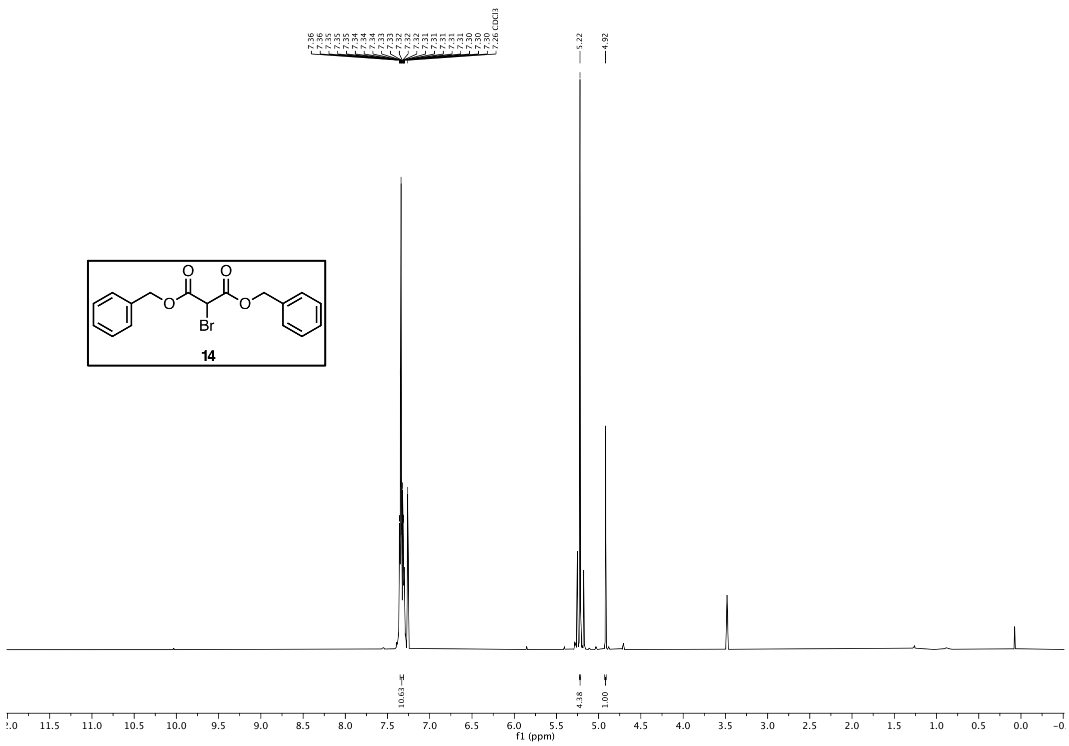

**Figure S52:** $^{1}H$ NMR Spectra of **14** (400 MHz, $CDCl_3$).

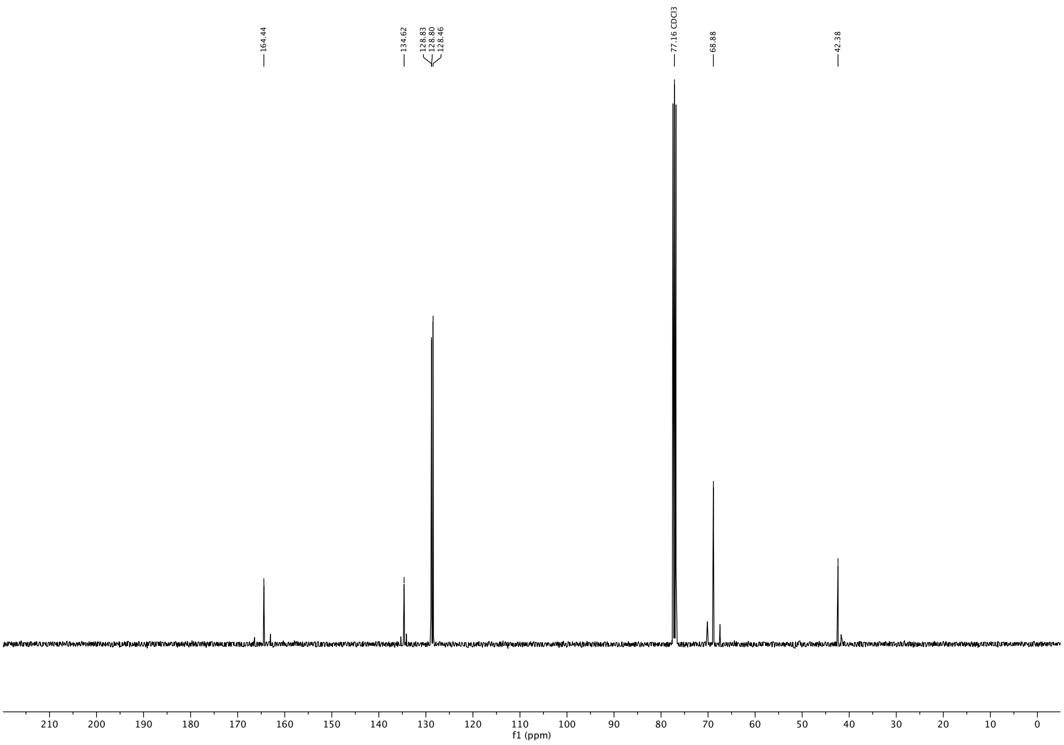


**Figure S53:** $^{13}C\{^{1}H\}$ NMR Spectra of **14** (101 MHz, $CDCl_3$).

## Diethyl 2-(2-oxocyclohexyl)malonate (**P1**)

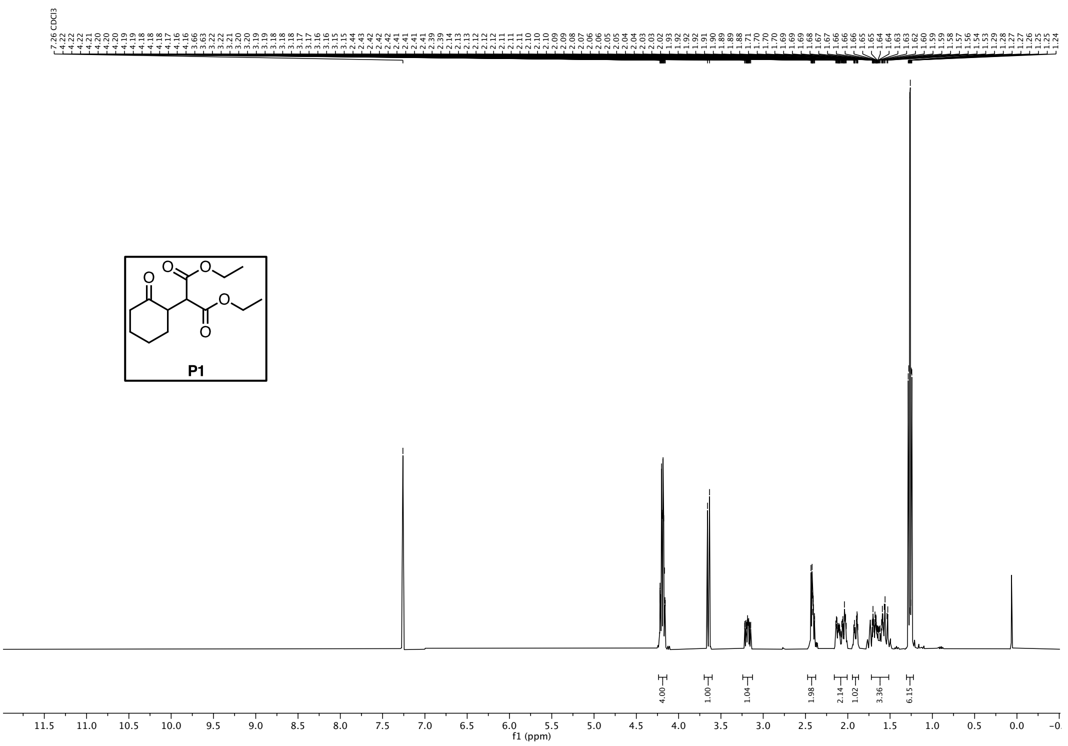


**Figure S54:** $^{1}H$ NMR Spectra of **P1** (400 MHz, $CDCl_3$).

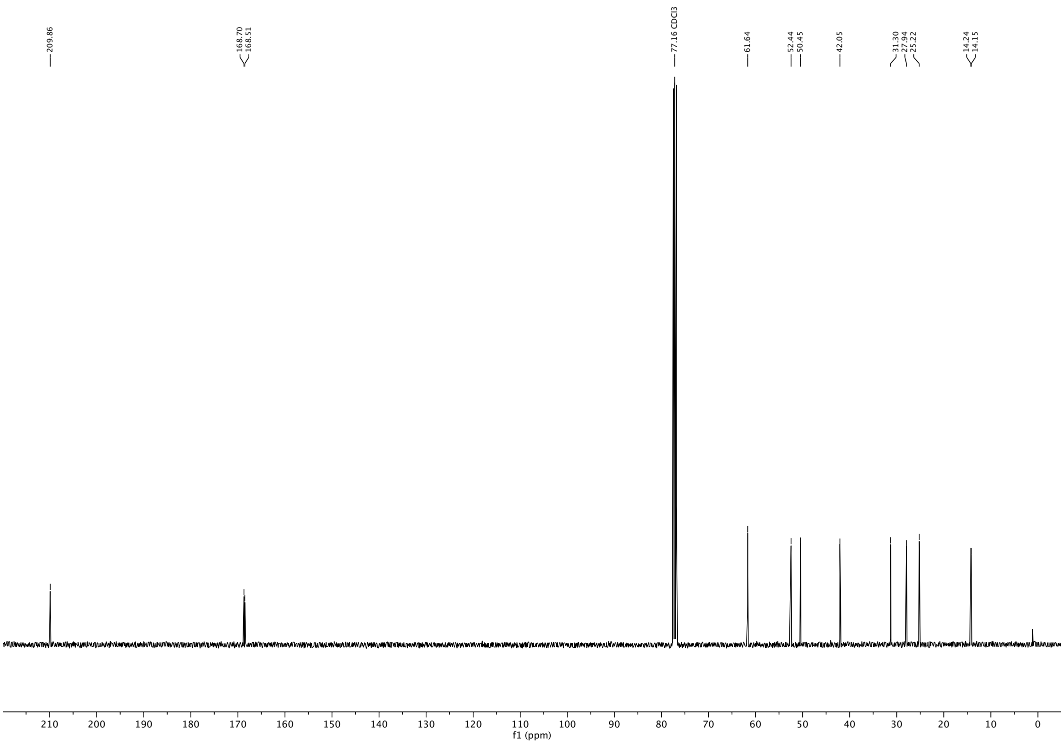


**Figure S55:** $^{13}C\{^{1}H\}$ NMR Spectra of **P1** (101 MHz, $CDCl_3$).

## Diethyl 2-(2-oxocyclopentyl)malonate (**P2**)

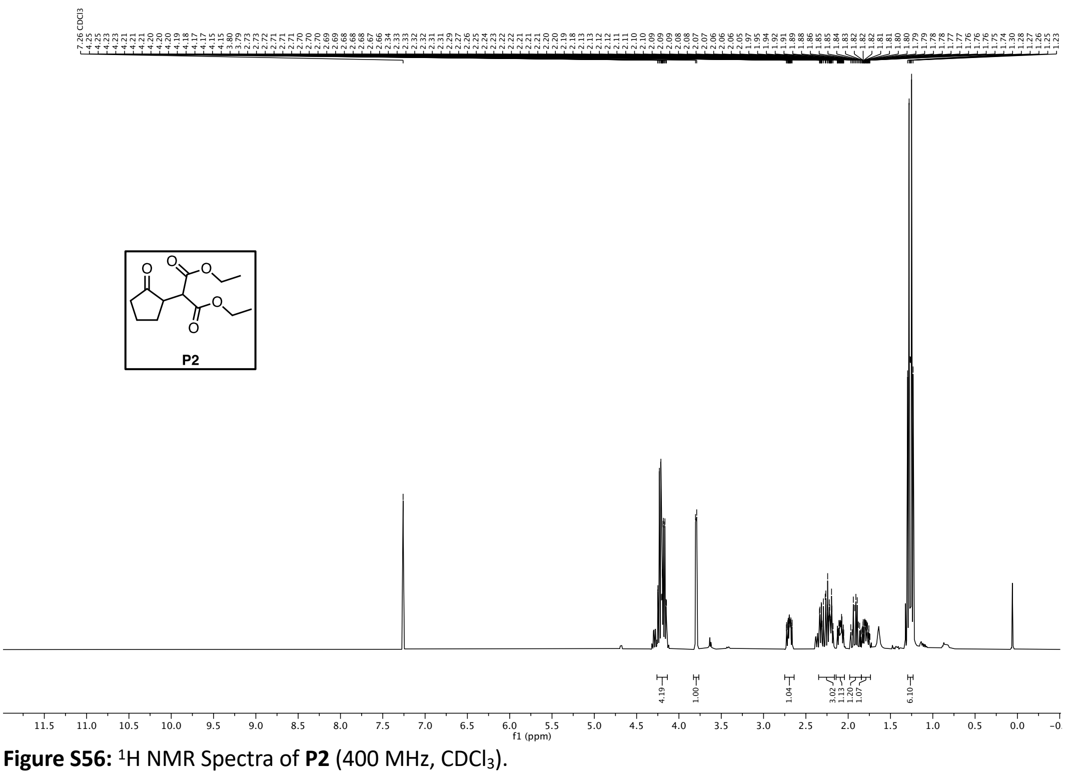


**Figure S56:** $^{1}H$ NMR Spectra of **P2** (400 MHz, $CDCl_3$).

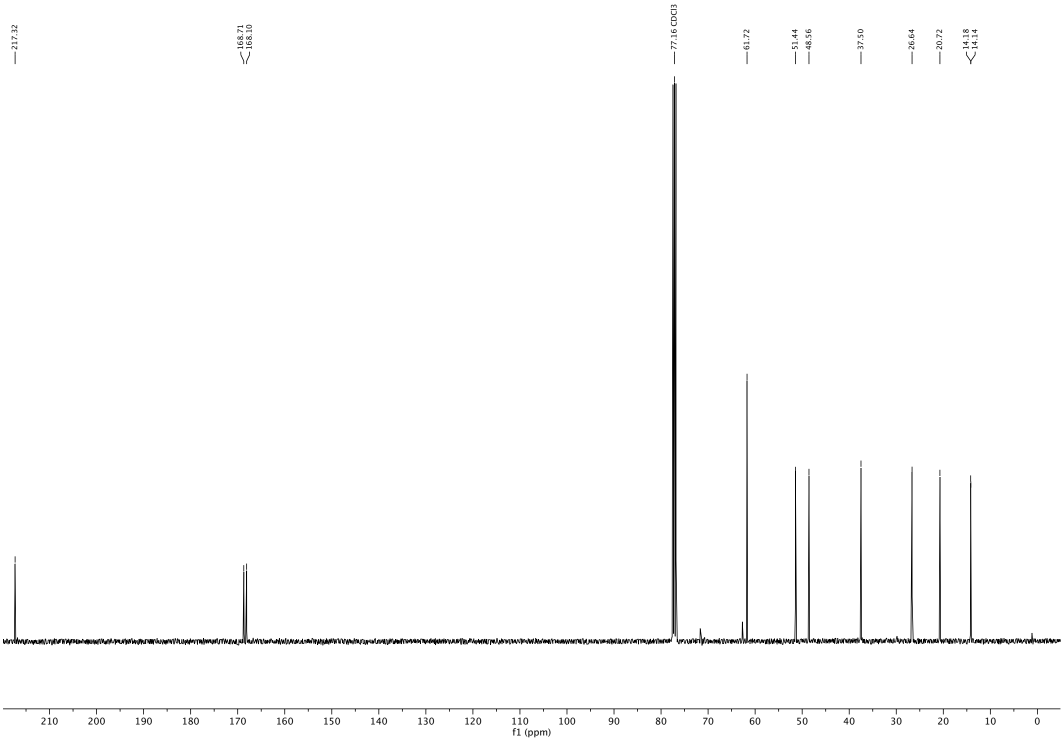


**Figure S57:** $^{13}C\{^{1}H\}$ NMR Spectra of **P2** (101 MHz, $CDCl_3$).

## Diethyl 2-(2-oxocycloheptyl)malonate (**P3**)

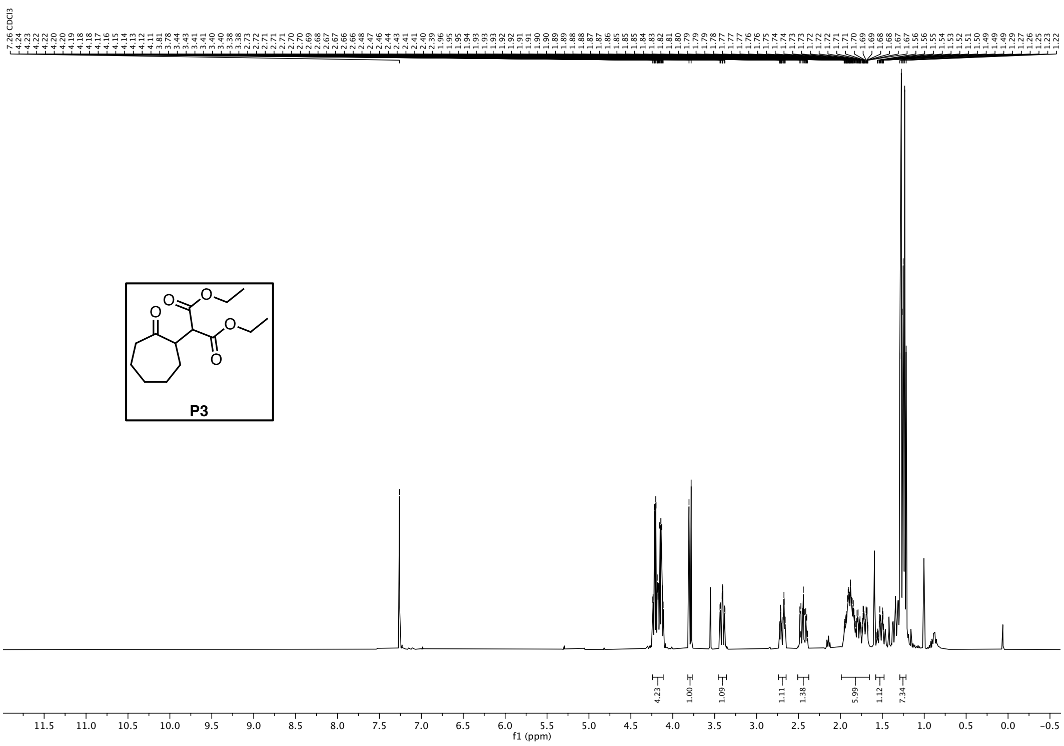


**Figure S58:** $^{1}H$ NMR Spectra of **P3** (400 MHz, $CDCl_3$).

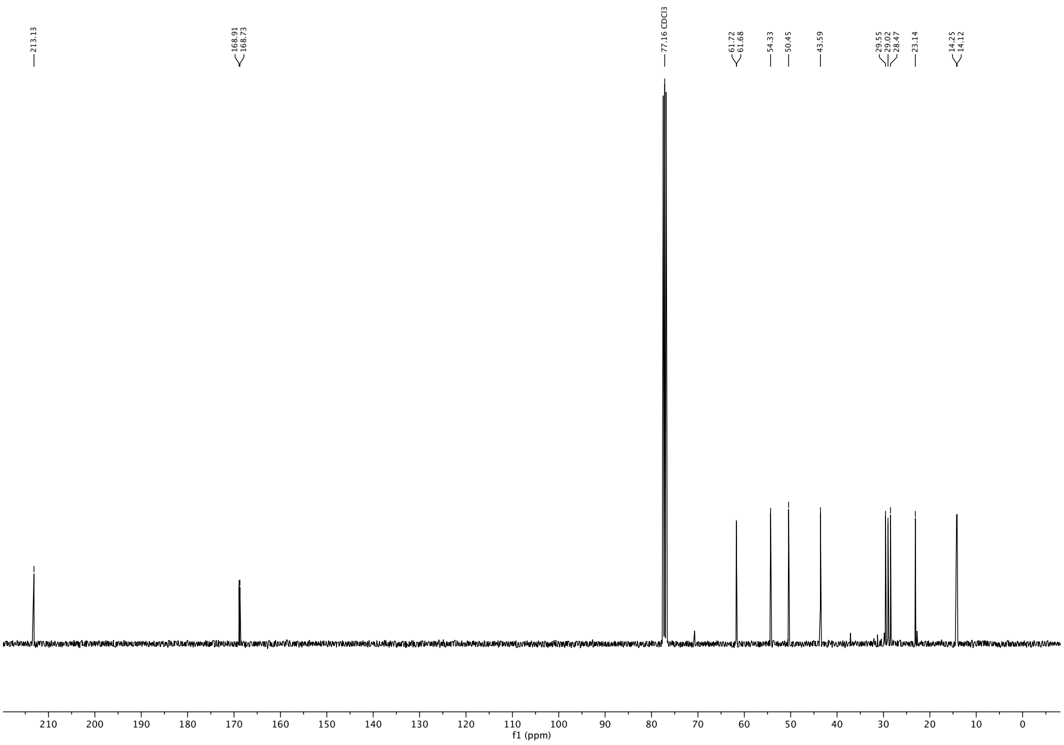


**Figure S59:** $^{13}C\{^{1}H\}$ NMR Spectra of **P3** (101 MHz, $CDCl_3$).

Diethyl 2-(5,5-dimethyl-2-oxocyclohexyl)malonate (**P4**)

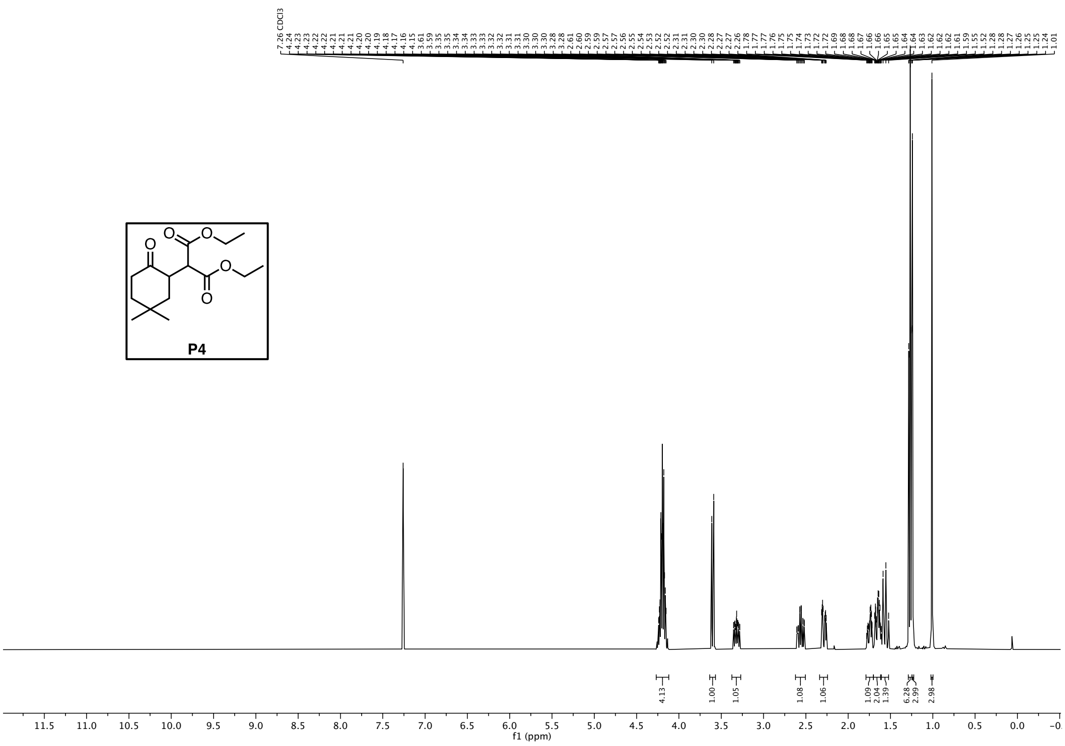


**Figure S60:** $^{1}H$ NMR Spectra of **P4** (400 MHz, $CDCl_3$).

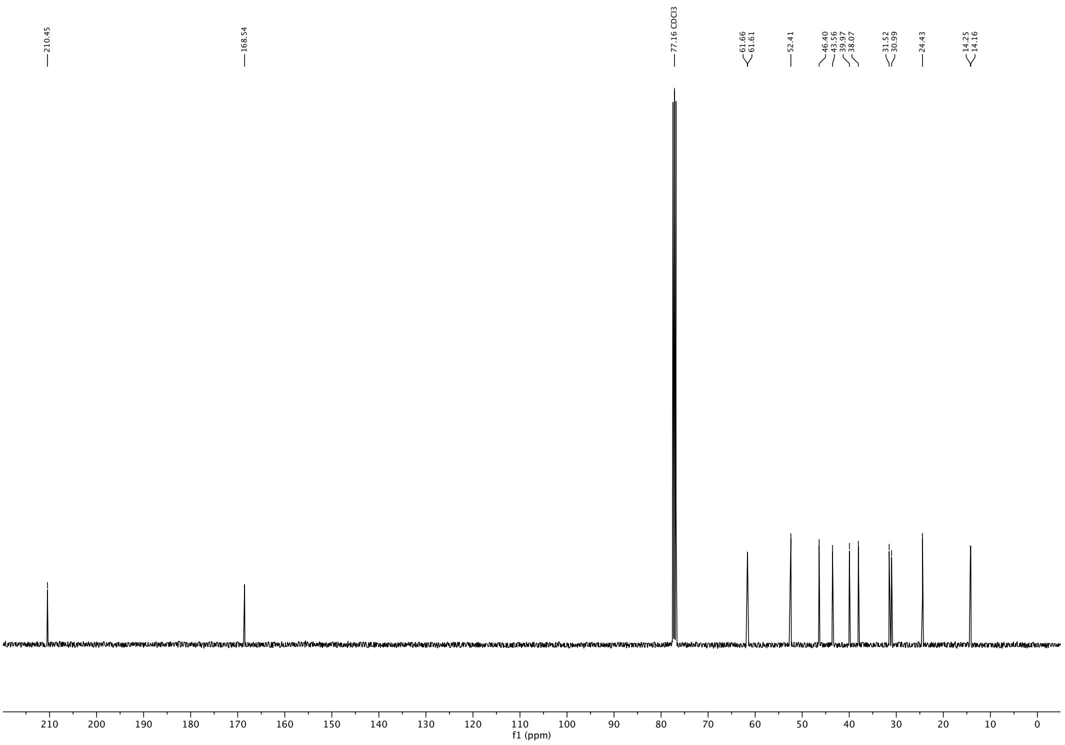


**Figure S61:** $^{13}C\{^{1}H\}$ NMR Spectra of **P4** (101 MHz, $CDCl_3$).

Diethyl 2-(4-oxotetrahydro-2*H*-pyran-3-yl)malonate (**P5**)

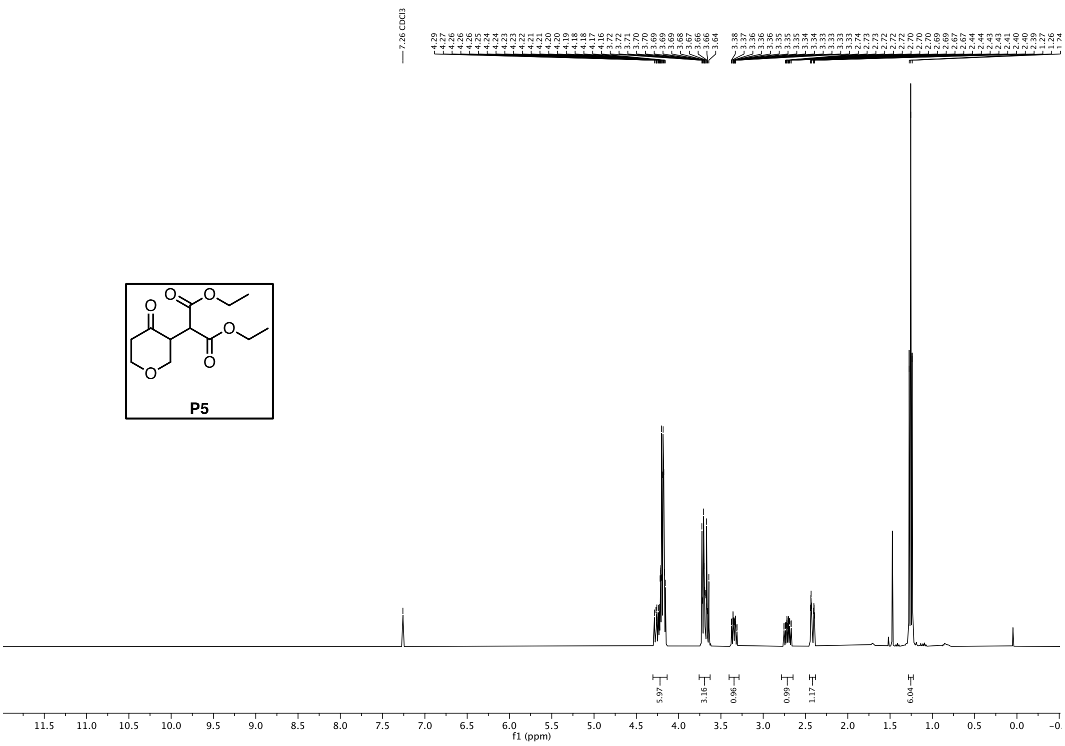


**Figure S62:** $^{1}H$ NMR Spectra of **P5** (400 MHz, $CDCl_3$).

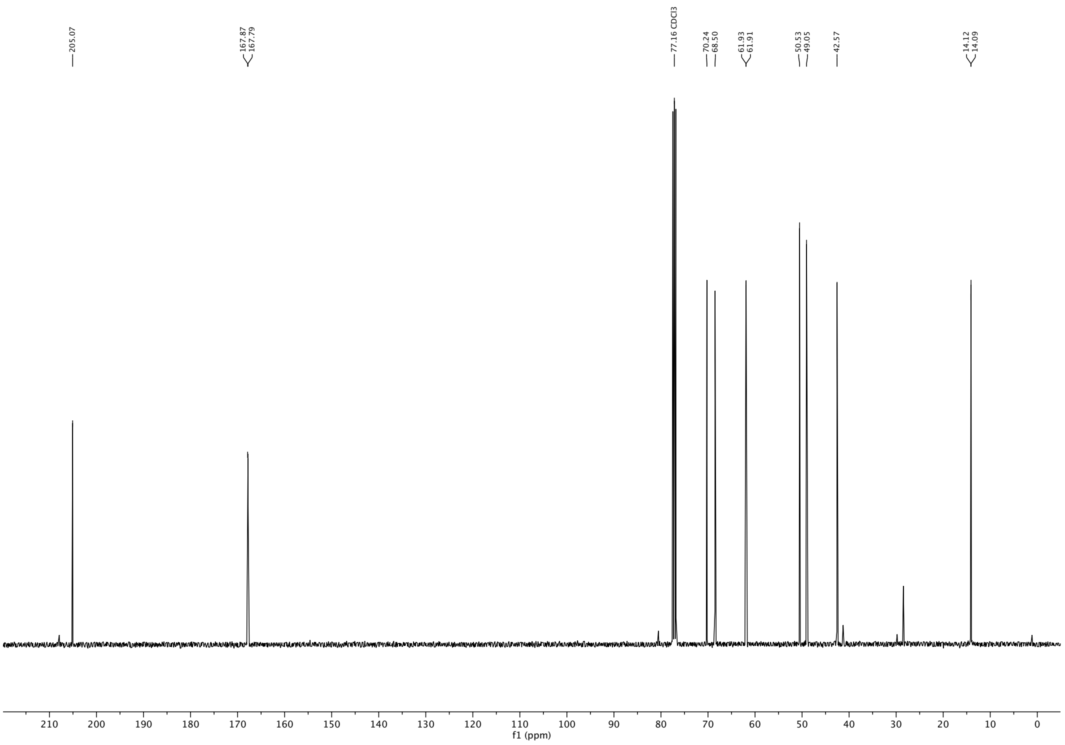


**Figure S63:** $^{13}C\{^{1}H\}$ NMR Spectra of **P5** (101 MHz, $CDCl_3$).

Diethyl 2-(4-oxotetrahydro-2*H*-thiopyran-3-yl)malonate (**P6**)

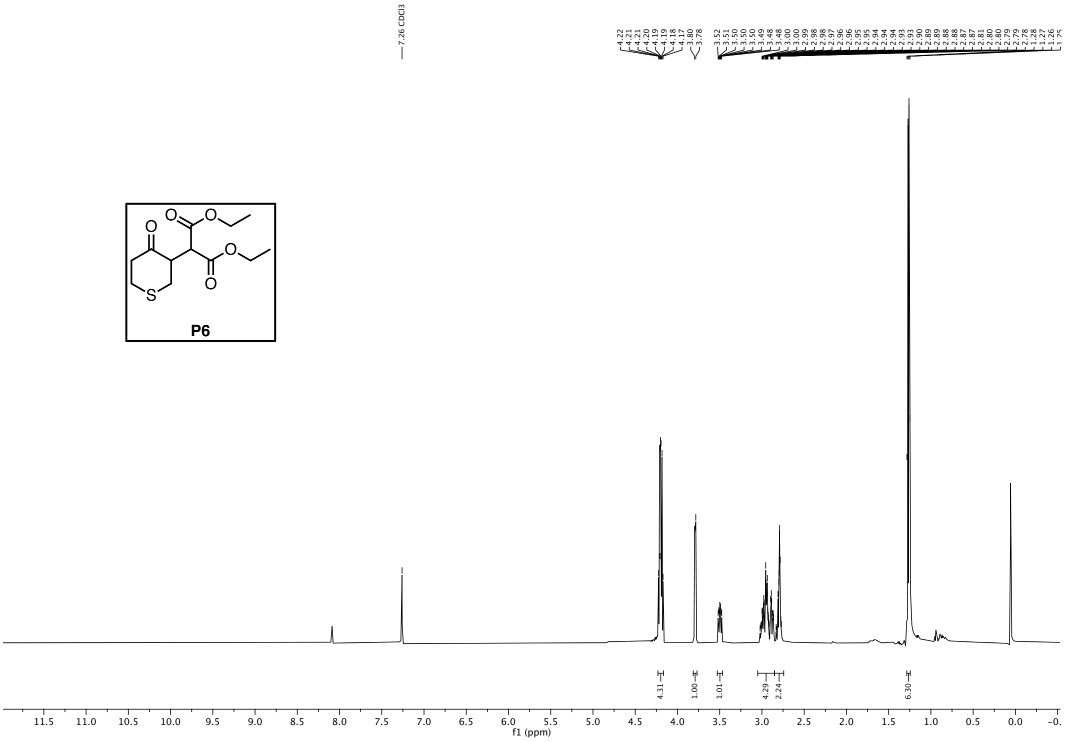


**Figure S64:** $^{1}H$ NMR Spectra of **P6** (400 MHz, $CDCl_3$).

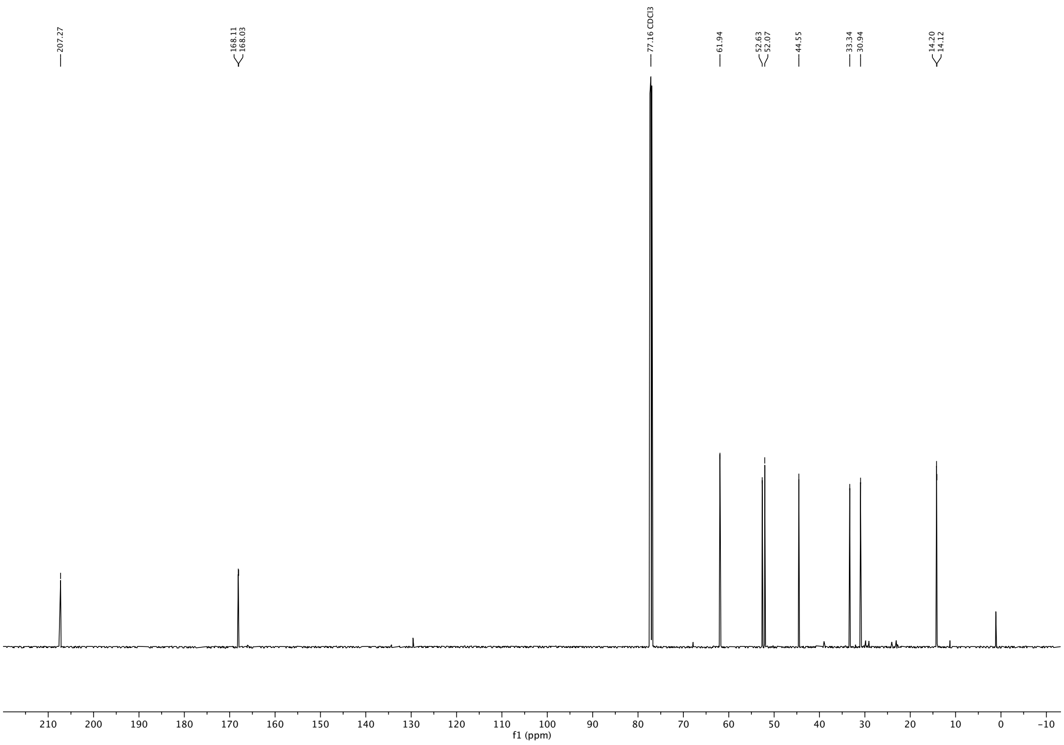

**Figure S65:** $^{13}C\{^1H\}$ NMR Spectra of **P6** (101 MHz, $CDCl_3$).

Diethyl 2-(5,5-difluoro-2-oxocyclohexyl)malonate (**P7**)

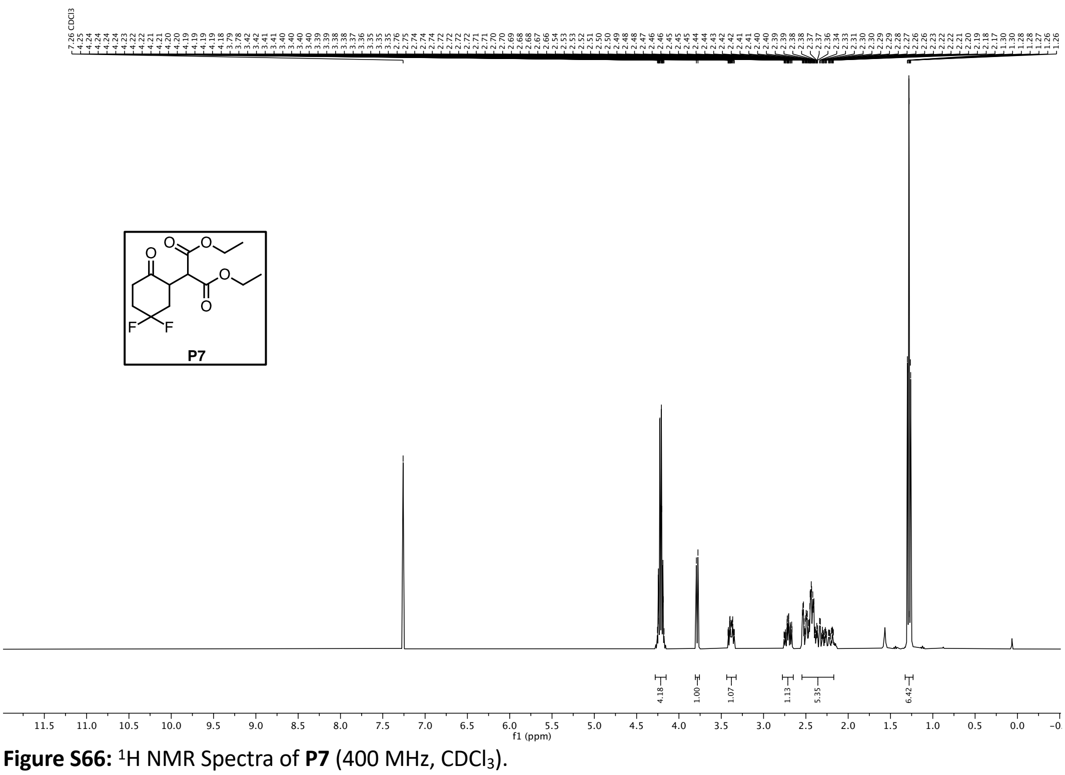

**Figure S66:** $^1H$ NMR Spectra of **P7** (400 MHz, $CDCl_3$).

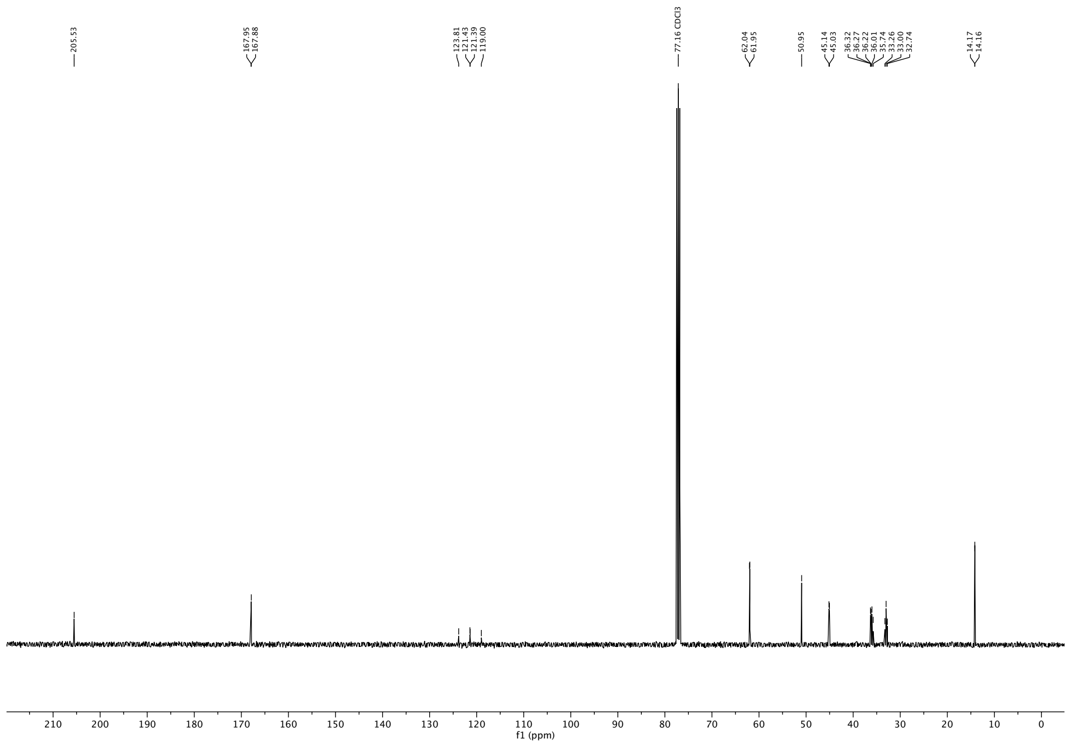


**Figure S67:** $^{13}C\{^{1}H\}$ NMR Spectra of **P7** (101 MHz, $CDCl_3$).

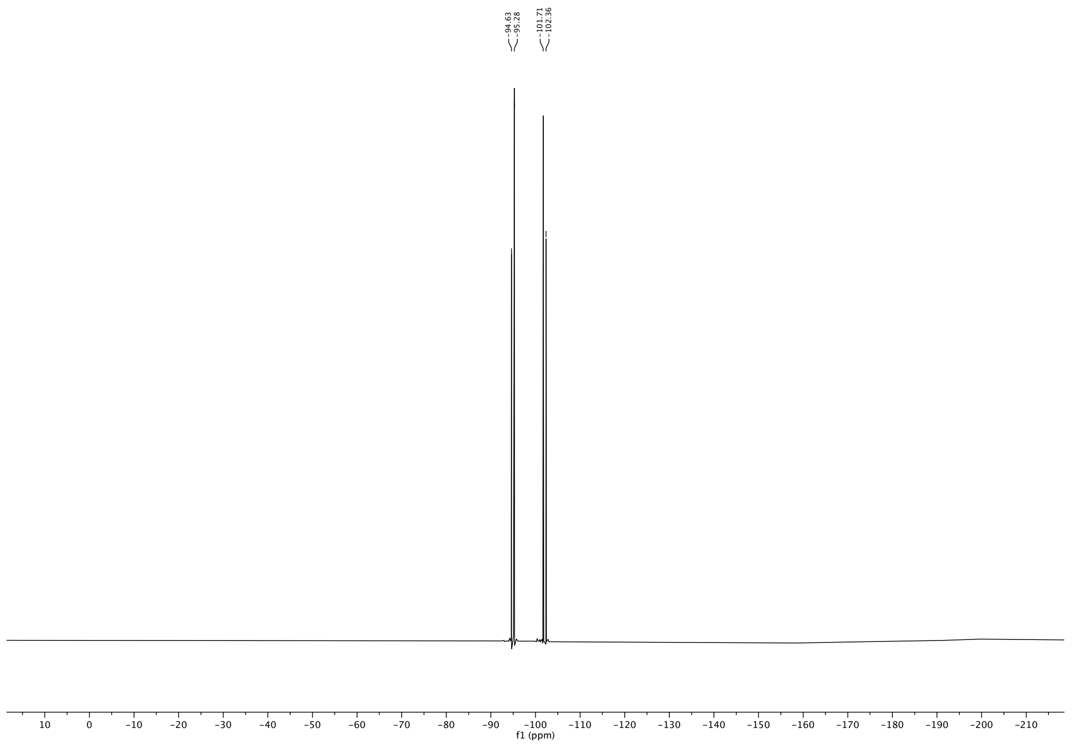


**Figure S68:** $^{19}F\{^{1}H\}$ Spectra of **P7** (376 MHz, $CDCl_3$).

Diethyl 2-(5,5-bis-ethoxycarbonyl-2-oxocyclohexyl)malonate (**P8**)

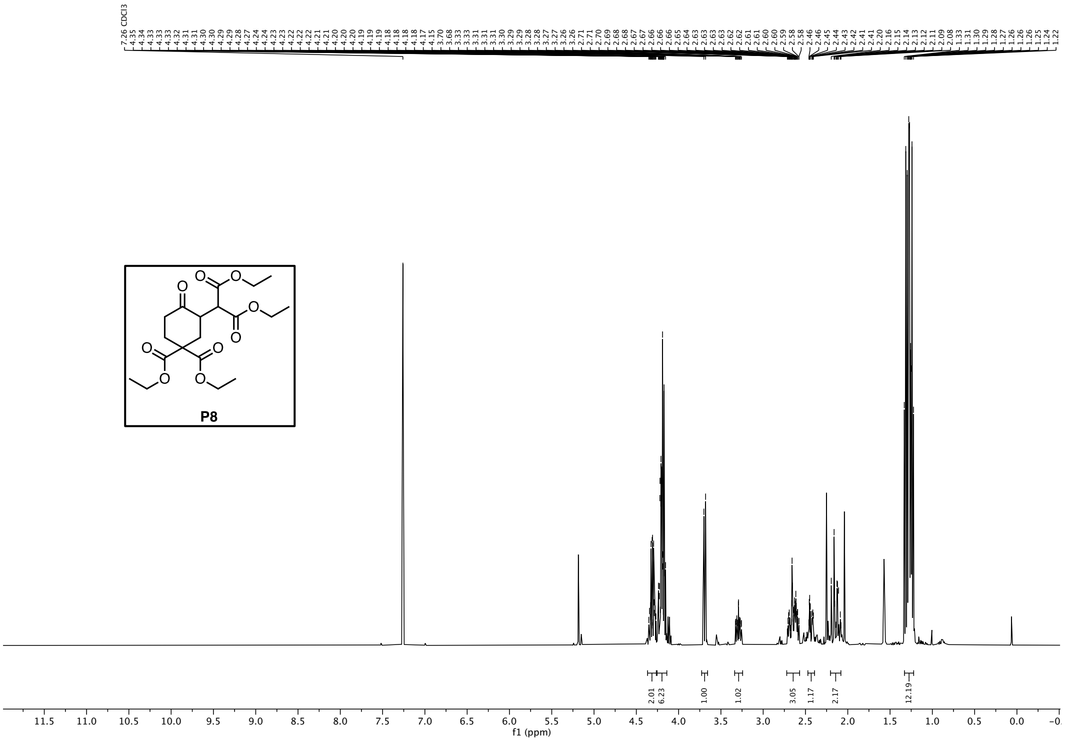


**Figure S69:** $^{1}H$ NMR Spectra of **P8** (400 MHz, $CDCl_3$).

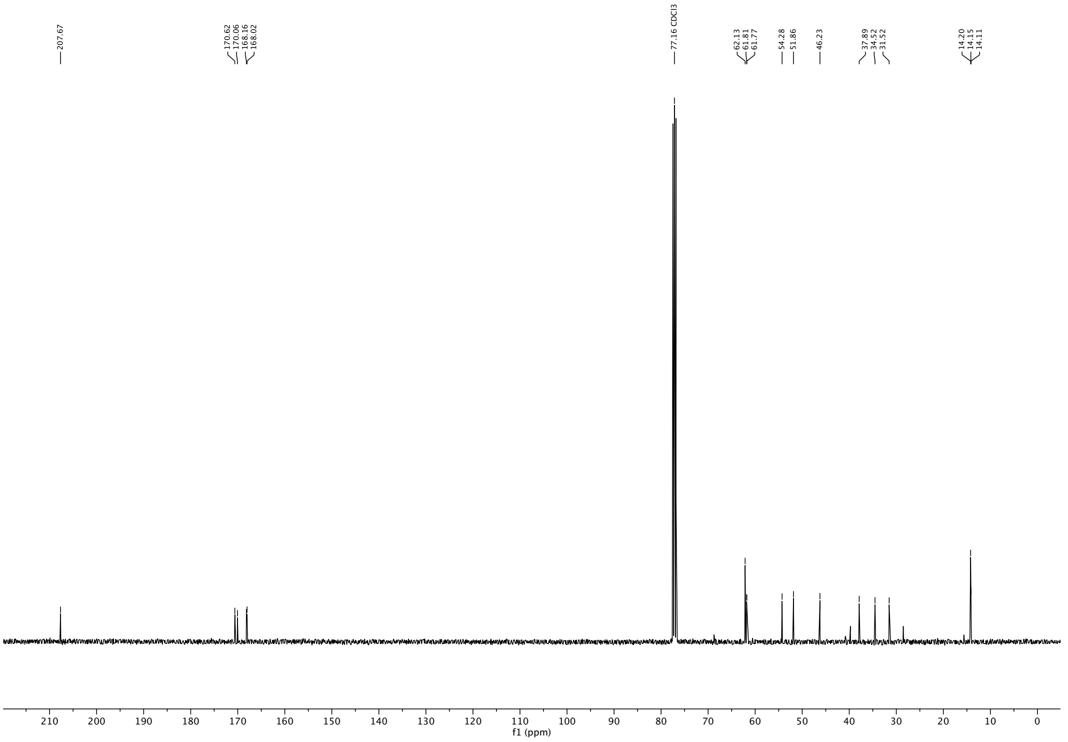


**Figure S70:** $^{13}C\{^{1}H\}$ NMR Spectra of **P8** (101 MHz, $CDCl_3$).

Diethyl 2-[1-(diethylcarbamoyl)-4-oxopiperidin-3-yl]malonate (**P9**)

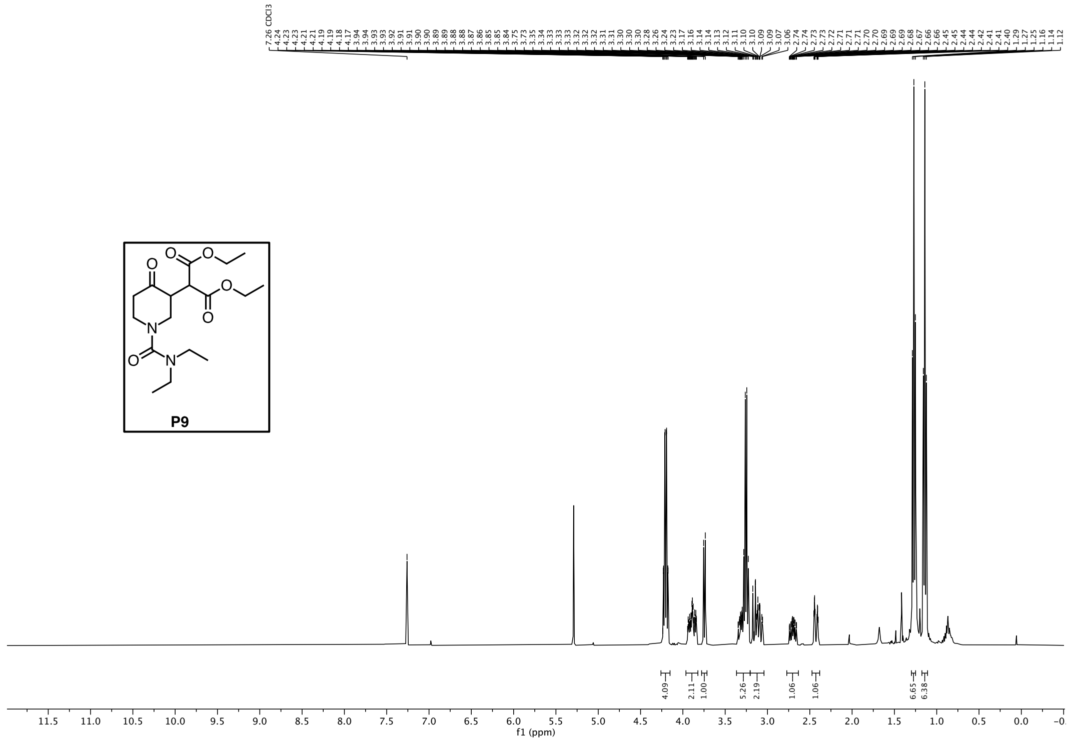


**Figure S71:** $^{1}H$ NMR Spectra of **P9** (400 MHz, $CDCl_3$).

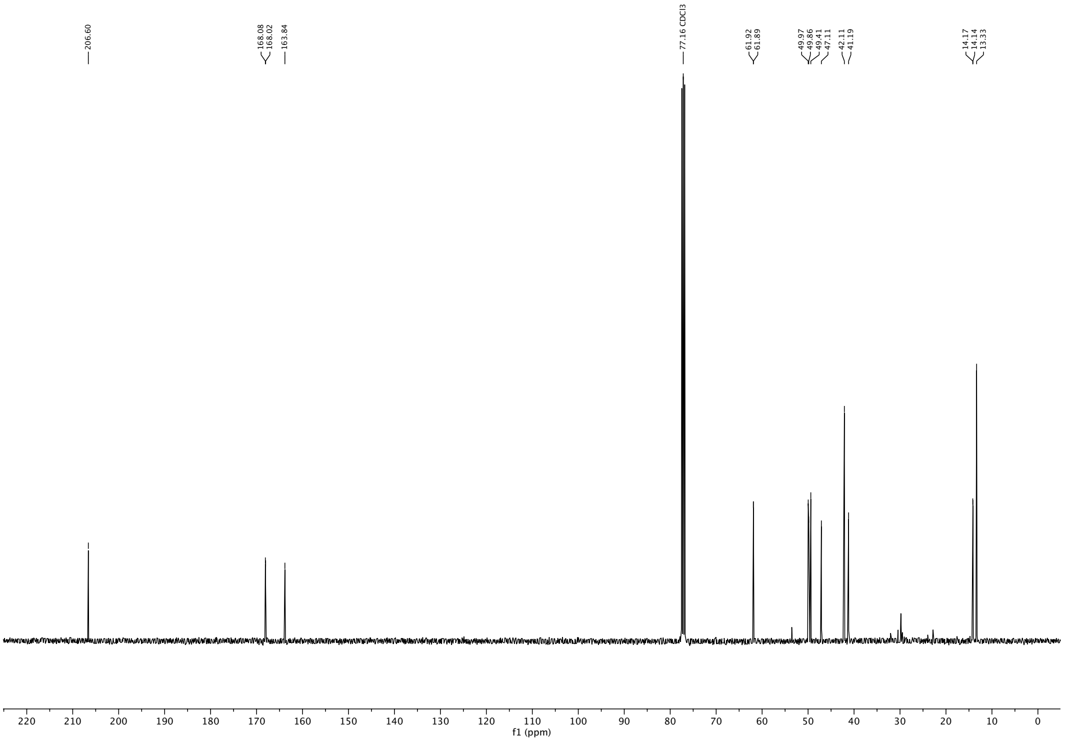


**Figure S72:** $^{13}C\{^{1}H\}$ NMR Spectra of **P9** (101 MHz, $CDCl_3$).

Diethyl 2-(1-benzoyl-4-oxopiperidin-3-yl)malonate (**P10**)

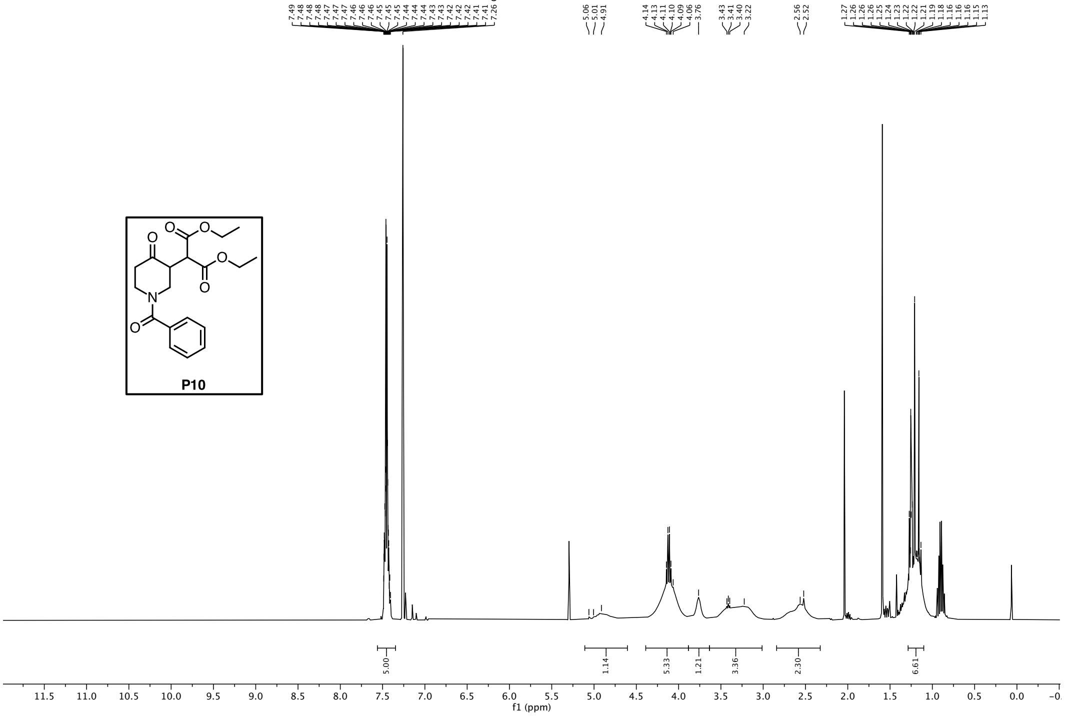


**Figure S73:** $^{1}H$ NMR Spectra of **P10** (400 MHz, $CDCl_3$).

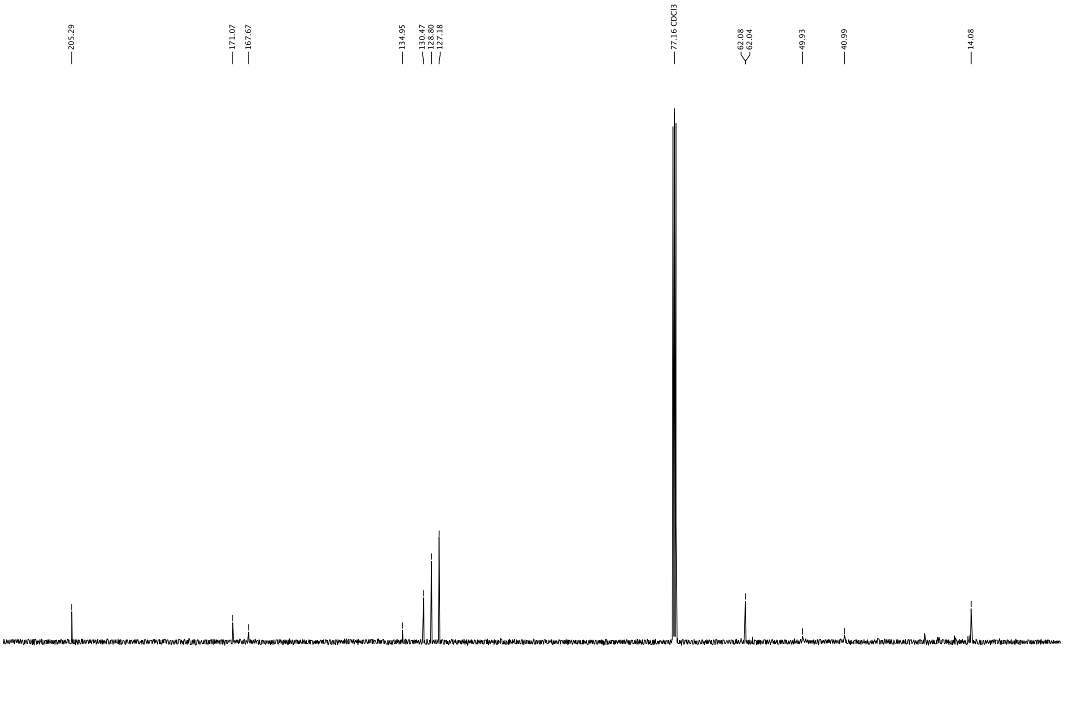


**Figure S74:** $^{13}C\{^{1}H\}$ NMR Spectra of **P10** (101 MHz, $CDCl_3$).

Diethyl 2-(1-phenoxycarbonyl-4-oxopiperidin-3-yl)malonate (**P11**)

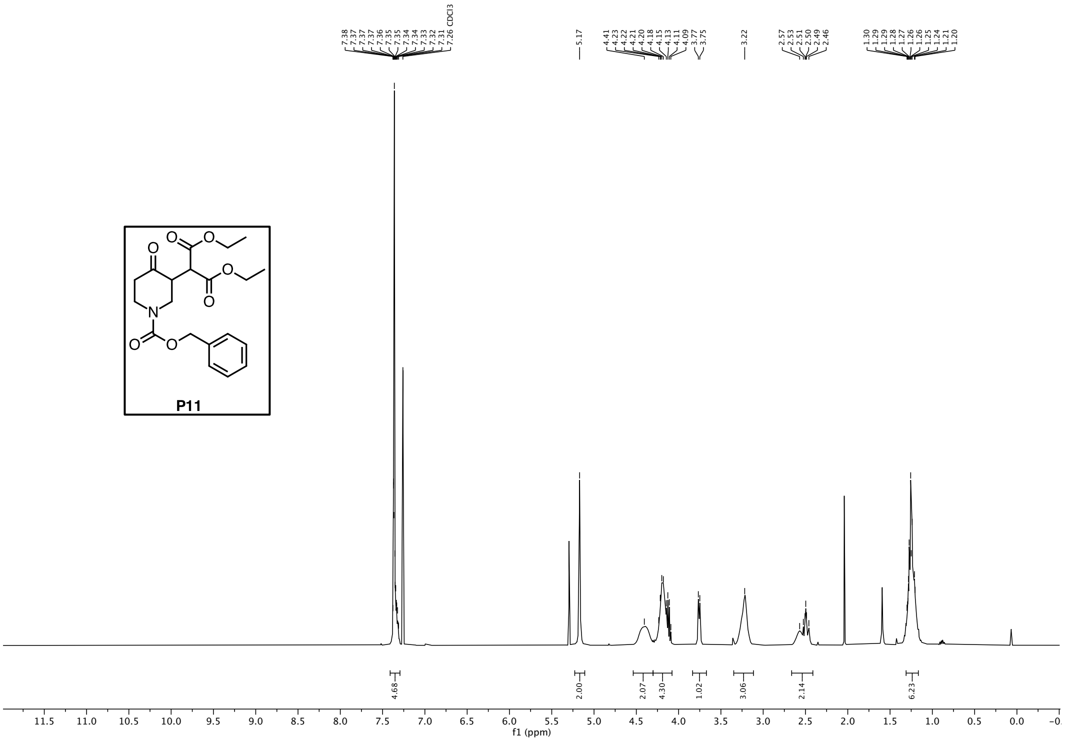

**Figure S75:** $^1$H NMR Spectra of **P11** (400 MHz, $CDCl_3$).

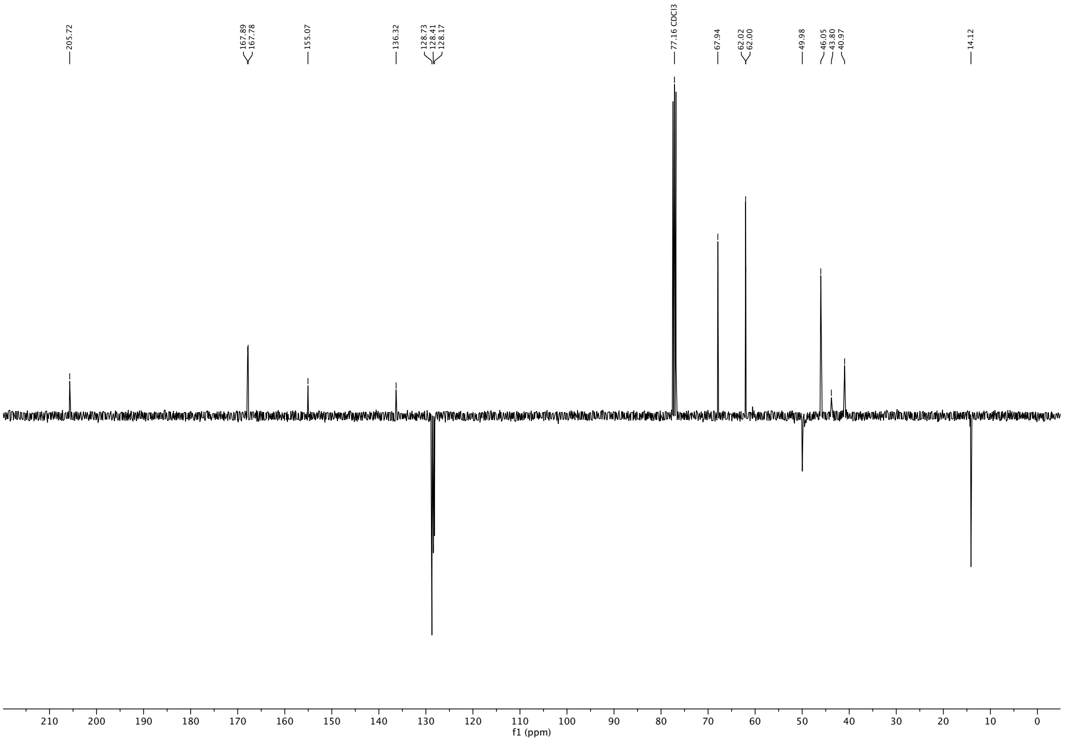

**Figure S76:** $^{13}C\{^1H\}$ NMR (APT) Spectra of **P11** (101 MHz, $CDCl_3$).

Diethyl 2-(1-(*tert*-butoxycarbonyl)-4-oxopiperidin-3-yl)malonate (**P12**)

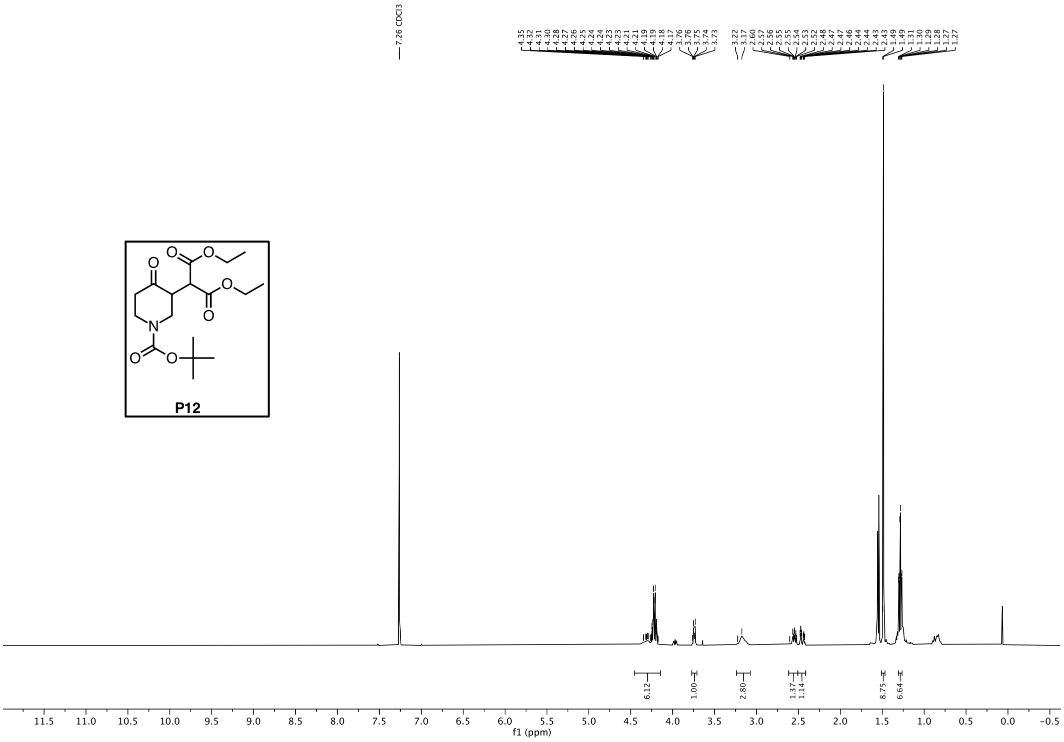


**Figure S77:** $^{1}H$ NMR Spectra of **P12** (400 MHz, $CDCl_3$).

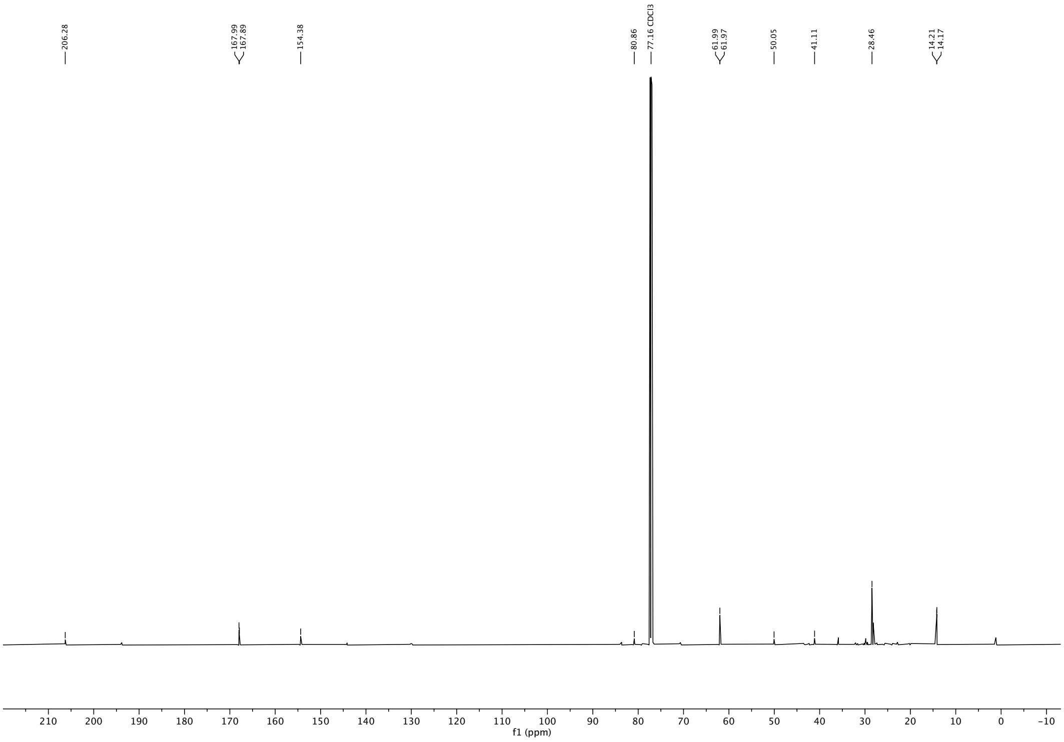


**Figure S78:** $^{13}C\{^{1}H\}$ NMR Spectra of **P12** (101 MHz, $CDCl_3$).

Diethyl 2-(8-oxo-1,4-dioxaspiro[4.5]dec-7-yl)malonate (**P13**)

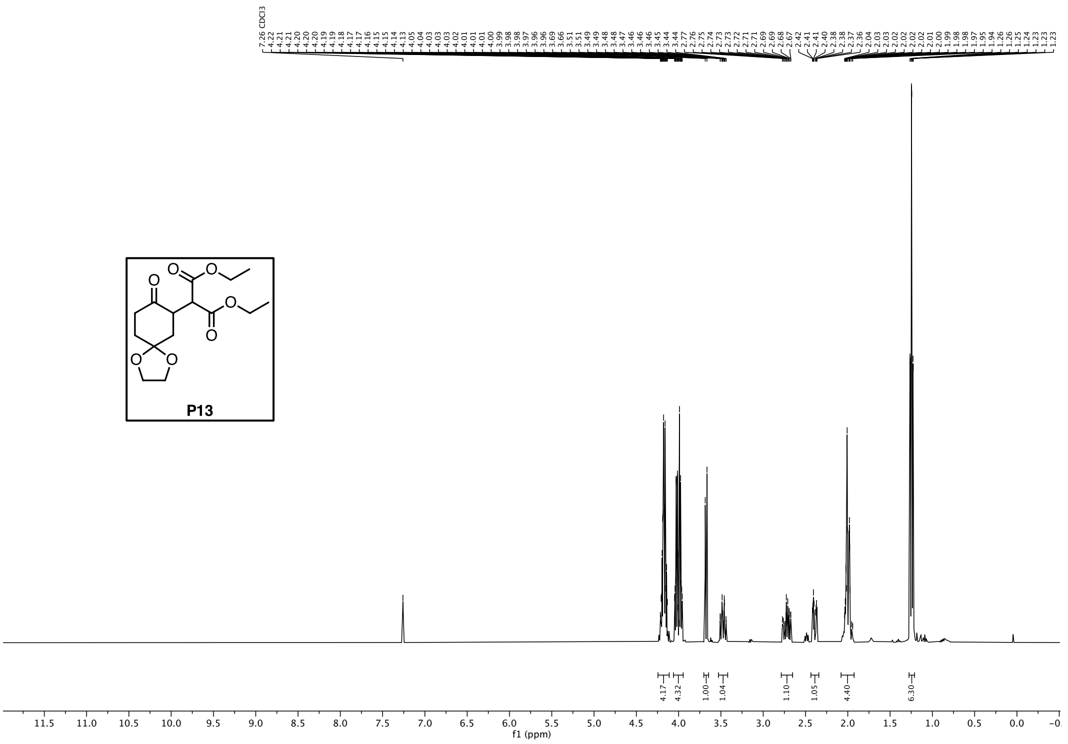

**Figure S79:** $^{1}H$ NMR Spectra of **P13** (400 MHz, $CDCl_3$).

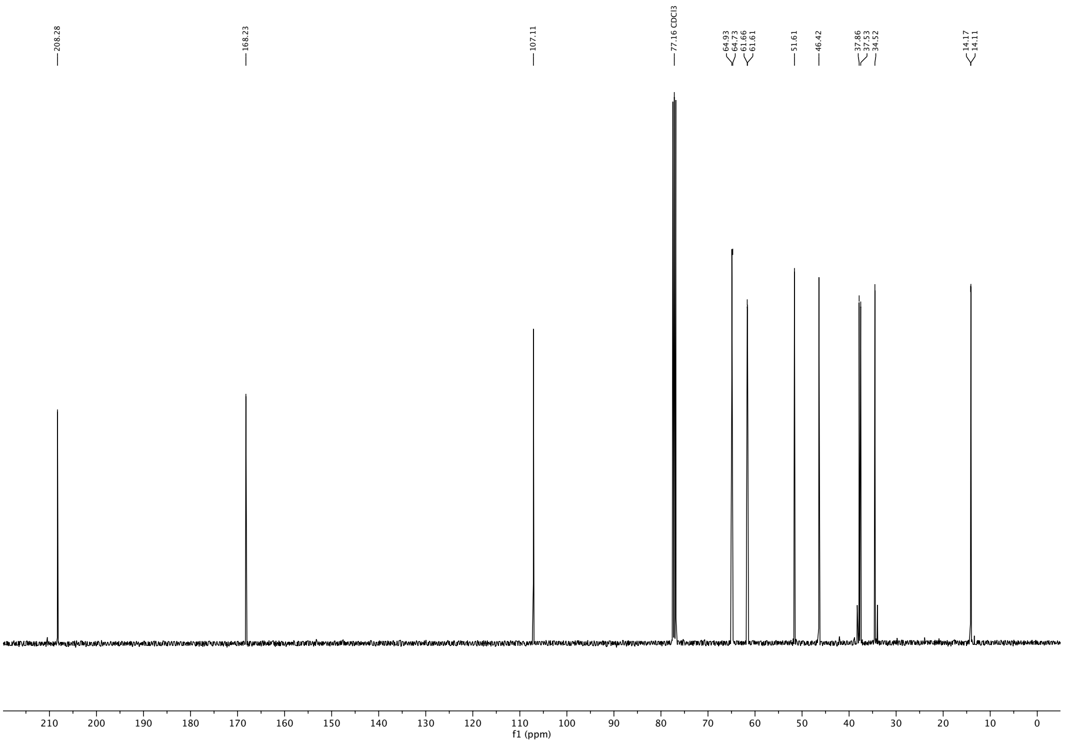

**Figure S80:** $^{13}C\{^{1}H\}$ NMR Spectra of **P13** (101 MHz, $CDCl_3$).

Diethyl 2-(3,3-dimethyl-9-oxo-1,5-dioxaspiro[5.5]undec-8-yl)malonate (**P14**)

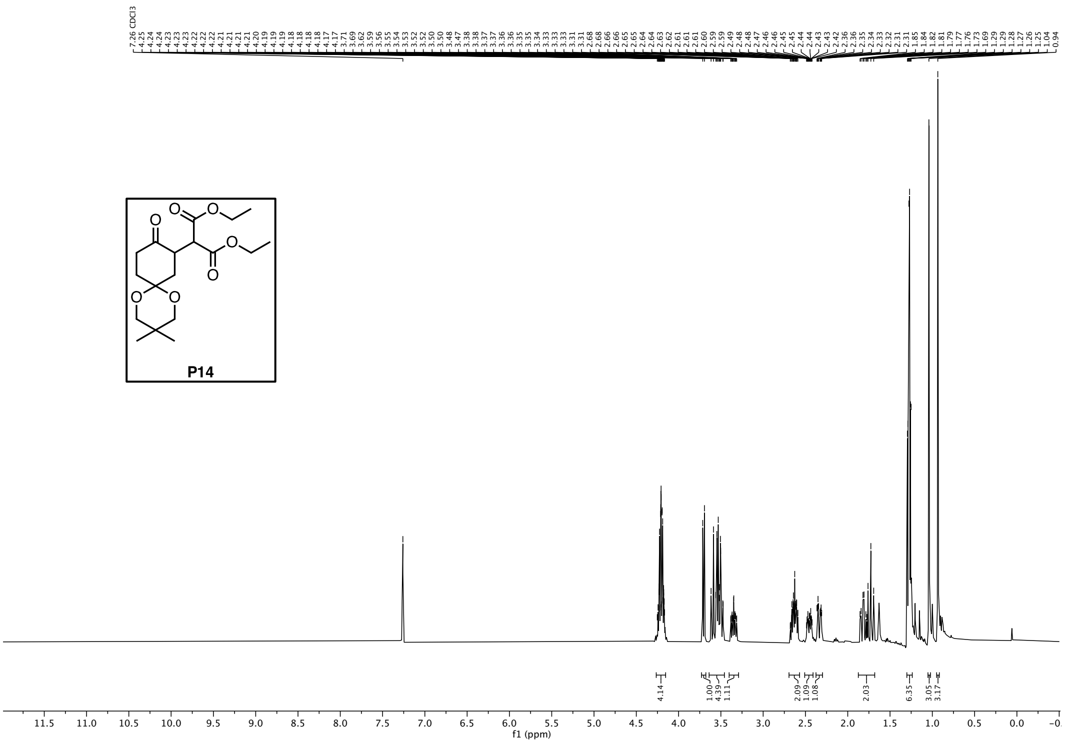

**Figure S81:** $^{1}H$ NMR Spectra of **P14** (400 MHz, $CDCl_3$).

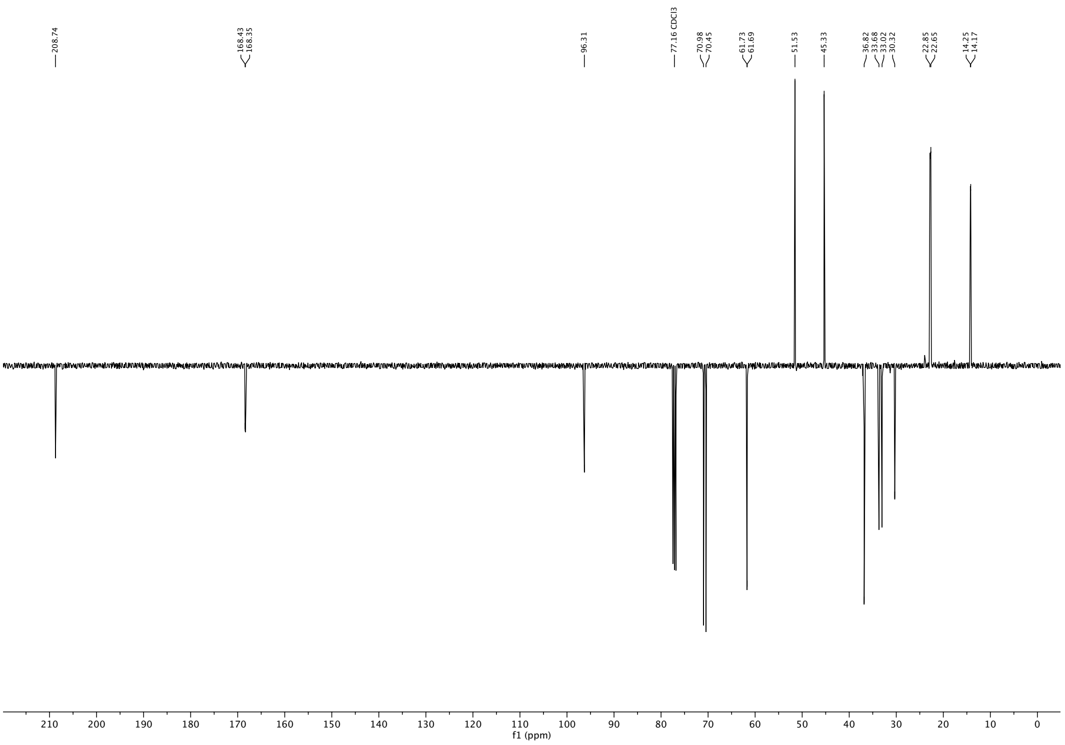

**Figure S82:** $^{13}C\{^{1}H\}$ NMR Spectra (APT) of **P14** (101 MHz, $CDCl_3$).

Dimethyl 2-(2-oxocyclohexyl)malonate (**P15**)

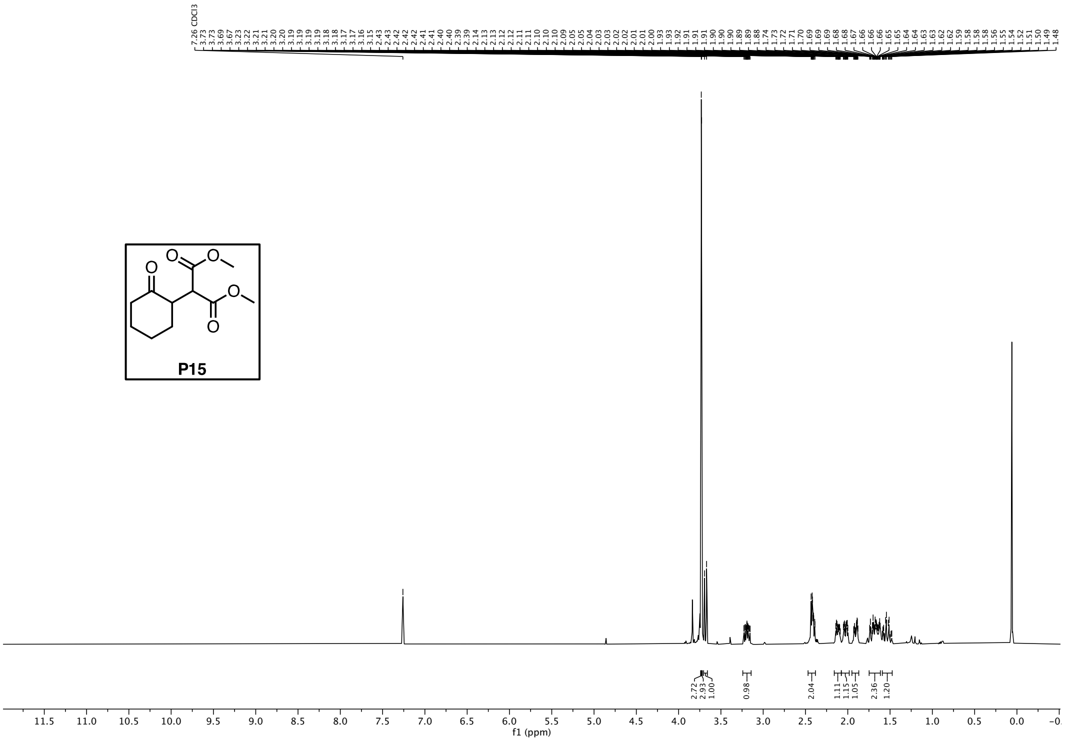


**Figure S83:** $^{1}H$ NMR Spectra of **P15** (400 MHz, $CDCl_3$).

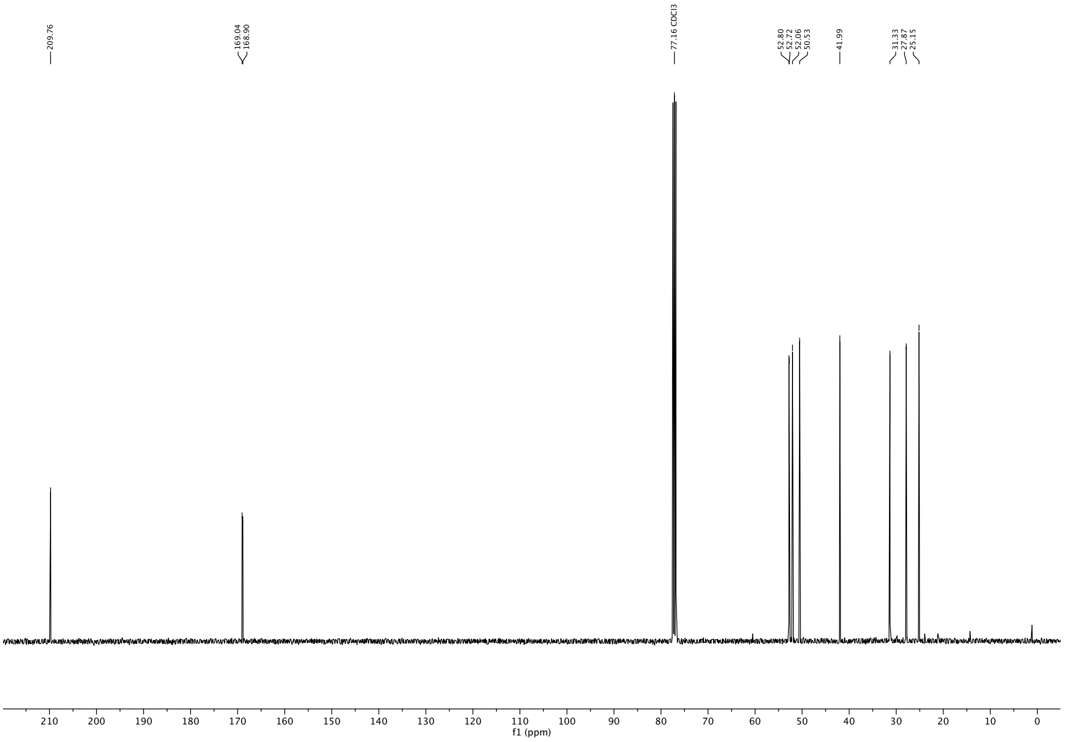


**Figure S84:** $^{13}C\{^{1}H\}$ NMR Spectra of **P15** (101 MHz, $CDCl_3$).

Dibutyl 2-(2-oxocyclohexyl)malonate (**P16**)

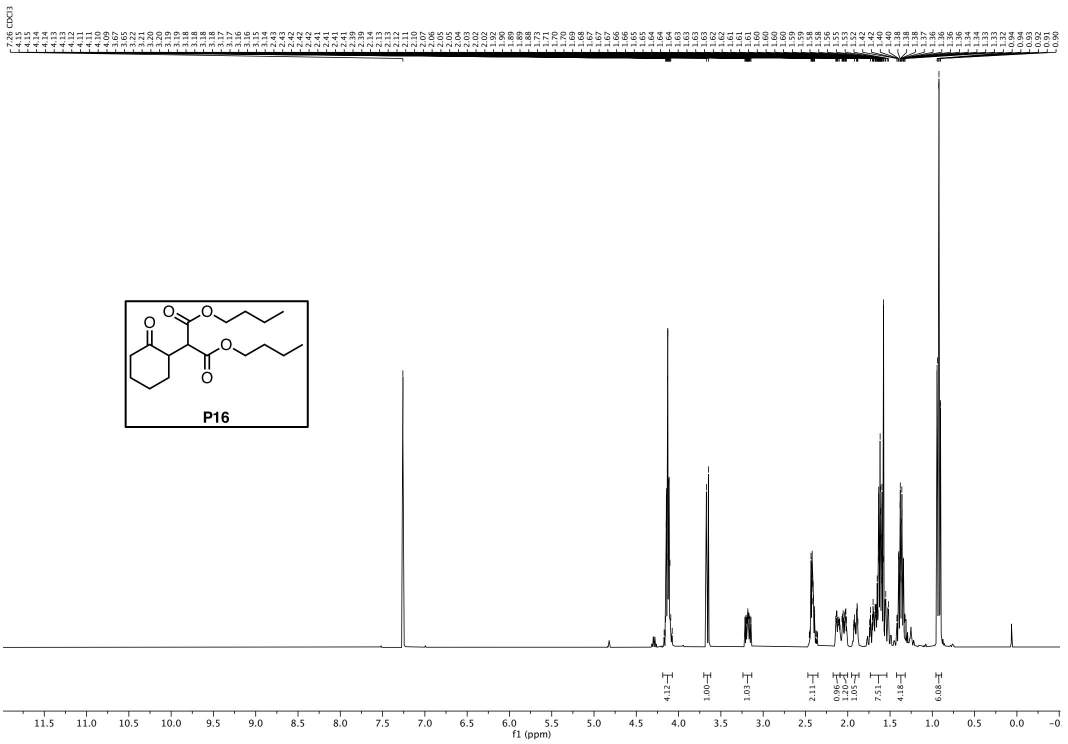

**Figure S85:** $^1H$ NMR Spectra of **P16** (400 MHz, $CDCl_3$).

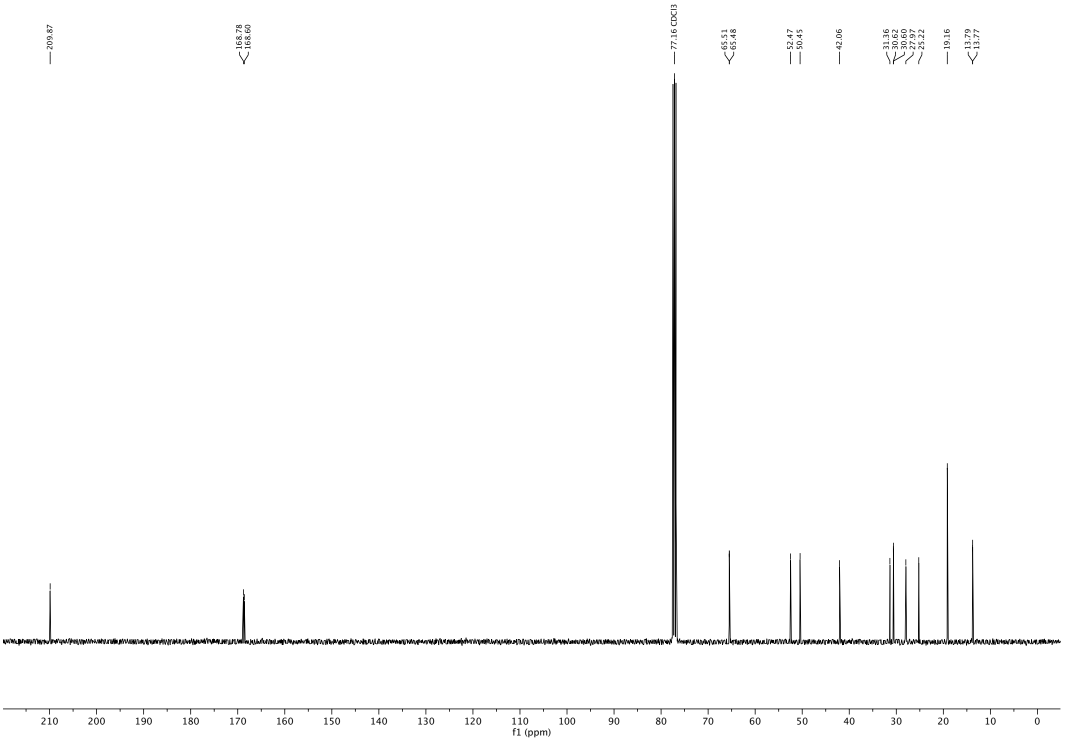

**Figure S86:** $^{13}C\{^1H\}$ NMR Spectra of **P16** (101 MHz, $CDCl_3$).

Di-*tert*-butyl 2-(2-oxocyclohexyl)malonate (**P17**)

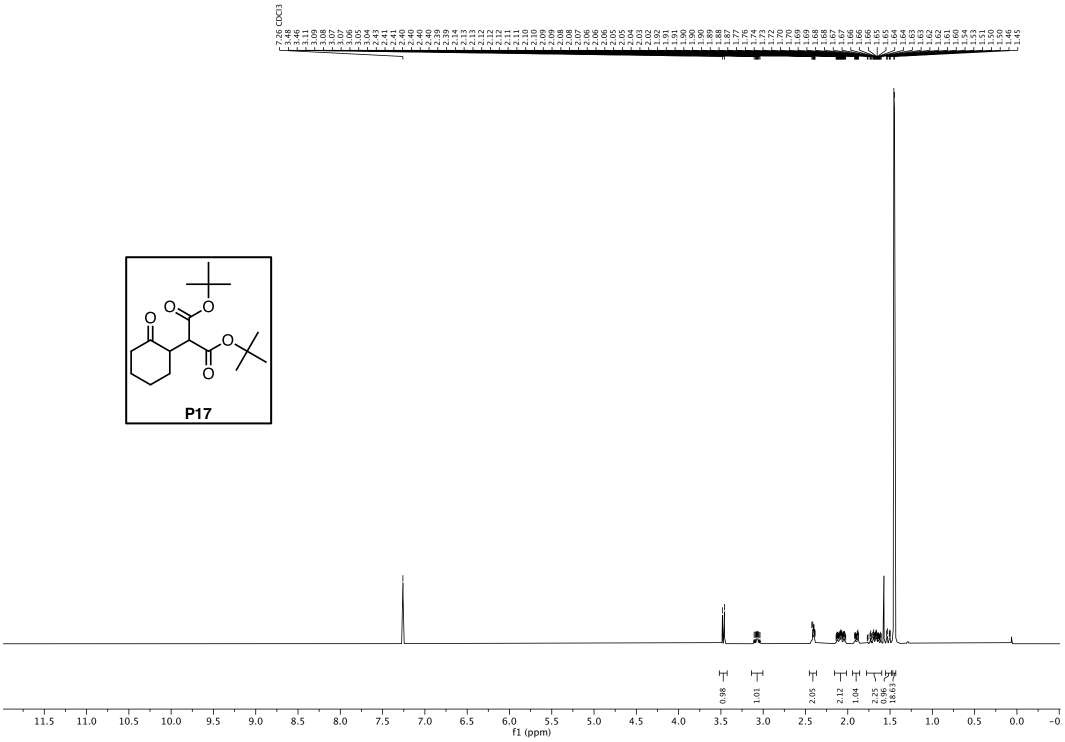


**Figure S87:** $^{1}H$ NMR Spectra of **P17** (400 MHz, $CDCl_3$).

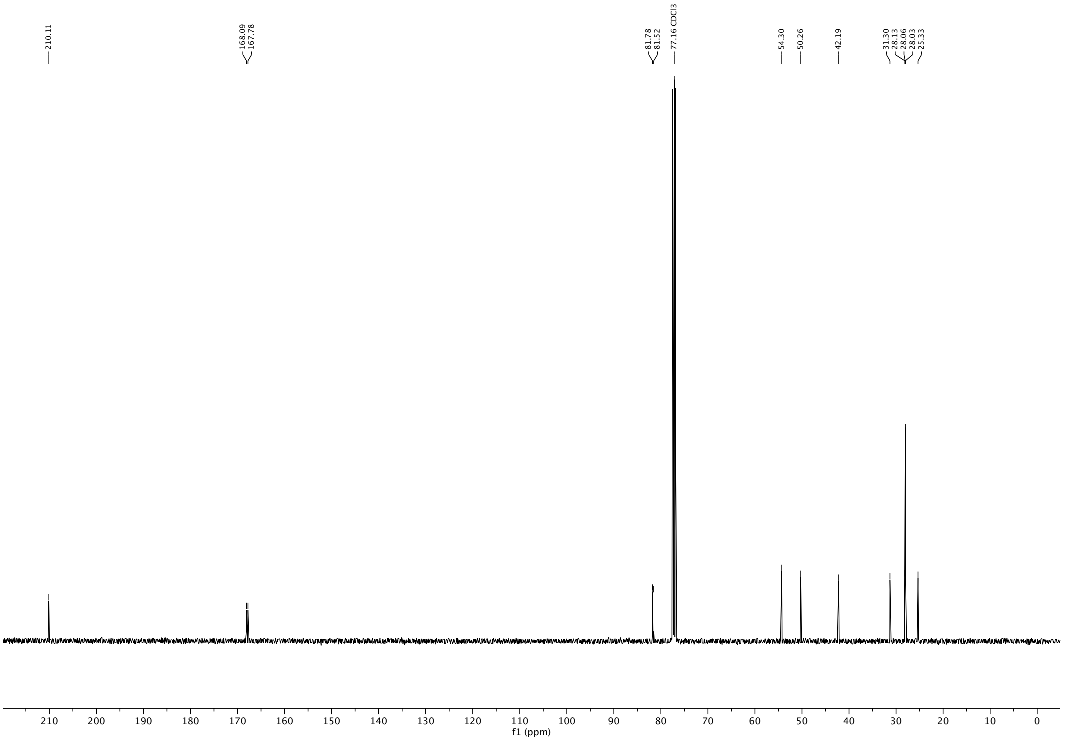


**Figure S88:** $^{13}C\{^{1}H\}$ NMR Spectra of **P17** (101 MHz, $CDCl_3$).

Di-*iso*-propyl 2-(2-oxocyclohexyl)malonate (**P18**)

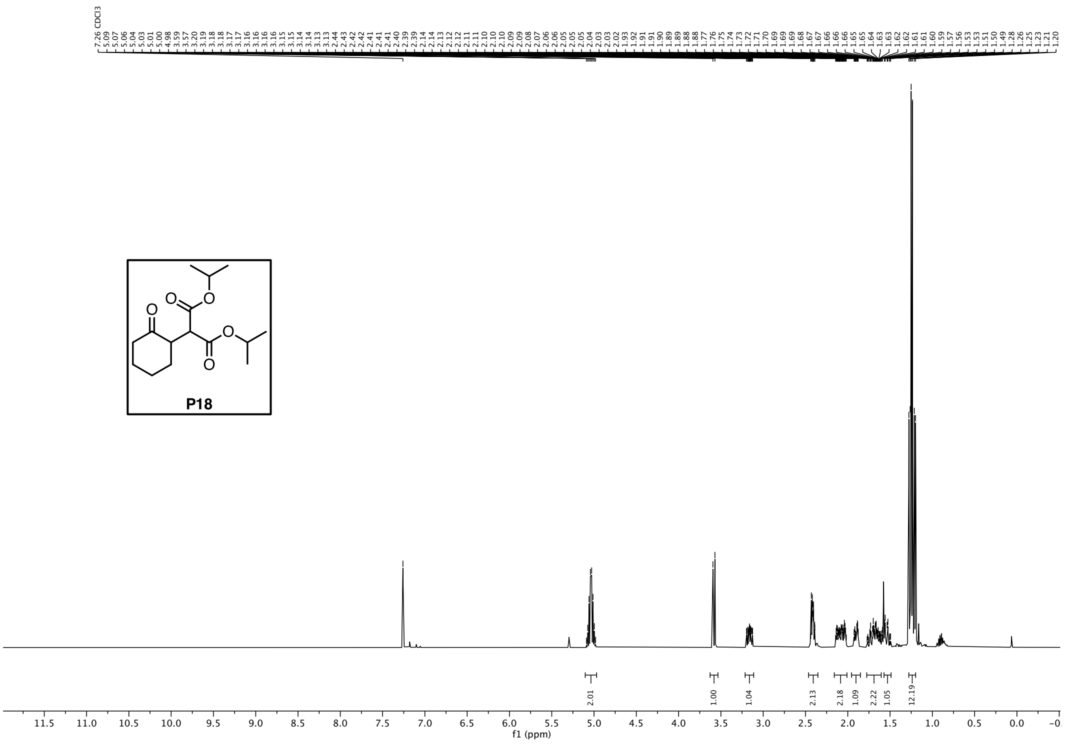


**Figure S89:** $^{1}H$ NMR Spectra of **P18** (400 MHz, $CDCl_3$).

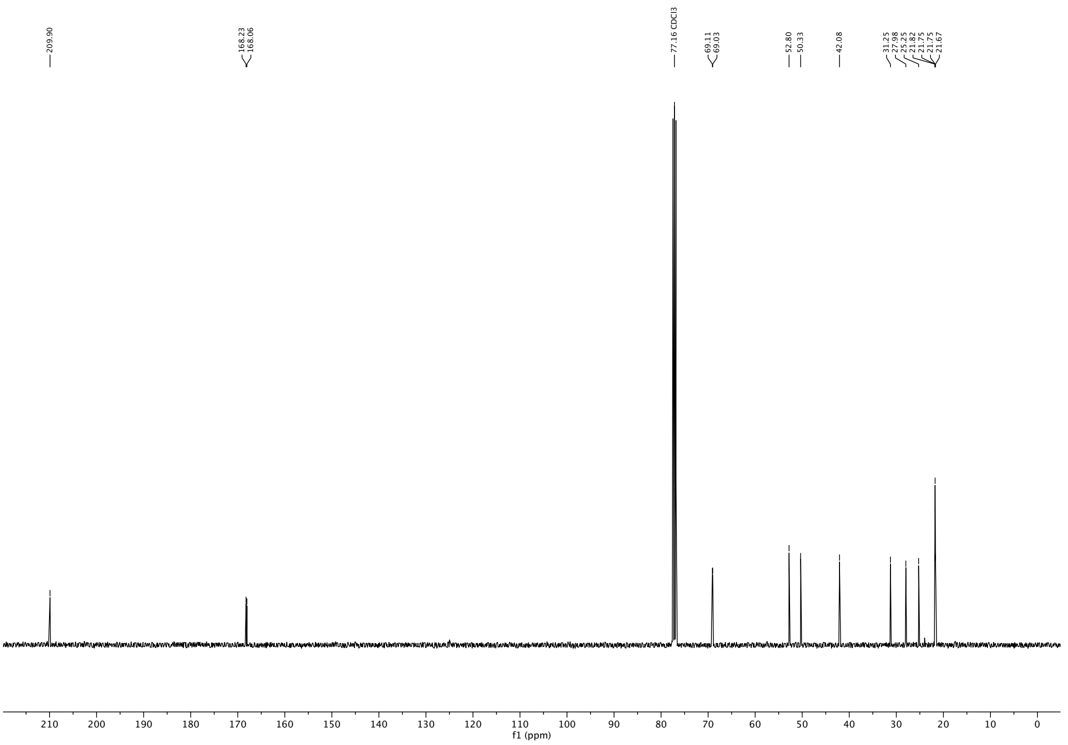


**Figure S90:** $^{13}C\{^{1}H\}$ NMR. Spectra of **P18** (101 MHz, $CDCl_3$).

Diethyl 2-(*trans*-2-hydroxycyclohexyl)malonate (***trans*-R1**)

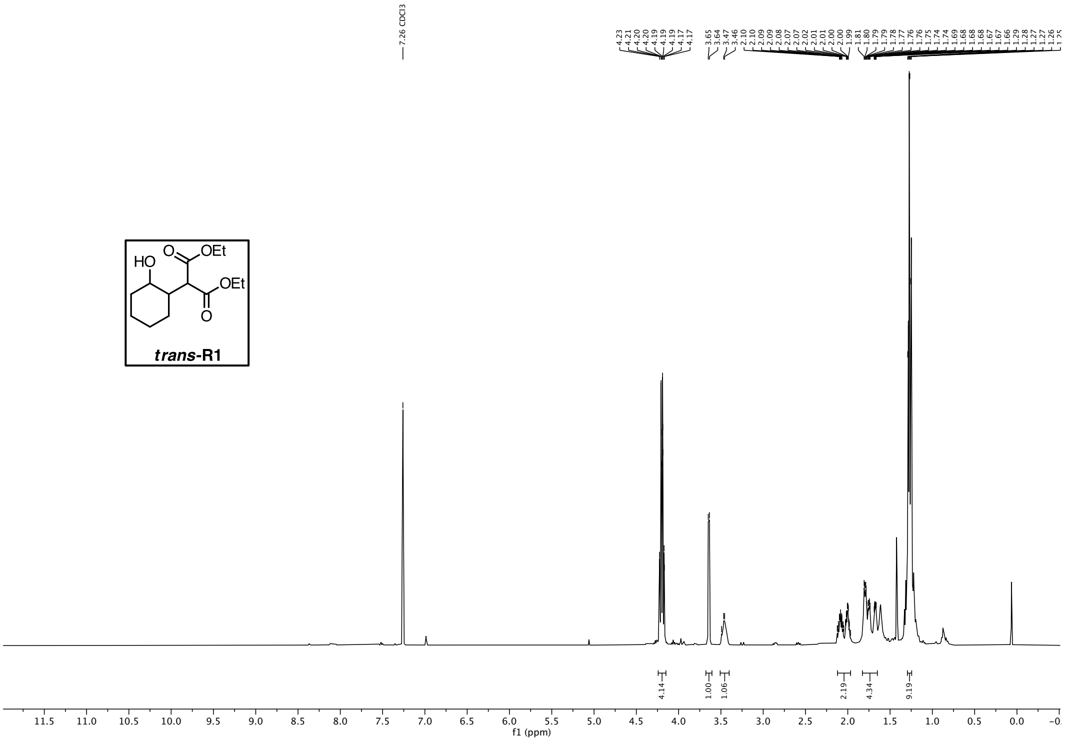


**Figure S91:** $^{1}H$ NMR Spectra of ***trans*-R1** (400 MHz, $CDCl_3$).

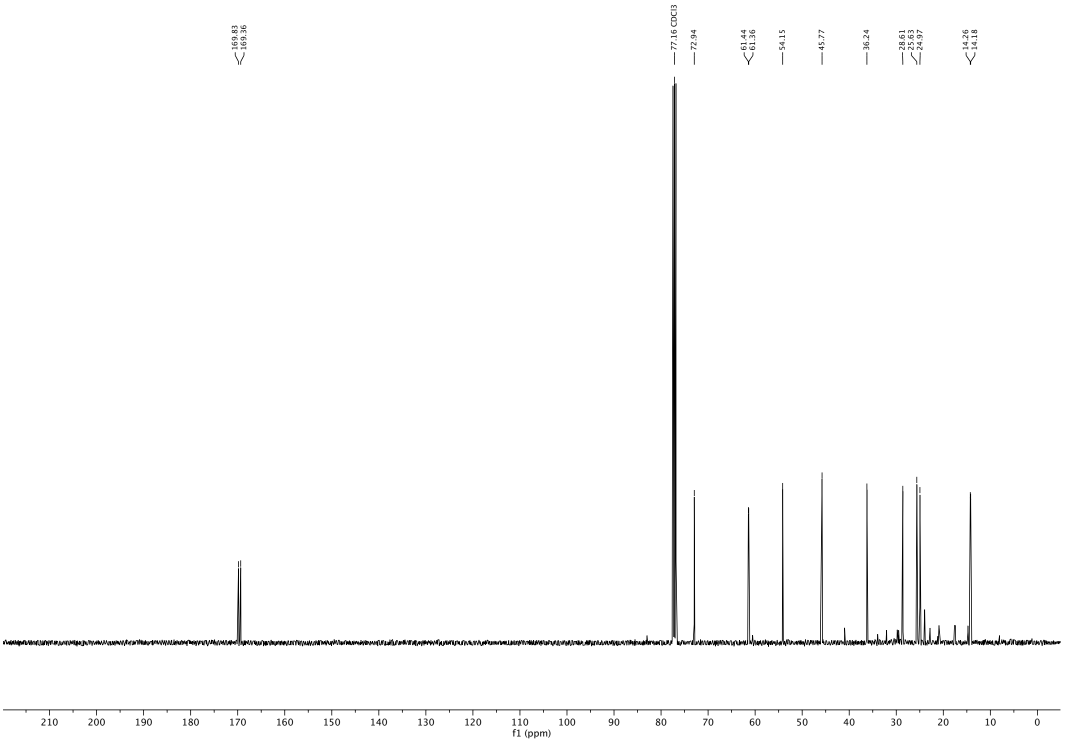


**Figure S92:** $^{13}C\{^{1}H\}$ NMR Spectra of ***trans*-R1** (101 MHz, $CDCl_3$).

Diethyl 2-(6-bromo-2,3,4,9-tetrahydro-1*H*-carbazol-1-yl)malonate (**R2**)

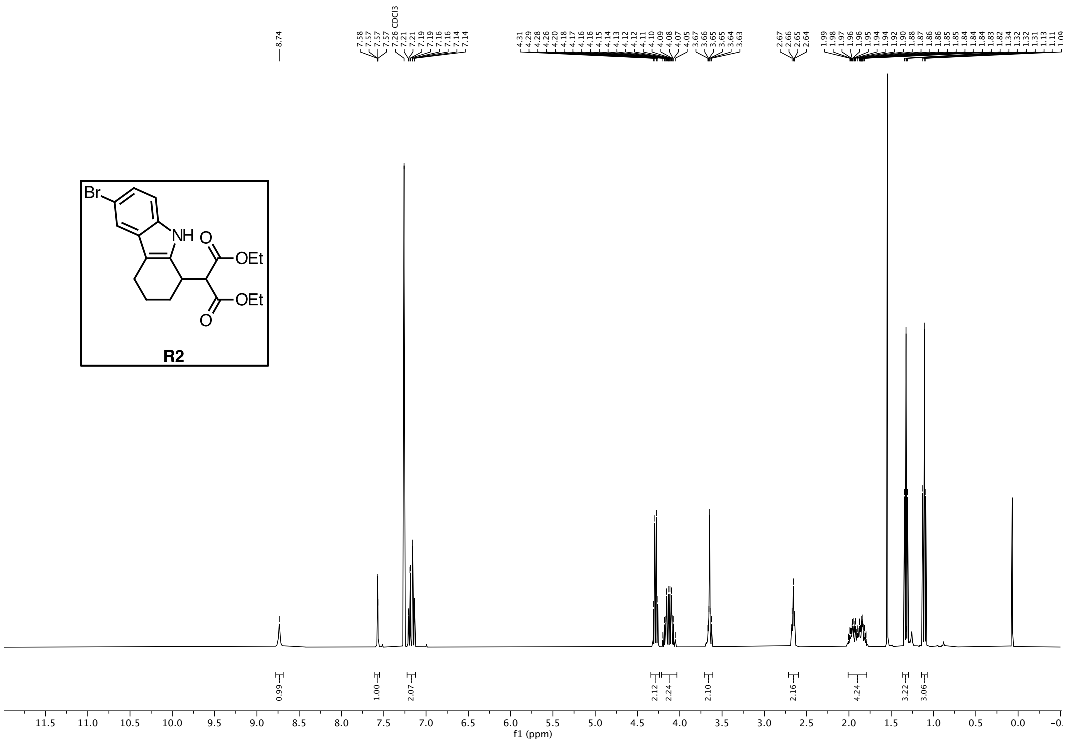

**Figure S93:** $^{1}H$ NMR Spectra of **R2** (400 MHz, $CDCl_3$).

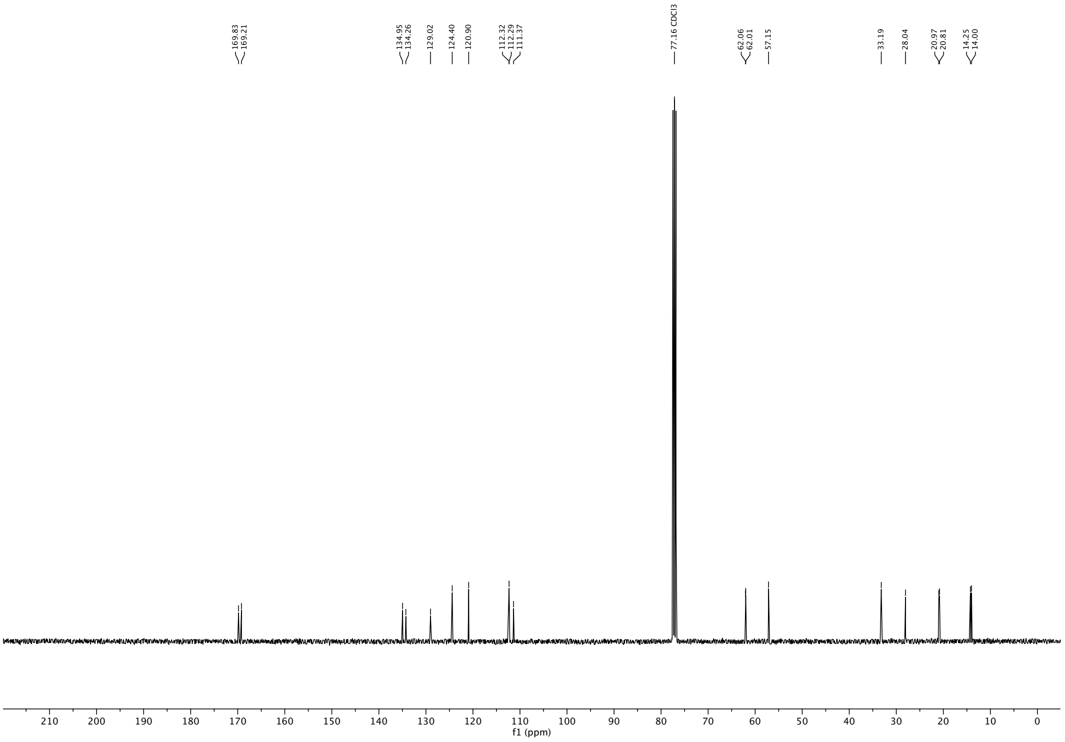

**Figure S94:** $^{13}C\{^{1}H\}$ NMR Spectra of **R2** (101 MHz, $CDCl_3$).

NMR Experiments to prove **PhQn** condensation

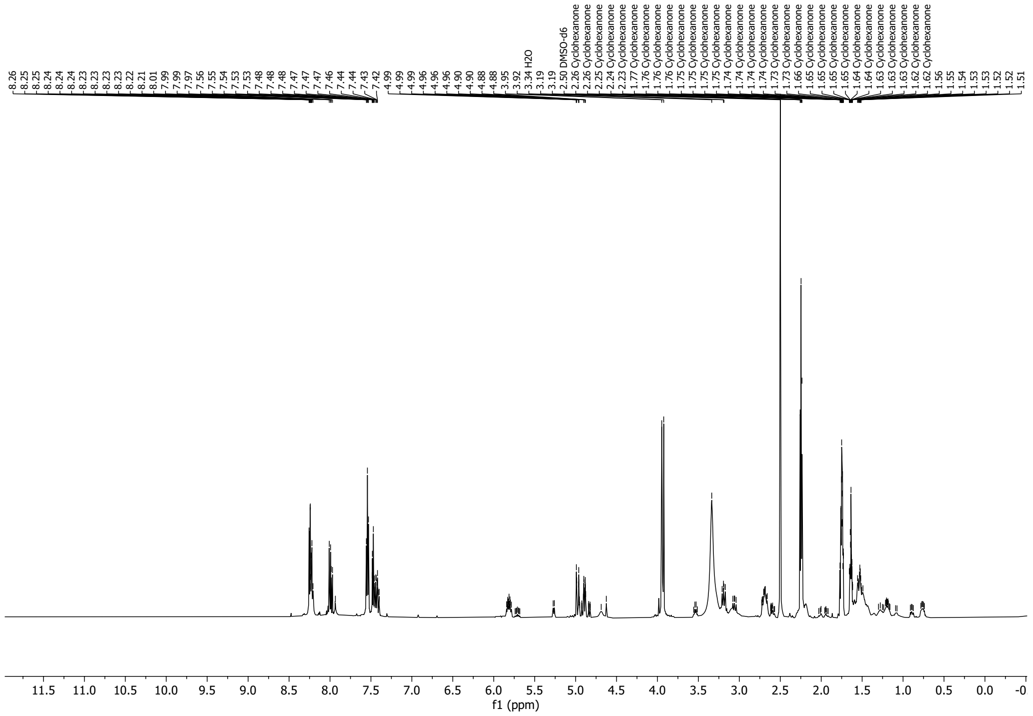

**Figure S95:** $^1$H NMR Spectra of **PhQn** and cyclohexanone (1:1) (600 MHz, DMSO-$d_6$).

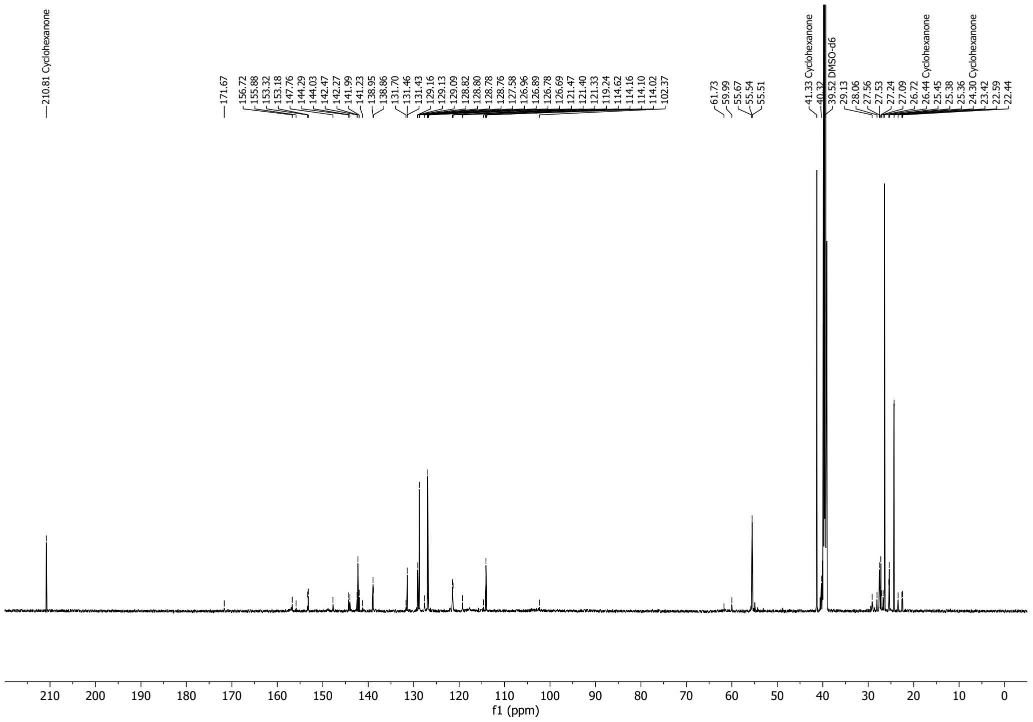

**Figure S96:** $^{13}$C{$^1$H} NMR Spectra of **PhQn** and cyclohexanone (1:1) (151 MHz, DMSO-$d_6$).

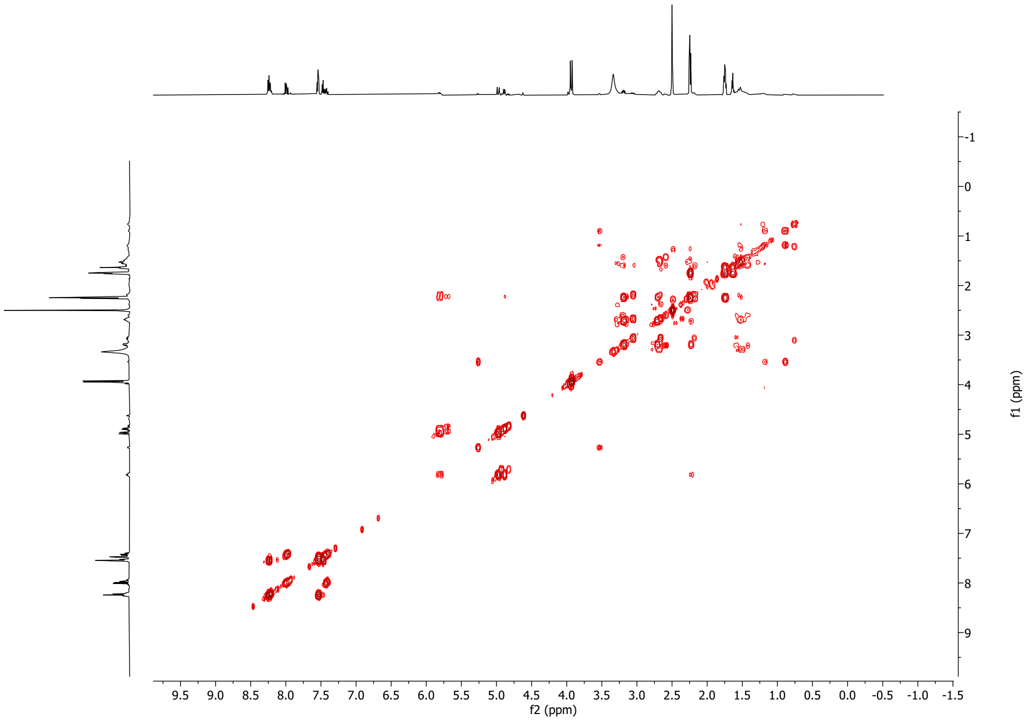


**Figure S97:** COSY Spectra of **PhQn** and cyclohexanone (1:1) (600 MHz, DMSO-d$_6$).

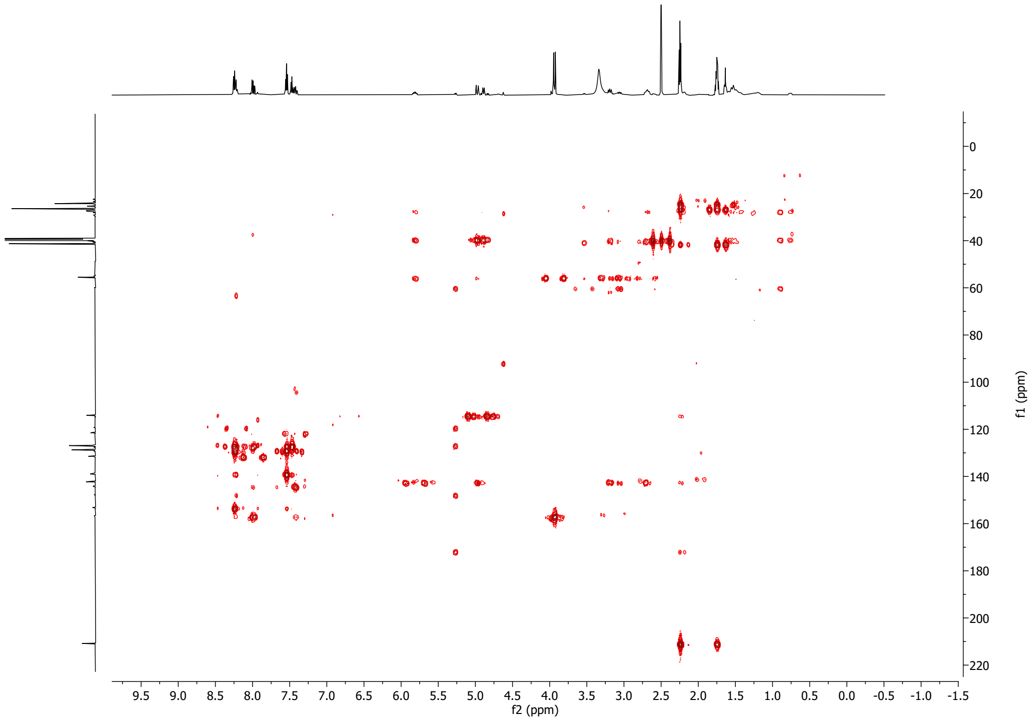


**Figure S98:** HMBC Spectra of **PhQn** and cyclohexanone (1:1) (600 and 151 MHz, DMSO-d$_6$).

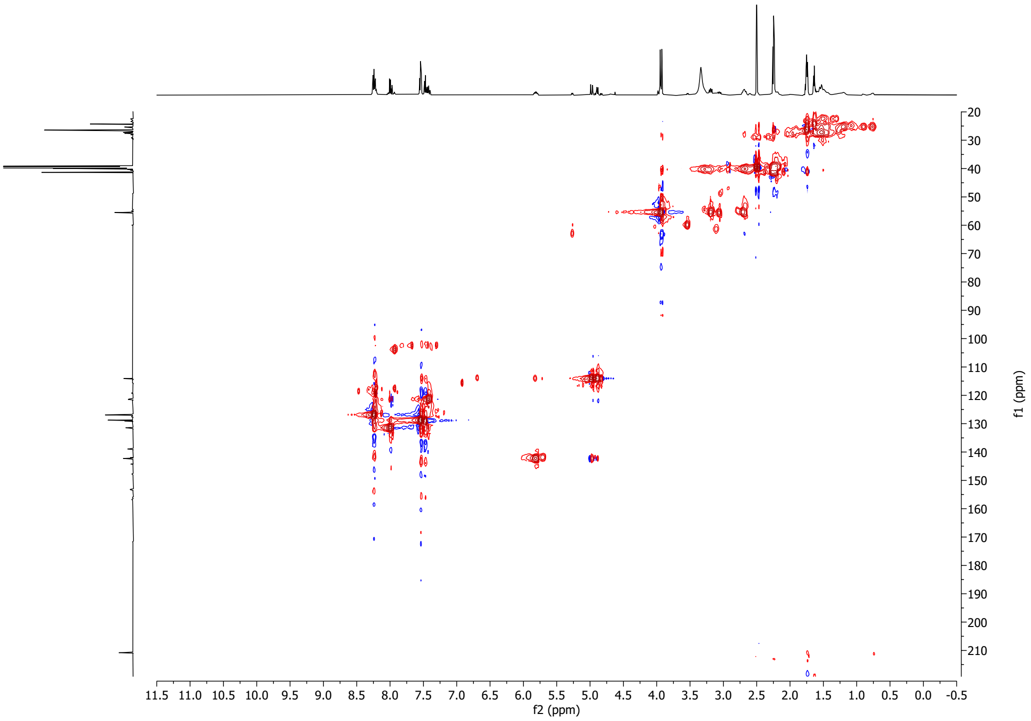


**Figure S99:** HSQC Spectra of **PhQn** and cyclohexanone (1:1) (600 and 151 MHz, DMSO-$d_6$).

# 14. Chiral HPLC Chromatograms

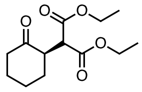


***R*-P1** using **PhQn**

| Chromatogram and Results | | | |
|---|---|---|---|
| *Injection Name:* | **MIW_204_Hept_EtOH_93_7_1mlmin_25C_1uL_60min_IA-3** | *Run Time (min):* | **44,73** |
| *Vial Number:* | **GB1** | *Report Template:* | **MIW_137_CD** |
| *Injection Volume:* | **5,00** | *Channel:* | **UV_VIS_1** |
| *Column:* | **IA-3** | *Wavelength:* | **220,0** |
| *Instrument Method:* | **Heptane_EtOH_93_7 _1mLmin_25 °C_60min** | *B %:* | **0,0** |
| *Processing Method:* | **Long alcohol** | *C %:* | **0,0** |
| *Injection Date/Time:* | **18.Sep.23 16:23** | *D %:* | **3,0** |
| *Operator:* | **Irena Jelenkovic - Didic** | | |

**Chromatogram**

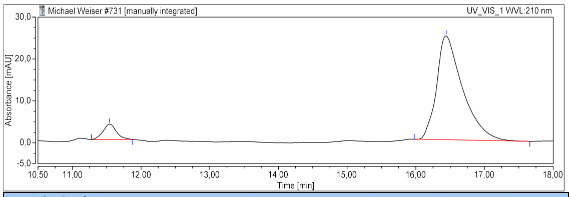


**Integration Results**

| No. | Peak Name | Retention Time min | Area mAU*min | Height mAU | Relative Area % | Relative Height % | Amount n.a. |
|---|---|---|---|---|---|---|---|
| 1 | | 11,540 | 0,835 | 3,726 | 7,14 | 13,05 | n.a. |
| 2 | | 16,442 | 10,870 | 24,817 | 92,86 | 86,95 | n.a. |
| **Total:** | | | **11,706** | **28,542** | **100,00** | **100,00** | |

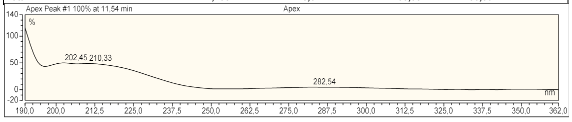


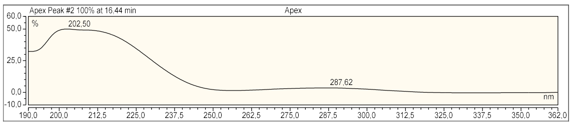


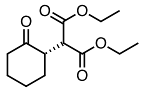


***S*-P1** using **PhQd**

| Chromatogram and Results | | | |
|---|---|---|---|
| *Injection Name:* | **MIW_224_Hept_EtOH_93_7_1mlmin_25C_1uL_60min_IA-3** | *Run Time (min):* | **19,31** |
| *Vial Number:* | **GA1** | *Report Template:* | **MIW_137_CD** |
| *Injection Volume:* | **10,00** | *Channel:* | **UV_VIS_1** |
| *Column:* | **IA-3** | *Wavelength:* | **220,0** |
| *Instrument Method:* | **Heptane_EtOH_93_7 _1mLmin_25 °C_60min** | *B %:* | **0,0** |
| *Processing Method:* | **Long alcohol** | *C %:* | **0,0** |
| *Injection Date/Time:* | **29.Sep.23 11:24** | *D %:* | **3,0** |
| *Operator:* | **Irena Jelenkovic - Didic** | | |

**Chromatogram**

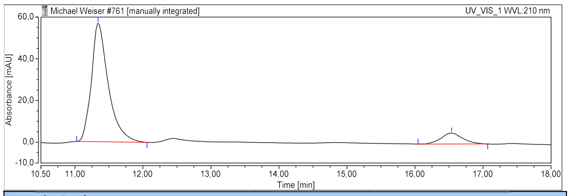


**Integration Results**

| No. | Peak Name | Retention Time min | Area mAU*min | Height mAU | Relative Area % | Relative Height % | Amount n.a. |
|---|---|---|---|---|---|---|---|
| 1 | | 11,342 | 16,389 | 56,800 | 89,43 | 91,50 | n.a. |
| 2 | | 16,538 | 1,936 | 5,278 | 10,57 | 8,50 | n.a. |
| **Total:** | | | **18,326** | **62,077** | **100,00** | **100,00** | |

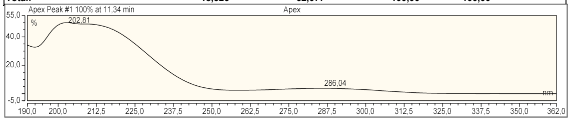


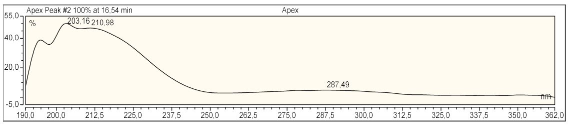


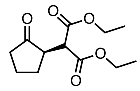


***R*-P2** using **PhQn**

| Chromatogram and Results | | | |
|---|---|---|---|
| *Injection Name:* | **MIW_111_Hept_EtOH_90_10_1mlmin_25C_1uL_60min_IA-3** | *Run Time (min):* | **15,41** |
| *Vial Number:* | **RE1** | *Report Template:* | **MIW_137_CD** |
| *Injection Volume:* | **1,00** | *Channel:* | **UV_VIS_1** |
| *Column:* | **IA-3** | *Wavelength:* | **220,0** |
| *Instrument Method:* | **Heptane_EtOH_90 _10_1mLmin_60min_25 °C** | *B %:* | **0,0** |
| *Processing Method:* | **Long alcohol** | *C %:* | **0,0** |
| *Injection Date/Time:* | **03.Nov.22 13:49** | *D %:* | **10,0** |
| *Operator:* | **Irena Jelenkovic - Didic** | | |

**Chromatogram**

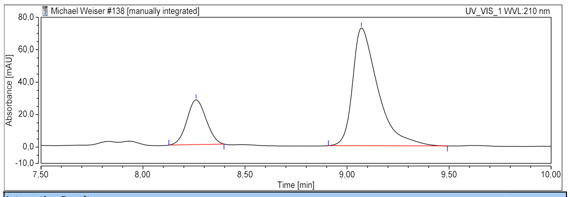


**Integration Results**

| No. | Peak Name | Retention Time min | Area mAU*min | Height mAU | Relative Area % | Relative Height % | Amount n.a. |
|---|---|---|---|---|---|---|---|
| 1 | | 8,260 | 3,009 | 27,578 | 22,37 | 27,54 | n.a. |
| 2 | | 9,070 | 10,441 | 72,546 | 77,63 | 72,46 | n.a. |
| **Total:** | | | **13,450** | **100,124** | **100,00** | **100,00** | |

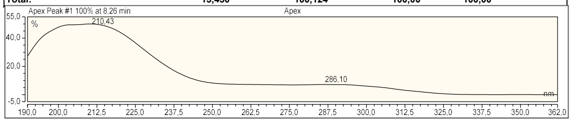


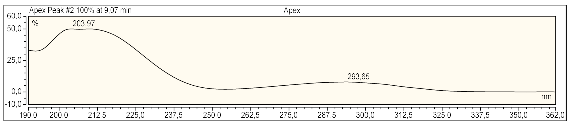


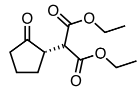


***S*-P2** using **PhQd**

| Chromatogram and Results | | | |
|---|---|---|---|
| *Injection Name:* | **MIW_191_fr1_Hept_EtOH_90_10_1mlmin_25C_1uL_60min_I/** | *Run Time (min):* | **12,29** |
| *Vial Number:* | **GA1** | *Report Template:* | **MIW_137_CD** |
| *Injection Volume:* | **10,00** | *Channel:* | **UV_VIS_1** |
| *Column:* | **IA-3** | *Wavelength:* | **220,0** |
| *Instrument Method:* | **Heptane_EtOH_90 _10_1mLmin_60min_25 °C** | *B %:* | **0,0** |
| *Processing Method:* | **Long alcohol** | *C %:* | **0,0** |
| *Injection Date/Time:* | **10.Aug.23 15:30** | *D %:* | **10,0** |
| *Operator:* | **Irena Jelenkovic - Didic** | | |

**Chromatogram**

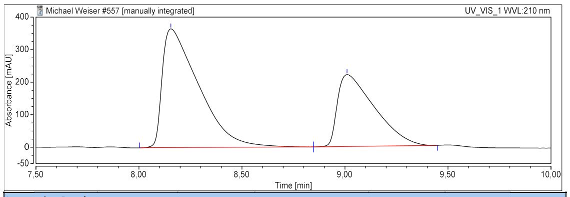


**Integration Results**

| No. | Peak Name | Retention Time min | Area mAU*min | Height mAU | Relative Area % | Relative Height % | Amount n.a. |
|---|---|---|---|---|---|---|---|
| 1 | | 8,155 | 74,769 | 364,571 | 61,86 | 62,26 | n.a. |
| 2 | | 9,010 | 46,100 | 220,957 | 38,14 | 37,74 | n.a. |
| **Total:** | | | **120,869** | **585,528** | **100,00** | **100,00** | |

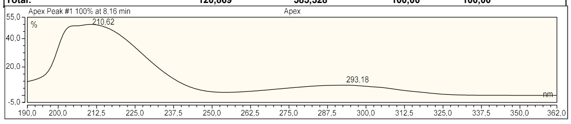


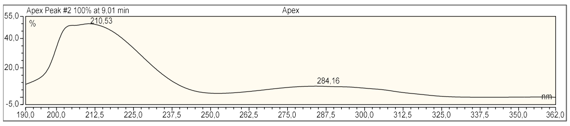

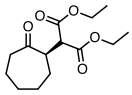

***R*-P3** using **PhQn**

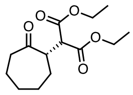

***S*-P3** using **PhQd**

### Chromatogram and Results

| | | | |
|---|---|---|---|
| *Injection Name:* | **MIW_189_AB_Hept_EtOH_90_10_1mlmin_25C_1uL_60min_I** | *Run Time (min):* | **22,62** |
| *Vial Number:* | **GA1** | *Report Template:* | **MIW_137_CD** |
| *Injection Volume:* | **10,00** | *Channel:* | **UV_VIS_3** |
| *Column:* | **IA-3** | *Wavelength:* | **220,0** |
| *Instrument Method:* | **Heptane_EtOH_90 _10_1mLmin_60min_25 °C** | *B %:* | **0,0** |
| *Processing Method:* | **Long alcohol** | *C %:* | **0,0** |
| *Injection Date/Time:* | **04.Aug.23 12:04** | *D %:* | **10,0** |
| *Operator:* | **Irena Jelenkovic - Didic** | | |

**Chromatogram**

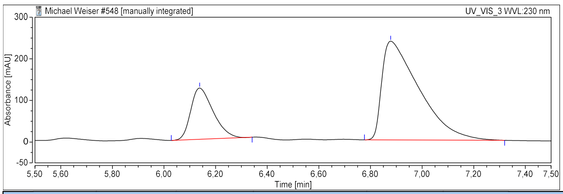


**Integration Results**

| No. | Peak Name | Retention Time min | Area mAU*min | Height mAU | Relative Area % | Relative Height % | Amount n.a. |
|---|---|---|---|---|---|---|---|
| 1 | | 6,138 | 12,388 | 123,164 | 23,81 | 34,16 | n.a. |
| 2 | | 6,878 | 39,636 | 237,405 | 76,19 | 65,84 | n.a. |
| **Total:** | | | **52,024** | **360,569** | **100,00** | **100,00** | |

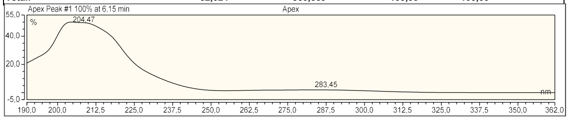


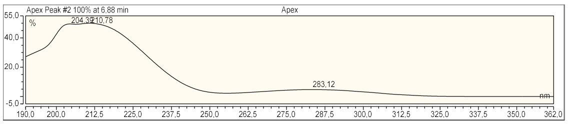


### Chromatogram and Results

| | | | |
|---|---|---|---|
| *Injection Name:* | **MIW_189_CD_Hept_EtOH_90_10_1mlmin_25C_1uL_60min_I** | *Run Time (min):* | **12,58** |
| *Vial Number:* | **GA2** | *Report Template:* | **MIW_137_CD** |
| *Injection Volume:* | **10,00** | *Channel:* | **UV_VIS_3** |
| *Column:* | **IA-3** | *Wavelength:* | **220,0** |
| *Instrument Method:* | **Heptane_EtOH_90 _10_1mLmin_60min_25 °C** | *B %:* | **0,0** |
| *Processing Method:* | **Long alcohol** | *C %:* | **0,0** |
| *Injection Date/Time:* | **04.Aug.23 12:29** | *D %:* | **10,0** |
| *Operator:* | **Irena Jelenkovic - Didic** | | |

**Chromatogram**

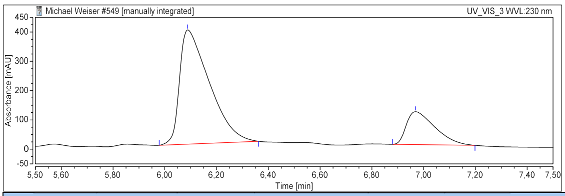


**Integration Results**

| No. | Peak Name | Retention Time min | Area mAU*min | Height mAU | Relative Area % | Relative Height % | Amount n.a. |
|---|---|---|---|---|---|---|---|
| 1 | | 6,088 | 51,961 | 389,642 | 78,40 | 77,64 | n.a. |
| 2 | | 6,968 | 14,315 | 112,197 | 21,60 | 22,36 | n.a. |
| **Total:** | | | **66,277** | **501,839** | **100,00** | **100,00** | |

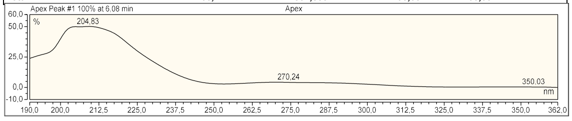


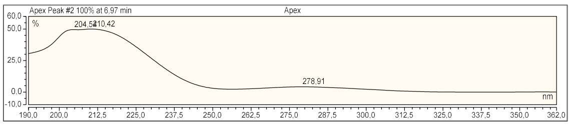


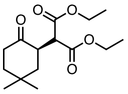

***R*-P4** using **PhQn**

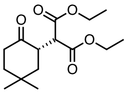

***S*-P4** using **PhQd**

### Chromatogram and Results

| | | | |
|---|---|---|---|
| *Injection Name:* | **MIW_183_AB_Hept_EtOH_99_1_1mlmin_25C_1uL_60min_IA** | *Run Time (min):* | **28,88** |
| *Vial Number:* | **GA1** | *Report Template:* | **MIW_137_CD** |
| *Injection Volume:* | **10,00** | *Channel:* | **UV_VIS_1** |
| *Column:* | **IA-3** | *Wavelength:* | **254,0** |
| *Instrument Method:* | **Heptane_EtOH_99 _1_1mLmin_60min_25 °C** | *B %:* | **0,0** |
| *Processing Method:* | **Long alcohol** | *C %:* | **0,0** |
| *Injection Date/Time:* | **23.Jun.23 11:45** | *D %:* | **1,0** |
| *Operator:* | **Irena Jelenkovic - Didic** | | |

**Chromatogram**

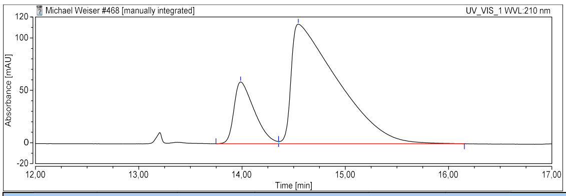


**Integration Results**

| No. | Peak Name | Retention Time min | Area mAU*min | Height mAU | Relative Area % | Relative Height % | Amount n.a. |
|---|---|---|---|---|---|---|---|
| 1 | | 13,987 | 13,484 | 58,976 | 18,47 | 34,08 | n.a. |
| 2 | | 14,545 | 59,536 | 114,099 | 81,53 | 65,92 | n.a. |
| **Total:** | | | **73,020** | **173,075** | **100,00** | **100,00** | |

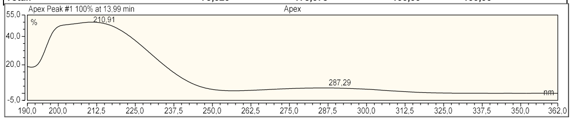


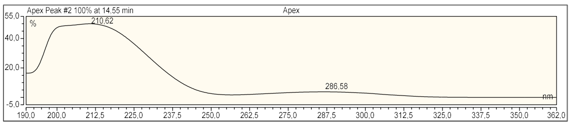


### Chromatogram and Results

| | | | |
|---|---|---|---|
| *Injection Name:* | **MIW_186_AB_Hept_EtOH_99_1_1mlmin_25C_1uL_60min_IA** | *Run Time (min):* | **17,66** |
| *Vial Number:* | **GA1** | *Report Template:* | **MIW_137_CD** |
| *Injection Volume:* | **10,00** | *Channel:* | **UV_VIS_1** |
| *Column:* | **IA-3** | *Wavelength:* | **254,0** |
| *Instrument Method:* | **Heptane_EtOH_99 _1_1mLmin_60min_25 °C** | *B %:* | **0,0** |
| *Processing Method:* | **Long alcohol** | *C %:* | **0,0** |
| *Injection Date/Time:* | **05.Jul.23 13:15** | *D %:* | **1,0** |
| *Operator:* | **Irena Jelenkovic - Didic** | | |

**Chromatogram**

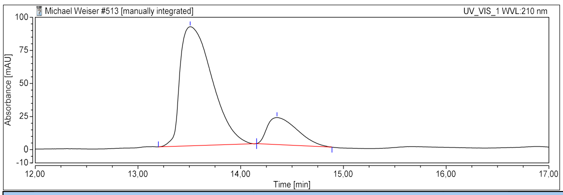


**Integration Results**

| No. | Peak Name | Retention Time min | Area mAU*min | Height mAU | Relative Area % | Relative Height % | Amount n.a. |
|---|---|---|---|---|---|---|---|
| 1 | | 13,508 | 32,426 | 89,878 | 82,32 | 81,50 | n.a. |
| 2 | | 14,353 | 6,966 | 20,402 | 17,68 | 18,50 | n.a. |
| **Total:** | | | **39,392** | **110,280** | **100,00** | **100,00** | |

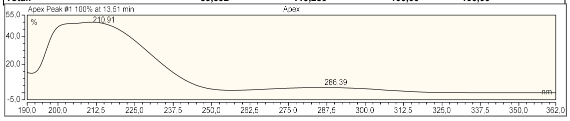


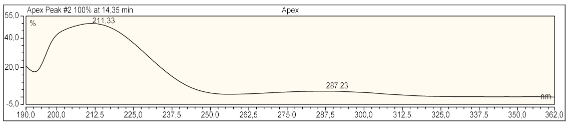

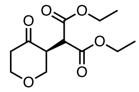

***R*-P5** using **PhQn**

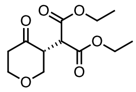

***S*-P5** using **PhQd**

## Chromatogram and Results

| | | | |
|---|---|---|---|
| *Injection Name:* | **MIW_145_AB_crude_Hept_EtOH_90_10_1mlmin_25C_1uL_6** | *Run Time (min):* | **21,94** |
| *Vial Number:* | **GB1** | *Report Template:* | **MIW_137_CD** |
| *Injection Volume:* | **1,00** | *Channel:* | **UV_VIS_1** |
| *Column:* | **IA-3** | *Wavelength:* | **220,0** |
| *Instrument Method:* | **Heptane_EtOH_90 _10_1mLmin_60min_25 °C** | *B %:* | **0,0** |
| *Processing Method:* | **Long alcohol** | *C %:* | **0,0** |
| *Injection Date/Time:* | **11.Mai.23 15:29** | *D %:* | **10,0** |
| *Operator:* | **Irena Jelenkovic - Didic** | | |

**Chromatogram**

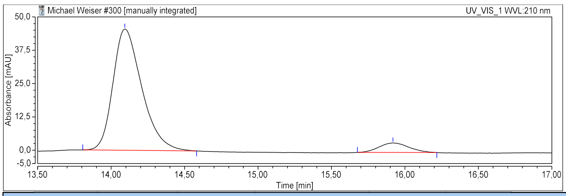


**Integration Results**

| No. | Peak Name | Retention Time min | Area mAU*min | Height mAU | Relative Area % | Relative Height % | Amount n.a. |
|---|---|---|---|---|---|---|---|
| 1 | | 14,093 | 10,356 | 45,460 | 92,47 | 92,67 | n.a. |
| 2 | | 15,918 | 0,843 | 3,594 | 7,53 | 7,33 | n.a. |
| **Total:** | | | **11,199** | **49,054** | **100,00** | **100,00** | |

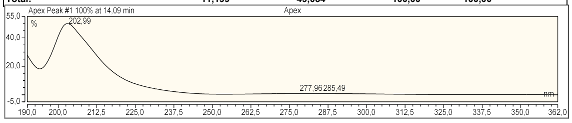


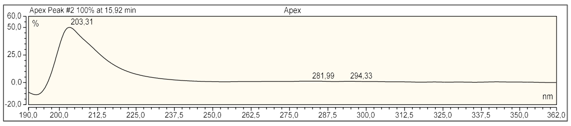


## Chromatogram and Results

| | | | |
|---|---|---|---|
| *Injection Name:* | **MIW_138_AB_crude_Hept_EtOH_90_10_1mlmin_25C_1uL_6** | *Run Time (min):* | **47,72** |
| *Vial Number:* | **GA3** | *Report Template:* | **MIW_137_CD** |
| *Injection Volume:* | **1,00** | *Channel:* | **UV_VIS_1** |
| *Column:* | **IA-3** | *Wavelength:* | **220,0** |
| *Instrument Method:* | **Heptane_EtOH_90 _10_1mLmin_60min_25 °C** | *B %:* | **0,0** |
| *Processing Method:* | **Long alcohol** | *C %:* | **0,0** |
| *Injection Date/Time:* | **28.Apr.23 10:43** | *D %:* | **10,0** |
| *Operator:* | **Irena Jelenkovic - Didic** | | |

**Chromatogram**

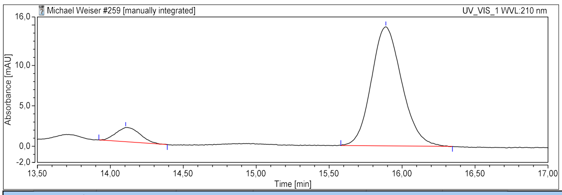


**Integration Results**

| No. | Peak Name | Retention Time min | Area mAU*min | Height mAU | Relative Area % | Relative Height % | Amount n.a. |
|---|---|---|---|---|---|---|---|
| 1 | | 14,105 | 0,338 | 1,761 | 8,65 | 10,68 | n.a. |
| 2 | | 15,888 | 3,572 | 14,733 | 91,35 | 89,32 | n.a. |
| **Total:** | | | **3,911** | **16,494** | **100,00** | **100,00** | |

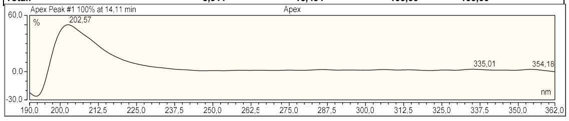


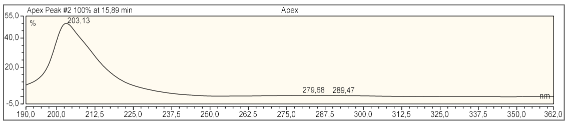


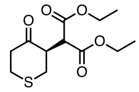

***R*-P6** using **PhQn**

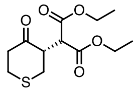

***S*-P6** using **PhQd**

## Chromatogram and Results

| | | | |
|---|---|---|---|
| *Injection Name:* | **MIW_133_crude_Hept_EtOH_90_10_1mlmin_25C_1uL_60mi** | *Run Time (min):* | **21,72** |
| *Vial Number:* | **GA1** | *Report Template:* | **MIW_137_CD** |
| *Injection Volume:* | **1,00** | *Channel:* | **UV_VIS_3** |
| *Column:* | **IA-3** | *Wavelength:* | **220,0** |
| *Instrument Method:* | **Heptane_EtOH_90 _10_1mLmin_60min_25 °C** | *B %:* | **0,0** |
| *Processing Method:* | **Long alcohol** | *C %:* | **0,0** |
| *Injection Date/Time:* | **20.Apr.23 11:35** | *D %:* | **10,0** |
| *Operator:* | **Irena Jelenkovic - Didic** | | |

**Chromatogram**

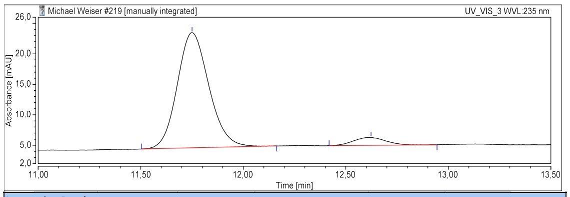


**Integration Results**

| No. | Peak Name | Retention Time min | Area mAU*min | Height mAU | Relative Area % | Relative Height % | Amount n.a. |
|---|---|---|---|---|---|---|---|
| 1 | | 11,750 | 3,374 | 18,890 | 93,50 | 93,63 | n.a. |
| 2 | | 12,623 | 0,235 | 1,286 | 6,50 | 6,37 | n.a. |
| **Total:** | | | **3,609** | **20,176** | **100,00** | **100,00** | |

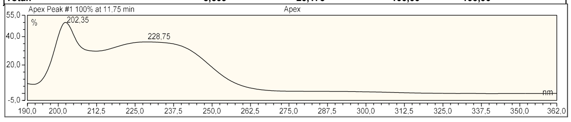


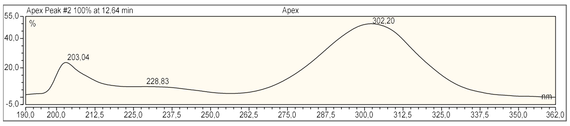


## Chromatogram and Results

| | | | |
|---|---|---|---|
| *Injection Name:* | **MIW_135_CD_crude_Hept_EtOH_90_10_1mlmin_25C_1uL_6** | *Run Time (min):* | **22,85** |
| *Vial Number:* | **GA2** | *Report Template:* | **MIW_137_CD** |
| *Injection Volume:* | **1,00** | *Channel:* | **UV_VIS_3** |
| *Column:* | **IA-3** | *Wavelength:* | **220,0** |
| *Instrument Method:* | **Heptane_EtOH_90 _10_1mLmin_60min_25 °C** | *B %:* | **0,0** |
| *Processing Method:* | **Long alcohol** | *C %:* | **0,0** |
| *Injection Date/Time:* | **25.Apr.23 11:58** | *D %:* | **10,0** |
| *Operator:* | **Irena Jelenkovic - Didic** | | |

**Chromatogram**

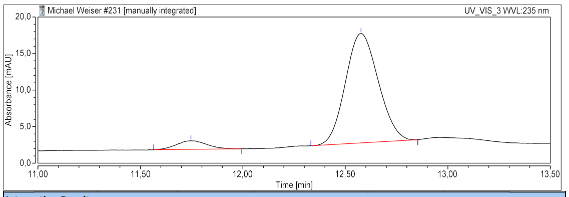


**Integration Results**

| No. | Peak Name | Retention Time min | Area mAU*min | Height mAU | Relative Area % | Relative Height % | Amount n.a. |
|---|---|---|---|---|---|---|---|
| 1 | | 11,747 | 0,200 | 1,168 | 6,71 | 7,23 | n.a. |
| 2 | | 12,577 | 2,785 | 14,983 | 93,29 | 92,77 | n.a. |
| **Total:** | | | **2,985** | **16,151** | **100,00** | **100,00** | |

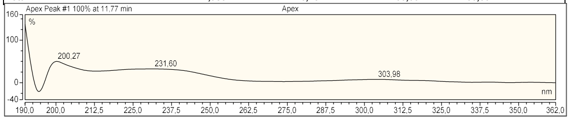


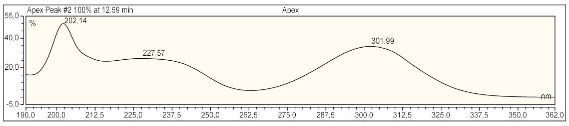

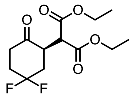


***R*-P7** using **PhQn**

## Chromatogram and Results

| | | | |
|---|---|---|---|
| *Injection Name:* | **MIW_183_CD_crude_Hept_EtOH_97_3_1mlmin_25C_1uL_60** | *Run Time (min):* | **18,05** |
| *Vial Number:* | **GA4** | *Report Template:* | **MIW_137_CD** |
| *Injection Volume:* | **10,00** | *Channel:* | **UV_VIS_1** |
| *Column:* | **IA-3** | *Wavelength:* | **220,0** |
| *Instrument Method:* | **Heptane_EtOH_97 _3_1mLmin_60min_25 °C** | *B %:* | **0,0** |
| *Processing Method:* | **Long alcohol** | *C %:* | **0,0** |
| *Injection Date/Time:* | **23.Jun.23 14:39** | *D %:* | **3,0** |
| *Operator:* | **Irena Jelenkovic - Didic** | | |

**Chromatogram**

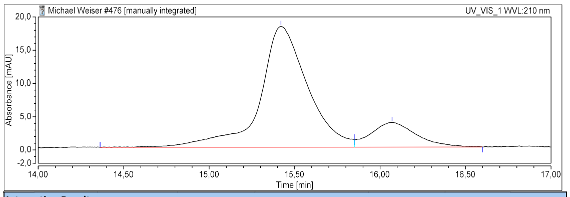


**Integration Results**

| No. | Peak Name | Retention Time min | Area mAU*min | Height mAU | Relative Area % | Relative Height % | Amount n.a. |
|---|---|---|---|---|---|---|---|
| 1 | | 15,420 | 5,887 | 18,189 | 83,81 | 83,09 | n.a. |
| 2 | | 16,070 | 1,137 | 3,702 | 16,19 | 16,91 | n.a. |
| **Total:** | | | **7,024** | **21,891** | **100,00** | **100,00** | |

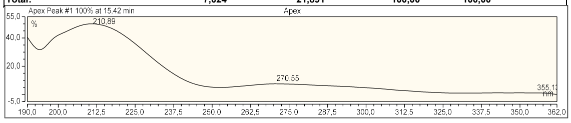


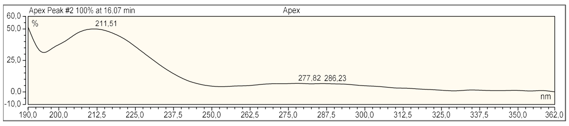


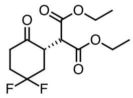


***S*-P7** using **PhQd**

## Chromatogram and Results

| | | | |
|---|---|---|---|
| *Injection Name:* | **MIW_188_C_Hept_EtOH_97_3_1mlmin_25C_1uL_60min_IA-3** | *Run Time (min):* | **22,95** |
| *Vial Number:* | **GA3** | *Report Template:* | **MIW_137_CD** |
| *Injection Volume:* | **10,00** | *Channel:* | **UV_VIS_1** |
| *Column:* | **IA-3** | *Wavelength:* | **220,0** |
| *Instrument Method:* | **Heptane_EtOH_97 _3_1mLmin_60min_25 °C** | *B %:* | **0,0** |
| *Processing Method:* | **Long alcohol** | *C %:* | **0,0** |
| *Injection Date/Time:* | **01.Aug.23 10:57** | *D %:* | **3,0** |
| *Operator:* | **Irena Jelenkovic - Didic** | | |

**Chromatogram**

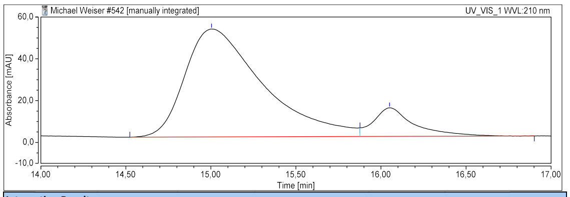


**Integration Results**

| No. | Peak Name | Retention Time min | Area mAU*min | Height mAU | Relative Area % | Relative Height % | Amount n.a. |
|---|---|---|---|---|---|---|---|
| 1 | | 15,003 | 27,425 | 51,606 | 87,93 | 79,08 | n.a. |
| 2 | | 16,050 | 3,765 | 13,649 | 12,07 | 20,92 | n.a. |
| **Total:** | | | **31,190** | **65,255** | **100,00** | **100,00** | |

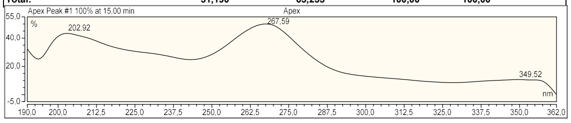


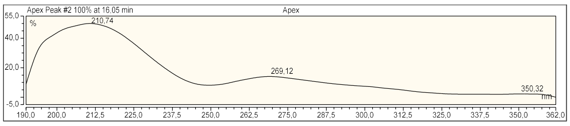


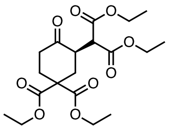

***R*-P8** using **PhQn**

## Chromatogram and Results

| | | | |
|---|---|---|---|
| *Injection Name:* | **MIW_194_A_Hept_EtOH_98_2_1mlmin_25C_1uL_60min_IA-3** | *Run Time (min):* | **20,05** |
| *Vial Number:* | **GA1** | *Report Template:* | **MIW_137_CD** |
| *Injection Volume:* | **10,00** | *Channel:* | **UV_VIS_1** |
| *Column:* | **IA-3** | *Wavelength:* | **220,0** |
| *Instrument Method:* | **Heptane_EtOH_97 _3_1mLmin_60min_25 °C** | *B %:* | **0,0** |
| *Processing Method:* | **Long alcohol** | *C %:* | **0,0** |
| *Injection Date/Time:* | **22.Aug.23 13:25** | *D %:* | **3,0** |
| *Operator:* | **Irena Jelenkovic - Didic** | | |

**Chromatogram**

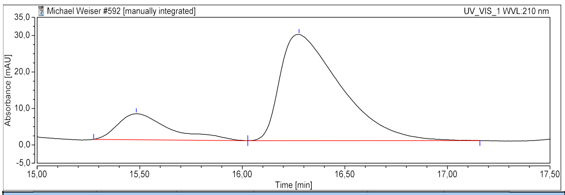


**Integration Results**

| No. | Peak Name | Retention Time min | Area mAU*min | Height mAU | Relative Area % | Relative Height % | Amount n.a. |
|---|---|---|---|---|---|---|---|
| 1 | | 15,483 | 2,103 | 7,132 | 17,46 | 19,68 | n.a. |
| 2 | | 16,277 | 9,942 | 29,106 | 82,54 | 80,32 | n.a. |
| **Total:** | | | **12,045** | **36,239** | **100,00** | **100,00** | |

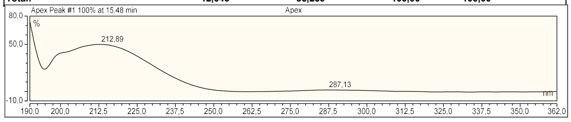


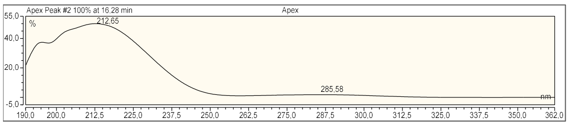


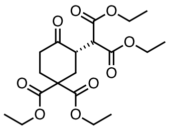

***S*-P8** using **PhQd**

## Chromatogram and Results

| | | | |
|---|---|---|---|
| *Injection Name:* | **MIW_194_B_Hept_EtOH_98_2_1mlmin_25C_1uL_60min_IA-3** | *Run Time (min):* | **20,06** |
| *Vial Number:* | **GA2** | *Report Template:* | **MIW_137_CD** |
| *Injection Volume:* | **10,00** | *Channel:* | **UV_VIS_1** |
| *Column:* | **IA-3** | *Wavelength:* | **220,0** |
| *Instrument Method:* | **Heptane_EtOH_97 _3_1mLmin_60min_25 °C** | *B %:* | **0,0** |
| *Processing Method:* | **Long alcohol** | *C %:* | **0,0** |
| *Injection Date/Time:* | **22.Aug.23 13:47** | *D %:* | **3,0** |
| *Operator:* | **Irena Jelenkovic - Didic** | | |

**Chromatogram**

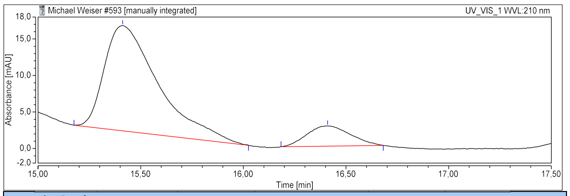


**Integration Results**

| No. | Peak Name | Retention Time min | Area mAU*min | Height mAU | Relative Area % | Relative Height % | Amount n.a. |
|---|---|---|---|---|---|---|---|
| 1 | | 15,412 | 4,390 | 14,422 | 86,62 | 83,86 | n.a. |
| 2 | | 16,410 | 0,678 | 2,775 | 13,38 | 16,14 | n.a. |
| **Total:** | | | **5,068** | **17,197** | **100,00** | **100,00** | |

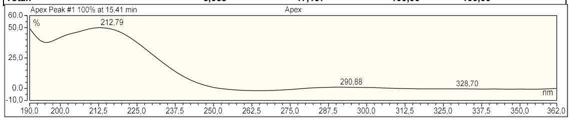


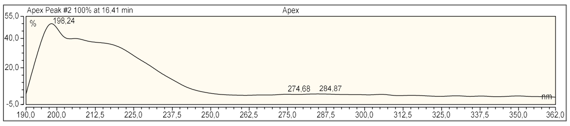

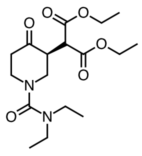

***R*-P9** using **PhQn**

### Chromatogram and Results

| | | | |
|---|---|---|---|
| *Injection Name:* | **MIW_193_AB_crude_Hept_EtOH_98_2_1mlmin_25C_1uL_60** | *Run Time (min):* | **72,15** |
| *Vial Number:* | **GA1** | *Report Template:* | **MIW_137_CD** |
| *Injection Volume:* | **10,00** | *Channel:* | **UV_VIS_1** |
| *Column:* | **IA-3** | *Wavelength:* | **220,0** |
| *Instrument Method:* | **Heptane_EtOH_98 _2_1mLmin_90min_25 °C** | *B %:* | **0,0** |
| *Processing Method:* | **Long alcohol** | *C %:* | **0,0** |
| *Injection Date/Time:* | **18.Aug.23 09:31** | *D %:* | **2,0** |
| *Operator:* | **Irena Jelenkovic - Didic** | | |

**Chromatogram**

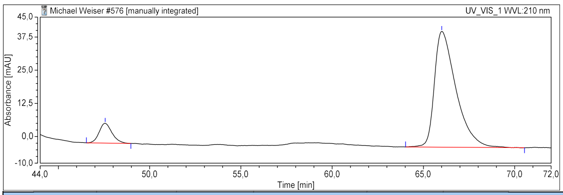


**Integration Results**

| No. | Peak Name | Retention Time min | Area mAU*min | Height mAU | Relative Area % | Relative Height % | Amount n.a. |
|---|---|---|---|---|---|---|---|
| 1 | | 47,565 | 6,104 | 7,461 | 9,28 | 14,61 | n.a. |
| 2 | | 66,005 | 59,694 | 43,600 | 90,72 | 85,39 | n.a. |
| **Total:** | | | **65,798** | **51,061** | **100,00** | **100,00** | |

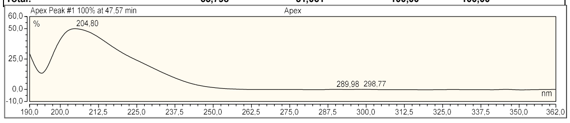


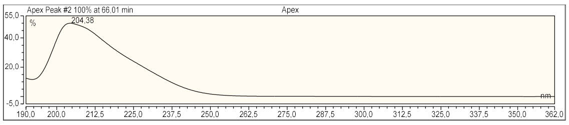


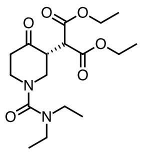

***S*-P9** using **PhQd**

### Chromatogram and Results

| | | | |
|---|---|---|---|
| *Injection Name:* | **MIW_193_CD_crude_Hept_EtOH_98_2_1mlmin_25C_1uL_60** | *Run Time (min):* | **77,37** |
| *Vial Number:* | **GA2** | *Report Template:* | **MIW_137_CD** |
| *Injection Volume:* | **10,00** | *Channel:* | **UV_VIS_1** |
| *Column:* | **IA-3** | *Wavelength:* | **220,0** |
| *Instrument Method:* | **Heptane_EtOH_98 _2_1mLmin_90min_25 °C** | *B %:* | **0,0** |
| *Processing Method:* | **Long alcohol** | *C %:* | **0,0** |
| *Injection Date/Time:* | **18.Aug.23 10:45** | *D %:* | **2,0** |
| *Operator:* | **Irena Jelenkovic - Didic** | | |

**Chromatogram**

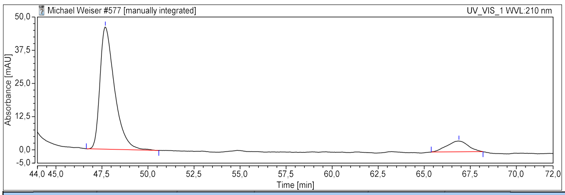


**Integration Results**

| No. | Peak Name | Retention Time min | Area mAU*min | Height mAU | Relative Area % | Relative Height % | Amount n.a. |
|---|---|---|---|---|---|---|---|
| 1 | | 47,683 | 42,243 | 46,005 | 88,61 | 91,88 | n.a. |
| 2 | | 66,883 | 5,430 | 4,064 | 11,39 | 8,12 | n.a. |
| **Total:** | | | **47,673** | **50,069** | **100,00** | **100,00** | |

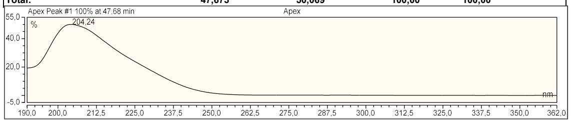


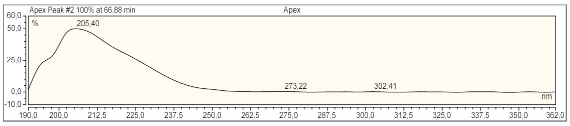


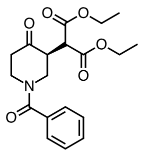

***R*-P10** using **PhQn**

### Chromatogram and Results

| | | | |
|---|---|---|---|
| *Injection Name:* | **MIW_214_AB_crude_Hept_EtOH_87_13_1mlmin_25C_1uL_6** | *Run Time (min):* | **60,00** |
| *Vial Number:* | **GB3** | *Report Template:* | **MIW_137_CD** |
| *Injection Volume:* | **5,00** | *Channel:* | **UV_VIS_1** |
| *Column:* | **IA-3** | *Wavelength:* | **220,0** |
| *Instrument Method:* | **Heptane_EtOH_87 _13_1mLmin_60min_25 °C** | *B %:* | **0,0** |
| *Processing Method:* | **Long alcohol** | *C %:* | **0,0** |
| *Injection Date/Time:* | **18.Sep.23 13:20** | *D %:* | **13,0** |
| *Operator:* | **Irena Jelenkovic - Didic** | | |

**Chromatogram**

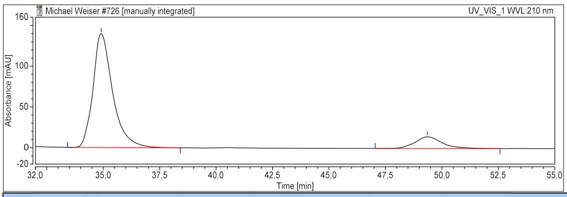


**Integration Results**

| No. | Peak Name | Retention Time min | Area mAU*min | Height mAU | Relative Area % | Relative Height % | Amount n.a. |
|---|---|---|---|---|---|---|---|
| 1 | | 34,910 | 140,735 | 139,508 | 87,63 | 90,69 | n.a. |
| 2 | | 49,355 | 19,861 | 14,320 | 12,37 | 9,31 | n.a. |
| **Total:** | | | **160,596** | **153,828** | **100,00** | **100,00** | |

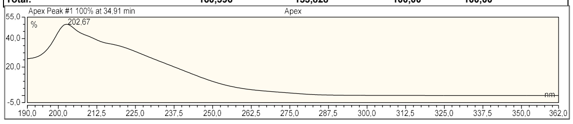


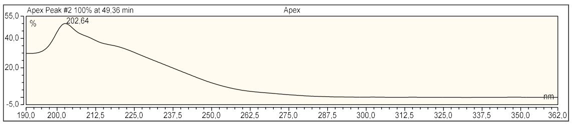


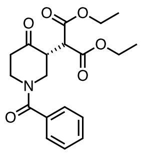

***S*-P10** using **PhQd**

### Chromatogram and Results

| | | | |
|---|---|---|---|
| *Injection Name:* | **MIW_214_CD_crude_Hept_EtOH_87_13_1mlmin_25C_1uL_6** | *Run Time (min):* | **60,00** |
| *Vial Number:* | **GB4** | *Report Template:* | **MIW_137_CD** |
| *Injection Volume:* | **5,00** | *Channel:* | **UV_VIS_1** |
| *Column:* | **IA-3** | *Wavelength:* | **220,0** |
| *Instrument Method:* | **Heptane_EtOH_87 _13_1mLmin_60min_25 °C** | *B %:* | **0,0** |
| *Processing Method:* | **Long alcohol** | *C %:* | **0,0** |
| *Injection Date/Time:* | **18.Sep.23 14:21** | *D %:* | **13,0** |
| *Operator:* | **Irena Jelenkovic - Didic** | | |

**Chromatogram**

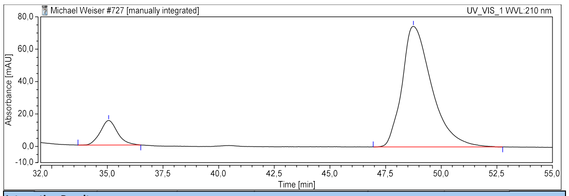


**Integration Results**

| No. | Peak Name | Retention Time min | Area mAU*min | Height mAU | Relative Area % | Relative Height % | Amount n.a. |
|---|---|---|---|---|---|---|---|
| 1 | | 35,055 | 13,599 | 15,135 | 10,58 | 16,90 | n.a. |
| 2 | | 48,748 | 114,873 | 74,417 | 89,42 | 83,10 | n.a. |
| **Total:** | | | **128,472** | **89,551** | **100,00** | **100,00** | |

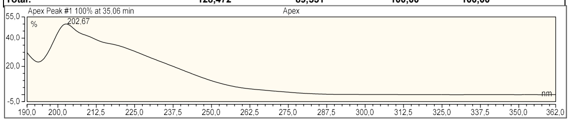


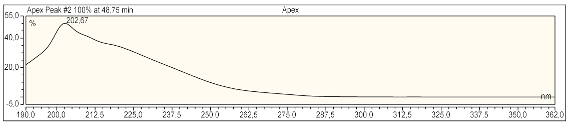

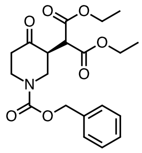

***R*-P11** using **PhQn**

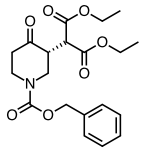

***S*-P11** using **PhQd**

## Chromatogram and Results (*R*-P11)

| | | | |
|---|---|---|---|
| *Injection Name:* | **MIW_203_AB_crude_Hept_EtOH_95_5_1mlmin_25C_1uL_60** | *Run Time (min):* | **27,06** |
| *Vial Number:* | **GA5** | *Report Template:* | **MIW_137_CD** |
| *Injection Volume:* | **2,00** | *Channel:* | **UV_VIS_1** |
| *Column:* | **IA-3** | *Wavelength:* | **220,0** |
| *Instrument Method:* | **Heptane_EtOH_85_15 _1mLmin_25 °C_60min** | *B %:* | **0,0** |
| *Processing Method:* | **Long alcohol** | *C %:* | **0,0** |
| *Injection Date/Time:* | **01.Sep.23 14:40** | *D %:* | **15,0** |
| *Operator:* | **Irena Jelenkovic - Didic** | | |

**Chromatogram**

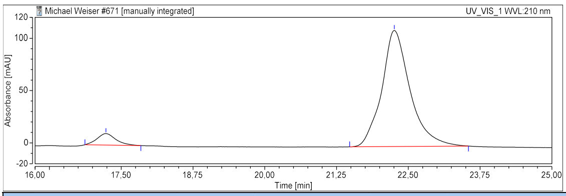


**Integration Results**

| No. | Peak Name | Retention Time min | Area mAU*min | Height mAU | Relative Area % | Relative Height % | Amount n.a. |
|---|---|---|---|---|---|---|---|
| 1 | | 17,235 | 4,160 | 10,955 | 6,15 | 8,98 | n.a. |
| 2 | | 22,255 | 63,492 | 110,985 | 93,85 | 91,02 | n.a. |
| **Total:** | | | **67,652** | **121,940** | **100,00** | **100,00** | |

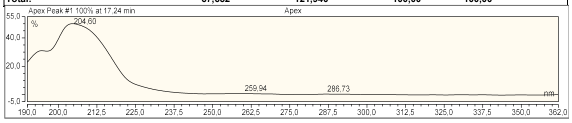


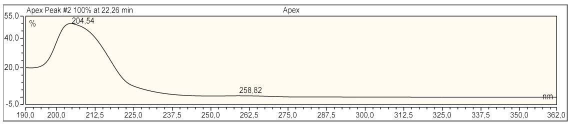


## Chromatogram and Results (*S*-P11)

| | | | |
|---|---|---|---|
| *Injection Name:* | **MIW_203_CD_crude_Hept_EtOH_85_15_1mlmin_25C_1uL_6** | *Run Time (min):* | **26,42** |
| *Vial Number:* | **GA6** | *Report Template:* | **MIW_137_CD** |
| *Injection Volume:* | **2,00** | *Channel:* | **UV_VIS_1** |
| *Column:* | **IA-3** | *Wavelength:* | **220,0** |
| *Instrument Method:* | **Heptane_EtOH_85_15 _1mLmin_25 °C_60min** | *B %:* | **0,0** |
| *Processing Method:* | **Long alcohol** | *C %:* | **0,0** |
| *Injection Date/Time:* | **01.Sep.23 16:08** | *D %:* | **15,0** |
| *Operator:* | **Irena Jelenkovic - Didic** | | |

**Chromatogram**

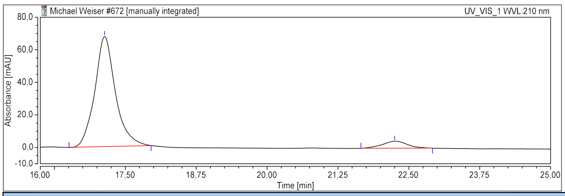


**Integration Results**

| No. | Peak Name | Retention Time min | Area mAU*min | Height mAU | Relative Area % | Relative Height % | Amount n.a. |
|---|---|---|---|---|---|---|---|
| 1 | | 17,135 | 28,528 | 67,640 | 92,86 | 93,94 | n.a. |
| 2 | | 22,248 | 2,193 | 4,362 | 7,14 | 6,06 | n.a. |
| **Total:** | | | **30,721** | **72,003** | **100,00** | **100,00** | |

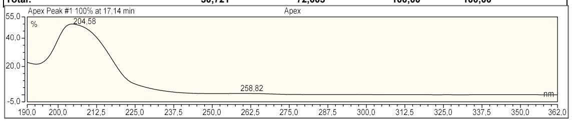


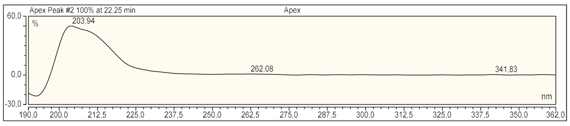


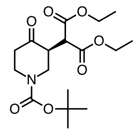

***R*-P12** using **PhQn**

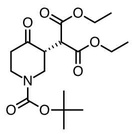

***S*-P12** using **PhQd**

## Chromatogram and Results (*R*-P12)

| | | | |
|---|---|---|---|
| *Injection Name:* | **MIW_144_AB_crude_Hept_EtOH_90_10_1mlmin_25C_1uL_6** | *Run Time (min):* | **34,09** |
| *Vial Number:* | **GA6** | *Report Template:* | **MIW_137_CD** |
| *Injection Volume:* | **1,00** | *Channel:* | **UV_VIS_1** |
| *Column:* | **IA-3** | *Wavelength:* | **220,0** |
| *Instrument Method:* | **Heptane_EtOH_90 _10_1mLmin_60min_25 °C** | *B %:* | **0,0** |
| *Processing Method:* | **Long alcohol** | *C %:* | **0,0** |
| *Injection Date/Time:* | **11.Mai.23 13:22** | *D %:* | **10,0** |
| *Operator:* | **Irena Jelenkovic - Didic** | | |

**Chromatogram**

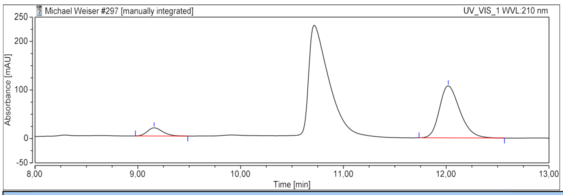


**Integration Results**

| No. | Peak Name | Retention Time min | Area mAU*min | Height mAU | Relative Area % | Relative Height % | Amount n.a. |
|---|---|---|---|---|---|---|---|
| 1 | | 9,158 | 2,894 | 16,744 | 10,49 | 13,53 | n.a. |
| 2 | | 12,020 | 24,700 | 107,031 | 89,51 | 86,47 | n.a. |
| **Total:** | | | **27,595** | **123,775** | **100,00** | **100,00** | |

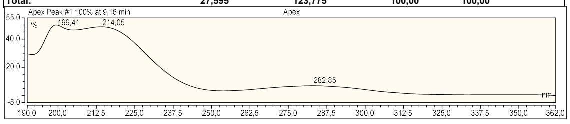


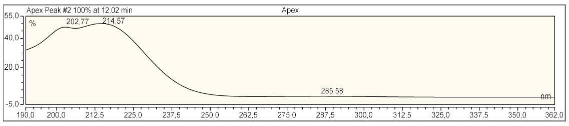


## Chromatogram and Results (*S*-P12)

| | | | |
|---|---|---|---|
| *Injection Name:* | **MIW_137_CD_crude_Hept_EtOH_90_10_1mlmin_25C_1uL_6** | *Run Time (min):* | **20,79** |
| *Vial Number:* | **GA4** | *Report Template:* | **MIW_137_CD** |
| *Injection Volume:* | **1,00** | *Channel:* | **UV_VIS_1** |
| *Column:* | **IA-3** | *Wavelength:* | **220,0** |
| *Instrument Method:* | **Heptane_EtOH_90 _10_1mLmin_60min_25 °C** | *B %:* | **0,0** |
| *Processing Method:* | **Long alcohol** | *C %:* | **0,0** |
| *Injection Date/Time:* | **27.Apr.23 13:57** | *D %:* | **10,0** |
| *Operator:* | **Irena Jelenkovic - Didic** | | |

**Chromatogram**

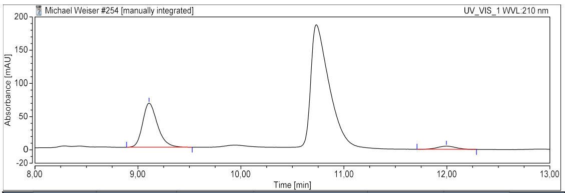


**Integration Results**

| No. | Peak Name | Retention Time min | Area mAU*min | Height mAU | Relative Area % | Relative Height % | Amount n.a. |
|---|---|---|---|---|---|---|---|
| 1 | | 9,108 | 11,075 | 66,274 | 91,74 | 93,28 | n.a. |
| 2 | | 11,995 | 0,997 | 4,778 | 8,26 | 6,72 | n.a. |
| **Total:** | | | **12,072** | **71,052** | **100,00** | **100,00** | |

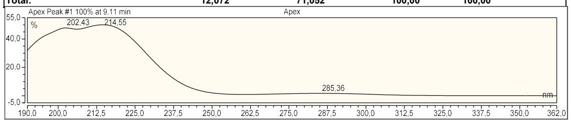


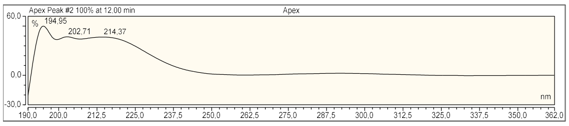

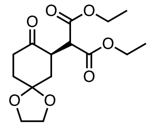

***R*-P13** using **PhQn**

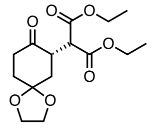

***S*-P13** using **PhQd**

### Chromatogram and Results

| | | | |
|---|---|---|---|
| *Injection Name:* | **MIW_142_CD_crude_Hept_EtOH_90_10_1mlmin_25C_1uL_6** | *Run Time (min):* | **34,94** |
| *Vial Number:* | **GA4** | *Report Template:* | **MIW_137_CD** |
| *Injection Volume:* | **1,00** | *Channel:* | **UV_VIS_1** |
| *Column:* | **IA-3** | *Wavelength:* | **220,0** |
| *Instrument Method:* | **Heptane_EtOH_90 _10_1mLmin_60min_25 °C** | *B %:* | **0,0** |
| *Processing Method:* | **Long alcohol** | *C %:* | **0,0** |
| *Injection Date/Time:* | **11.Mai.23 12:23** | *D %:* | **10,0** |
| *Operator:* | **Irena Jelenkovic - Didic** | | |

**Chromatogram**

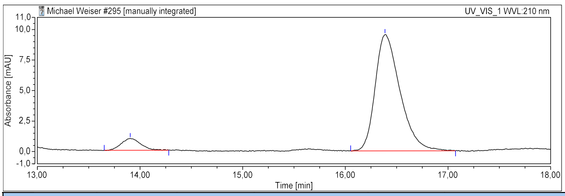


**Integration Results**

| No. | Peak Name | Retention Time min | Area mAU*min | Height mAU | Relative Area % | Relative Height % | Amount n.a. |
|---|---|---|---|---|---|---|---|
| 1 | | 13,905 | 0,212 | 0,968 | 7,35 | 9,21 | n.a. |
| 2 | | 16,387 | 2,675 | 9,539 | 92,65 | 90,79 | n.a. |
| **Total:** | | | **2,887** | **10,508** | **100,00** | **100,00** | |

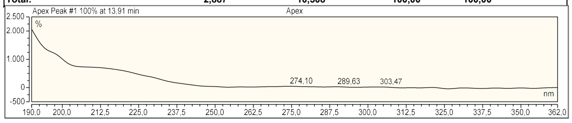


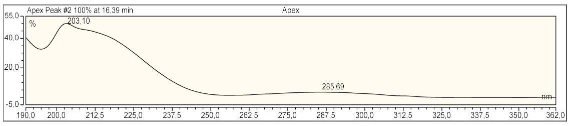


### Chromatogram and Results

| | | | |
|---|---|---|---|
| *Injection Name:* | **MIW_138_CD_crude_Hept_EtOH_90_10_1mlmin_25C_1uL_6** | *Run Time (min):* | **31,12** |
| *Vial Number:* | **GA4** | *Report Template:* | **MIW_137_CD** |
| *Injection Volume:* | **1,00** | *Channel:* | **UV_VIS_1** |
| *Column:* | **IA-3** | *Wavelength:* | **220,0** |
| *Instrument Method:* | **Heptane_EtOH_90 _10_1mLmin_60min_25 °C** | *B %:* | **0,0** |
| *Processing Method:* | **Long alcohol** | *C %:* | **0,0** |
| *Injection Date/Time:* | **28.Apr.23 11:33** | *D %:* | **10,0** |
| *Operator:* | **Irena Jelenkovic - Didic** | | |

**Chromatogram**

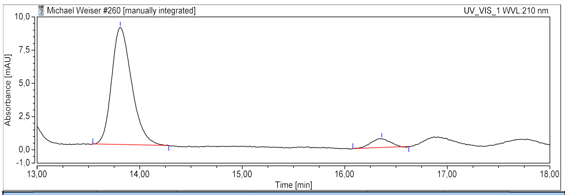


**Integration Results**

| No. | Peak Name | Retention Time min | Area mAU*min | Height mAU | Relative Area % | Relative Height % | Amount n.a. |
|---|---|---|---|---|---|---|---|
| 1 | | 13,810 | 1,995 | 8,815 | 93,29 | 92,91 | n.a. |
| 2 | | 16,360 | 0,143 | 0,673 | 6,71 | 7,09 | n.a. |
| **Total:** | | | **2,138** | **9,488** | **100,00** | **100,00** | |

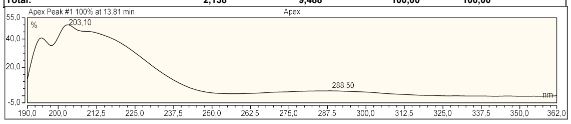


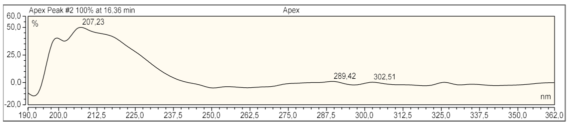


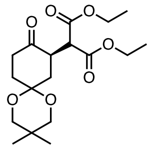

***R*-P14** using **PhQn**

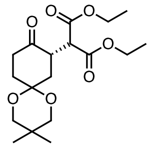

***S*-P14** using **PhQd**

### Chromatogram and Results

| | | | |
|---|---|---|---|
| *Injection Name:* | **MIW_196_AB_crude_Hept_EtOH_99_1_1mlmin_25C_1uL_60** | *Run Time (min):* | **42,56** |
| *Vial Number:* | **GA3** | *Report Template:* | **MIW_137_CD** |
| *Injection Volume:* | **50,00** | *Channel:* | **UV_VIS_1** |
| *Column:* | **IA-3** | *Wavelength:* | **254,0** |
| *Instrument Method:* | **Heptane_EtOH_99 _1_1mLmin_60min_25 °C** | *B %:* | **0,0** |
| *Processing Method:* | **Long alcohol** | *C %:* | **0,0** |
| *Injection Date/Time:* | **24.Aug.23 11:48** | *D %:* | **1,0** |
| *Operator:* | **Irena Jelenkovic - Didic** | | |

**Chromatogram**

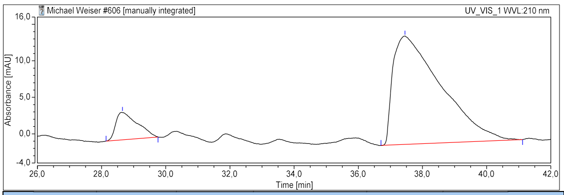


**Integration Results**

| No. | Peak Name | Retention Time min | Area mAU*min | Height mAU | Relative Area % | Relative Height % | Amount n.a. |
|---|---|---|---|---|---|---|---|
| 1 | | 28,648 | 2,936 | 3,782 | 10,21 | 20,29 | n.a. |
| 2 | | 37,455 | 25,813 | 14,854 | 89,79 | 79,71 | n.a. |
| **Total:** | | | **28,749** | **18,636** | **100,00** | **100,00** | |

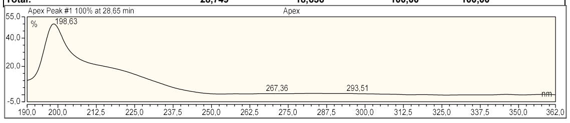


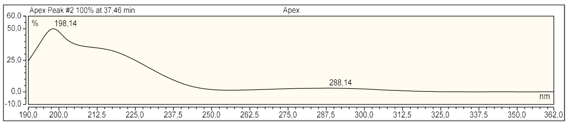


### Chromatogram and Results

| | | | |
|---|---|---|---|
| *Injection Name:* | **MIW_196_CD_Hept_EtOH_99_1_1mlmin_25C_1uL_60min_IA** | *Run Time (min):* | **44,14** |
| *Vial Number:* | **GA2** | *Report Template:* | **MIW_137_CD** |
| *Injection Volume:* | **10,00** | *Channel:* | **UV_VIS_1** |
| *Column:* | **IA-3** | *Wavelength:* | **254,0** |
| *Instrument Method:* | **Heptane_EtOH_99 _1_1mLmin_60min_25 °C** | *B %:* | **0,0** |
| *Processing Method:* | **Long alcohol** | *C %:* | **0,0** |
| *Injection Date/Time:* | **24.Aug.23 14:56** | *D %:* | **1,0** |
| *Operator:* | **Irena Jelenkovic - Didic** | | |

**Chromatogram**

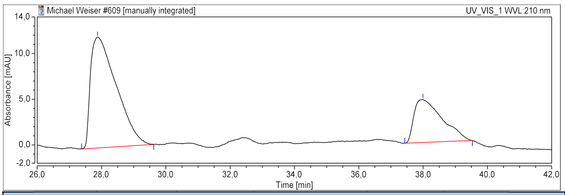


**Integration Results**

| No. | Peak Name | Retention Time min | Area mAU*min | Height mAU | Relative Area % | Relative Height % | Amount n.a. |
|---|---|---|---|---|---|---|---|
| 1 | | 27,872 | 11,260 | 12,135 | 70,25 | 71,97 | n.a. |
| 2 | | 37,997 | 4,768 | 4,725 | 29,75 | 28,03 | n.a. |
| **Total:** | | | **16,028** | **16,861** | **100,00** | **100,00** | |

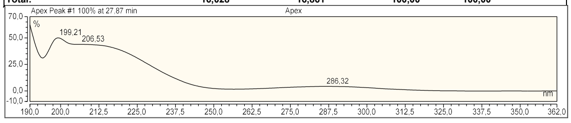


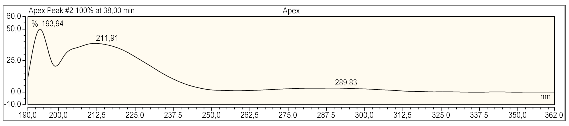

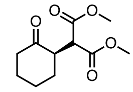

***R*-P15** using **PhQn**

### Chromatogram and Results

| | | | |
|---|---|---|---|
| *Injection Name:* | **MIW_140_CD_Hept_EtOH_96_4_1mlmin_25C_1uL_60min_IA** | *Run Time (min):* | **30,55** |
| *Vial Number:* | **GA1** | *Report Template:* | **MIW_137_CD** |
| *Injection Volume:* | **10,00** | *Channel:* | **UV_VIS_1** |
| *Column:* | **IA-3** | *Wavelength:* | **220,0** |
| *Instrument Method:* | **Heptane_EtOH_96 _4_1mLmin_60min_25 °C** | *B %:* | **0,0** |
| *Processing Method:* | **Long alcohol** | *C %:* | **0,0** |
| *Injection Date/Time:* | **30.Aug.23 14:32** | *D %:* | **4,0** |
| *Operator:* | **Irena Jelenkovic - Didic** | | |

**Chromatogram**

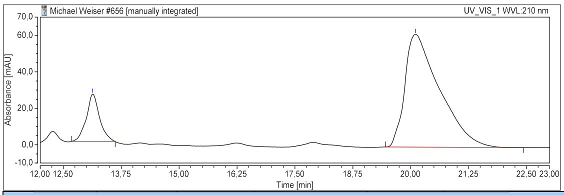


**Integration Results**

| No. | Peak Name | Retention Time min | Area mAU*min | Height mAU | Relative Area % | Relative Height % | Amount n.a. |
|---|---|---|---|---|---|---|---|
| 1 | | 13,133 | 8,640 | 26,036 | 13,89 | 29,63 | n.a. |
| 2 | | 20,102 | 53,575 | 61,827 | 86,11 | 70,37 | n.a. |
| **Total:** | | | **62,216** | **87,863** | **100,00** | **100,00** | |

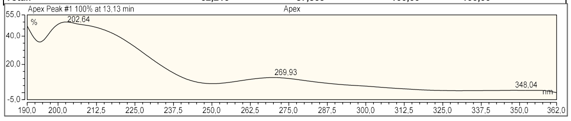


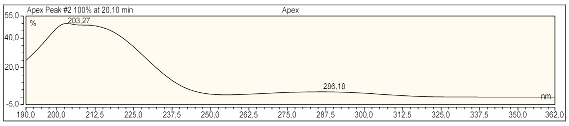


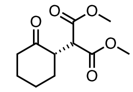

***S*-P15** using **PhQd**

### Chromatogram and Results

| | | | |
|---|---|---|---|
| *Injection Name:* | **MIW_197_Hept_EtOH_96_4_1mlmin_25C_1uL_60min_IA-3** | *Run Time (min):* | **29,92** |
| *Vial Number:* | **GA2** | *Report Template:* | **MIW_137_CD** |
| *Injection Volume:* | **10,00** | *Channel:* | **UV_VIS_1** |
| *Column:* | **IA-3** | *Wavelength:* | **220,0** |
| *Instrument Method:* | **Heptane_EtOH_96 _4_1mLmin_60min_25 °C** | *B %:* | **0,0** |
| *Processing Method:* | **Long alcohol** | *C %:* | **0,0** |
| *Injection Date/Time:* | **30.Aug.23 15:04** | *D %:* | **4,0** |
| *Operator:* | **Irena Jelenkovic - Didic** | | |

**Chromatogram**

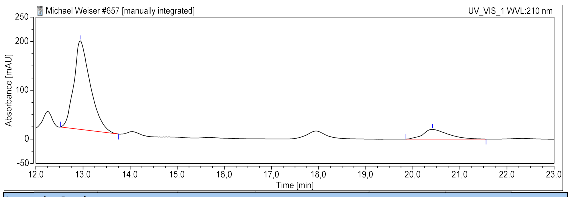


**Integration Results**

| No. | Peak Name | Retention Time min | Area mAU*min | Height mAU | Relative Area % | Relative Height % | Amount n.a. |
|---|---|---|---|---|---|---|---|
| 1 | | 12,940 | 73,800 | 181,572 | 86,03 | 90,16 | n.a. |
| 2 | | 20,418 | 11,989 | 19,821 | 13,97 | 9,84 | n.a. |
| **Total:** | | | **85,789** | **201,394** | **100,00** | **100,00** | |

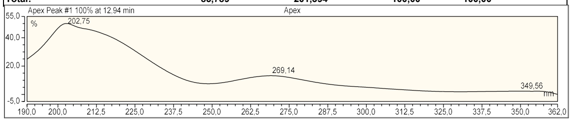


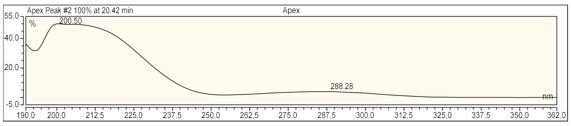


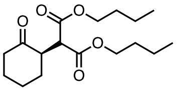

***R*-P16** using **PhQn**

### Chromatogram and Results

| | | | |
|---|---|---|---|
| *Injection Name:* | **MIW_212_AB_Hept_EtOH_98_2_1mlmin_25C_1uL_60min_IA** | *Run Time (min):* | **26,01** |
| *Vial Number:* | **GB1** | *Report Template:* | **MIW_137_CD** |
| *Injection Volume:* | **5,00** | *Channel:* | **UV_VIS_1** |
| *Column:* | **IA-3** | *Wavelength:* | **220,0** |
| *Instrument Method:* | **Heptane_EtOH_98 _2_1mLmin_60min_25 °C** | *B %:* | **0,0** |
| *Processing Method:* | **Long alcohol** | *C %:* | **0,0** |
| *Injection Date/Time:* | **15.Sep.23 10:52** | *D %:* | **2,0** |
| *Operator:* | **Irena Jelenkovic - Didic** | | |

**Chromatogram**

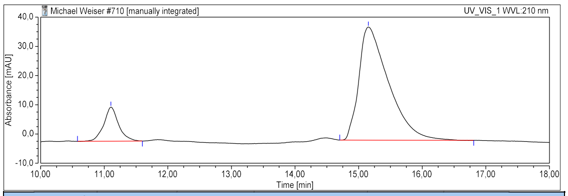


**Integration Results**

| No. | Peak Name | Retention Time min | Area mAU*min | Height mAU | Relative Area % | Relative Height % | Amount n.a. |
|---|---|---|---|---|---|---|---|
| 1 | | 11,105 | 3,168 | 11,695 | 13,10 | 23,20 | n.a. |
| 2 | | 15,152 | 21,013 | 38,716 | 86,90 | 76,80 | n.a. |
| **Total:** | | | **24,181** | **50,411** | **100,00** | **100,00** | |

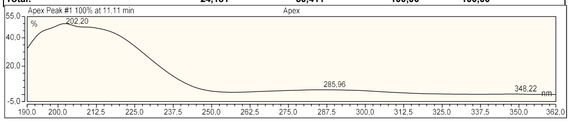


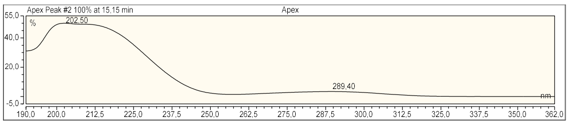


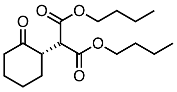

***S*-P16** using **PhQd**

### Chromatogram and Results

| | | | |
|---|---|---|---|
| *Injection Name:* | **MIW_212_CD_Hept_EtOH_98_2_1mlmin_25C_1uL_60min_IA** | *Run Time (min):* | **20,54** |
| *Vial Number:* | **GB2** | *Report Template:* | **MIW_137_CD** |
| *Injection Volume:* | **5,00** | *Channel:* | **UV_VIS_1** |
| *Column:* | **IA-3** | *Wavelength:* | **220,0** |
| *Instrument Method:* | **Heptane_EtOH_98 _2_1mLmin_60min_25 °C** | *B %:* | **0,0** |
| *Processing Method:* | **Long alcohol** | *C %:* | **0,0** |
| *Injection Date/Time:* | **15.Sep.23 11:20** | *D %:* | **2,0** |
| *Operator:* | **Irena Jelenkovic - Didic** | | |

**Chromatogram**

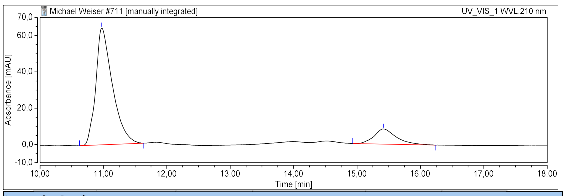


**Integration Results**

| No. | Peak Name | Retention Time min | Area mAU*min | Height mAU | Relative Area % | Relative Height % | Amount n.a. |
|---|---|---|---|---|---|---|---|
| 1 | | 10,973 | 19,592 | 64,349 | 85,34 | 88,56 | n.a. |
| 2 | | 15,420 | 3,365 | 8,313 | 14,66 | 11,44 | n.a. |
| **Total:** | | | **22,957** | **72,662** | **100,00** | **100,00** | |

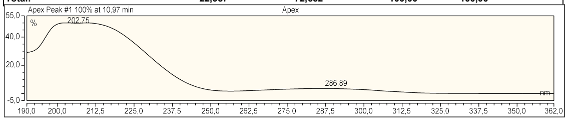


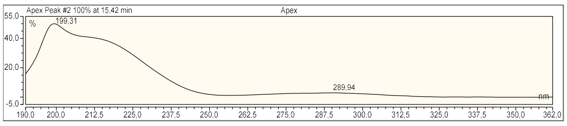

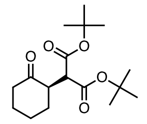

*R*-**P17** using **PhQn**

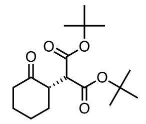

*S*-**P17** using **PhQd**

### Chromatogram and Results

| | | | |
|---|---|---|---|
| *Injection Name:* | **MIW_211_AB_Hept_EtOH_99_1_1mlmin_25C_1uL_60min_IA** | *Run Time (min):* | **18,51** |
| *Vial Number:* | **GA1** | *Report Template:* | **MIW_137_CD** |
| *Injection Volume:* | **5,00** | *Channel:* | **UV_VIS_1** |
| *Column:* | **IA-3** | *Wavelength:* | **220,0** |
| *Instrument Method:* | **Heptane_EtOH_99 _1_1mLmin_60min_25 °C** | *B %:* | **0,0** |
| *Processing Method:* | **Long alcohol** | *C %:* | **0,0** |
| *Injection Date/Time:* | **13.Sep.23 10:40** | *D %:* | **1,0** |
| *Operator:* | **Irena Jelenkovic - Didic** | | |

**Chromatogram**

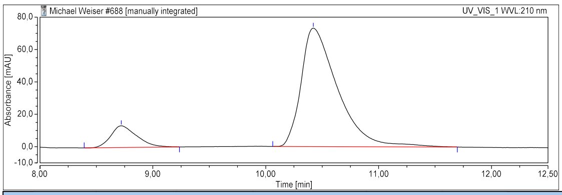


**Integration Results**

| No. | Peak Name | Retention Time min | Area mAU*min | Height mAU | Relative Area % | Relative Height % | Amount n.a. |
|---|---|---|---|---|---|---|---|
| 1 | | 8,720 | 3,562 | 13,401 | 11,50 | 15,48 | n.a. |
| 2 | | 10,422 | 27,399 | 73,143 | 88,50 | 84,52 | n.a. |
| **Total:** | | | **30,961** | **86,543** | **100,00** | **100,00** | |

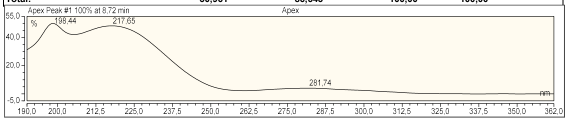


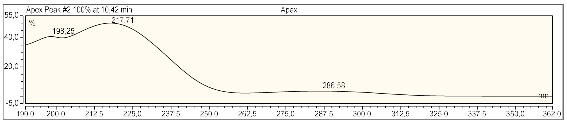


### Chromatogram and Results

| | | | |
|---|---|---|---|
| *Injection Name:* | **MIW_211_CD_Hept_EtOH_99_1_1mlmin_25C_1uL_60min_IA** | *Run Time (min):* | **15,95** |
| *Vial Number:* | **GA2** | *Report Template:* | **MIW_137_CD** |
| *Injection Volume:* | **5,00** | *Channel:* | **UV_VIS_1** |
| *Column:* | **IA-3** | *Wavelength:* | **220,0** |
| *Instrument Method:* | **Heptane_EtOH_99 _1_1mLmin_60min_25 °C** | *B %:* | **0,0** |
| *Processing Method:* | **Long alcohol** | *C %:* | **0,0** |
| *Injection Date/Time:* | **13.Sep.23 12:12** | *D %:* | **1,0** |
| *Operator:* | **Irena Jelenkovic - Didic** | | |

**Chromatogram**

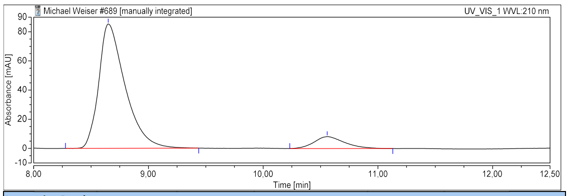


**Integration Results**

| No. | Peak Name | Retention Time min | Area mAU*min | Height mAU | Relative Area % | Relative Height % | Amount n.a. |
|---|---|---|---|---|---|---|---|
| 1 | | 8,650 | 22,712 | 85,272 | 90,05 | 91,27 | n.a. |
| 2 | | 10,558 | 2,508 | 8,157 | 9,95 | 8,73 | n.a. |
| **Total:** | | | **25,220** | **93,429** | **100,00** | **100,00** | |

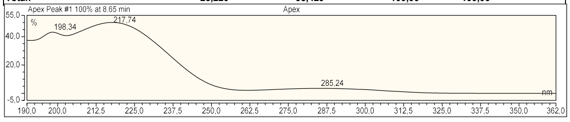


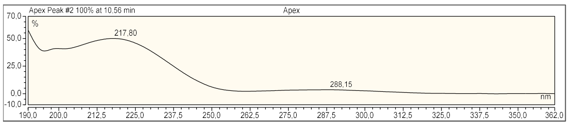


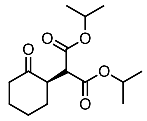

*R*-**P18** using **PhQn**

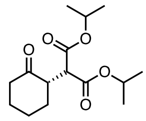

*S*-**P18** using **PhQd**

### Chromatogram and Results

| | | | |
|---|---|---|---|
| *Injection Name:* | **MIW_228_AB_Hept_EtOH_98_2_1mlmin_25C_1uL_60min_IA** | *Run Time (min):* | **15,35** |
| *Vial Number:* | **GA1** | *Report Template:* | **MIW_137_CD** |
| *Injection Volume:* | **10,00** | *Channel:* | **UV_VIS_2** |
| *Column:* | **IA-3** | *Wavelength:* | **220,0** |
| *Instrument Method:* | **Heptane_EtOH_98 _2_1mLmin_60min_25 °C** | *B %:* | **0,0** |
| *Processing Method:* | **Long alcohol** | *C %:* | **0,0** |
| *Injection Date/Time:* | **12.Okt.23 11:30** | *D %:* | **2,0** |
| *Operator:* | **Irena Jelenkovic - Didic** | | |

**Chromatogram**

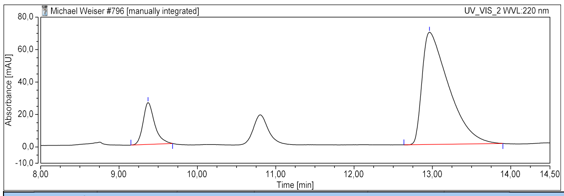


**Integration Results**

| No. | Peak Name | Retention Time min | Area mAU*min | Height mAU | Relative Area % | Relative Height % | Amount n.a. |
|---|---|---|---|---|---|---|---|
| 1 | | 9,370 | 4,404 | 25,779 | 13,90 | 27,15 | n.a. |
| 2 | | 12,963 | 27,286 | 69,174 | 86,10 | 72,85 | n.a. |
| **Total:** | | | **31,690** | **94,953** | **100,00** | **100,00** | |

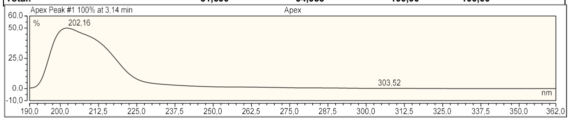


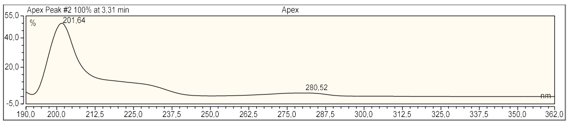


### Chromatogram and Results

| | | | |
|---|---|---|---|
| *Injection Name:* | **MIW_228_CD_Hept_EtOH_98_2_1mlmin_25C_1uL_60min_IA** | *Run Time (min):* | **21,63** |
| *Vial Number:* | **GA2** | *Report Template:* | **MIW_137_CD** |
| *Injection Volume:* | **10,00** | *Channel:* | **UV_VIS_2** |
| *Column:* | **IA-3** | *Wavelength:* | **220,0** |
| *Instrument Method:* | **Heptane_EtOH_98 _2_1mLmin_60min_25 °C** | *B %:* | **0,0** |
| *Processing Method:* | **Long alcohol** | *C %:* | **0,0** |
| *Injection Date/Time:* | **11.Okt.23 17:00** | *D %:* | **2,0** |
| *Operator:* | **Irena Jelenkovic - Didic** | | |

**Chromatogram**

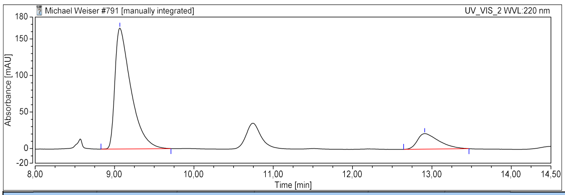


**Integration Results**

| No. | Peak Name | Retention Time min | Area mAU*min | Height mAU | Relative Area % | Relative Height % | Amount n.a. |
|---|---|---|---|---|---|---|---|
| 1 | | 9,067 | 38,645 | 165,404 | 85,26 | 88,63 | n.a. |
| 2 | | 12,908 | 6,682 | 21,220 | 14,74 | 11,37 | n.a. |
| **Total:** | | | **45,326** | **186,623** | **100,00** | **100,00** | |

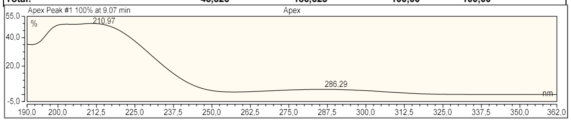


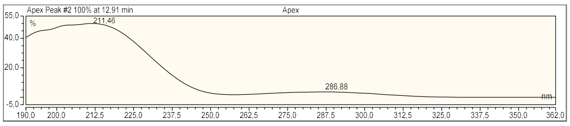

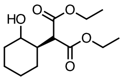

***trans-R*-R1** starting from ***R*-P1**

## Chromatogram and Results

| | | | |
|---|---|---|---|
| *Injection Name:* | **MIW_221_Hept_EtOH_98_2_1mlmin_25C_1uL_60min_IA-3** | *Run Time (min):* | **35,18** |
| *Vial Number:* | **GA5** | *Report Template:* | **MIW_137_CD** |
| *Injection Volume:* | **5,00** | *Channel:* | **UV_VIS_2** |
| *Column:* | **IA-3** | *Wavelength:* | **220,0** |
| *Instrument Method:* | **Heptane_EtOH_98 _2_1mLmin_90min_25 °C** | *B %:* | **0,0** |
| *Processing Method:* | **Long alcohol** | *C %:* | **0,0** |
| *Injection Date/Time:* | **26.Sep.23 14:43** | *D %:* | **2,0** |
| *Operator:* | **Irena Jelenkovic - Didic** | | |

**Chromatogram**

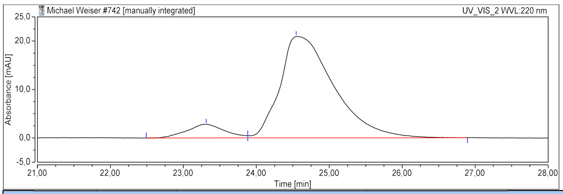


**Integration Results**

| No. | Peak Name | Retention Time min | Area mAU*min | Height mAU | Relative Area % | Relative Height % | Amount n.a. |
|---|---|---|---|---|---|---|---|
| 1 | | 23,313 | 1,683 | 2,822 | 8,58 | 11,89 | n.a. |
| 2 | | 24,547 | 17,937 | 20,913 | 91,42 | 88,11 | n.a. |
| **Total:** | | | **19,619** | **23,735** | **100,00** | **100,00** | |

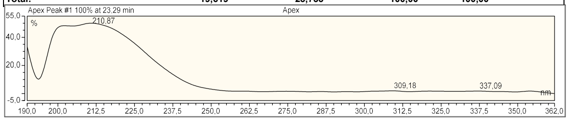


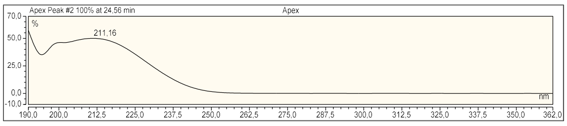


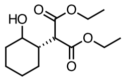

***trans-S*-R1** starting from ***S*-P1**

## Chromatogram and Results

| | | | |
|---|---|---|---|
| *Injection Name:* | **MIW_225_B_crude_Hept_EtOH_98_2_1mlmin_25C_1uL_60m** | *Run Time (min):* | **43,43** |
| *Vial Number:* | **GA4** | *Report Template:* | **MIW_137_CD** |
| *Injection Volume:* | **20,00** | *Channel:* | **UV_VIS_1** |
| *Column:* | **IA-3** | *Wavelength:* | **220,0** |
| *Instrument Method:* | **Heptane_EtOH_98 _2_1mLmin_60min_25 °C** | *B %:* | **0,0** |
| *Processing Method:* | **Long alcohol** | *C %:* | **0,0** |
| *Injection Date/Time:* | **05.Okt.23 15:16** | *D %:* | **2,0** |
| *Operator:* | **Irena Jelenkovic - Didic** | | |

**Chromatogram**

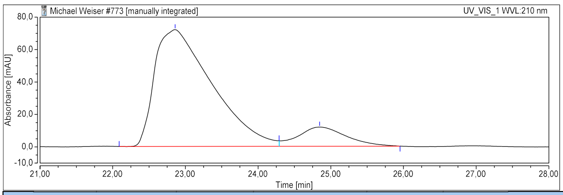


**Integration Results**

| No. | Peak Name | Retention Time min | Area mAU*min | Height mAU | Relative Area % | Relative Height % | Amount n.a. |
|---|---|---|---|---|---|---|---|
| 1 | | 22,858 | 66,070 | 71,997 | 87,95 | 85,88 | n.a. |
| 2 | | 24,847 | 9,051 | 11,836 | 12,05 | 14,12 | n.a. |
| **Total:** | | | **75,121** | **83,833** | **100,00** | **100,00** | |

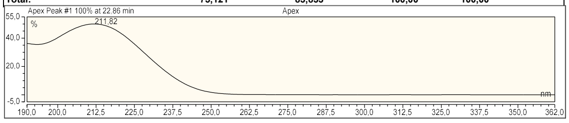


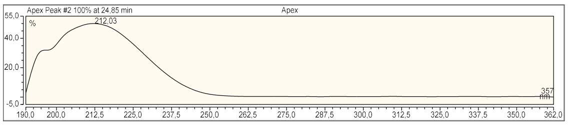


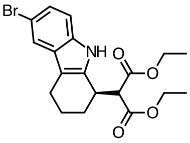

***R*-R2** starting from ***R*-P1**

## Chromatogram and Results

| | | | |
|---|---|---|---|
| *Injection Name:* | **MIW_225_C_crude_Hept_EtOH_98_2_1mlmin_25C_1uL_60m** | *Run Time (min):* | **20,70** |
| *Vial Number:* | **GA1** | *Report Template:* | **MIW_137_CD** |
| *Injection Volume:* | **10,00** | *Channel:* | **UV_VIS_1** |
| *Column:* | **IA-3** | *Wavelength:* | **220,0** |
| *Instrument Method:* | **Heptane_EtOH_98 _2_1mLmin_60min_25 °C** | *B %:* | **0,0** |
| *Processing Method:* | **Long alcohol** | *C %:* | **0,0** |
| *Injection Date/Time:* | **06.Okt.23 11:50** | *D %:* | **2,0** |
| *Operator:* | **Irena Jelenkovic - Didic** | | |

**Chromatogram**

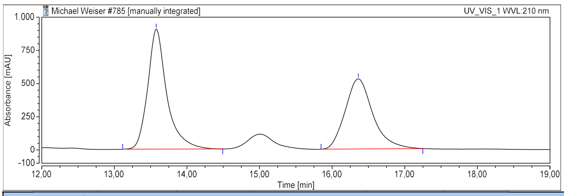


**Integration Results**

| No. | Peak Name | Retention Time min | Area mAU*min | Height mAU | Relative Area % | Relative Height % | Amount n.a. |
|---|---|---|---|---|---|---|---|
| 1 | | 13,577 | 274,826 | 904,411 | 54,06 | 63,11 | n.a. |
| 2 | | 16,360 | 233,521 | 528,629 | 45,94 | 36,89 | n.a. |
| **Total:** | | | **508,348** | **1433,040** | **100,00** | **100,00** | |

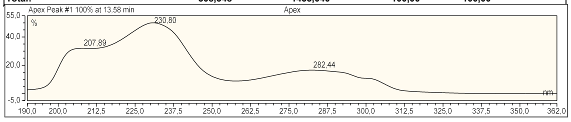


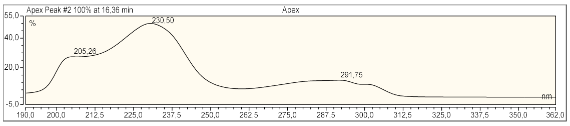


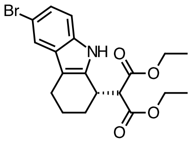

***S*-R2** starting from ***S*-P1**

## Chromatogram and Results

| | | | |
|---|---|---|---|
| *Injection Name:* | **MIW_223_A_Hept_EtOH_98_2_1mlmin_25C_1uL_60min_IA-3** | *Run Time (min):* | **24,58** |
| *Vial Number:* | **GA2** | *Report Template:* | **MIW_137_CD** |
| *Injection Volume:* | **5,00** | *Channel:* | **UV_VIS_1** |
| *Column:* | **IA-3** | *Wavelength:* | **220,0** |
| *Instrument Method:* | **Heptane_EtOH_98 _2_1mLmin_60min_25 °C** | *B %:* | **0,0** |
| *Processing Method:* | **Long alcohol** | *C %:* | **0,0** |
| *Injection Date/Time:* | **27.Sep.23 08:28** | *D %:* | **2,0** |
| *Operator:* | **Irena Jelenkovic - Didic** | | |

**Chromatogram**

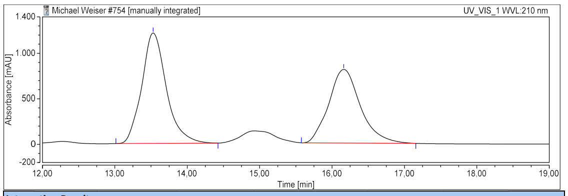


**Integration Results**

| No. | Peak Name | Retention Time min | Area mAU*min | Height mAU | Relative Area % | Relative Height % | Amount n.a. |
|---|---|---|---|---|---|---|---|
| 1 | | 13,528 | 477,267 | 1212,984 | 53,75 | 60,01 | n.a. |
| 2 | | 16,162 | 410,590 | 808,195 | 46,25 | 39,99 | n.a. |
| **Total:** | | | **887,857** | **2021,179** | **100,00** | **100,00** | |

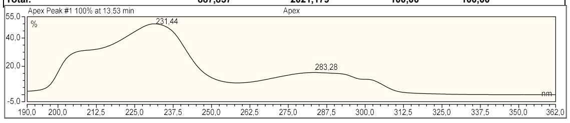


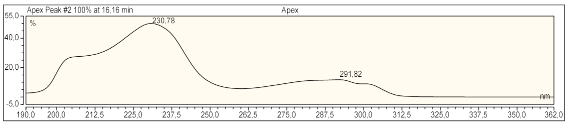

# 15. References


(1) Scharinger, F.; Márk Pálvölgyi, Á.; Zeindlhofer, V.; Schnürch, M.; Schröder, C.; Bica-Schröder, K. Counterion Enhanced Organocatalysis: A Novel Approach for the Asymmetric Transfer Hydrogenation of Enones. *ChemCatChem* **2020**, *12* (14), 3776-3782. DOI: 10.1002/cctc.202000414.

(2) Scharinger, F.; Pálvölgyi, Á. M.; Weisz, M.; Weil, M.; Stanetty, C.; Schnürch, M.; Bica-Schröder, K. Sterically Demanding Flexible Phosphoric Acids for Constructing Efficient and Multi-Purpose Asymmetric Organocatalysts. *Angew. Chem., Int. Ed.* **2022**, *61* (26), e202202189. DOI: 10.1002/anie.202202189.

(3) Wang, B. G.; Ma, B. C.; Wang, Q.; Wang, W. Superparamagnetic Nanoparticle-Supported (S)-Diphenyl- prolinol Trimethylsilyl Ether as a Recyclable Catalyst for Asymmetric Michael Addition in Water. *Adv. Synth. Catal.* **2010**, *352* (17), 2923-2928. DOI: 10.1002/adsc.201000508.

(4) Dalicsek, Z.; Pollreisz, F.; Soós, T. Efficient separation of a trifluoromethyl substituted organocatalyst: just add water. *Chem. Commun.* **2009**, (30), 4587-4589, 10.1039/B908967E. DOI: 10.1039/B908967E.

(5) Marigo, M.; Wabnitz, T. C.; Fielenbach, D.; Jørgensen, K. A. Enantioselective Organocatalyzed α Sulfenylation of Aldehydes. *Angew. Chem., Int. Ed.* **2005**, *44* (5), 794-797. DOI: 10.1002/anie.200462101.

(6) Wong, M. L. J.; Sterling, A. J.; Mousseau, J. J.; Duarte, F.; Anderson, E. A. Direct catalytic asymmetric synthesis of α-chiral bicyclo[1.1.1]pentanes. *Nat. Commun.* **2021**, *12* (1), 1644. DOI: 10.1038/s41467-021-21936-4.

(7) Liu, Y.; Kang, T.-R.; Liu, Q.-Z.; Chen, L.-M.; Wang, Y.-C.; Liu, J.; Xie, Y.-M.; Yang, J.-L.; He, L. Enantioselective [4 + 2] Cycloaddition of Cyclic N-Sulfimines and Acyclic Enones or Ynones: A Concise Route to Sulfamidate-Fused 2,6-Disubstituted Piperidin-4-ones. *Org. Lett.* **2013**, *15* (23), 6090-6093. DOI: 10.1021/ol402977w.

(8) Cassani, C.; Martín-Rapún, R.; Arceo, E.; Bravo, F.; Melchiorre, P. Synthesis of 9-amino(9-deoxy)epi cinchona alkaloids, general chiral organocatalysts for the stereoselective functionalization of carbonyl compounds. *Nat. Protoc.* **2013**, *8* (2), 325-344. DOI: 10.1038/nprot.2012.155.

(9) Svestka, D.; Otevrel, J.; Bobal, P. Asymmetric Organocatalyzed Friedel–Crafts Reaction of Trihaloacetaldehydes and Phenols. *Adv. Synth. Catal.* **2022**, *364* (13), 2174-2183. DOI: 10.1002/adsc.202200180.

(10) Varró, G.; Hegedűs, L.; Simon, A.; Balogh, A.; Grün, A.; Leveles, I.; Vértessy, B. G.; Kádas, I. The First Enantioselective Total Synthesis of (−)-trans-Dihydronarciclasine. *J. Nat. Prod.* **2017**, *80* (6), 1909-1917. DOI: 10.1021/acs.jnatprod.7b00208.

(11) Craig, R.; Litvajova, M.; Cronin, S. A.; Connon, S. J. Enantioselective acyl-transfer catalysis by fluoride ions. *Chem. Commun.* **2018**, *54* (72), 10108-10111, 10.1039/C8CC05692G. DOI: 10.1039/C8CC05692G.

(12) Hintermann, L.; Schmitz, M.; Englert, U. Nucleophilic Addition of Organometallic Reagents to Cinchona Alkaloids: Simple Access to Diverse Architectures. *Angew. Chem., Int. Ed.* **2007**, *46* (27), 5164-5167. DOI: 10.1002/anie.200701341.

(13) Jurberg, I. D. An Aminocatalyzed Stereoselective Strategy for the Formal α-Propargylation of Ketones. *Chem. Eur. J.* **2017**, *23* (41), 9716-9720. DOI: 10.1002/chem.201701433.

(14) Amewu, R.; O'Neill, P. M.; Stachulski, A.; Ellis, G.; Ward, S. A. DISPIRO TETRAOXANE COMPOUNDS. WO2008038030A2, April 3, 2008.

(15) Howell, J. M.; Feng, K.; Clark, J. R.; Trzepkowski, L. J.; White, M. C. Remote Oxidation of Aliphatic C–H Bonds in Nitrogen-Containing Molecules. *J. Am. Chem. Soc.* **2015**, *137* (46), 14590-14593. DOI: 10.1021/jacs.5b10299.
(16) Peschke, B.; Ankersen, M.; Sehested Hansen, B.; Kruse Hansen, T.; Langeland Johansen, N.; Lau, J.; Madsen, K.; Petersen, H.; Thøgersen, H.; Watson, B. Synthesis and in vitro characterization of new growth hormone secretagogues derived from ipamorelin with dipeptidomimetic N-terminals. *Eur. J. Med. Chem.* **1999**, *34* (5), 363-380. DOI: 10.1016/S0223-5234(99)80086-5.
(17) Fernandez-Bartolome, E.; Santos, J.; Gamonal, A.; Khodabakhshi, S.; McCormick, L. J.; Teat, S. J.; Sañudo, E. C.; Costa, J. S.; Martín, N. A Three-Dimensional Dynamic Supramolecular "Sticky Fingers" Organic Framework. *Angew. Chem., Int. Ed.* **2019**, *58* (8), 2310-2315. DOI: 10.1002/anie.201812419.
(18) Adimurthy, S.; Ramachandraiah, G.; Bedekar, A. V.; Ghosh, S.; Ranu, B. C.; Ghosh, P. K. Eco-friendly and versatile brominating reagent prepared from a liquid bromine precursor. *Green Chem.* **2006**, *8* (10), 916-922, 10.1039/B606586D. DOI: 10.1039/B606586D.
(19) Gai, K.; Fang, X.; Li, X.; Xu, J.; Wu, X.; Lin, A.; Yao, H. Synthesis of spiro[2.5]octa-4,7-dien-6-one with consecutive quaternary centers via 1,6-conjugate addition induced dearomatization of para-quinone methides. *Chem. Commun.* **2015**, *51* (87), 15831-15834, 10.1039/C5CC06287J. DOI: 10.1039/C5CC06287J.
(20) He, D.; Du, X.; Xiao, Z.; Ding, L. Methanofullerenes, C60(CH2)n (n = 1, 2, 3), as Building Blocks for High-Performance Acceptors Used in Organic Solar Cells. *Org. Lett.* **2014**, *16* (2), 612-615. DOI: 10.1021/ol4035275.
(21) Marx, L. B.; Burton, J. W. A Total Synthesis of Salinosporamide A. *Chem. Eur. J.* **2018**, *24* (26), 6747-6754. DOI: 10.1002/chem.201800046.
(22) de Alaniz, J. R.; Kerr, M. S.; Moore, J. L.; Rovis, T. Scope of the Asymmetric Intramolecular Stetter Reaction Catalyzed by Chiral Nucleophilic Triazolinylidene Carbenes. *J. Org. Chem.* **2008**, *73* (6), 2033-2040. DOI: 10.1021/jo702313f.
(23) Fuks, E.; Huber, L.; Schinkel, T.; Trapp, O. Investigation of Straightforward, Photoinduced Alkylations of Electron-Rich Heterocompounds with Electron-Deficient Alkyl Bromides in the Sole Presence of 2,6-Lutidine. *Eur. J. Org. Chem.* **2020**, *2020* (39), 6192-6198. DOI: 10.1002/ejoc.202001003.
(24) Roiban, G.-D.; Agudo, R.; Reetz, M. T. Cytochrome P450 Catalyzed Oxidative Hydroxylation of Achiral Organic Compounds with Simultaneous Creation of Two Chirality Centers in a Single C-H Activation Step. *Angew. Chem., Int. Ed.* **2014**, *53* (33), 8659-8663. DOI: 10.1002/anie.201310892.
(25) Schmid, M. B.; Zeitler, K.; Gschwind, R. M. The Elusive Enamine Intermediate in Proline-Catalyzed Aldol Reactions: NMR Detection, Formation Pathway, and Stabilization Trends. *Angew. Chem., Int. Ed.* **2010**, *49* (29), 4997-5003. DOI: 10.1002/anie.200906629.
(26) Melchiorre, P. Cinchona-based Primary Amine Catalysis in the Asymmetric Functionalization of Carbonyl Compounds. *Angew. Chem., Int. Ed.* **2012**, *51* (39), 9748-9770. DOI: 10.1002/anie.201109036.
(27) Cismesia, M. A.; Yoon, T. P. Characterizing chain processes in visible light photoredox catalysis. *Chem. Sci.* **2015**, *6* (10), 5426-5434, 10.1039/C5SC02185E. DOI: 10.1039/C5SC02185E.
(28) Carriazo, J. Inquiry-based experiment on the synthesis of K3[Fe(C2O4)3].3H2O. *Chemistry* **2010**, *19* (4), 103-112.

(29) Rabani, J.; Mamane, H.; Pousty, D.; Bolton, J. R. Practical Chemical Actinometry—A Review. *Photochem. Photobiol.* **2021**, *97* (5), 873-902. DOI: 10.1111/php.13429.
(30) Krause, L.; Herbst-Irmer, R.; Sheldrick, G. M.; Stalke, D. Comparison of silver and molybdenum microfocus X-ray sources for single-crystal structure determination. *J. Appl. Crystallogr.* **2015**, *48* (1), 3-10. DOI: 10.1107/s1600576714022985.
(31) Sheldrick, G. M. SHELXT – Integrated space-group and crystal-structure determination. *Acta Crystallogr., Sect. A: Found. Adv.* **2015**, *71* (1), 3-8. DOI: 10.1107/s2053273314026370.
(32) Sheldrick, G. M. Crystal structure refinement with SHELXL. *Acta Crystallogr., Sect. C: Struct. Chem.* **2015**, *71* (1), 3-8. DOI: 10.1107/s2053229614024218.
